# MkcDBGAS: a reference-free approach to identify comprehensive alternative splicing events in a transcriptome

**DOI:** 10.1093/bib/bbad367

**Published:** 2023-10-13

**Authors:** Quanbao Zhang, Lei Cao, Hongtao Song, Kui Lin, Erli Pang

**Affiliations:** MOE Key Laboratory for Biodiversity Science and Ecological Engineering and Beijing Key Laboratory of Gene Resource and Molecular Development, College of Life Sciences, Beijing Normal University, Beijing 100875, China; MOE Key Laboratory for Biodiversity Science and Ecological Engineering and Beijing Key Laboratory of Gene Resource and Molecular Development, College of Life Sciences, Beijing Normal University, Beijing 100875, China; MOE Key Laboratory for Biodiversity Science and Ecological Engineering and Beijing Key Laboratory of Gene Resource and Molecular Development, College of Life Sciences, Beijing Normal University, Beijing 100875, China; MOE Key Laboratory for Biodiversity Science and Ecological Engineering and Beijing Key Laboratory of Gene Resource and Molecular Development, College of Life Sciences, Beijing Normal University, Beijing 100875, China; MOE Key Laboratory for Biodiversity Science and Ecological Engineering and Beijing Key Laboratory of Gene Resource and Molecular Development, College of Life Sciences, Beijing Normal University, Beijing 100875, China

**Keywords:** alternative splicing, reference-free, mixed k-mers, colored de Bruijn graph, motifs

## Abstract

Alternative splicing (AS) is an essential post-transcriptional mechanism that regulates many biological processes. However, identifying comprehensive types of AS events without guidance from a reference genome is still a challenge. Here, we proposed a novel method, MkcDBGAS, to identify all seven types of AS events using transcriptome alone, without a reference genome. MkcDBGAS, modeled by full-length transcripts of human and *Arabidopsis thaliana*, consists of three modules. In the first module, MkcDBGAS, for the first time, uses a colored de Bruijn graph with dynamic- and mixed- kmers to identify bubbles generated by AS with precision higher than 98.17% and detect AS types overlooked by other tools. In the second module, to further classify types of AS, MkcDBGAS added the motifs of exons to construct the feature matrix followed by the XGBoost-based classifier with the accuracy of classification greater than 93.40%, which outperformed other widely used machine learning models and the state-of-the-art methods. Highly scalable, MkcDBGAS performed well when applied to Iso-Seq data of *Amborella* and transcriptome of mouse. In the third module, MkcDBGAS provides the analysis of differential splicing across multiple biological conditions when RNA-sequencing data is available. MkcDBGAS is the first accurate and scalable method for detecting all seven types of AS events using the transcriptome alone, which will greatly empower the studies of AS in a wider field.

## INTRODUCTION

Alternative splicing (AS) is a post-transcriptional process that regulates multi-exons genes to generate different transcripts with diverse exon structures by selection different splicing sites [1]. AS is widespread in eukaryotes [2, 3]. Recent studies have shown that AS plays important roles in biology processes [4–9], human immune disease [10, 11] and also provides an opportunity for rapid evolutionary innovation [12]. According to their generation mechanisms, so far seven AS types have been reported: exon skipping (ES), alternative acceptor site (AA), alternative donor site (AD), intron retention (IR), alternative first exons (AF), alternative last exons (AL) and mutually exclusive exons (MX) [13]. AS event types and amounts of AS vary substantially among eukaryotic lineages [14, 15].

RNA-sequencing (RNA-Seq) [16] greatly increases the ability of studies on AS [17], and isoform sequencing (Iso-Seq) [18] is a powerful tool to obtain a full-length transcriptome [19]. Based on these data, many methods have been proposed to detect AS at the genome-wide scale, such as rMATS [20], SUPPA2 [21] and AStool [22]. These methods greatly enriched the knowledge about AS in species with reference genomes. However, to date, many species have at least no well-annotated reference genomes, limiting the studies on AS in these species.

In recent years, several studies have tried to design algorithms to detect AS using only full-length transcripts. Liu *et al.* [23] first used BLAST [24], to detect AS transcripts from transcriptomes without guidance from references. Later, Ji *et al.* [25] developed AStrap to improve Liu’s method with two-round mapping by combining cluster and pairwise alignment using CD-HIT [26] and GMAP [27]. Ji *et al.* further classified AS events using a traditional machine learning model. Wang *et al.* [28] proposed IsoSplitter to identify AS transcripts from a transcriptome using SIM4 [29] without guidance from references. Cao *et al.* designed DeepASmRNA [30], using adjacent high-scoring segment pairs in the results of BLAST to greatly increase the recall while maintaining the precision. DeepASmRNA classified the AS events using an attention-based deep learning model. The shortcoming of the above methods is that they can only identify 4 types of AS, although IsoSplitter cannot be further classified. The overlooked AS events (i.e. AF, AL and MX), occupying more than half of AS events in human, are related to adaptation to selective pressure [31], regulation of differential expression [32], and human diseases [33].

**Figure 1:**
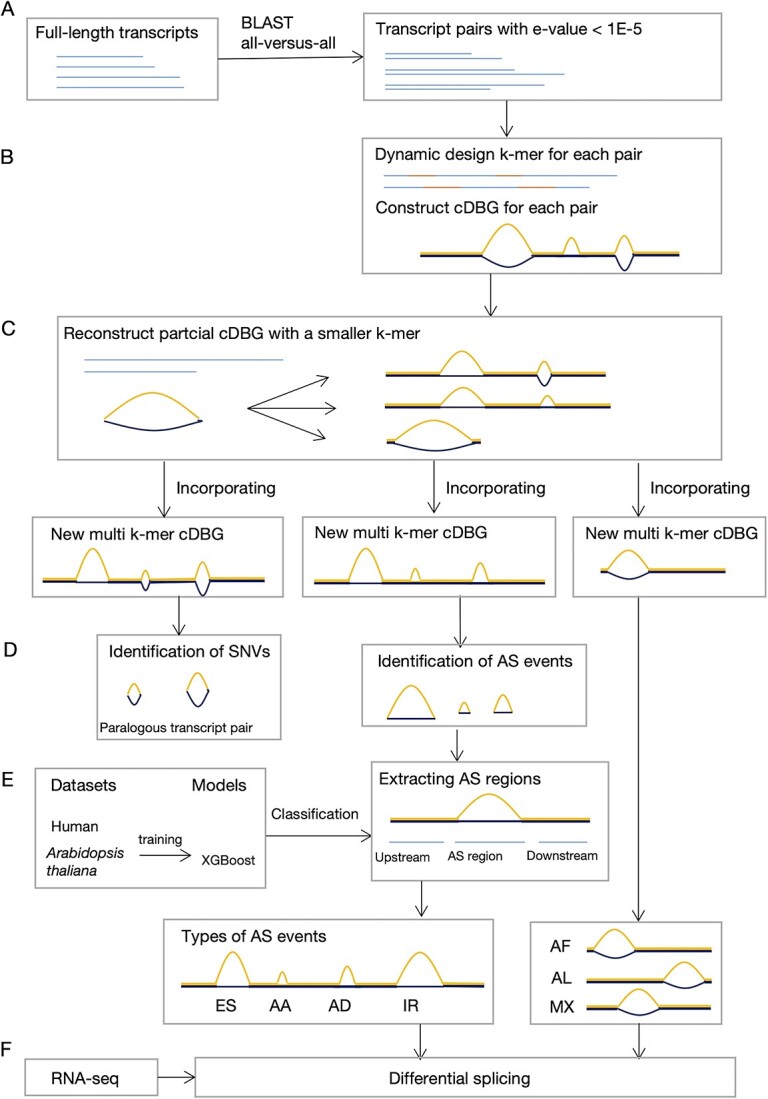
The workflow of MkcDBGAS. (**A**) All-versus-all alignment. (**B**) Constructing cDBGs and calling other-induced bubbles. A cDBG was constructed from two sequences using a *k-*mer. According to the topologies of bubbles, bubbles fall into three categories: SNV-induced, AS-induced and other-induced. (**C**) Reconstructing and incorporating sub-cDBGs. For each other-induced bubble, sequences of two arms were extracted to repeat the B step and obtained a sub-cDBG with a small *k′-*mer. All the sub-cDBGs were incorporated into the original cDBG by replacing the vertices and edges of the other-induced bubbles with new corresponding sub-cDBGs. (**D**) Calling bubbles. Three jobs are needed: (i) counting SNV-induced bubbles, (ii) identifying AF, AL and MX and (iii) identifying AS-induced bubbles. (**E**) Classification. AS events from human and *Arabidopsis thaliana* were used as training datasets to train two classifiers for four types of AS events based on XGBoost, respectively. (**F**) Analysis of differential splicing. When RNA seq data is available, we further conduct analysis of quantitative and differential splicing across multiple biological conditions.

The key idea of the above methods is to detect alternative regions using sequence alignments. The limitation of sequence alignments is that AF, AL and MX AS events cannot be detected. De Bruijn graph (DBG) [34] can potentially solve the limitation. DBG, using a set of nodes and edges to represent sequence, has been widely used in the genome and transcriptome assembly [35–39]. Notably, Iqbal *et al.* [40] introduced the colored DBG (cDBG) that extends the DBG with colored nodes and edges representing samples. In a cDBG, the variation between sequences appears as a bubble. Inspired by this idea, we presented MkcDBGAS for detecting AS events by calling AS-induced bubbles in cDBGs constructed by transcript pairs. To improve the precision, we proposed mixed *k*-mers cDBG to detect variants between transcripts.

To classify AS events, MkcDBGAS adopted AStrap’s approach by adding sequence motifs that affect splice site selection in different contexts [41]. Due to sparse matrix of features constructed with the sequence motifs, MkcDBGAS uses XGBoost [42], a novel sparsity-aware algorithm, to increase the accuracy of classification. By leveraging cDBG with mixed *k*-mers and XGBoost with added motif features, MkcDBGAS accurately predicts all seven types of AS on transcriptome-wide using only transcripts. In particular, MkcDBGAS can accurately detect AS in other species, meaning that it is scalable.

## MATERIALS AND METHODS

### Overview

**Figure 2:**
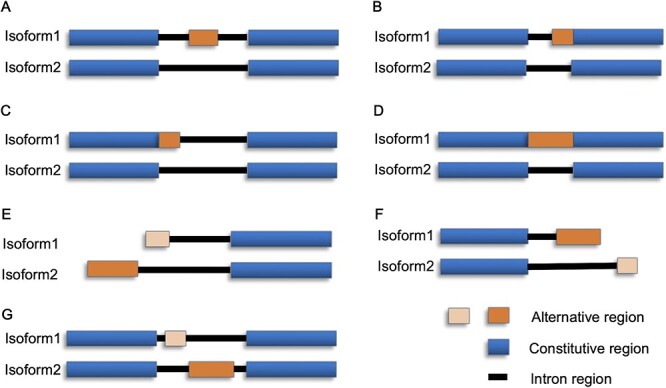
Seven types of AS events. (**A**) ES. (**B**) AA. (**C**) AD. (**D**) IR. (**E**) AF. (**F**) AL. (**G**) MX.

We developed MkcDBGAS that includes (i) identification module for identifying alternatively spliced transcripts via dynamic- and mixed-kmers cDBGs, (ii) classification module for distinguishing AS event types based on XGBoost [42] and (iii) differential splicing module for calculating percent-spliced-in (PSI) values and analysis of differential splicing across multiple biological conditions when RNA-Seq is available. Three steps are used to identify AS transcripts: alignments of transcripts, construction of cDBGs and calling bubbles (Figure 1A and D). The steps for the classification of AS events include extracting features, training the classification model and classifying AS events (Figure 1E). Seven types of AS can be identified: ES, AA, AD, IR, AF, AL and MX (Figure 2). Calculating PSI values and differential analysis were implemented in the differential splicing module (Figure 1F).

### Data preparation

Full-length transcripts of human and *Arabidopsis thaliana* were used to train and evaluate MkcDBGAS. Transcripts of mouse, RNA-Seq of human from two cell lines, and Iso-Seq data of *Amborella* were adopted to test MkcDBGAS (Supplementary Table S1). All datasets and the number of samples used in this work were listed in Supplementary Table S2.

### CDBGs construction and bubbles calling

Accurately detecting the alternative regions and their positions between alternatively spliced transcript pairs is the central task to detect AS. The bubbles with special topological structures in cDBG are the alternative regions. MkcDBGAS constructs cDBGs using transcript pairs. The graph is defined as G = (V, E, C), where V is the set of nodes representing *k*-mers that are dynamically determined by the transcript pairs; $\mathrm{E}=\left\{\left(\mathrm{u},\mathrm{v}\right),\mathrm{u},\mathrm{v}\in \mathrm{V}\ \right\}$is a set of directed edges connecting node u and v if the (*k* − 1)-suffix of u equals the (*k* − 1)-prefix of *v*; C is the color label indicating whether nodes come from the same transcript.

For each pair of transcripts with similar sequences (e-value = 1E – 10) identified by BLASTN [24], the minimum *k* was determined (Supplementary Methods Algorithm S1) as the length of the shortest nonrepeat sequences in the two transcript sequences to ensure that there was no cycle in the cDBG. Then, a cDBG was constructed using the *k-*mer, and bubbles were called (Supplementary Methods Algorithm S2). The bubbles are variant regions in two transcripts.

**Figure 3:**
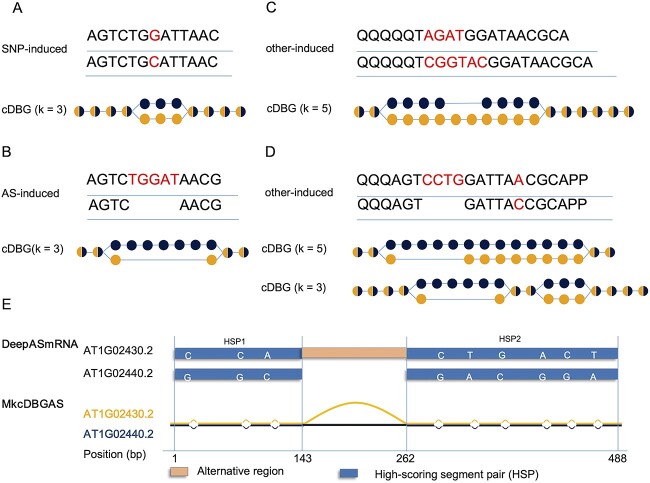
Topologies of bubbles in a cDBG. (**A**) The SNV-induced bubble. The topology of the bubble in the cDBG (*k* = 3) is that the lengths of both arms are equal to *k*. (**B**) The AS-induced bubble. The topology of the bubble in cDBG (*k* = 3) is that the length of the shorter arm is *k –* 1. (**C**) The other-induced bubble. For AF, AL and MX, the topology of the bubble in cDBG (*k* = 5) is that the length of both arms is larger than *k*. (**D**) The other-induced bubble harboring an SNV. In an other-induced bubble, if the distance of two adjacent bubbles is less than *k*, we can only observe one bubble. When a smaller *k′*-mer was used, two bubbles were observed. Letter ‘Q’ at the beginning and ‘P’ at the end of the sequence was used to ensure that the sequence pair had the same source and sink node. (**E**) Example of avoiding misidentification AS transcript pairs by identifying SNVs in MkcDBGAS. AT1G02430 (*ARFD1B*) and AT1G02440 (*ARFD1A*) are paralogous genes, AT1G02430.2 is a transcript from AT1G02430, and AT1G02440.2 is a transcript from AT1G02440. MkcDBGAS avoided misidentification by identifying 14 SNVs between AT1G02440.2 and AT1G02430.2.

According to the topology, the bubbles in the cDBG are divided into three categories: single nucleotide variants-induced (SNV-induced), AS-induced and other-induced, where for an SNV-induced bubble, the lengths of its two arms are *k* (Figure 3A); for an AS-induced bubble, the length of the shorter arm is *k* – 1 (Figure 3B); and for an other-induced bubble, the arms are both greater than *k* (Figure 3C and D).

### Reconstructing sub-cDBGs

The other-induced bubbles were complex. In one condition, the sequences of the two arms are different, such as AF or AL (Figure 3C). In another condition, two adjacent bubbles with a distance less than *k* will form one single bubble (Figure 3D). To illuminate detailed variants in the other-induced bubble, MkcDBGAS provides a novel solution by reconstructing sub-cDBG using *k′* smaller than *k* instead of using a single *k*. The *k′* was determined as the length of the shortest nonrepeat sequences in the two sequences of arms of the other-induced bubble. For each other-induced bubble, the two sequences of arms are used to reconstruct the sub-cDBG with *k′*-mers (Supplementary Methods Algorithm S3). Thus, the sub-cDBGs with *k′*-mers were obtained.

### Incorporating cDBGs and identifying alternatively spliced transcripts

We merged the cDBG and the sub-cDBGs into a new cDBG by replacing the other-induced bubbles with their corresponding sub-cDBGs. Thus, we obtained a new cDBG G*′* = (V*′*, K, E*′*, C), where V*′* is the set of nodes with mixed *k*-mers, and K is the set of *k*-mer sizes. ${\mathrm{E}}^{\prime }=\left\{\left({\mathrm{u}}^{\prime },{\mathrm{v}}^{\prime}\right),{\mathrm{u}}^{\prime },{\mathrm{v}}^{\prime}\in{\mathrm{V}}^{\prime}\right\}$the set of directed edges connecting u*′* and v*′* with the (*k* − 1)-suffix of u*′* equal to the (*k* − 1)-prefix of *v′* (*k*$\in \mathrm{K}$)*,* and C is the set of color labels same with the origin cDBG.

To detect AS events, we tracked the cDBG and queried AS-induced bubbles in the graph (Supplementary Methods Algorithm S4). For AF, AL and MX events, the criteria are (i) only one other-induced bubble in a cDBG, (ii) the arm length of the shorter transcript smaller than 30% of the length of the corresponding transcript and (iii) the bubble located at the beginning determined as AF, end as AL or middle as MX. For other AS events, the criteria are (i) one or more AS-induced bubbles, and (ii) less than three SNV-induced bubbles. The transcript pair with the AS event(s) was defined as alternatively spliced transcripts. In addition, MkcDBGAS can analyze differential splicing across multiple biological conditions when RNA-Seq is available (Supplementary Methods S1.1).

### Training datasets of AS events

**Figure 4:**
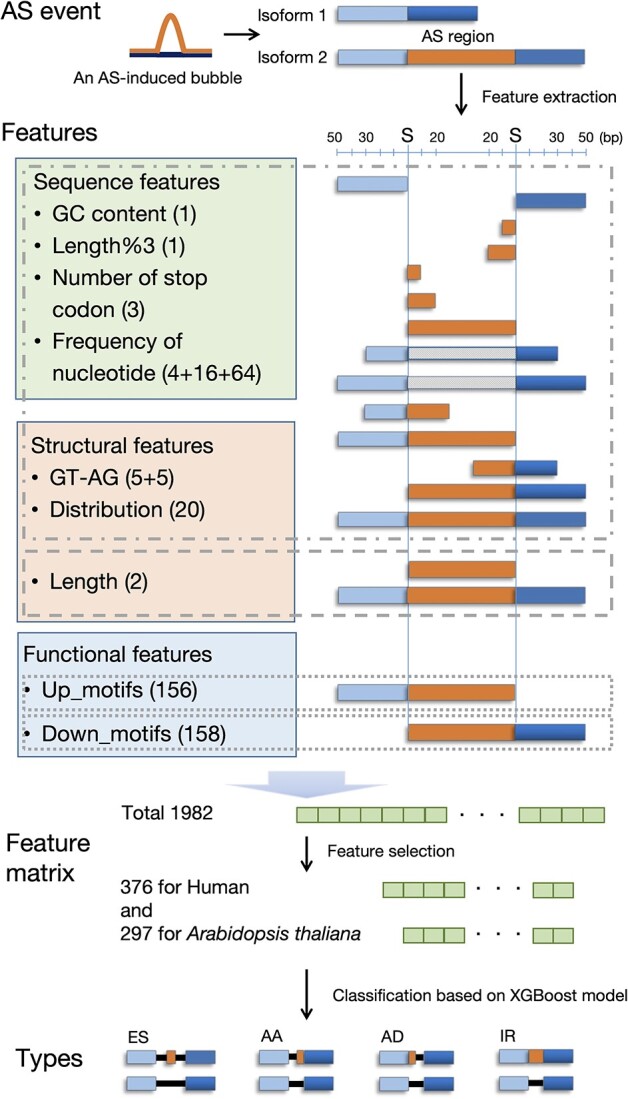
The workflow of the classification of AS events. For each AS event obtained by MkcDBGAS, three regions were extracted, including the AS region, upstream region, and downstream region. In total, 14 sequence around the splicing site were used to calculate the feature matrix, including sequence feature, structure feature, and functional feature. The XGBoost classifiers were trained based on human and *Arabidopsis thaliana* AS event datasets, respectively. The S on the axis represents the splicing sites and the gray box represents no sequences. Length%3: whether the length of the sequence is divisible by 3; GT-AG: whether there are ‘GT’, ‘GC’, ‘AT’, ‘AG’ and ‘AC’ at the 3 and 5 ends of the sequence; Up_motifs and Down_motifs: whether there are specific motifs in the upstream and downstream sequence of the splicing site.

To determine the other four AS event types, we trained a machine learning framework to classify AS events (Figure 4). To train different models for animals and plants, the function ‘generateEvents’ in SUPPA2 [21] with the default parameters was applied to the annotation file of human and *A. thaliana* for obtaining labeled AS events since SUPPA2 is suitable for the accurate, systematic and rapid genome-wide identification of all seven types of AS event from an annotation file. To ensure that there was no data leakage, only one event was retained when the above-mentioned sequences of several events are consistent. The final labeled datasets contained 39 860 ES, 16 623 AA, 15 159 AD and 7021 IR for human and 1051 ES, 4205 AA, 3156 AD and 5479 IR for *A. thaliana*.

Different from AStrap [25], our feature matrix includes flanking regions of splicing sites and added 314 AS motifs (Supplementary Table S3). We used alternative regions, upstream 50 bp, and downstream 50 bp to extract features, given that the sequences around the splicing sites are useful to classify AS events [43]. In addition, we added the features of 314 AS motifs from alternative regions and their flanking 50 bp regions, which were conversed splicing regulatory elements [41]. For each AS event, we obtained 14 different continuous sequences (Supplementary Methods S1.2). Then, we calculated features of the 14 sequences. Finally, a feature matrix with 1982-dimensional feature was constructed for each labeled AS event (Supplementary Table S4).

### Training classification models of AS events

XGBoost [42], a novel sparsity-aware algorithm, was adapted to handle the motif features that are quite sparse (Supplementary Methods S1.3). XGBoost was used to train models for human and *A. thaliana*. Training classification models were implemented with scikit-learn (v0.24.1) [44] including scaling of the feature matrix and feature selection based on ExtraTreesClassifier. Moreover, to ensure the reliability of the models, the Borderlinesmote [45], an oversampling method in the Python package imblearn (v 0.8.0), was used to keep samples of the four classes balanced. Then each labeled dataset was randomly divided into a training set and a test set at a ratio of 7:3 [44]. To optimize the hyperparameters of the XGBoost classifier, we used a grid search approach (Supplementary Methods S1.4). The 10-fold inner cross-validation was performed on the training set. Finally, the hyperparameters of each model were determined according to model performance in the training set.

### Comparing to other machine learning models

To find the most suitable model for classifying AS, we tried several widely used models of machine learning, including nearest neighbors [46], AdaBoost [47], SVM with linear kernel [48], SVM with RBF kernel [49], DecisionTree [50], Stochastic Gradient Descent (SGD) [51] and Random Forest [52]. Except for the classification model, other parameters (including feature selection, data balancing, training set and validation set) were consistent. The default hyperparameters were used to compare the performance of the models. Two datasets of human and *A. thaliana* were validated.

### Implementation of MkcDBGAS for Iso-Seq data of *Amborella*, transcripts of mouse and RNA-Seq data of human from two cell lines

MkcDBGAS was applied to the transcriptomes of *Amborella* and mouse. Since *Amborella* had no annotated file, PASA [53] was applied to full-length transcripts for obtaining the evaluation set. For mouse with an annotation file, SUPPA2 with default parameters [21] was used to obtain the evaluation set. MkcDBGAS was also applied to analyze differential splicing between RNA-Seq datasets of humans from two cell lines, PC3E and GS689 downloaded from previous research [20] (Supplementary Methods S1.5).

## RESULTS

We present MkcDBGAS, a new tool to predict AS from full-length transcripts without guidance from a reference genome (Figure 1). The inputs of MkcDBGAS are full-length transcripts. The three outputs of MkcDBGAS are (i) AS transcript pairs and the position of the AS occurred, (ii) AS event type and its probability and (iii) differential splicing if RNA-Seq is available.

### Accurate identification of alternatively spliced transcripts from transcriptomes

To build the ground truth dataset, we obtained 159 505 and 48 358 protein-coding transcripts in human and *A. thaliana*, respectively. SUPPA2 [21] generated 519 906 AS transcript pairs involving 190 107 AS events and 26 592 transcript pairs involving 15 488 AS events (Table 1) in human and *A. thaliana,* respectively. These AS transcript pairs and AS events were used as the ground truth datasets.

**Table 1:** Labeled data for human and *Arabidopsis thaliana*

Features	Human	*A. thaliana*
ES	40 055	1051
AA	16 727	4211
AD	15 209	3161
IR	7329	5992
AF	86 273	936
AL	18 923	106
MX	5591	31
Total AS events	190 107	15 488
AS transcript pairs	519 906	26 592

To evaluate MkcDBGAS, the above full-length protein-coding transcripts of human and *A. thaliana* were put into MkcDBGAS, respectively. MkcDBGAS predicted 494 910 AS transcript pairs for human, and 25 528 AS transcript pairs for *A. thaliana* (Table 2). The precision of MkcDBGAS was 98.17% and 99.31% in human and *A. thaliana*, respectively, and the recall reached 93.45% and 95.34% (Table 2). For AF, AL and MX, the precision was 95.33%, 94.37% and 93.38% in human, respectively (Table 2). Meanwhile, using the gene locus to verify the AS transcripts, the precision was 99.12% and 99.98% in human and *A. thaliana*, respectively.

**Table 2:** Performance of identifying AS transcript pairs using the results of SUPPA2 as the ground truth

		Human	*Arabidopsis thaliana*
AS transcript pairs	Predicted AS transcript pairs	494 910	25 528
	True positive	485 853	25 352
	Precision (%)	98.17	99.31
	Recall (%)	93.45	95.34
	F1-score	0.96	0.97
AF	Predicted AF	82 763	896
	True positive	78 898	870
	Precision (%)	95.33	97.12
	Recall (%)	91.45	92.93
	F1-score	0.93	0.95
AL	Predicted AL	17 928	102
	True positive	16 919	96
	Precision (%)	94.37	94.54
	Recall (%)	89.41	90.75
	F1-score	0.92	0.93
MX	Predicted MX	5422	30
	True positive	5063	28
	Precision (%)	93.38	94.53
	Recall (%)	90.56	91.27
	F1-score	0.92	0.93

**Figure 5:**
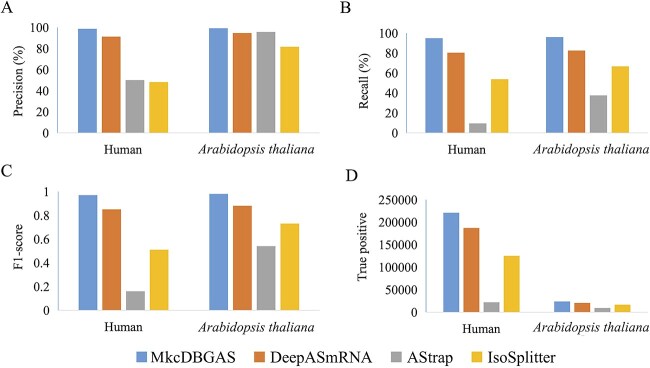
Performance of identifying AS transcript pairs among MkcDBGAS and existing methods taking the results of SUPPA2 as the ground truth. (**A**) Precision. (**B**) Recall. (**C**) F1-sore. (**D**) True positive.

To compare MkcDBGAS with AStrap, IsoSplitter and DeepASmRNA in identifying AS transcript pairs, only ES, AA, AD and IR events were used, since those methods could only identify four AS types, although IsoSplitter cannot be further classified. Overall, 232 908 and 24 964 AS transcript pairs involving the four types of AS events were obtained from the results of SUPPA2 in human and *A. thaliana,* respectively. For those transcript pairs, the precision of MkcDBGAS was 98.72% and 99.19% and the recall achieved 94.88% and 96.01% in human and *A. thaliana*, respectively (Figure 5 and Supplementary Table S5). These results indicated that MkcDBGAS outperformed AStrap, IsoSplitter and DeepASmRNA.

### A highly accurate landscape of AS classification

Identification module in MkcDBGAS can’t tell the type of ES, AA, AD and IR as the topology is too similar to be classified. XGBoost-based model was used to classify the four types only taking the sequences of AS regions and flanking regions as input (Figure 4). AS events from human and *A. thaliana* obtained by SUPPA2 were two labeled datasets. After 1982-dimensional feature matrix was obtained, feature selection was implemented by ExtraTreesClassifier [44]. We obtained 376 features for human (Supplementary Table S6) and 297 features for *A. thaliana* (Supplementary Table S7). Borderline-SMOTE [45] was used to balance the classes of AS in the labeled data. The results showed that the balanced dataset increased the accuracy (~3%) of the multiple classifiers for human and *A. thaliana* (Supplementary Table S8).

**Figure 6:**
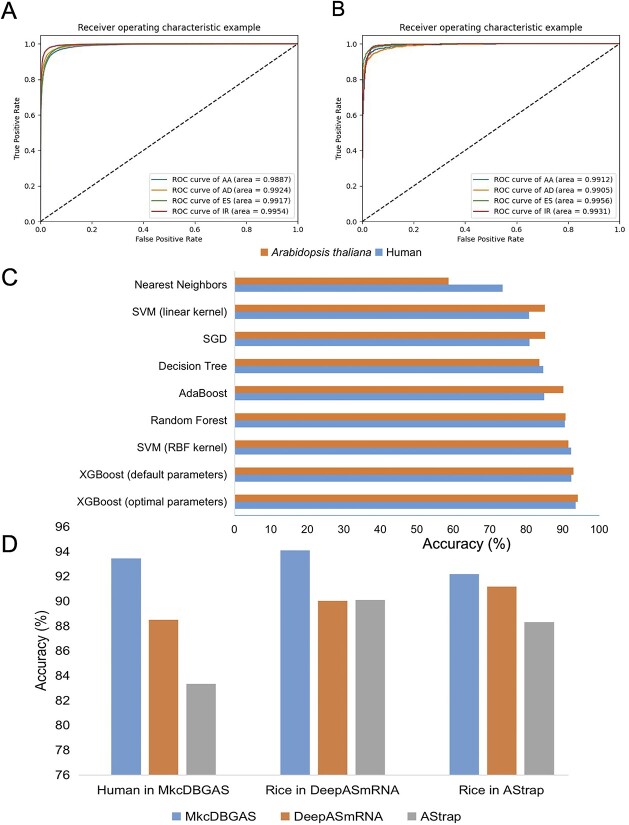
Performance of classifying four types of AS events. (**A**) ROC curves of human model in MkcDBGAS for classification of four types of AS events. (**B**) ROC curves of *Arabidopsis thaliana* model in MkcDBGAS for classification of four types of AS events. (**C**) Comparison of the accuracies of XGBoost and other widely used machine learning models. (**D**) Comparison of AS classification in accuracies among MkcDBGAS, DeepASmRNA and AStrap.

To better choose the hyperparameters of the XGBoost classifier, a grid search approach was performed by the GridSearchCV function in scikit-learn. The optimal hyperparameter combinations of XGBoost classifiers were colsample_bytree = 0.6, learning_rate = 0.2, max_depth = 8, min_child_weight = 4 and subsample = 0.8 for human (Supplementary Table S9) and colsample_bytree = 0.5, learning_rate = 0.2, max_depth = 9, min_child_ weight = 4 and subsample = 0.7 for *A. thaliana* (Supplementary Table S10). The accuracy reached 93.44% and 94.09% for the human and *A. thaliana* datasets, respectively (Table 3). The AUCs of our models were consistently higher than 0.99 (Figure 6A and B), highlighting the strong ability of classification of MkcDBGAS.

**Table 3:** Performance of mkcDBGAS in AS classification

Species	Metric	Overall	ES	AA	AD	IR
Human	Accuracy (%)	93.44				
	Precision (%)		90.80	93.28	95.22	94.47
	Recall (%)		92.01	90.54	93.45	97.71
	F1-score		0.91	0.92	0.94	0.96
	True positive	44 918	10 865	11 109	11 532	11 412
*Arabidopsis thaliana*	Accuracy (%)	94.09				
	Precision (%)		96.63	95.32	94.23	90.60
	Recall (%)		95.24	92.16	91.43	97.52
	F1-score		0.96	0.94	0.93	0.94
	True positive	6772	1688	1752	1683	1649

**Table 4:** Performance of mkcDBGAS in the mouse dataset

Models	Metrics	ES	AA	AD	IR	AF	AL	MX
Human	Precision (%)	92.21	93.32	90.96	94.68	99.06	97.66	97.23
	Recall (%)	91.36	90.59	92.2	94.36	92.71	90.13	89.74
	F1-score	0.92	0.92	0.92	0.95	0.96	0.94	0.93
	True positive	59 764	30 847	22 153	10 272	29 631	4966	3245

### Comparison with other widely used machine learning models

To compare the performance of our model with those of other widely used machine learning models, including nearest neighbors [46], AdaBoost [47], SVM with linear kernel [48], SVM with RBF kernel [49], Decision Tree [50], SGD [51] and Random Forest [52], we used the same training and test datasets as those in XGBoost in human and *A. thaliana*. The accuracy of MkcDBGAS based on XGBoost was the highest among the 8 models in human and *A. thaliana*, showing that MkcDBGAS outperformed the other machine learning models (Figure 6C, Supplementary Table S11), highlighting the power of XGBoost models in the classification of AS events.

### Comparison with previous methods

The performance of classification module in MkcDBGAS was competed to the state-of-the-art methods: AStrap and DeepASmRNA. In AStrap, two models, human and rice, were reported. In DeepASmRNA, three models, human, *A. thaliana*, and rice, were proposed. In MkcDBGAS, two models, human and *A. thaliana,* were provided. To compare the performance of the three tools, three different datasets were used: the human test dataset in MkcDBGAS, the rice test dataset in DeepASmRNA, and the rice test dataset in AStrap. The *A. thaliana* model in MkcDBGAS was used to classify AS events of rice. MkcDBGAS improved performance by ~1–6% over AStrap and DeepASmRNA in three datasets (Figure 6D, Supplementary Table S12).

### Implementation of MkcDBGAS in full-length transcripts of mouse and human from two cell lines

To estimate the scalability, MkcDBGAS was applied to the transcriptome of mouse. The full-length transcripts and gene annotation file of mouse were downloaded from GENCODE. SUPPA2 [21] with the default parameters generated 275 788 transcript pairs involving 175 449 AS events, including 65 416 ES, 34 051 AA, 24 027 AD, 10 886 IR, 31 942 AF, 5510 AL and 3617 MX.

Then, the full-length transcripts were input into MkcDBGAS using the human model. At the transcript pair level, MkcDBGAS identified 263 845 AS transcript pairs, including all seven types of AS events, in which 259 544 AS transcript pairs overlapped with results of SUPPA2. The precision and recall were 98.37% and 94.11%, respectively. At the AS event level, the precision of each type of AS event was ≥90.96%, and the recall of each type of AS event was ≥89.74% (Table 4), indicating that the human model was suitable for implementation in animals, which have a similar distribution of AS events as humans.

To apply the differential splicing module of MkcDBGAS, full-length transcripts, assembled by RNA-Seq datasets from two cell lines, PC3E and GS689 downloaded from rMATS [20] (Supplementary Methods S1.5), were put into MkcDBGAS for identifying AS, classifying using the human model, and analyzing differential splicing between the two cell lines (Supplementary Methods S1.6). Meanwhile, when we used the results of rMATS to test MkcDBGAS, MkcDBGAS performed well (Supplementary Methods S1.7). In addition, we used the 34 ES events obtained through RT-PCR from the PC3E cell line downloaded from the previous research [20] for experimental validation. The strict criteria were carried out: (i) the start and end position of alternative regions were completely consistent, and (ii) the type of AS must be an ES event. Only when both of the above criteria were met, an AS event was considered a true positive event. Finally, we found 31 of the 34 events were completely consistent with those of our prediction, indicating that the precision of method based on this experimental dataset is 91.2% (31/34) (Supplementary Table S13).

### Implementation of MkcDBGAS in the Iso-Seq data of *Amborella*

MkcDBGAS was also applied to the Iso-Seq data of *Amborella*. Full-length transcripts and AS events of *Amborella* were downloaded from DeepASmRNA [30], involving 1973 AS transcript pairs and 3312 AS events containing 325 ES, 1236 AA, 444 AD and 1307 IR. The 8918 full-length transcripts were put into MkcDBGAS using the model of *A. thaliana.* Sequencing errors of third-generation sequencing were considered through a parameter—seqtype. MkcDBGAS outputted 2117 AS transcript pairs and 3628 AS events. These AS events included 311 AF, 54 AL and 19 MX except for 329 ES, 1258 AA, 456 AD and 1221 IR (Table 5).

**Table 5:** The performance of mkcDBGAS in the Iso-Seq data of *Amborella*

Method	Metrics	ES	AA	AD	IR	All
PASA	AS events	325	1236	444	1307	3312
mkcDBGAS	AS events	329	1258	456	1221	3244
	True positive	312	1163	416	1192	3029
	Precision (%)	94.89	92.41	91.32	97.61	94.45
	Recall (%)	96.13	94.12	93.91	91.21	93.08
	F1-score	0.96	0.93	0.93	0.94	0.94
DeepASmRNA	True positive	281	852	361	1128	2622
	Precision (%)	75.54	84.02	74.59	94.95	85.74
	Recall (%)	86.46	68.93	81.31	86.3	79.17
	F1-score	0.81	0.76	0.78	0.90	0.82
	AS events	139	66	90	465	760
AStrap	True positive	109	47	57	441	654
	Precision (%)	78.42	71.21	63.33	94.84	86.05
	Recall (%)	33.54	3.80	12.84	33.74	22.95
	F1-score	0.47	0.07	0.21	0.50	0.36

Considering there were only ES, IR, AA and AD events, we only assessed these four types of AS events, involving 1859 AS transcript pairs and 3244 AS events detected by MkcDBGAS. At the transcript pair level, 1835 alternatively spliced transcript pairs overlapped with the results of PASA, meaning that the precision and recall were 98.71% and 93.02%, respectively. At the event level, in the shared 1835 transcript pairs, we predicted 3244 AS events in which 3029 AS events were the same as the results of PASA, suggesting that the precision and recall were 94.5% and 93.08%, respectively. These results showed that MkcDBGAS was robust when applying the model of *A. thaliana* to Iso-Seq of *Amborella*, suggesting that the model of *A. thaliana* was suitable for analyzing the transcript from other plants.

### Computation time and memory

The runtime and memory were assessed among MkcDBGAS, MkcDBGAS in parallel processing, DeepASmRNA, AStrap and IsoSplitter using the full-length transcripts of *Amborella.* All programs were run on an Inspur TS860M5 server with CentOS 7.5 Linux operating system [CPU: Intel(R) Xeon(R) E5-2620 v4 @ 2.10GHz, 1 TB RAM] allocating 192 threads maximum. The runtime of MkcDBGAS is significantly reduced in a multi-threaded mode compared to a single-threaded mode, but consumed more memory (Table 6). Moreover, MkcDBGAS outperformed AStrap and IsoSplitter both in runtime and memory, but did not perform as well as DeepASmRNA in runtime.

**Table 6:** Comparison of runtime and memory among mkcDBGAS, DeepASmRNA, AStrap and IsoSplitter using the *Amborella* dataset

Methods	Modules	Runtime	Memory
mkcDBGAS (parallel 10 threads)	Identification	2 min 11 s	372 M
	Classification	29 s	246 M
mkcDBGAS	Identification	19 min	43 M
	Classification	29 s	246 M
DeepASmRNA	Identification	32 s	282 M
	Classification	9 s	378 M
AStrap	Identification	3 min 16 s	460 M
	Classification	31 s	1240 M
IsoSplitter	Identification	67 min	2662 M

## DISCUSSION

We developed MkcDBGAS, a novel approach to identify all seven types of AS events from a transcriptome without relying on reference genomes. MkcDBGAS, leveraging a mixed *k*-mers cDBGs, improves the detection of three overlooked types of AS events (AF, AL and MX). Our method outperformed the state-of-the-art methods in both the identification of AS transcripts and the classification of AS events. Moreover, MkcDBGAS showed robust performance when applying trained models to other organisms.

MkcDBGAS shows advantages over sequence alignment tools in precision of AS identification by distinguishing between AS transcripts and transcripts from separate paralogous genes. The key differences are single nucleotide variants (SNVs) and small insertion/deletion (InDels), which are not easily captured by the alignments of sequences. However, in MkcDBGAS, SNVs were identified by the topology of bubble, which cannot occur in AS transcripts from the same gene. Therefore, using SNVs to distinguish AS transcripts from transcripts of paralogous genes effectively improved the precision of AS identification. For instance, AT1G02430 (*ARFD1B*) and AT1G02440 (*ARFD1A*) are paralogous genes, MkcDBGAS avoided the misidentification by identifying 14 SNVs between AT1G02430.2 and AT1G02440.2 (Figure 3E).

MkcDBGAS outperformed the state-of-the-art methods in AS classification. The addition of functional features and the using XGBoost-based classifier improve performance than AStrap. Functional features include 314 motifs that are conservative splicing regulatory elements and highly related to the selection of splicing sites. These motifs are enriched near the selective splicing exon, which is close to the binding sites of almost all known tissue-specific selective splicing factors [41]. Compared with the model trained by the feature matrix without functional features, the accuracy of the XGBoost model trained by the feature matrix with functional features increased by 3% (Figure S1, Supplementary Table S14).

Feature importance analysis revealed ‘asseqacceptorGT’ and ‘asseqdonerAG’ as the shared features in the top 10 significant baseline features in the two classification models, meaning that ‘Is there GT in acceptor of AS region sequence’ and ‘Is there AG in donor of AS region sequence’ were important for our models. Therefore, the GT-AG rule is important for our classification models. The result also conforms to the mechanism of AS events: the variable splicing regions of the four types of events belong to different fragments of introns, where an IR event involves the complete intron, an AA event contains the 5′ end of the intron, an AD event contains the 3′ end of the intron. In addition, the motif ‘GTGAG’ was an important feature in training the human classification model, implying that the existence of the motif is also helpful for classifying the four types of AS events.

The performance of DeepASmRNA and MkcDBGAS also showed that when there is little prior knowledge of a research question, deep learning [54] may be the optimal choice, while complete prior knowledge has been accumulated, and machine learning algorithms may be better [55].

Sequencing errors of third-generation sequencing were considered through a parameter named—seqtype in the application of Iso-Seq. Over 98% of the third-generation sequencing errors were 1–2 bp gaps and 1–2% errors were single nucleotide errors [56]. For 1–2 bp gaps, MkcDBGAS ignored bubbles with arm lengths less than *k* + 2. For single nucleotide errors, MkcDBGAS increased the number of SNVs to 5 (default 2) to enhance the robustness when distinguishing AS transcripts from transcripts of homologous genes.

MkcDBGAS also has limitations. It is sensitive to single nucleotide errors. Now, we arbitrarily defined the tolerant number of single nucleotide errors (default 2 and 5 for third-generation sequencing). In the future, a dynamic tolerant number of single nucleotide errors according to the sequencing platforms and sequencing technologies should be made.

## CONCLUSIONS

MkcDBGAS is an accurate and scalable method for predicting all the seven types of AS events using a transcriptome data alone, which is a step toward investigating AS transcriptome-wide in species without well-annotated reference genomes. This method will greatly empower the studies of AS in a species without a well-annotated genome and further help understand the mechanism of AS and how AS contributes to protein function diversification.

**Key Points**

- MkcDBGAS is a reference-free predictor of all seven types of AS events in a transcriptome.
- MkcDBGAS is the first attempt to implement colored de Bruijn graphs with mixed k-mers to detect variants between transcripts.
- MkcDBGAS is the first attempt to use motif features to construct the feature matrix for the classification of AS events.
- MkcDBGAS integrate the differential splicing module for the analysis of differential splicing across multiple biological conditions when RNA-Seq is available.

## Supplementary Material

Supplementary_Tables_bbad367

Supplementary_Methods_bbad367

## Data Availability

All datasets and codes used in this study are available at GitHub: https://github.com/CMB-BNU/mkcDBGAS and the website of our lab: http://cmb.bnu.edu.cn/mkcDBGAS/index.php/download.

## Notes

**Quanbao Zhang** is a PhD student at Beijing Normal University under Prof. Erli Pang. His research focuses on alternative splicing and machine learning.

**Lei Cao** is a master student at Beijing Normal University under Prof. Erli Pang. His research focuses on deep learning.

**Hongtao Song** is an associate professor at Beijing Normal University. His research focuses on conserved non-coding elements.

**Kui Lin** is a professor at Beijing Normal University. His research focuses on developing statistical methods for the analysis of omics.

**Erli Pang** is a professor at Beijing Normal University. Her research focuses on the variation of multi-omic data, positive selection, and conserved non-coding elements.
