# Supplementary material for: MkcDBGAS: a reference-free approach to identify comprehensive alternative splicing events in a transcriptome
Source: Brief Bioinform. 2023 Oct 13;24(6):bbad367. doi: 10.1093/bib/bbad367 (PMC10576019; doi:10.1093/bib/bbad367)
Supplement: Supplementary_Tables_bbad367 [file supplementary_tables_bbad367.docx]

# MkcDBGAS: a reference-free approach to identify comprehensive alternative splicing events in a transcriptome

Quanbao Zhang^1^, Lei Cao^1^, Hongtao Song^1^, Kui Lin^1^ and Erli Pang^1*^

^1^ MOE Key Laboratory for Biodiversity Science and Ecological Engineering and Beijing Key Laboratory of Gene Resource and Molecular Development, College of Life Sciences, Beijing Normal University, Beijing 100875, China

*To whom correspondence should be addressed

Erli Pang, College of Life Sciences, Beijing Normal University, No 19 Xinjiekouwai Street, Beijing, 100875, China

Email: [pangerli@bnu.edu.cn](mailto:pangerli@bnu.edu.cn)

Table S1 Data source used by MkcDBGAS

| Species | Data | Version | Download website |
| --- | --- | --- | --- |
| Human | genome | GRCh38.p13 | https://www.gencodegenes.org/human/release_37.html |
|  | annotation file | v37 |  |
|  | RNA-seq | PC3E | https://www.ncbi.nlm.nih.gov/sra/SRX174803 |
|  | RNA-seq | GS689 | https://www.ncbi.nlm.nih.gov/sra/SRX174805 |
| mice | genome | release M31 | https://www.gencodegenes.org/mouse/release_M31.html |
|  | annotation file | release M31 |  |
| *Arabidopsis thaliana* | genome | TAIR10 | https://www.arabidopsis.org |
|  | annotation file | Araport11 |  |
| *Amborella* | genome | release v1 | http://cmb.bnu.edu.cn/DeepASmRNA/index.php/download |
|  | Iso-Seq data | release v1 |  |

Table S2 All datasets and the number of samples used in this work

| Usage | Species | Dataset | The number of AS events |
| --- | --- | --- | --- |
| Models training and testing | Human | AS events obtained by SUPPA | 79,320 |
|  |  | Non-redundant dataset | 78,663 |
|  |  | Balanced dataset | 160,220 |
|  |  | Training dataset | 112,154 |
|  |  | Test dataset | 48,066 |
|  | *Arabidopsis thalian* | AS events obtained by SUPPA | 14,415 |
|  |  | Non-redundant dataset | 13,891 |
|  |  | Balanced dataset | 23,970 |
|  |  | Training dataset | 16,779 |
|  |  | Test dataset | 7,191 |
| Comparing with the previous methods | Human | The human test dataset in MkcDBGAS | 48,066 |
|  | Rice | The rice test dataset downloaded from DeepASmRNA | 2,267 |
|  | Rice | The rice test dataset downloaded from AStrap | 8,455 |
| Implementation of MkcDBGAS | Human | AS events from GS689 cell lines obtained by rMATS | 160,475 |
|  | Human | AS events from PC3E cell line obtained by rMATS | 164,286 |
|  | *Amborella* | AS events from Iso-Seq data obtained by PASA | 3,312 |
|  | Mouse | AS events obtained by SUPPA | 175,449 |
| Experimental validation | Human | AS events obtained by RT-PCR from the previous research [1] | 34 |

Table S3 Motifs related to alternative splicing events

| No. | Motifs | Description |
| --- | --- | --- |
| D1 | GTAAC | motif GTAAC in downstream of splicing site |
| D2 | AAGTGT | motif AAGTGT in downstream of splicing site |
| D3 | GTTTGT | motif GTTTGT in downstream of splicing site |
| D4 | ATTAACA | motif ATTAACA in downstream of splicing site |
| D5 | TGAAG | motif TGAAG in downstream of splicing site |
| D6 | TAACC | motif TAACC in downstream of splicing site |
| D7 | TTGAAAT | motif TTGAAAT in downstream of splicing site |
| D8 | AATTG | motif AATTG in downstream of splicing site |
| D9 | CTGCT | motif CTGCT in downstream of splicing site |
| D10 | TTTATG | motif TTTATG in downstream of splicing site |
| D11 | TGATAAA | motif TGATAAA in downstream of splicing site |
| D12 | ATGTTT | motif ATGTTT in downstream of splicing site |
| D13 | TTTCCAA | motif TTTCCAA in downstream of splicing site |
| D14 | AAGTC | motif AAGTC in downstream of splicing site |
| D15 | AAAGA | motif AAAGA in downstream of splicing site |
| D16 | GTACGT | motif GTACGT in downstream of splicing site |
| D17 | GTTAAA | motif GTTAAA in downstream of splicing site |
| D18 | GAGCTG | motif GAGCTG in downstream of splicing site |
| D19 | TCATTTT | motif TCATTTT in downstream of splicing site |
| D20 | TGCATG | motif TGCATG in downstream of splicing site |
| D21 | TTCTT | motif TTCTT in downstream of splicing site |
| D22 | TTTATC | motif TTTATC in downstream of splicing site |
| D23 | ACATTT | motif ACATTT in downstream of splicing site |
| D24 | TGCCAGC | motif TGCCAGC in downstream of splicing site |
| D25 | ATAATT | motif ATAATT in downstream of splicing site |
| D26 | GTAGG | motif GTAGG in downstream of splicing site |
| D27 | GTATCCT | motif GTATCCT in downstream of splicing site |
| D28 | CATTTG | motif CATTTG in downstream of splicing site |
| D29 | ACTAAC | motif ACTAAC in downstream of splicing site |
| D30 | TTTCAG | motif TTTCAG in downstream of splicing site |
| D31 | AATTGA | motif AATTGA in downstream of splicing site |
| D32 | TTAGCA | motif TTAGCA in downstream of splicing site |
| D33 | CAAAT | motif CAAAT in downstream of splicing site |
| D34 | TAATG | motif TAATG in downstream of splicing site |
| D35 | TTTTGAT | motif TTTTGAT in downstream of splicing site |
| D36 | AGAAAT | motif AGAAAT in downstream of splicing site |
| D37 | TTTCTA | motif TTTCTA in downstream of splicing site |
| D38 | TATTTC | motif TATTTC in downstream of splicing site |
| D39 | TAACT | motif TAACT in downstream of splicing site |
| D40 | TGAGG | motif TGAGG in downstream of splicing site |
| D41 | TAAAAT | motif TAAAAT in downstream of splicing site |
| D42 | TTTATA | motif TTTATA in downstream of splicing site |
| D43 | TATCCT | motif TATCCT in downstream of splicing site |
| D44 | GTTAGT | motif GTTAGT in downstream of splicing site |
| D45 | TTTACAG | motif TTTACAG in downstream of splicing site |
| D46 | TATTTG | motif TATTTG in downstream of splicing site |
| D47 | GTACTGT | motif GTACTGT in downstream of splicing site |
| D48 | TTAAG | motif TTAAG in downstream of splicing site |
| D49 | CATAAA | motif CATAAA in downstream of splicing site |
| D50 | GCATG | motif GCATG in downstream of splicing site |
| D51 | TGATTA | motif TGATTA in downstream of splicing site |
| D52 | TTTTAAA | motif TTTTAAA in downstream of splicing site |
| D53 | CTGACT | motif CTGACT in downstream of splicing site |
| D54 | ACTAAT | motif ACTAAT in downstream of splicing site |
| D55 | GAGTA | motif GAGTA in downstream of splicing site |
| D56 | TCTTAA | motif TCTTAA in downstream of splicing site |
| D57 | TTGGTT | motif TTGGTT in downstream of splicing site |
| D58 | ATATTT | motif ATATTT in downstream of splicing site |
| D59 | AGAGCCA | motif AGAGCCA in downstream of splicing site |
| D60 | TCTTT | motif TCTTT in downstream of splicing site |
| D61 | AGTTTT | motif AGTTTT in downstream of splicing site |
| D62 | GTATTT | motif GTATTT in downstream of splicing site |
| D63 | TCAGA | motif TCAGA in downstream of splicing site |
| D64 | TAAGT | motif TAAGT in downstream of splicing site |
| D65 | AAGCA | motif AAGCA in downstream of splicing site |
| D66 | TTCACAG | motif TTCACAG in downstream of splicing site |
| D67 | AGTAA | motif AGTAA in downstream of splicing site |
| D68 | TCTGG | motif TCTGG in downstream of splicing site |
| D69 | AGCTTT | motif AGCTTT in downstream of splicing site |
| D70 | TGATTTG | motif TGATTTG in downstream of splicing site |
| D71 | TTTTGC | motif TTTTGC in downstream of splicing site |
| D72 | TAGAAA | motif TAGAAA in downstream of splicing site |
| D73 | GTGAG | motif GTGAG in downstream of splicing site |
| D74 | TTCTGT | motif TTCTGT in downstream of splicing site |
| D75 | GTAAG | motif GTAAG in downstream of splicing site |
| D76 | ATGAAA | motif ATGAAA in downstream of splicing site |
| D77 | AGAAAA | motif AGAAAA in downstream of splicing site |
| D78 | TGAGC | motif TGAGC in downstream of splicing site |
| D79 | TGGCTT | motif TGGCTT in downstream of splicing site |
| D80 | TTAATCT | motif TTAATCT in downstream of splicing site |
| D81 | AATTAT | motif AATTAT in downstream of splicing site |
| D82 | TGGAAAT | motif TGGAAAT in downstream of splicing site |
| D83 | CCACAG | motif CCACAG in downstream of splicing site |
| D84 | AAATGA | motif AAATGA in downstream of splicing site |
| D85 | GCAAGT | motif GCAAGT in downstream of splicing site |
| D86 | GTAAAA | motif GTAAAA in downstream of splicing site |
| D87 | GTCTG | motif GTCTG in downstream of splicing site |
| D88 | AAATGT | motif AAATGT in downstream of splicing site |
| D89 | TGCAT | motif TGCAT in downstream of splicing site |
| D90 | GAGAAA | motif GAGAAA in downstream of splicing site |
| D91 | TTAGA | motif TTAGA in downstream of splicing site |
| D92 | TTTATAA | motif TTTATAA in downstream of splicing site |
| D93 | GTTTT | motif GTTTT in downstream of splicing site |
| D94 | GCTTGGC | motif GCTTGGC in downstream of splicing site |
| D95 | TAAGC | motif TAAGC in downstream of splicing site |
| D96 | GTATG | motif GTATG in downstream of splicing site |
| D97 | AAATT | motif AAATT in downstream of splicing site |
| D98 | GTAAT | motif GTAAT in downstream of splicing site |
| D99 | TTCTCT | motif TTCTCT in downstream of splicing site |
| D100 | TGAGAA | motif TGAGAA in downstream of splicing site |
| D101 | TTAGTT | motif TTAGTT in downstream of splicing site |
| D102 | TAAGG | motif TAAGG in downstream of splicing site |
| D103 | TGTTTAA | motif TGTTTAA in downstream of splicing site |
| D104 | GTCAGT | motif GTCAGT in downstream of splicing site |
| D105 | AGAATT | motif AGAATT in downstream of splicing site |
| D106 | TAAATG | motif TAAATG in downstream of splicing site |
| D107 | AATTCA | motif AATTCA in downstream of splicing site |
| D108 | TCCTTT | motif TCCTTT in downstream of splicing site |
| D109 | TAAGA | motif TAAGA in downstream of splicing site |
| D110 | AAATCA | motif AAATCA in downstream of splicing site |
| D111 | TAATTTG | motif TAATTTG in downstream of splicing site |
| D112 | GAAATA | motif GAAATA in downstream of splicing site |
| D113 | TGGTTT | motif TGGTTT in downstream of splicing site |
| D114 | TGTTAA | motif TGTTAA in downstream of splicing site |
| D115 | TGTCT | motif TGTCT in downstream of splicing site |
| D116 | GTTGGT | motif GTTGGT in downstream of splicing site |
| D117 | TGAATT | motif TGAATT in downstream of splicing site |
| D118 | AATTTA | motif AATTTA in downstream of splicing site |
| D119 | TATGT | motif TATGT in downstream of splicing site |
| D120 | GCATTT | motif GCATTT in downstream of splicing site |
| D121 | AAGTA | motif AAGTA in downstream of splicing site |
| D122 | GCTTCT | motif GCTTCT in downstream of splicing site |
| D123 | TTCTAA | motif TTCTAA in downstream of splicing site |
| D124 | GTTTCT | motif GTTTCT in downstream of splicing site |
| D125 | AGATTT | motif AGATTT in downstream of splicing site |
| D126 | GAAAAT | motif GAAAAT in downstream of splicing site |
| D127 | TGCTAA | motif TGCTAA in downstream of splicing site |
| D128 | AAGCT | motif AAGCT in downstream of splicing site |
| D129 | CTTTGCT | motif CTTTGCT in downstream of splicing site |
| D130 | TCTGA | motif TCTGA in downstream of splicing site |
| D131 | TTTCTC | motif TTTCTC in downstream of splicing site |
| D132 | TTTATTC | motif TTTATTC in downstream of splicing site |
| D133 | TTTGCC | motif TTTGCC in downstream of splicing site |
| D134 | TGAAAG | motif TGAAAG in downstream of splicing site |
| D135 | TGTTCT | motif TGTTCT in downstream of splicing site |
| D136 | CTTTT | motif CTTTT in downstream of splicing site |
| D137 | TTTTCTG | motif TTTTCTG in downstream of splicing site |
| D138 | TGAGT | motif TGAGT in downstream of splicing site |
| D139 | TTGCAG | motif TTGCAG in downstream of splicing site |
| D140 | TAATA | motif TAATA in downstream of splicing site |
| D141 | AGTAT | motif AGTAT in downstream of splicing site |
| D142 | ATTCT | motif ATTCT in downstream of splicing site |
| D143 | TGCCTTT | motif TGCCTTT in downstream of splicing site |
| D144 | ATCAAA | motif ATCAAA in downstream of splicing site |
| D145 | GAGTG | motif GAGTG in downstream of splicing site |
| D146 | TAGGT | motif TAGGT in downstream of splicing site |
| D147 | CTTTA | motif CTTTA in downstream of splicing site |
| D148 | TTTAG | motif TTTAG in downstream of splicing site |
| D149 | TGATTTT | motif TGATTTT in downstream of splicing site |
| D150 | TTTCAT | motif TTTCAT in downstream of splicing site |
| D151 | CTTTCA | motif CTTTCA in downstream of splicing site |
| D152 | AAGAT | motif AAGAT in downstream of splicing site |
| D153 | TGCTT | motif TGCTT in downstream of splicing site |
| D154 | GTGGGT | motif GTGGGT in downstream of splicing site |
| D155 | GTAAAG | motif GTAAAG in downstream of splicing site |
| D156 | CTGAA | motif CTGAA in downstream of splicing site |
| D157 | TCTGC | motif TCTGC in downstream of splicing site |
| D158 | CTAAA | motif CTAAA in downstream of splicing site |
| U1 | GTTTGT | motif GTTTGT in upstream of splicing site |
| U2 | TCTCC | motif TCTCC in upstream of splicing site |
| U3 | GATTTT | motif GATTTT in upstream of splicing site |
| U4 | TTTTTC | motif TTTTTC in upstream of splicing site |
| U5 | TAACC | motif TAACC in upstream of splicing site |
| U6 | TTGAAAT | motif TTGAAAT in upstream of splicing site |
| U7 | AAGCCA | motif AAGCCA in upstream of splicing site |
| U8 | AATTG | motif AATTG in upstream of splicing site |
| U9 | CTGCT | motif CTGCT in upstream of splicing site |
| U10 | TTTATG | motif TTTATG in upstream of splicing site |
| U11 | TTCACA | motif TTCACA in upstream of splicing site |
| U12 | TGATAA | motif TGATAA in upstream of splicing site |
| U13 | ATGTTT | motif ATGTTT in upstream of splicing site |
| U14 | TCCAG | motif TCCAG in upstream of splicing site |
| U15 | TTTCCAA | motif TTTCCAA in upstream of splicing site |
| U16 | TTATTTC | motif TTATTTC in upstream of splicing site |
| U17 | TGTGTT | motif TGTGTT in upstream of splicing site |
| U18 | TCTTG | motif TCTTG in upstream of splicing site |
| U19 | TTGTAA | motif TTGTAA in upstream of splicing site |
| U20 | CTTGAC | motif CTTGAC in upstream of splicing site |
| U21 | TTAAAAC | motif TTAAAAC in upstream of splicing site |
| U22 | CTAAC | motif CTAAC in upstream of splicing site |
| U23 | AAAGCT | motif AAAGCT in upstream of splicing site |
| U24 | TCTTC | motif TCTTC in upstream of splicing site |
| U25 | TGCATG | motif TGCATG in upstream of splicing site |
| U26 | TTCTT | motif TTCTT in upstream of splicing site |
| U27 | ACATTT | motif ACATTT in upstream of splicing site |
| U28 | TTTATC | motif TTTATC in upstream of splicing site |
| U29 | ATTTTCT | motif ATTTTCT in upstream of splicing site |
| U30 | GCTGACC | motif GCTGACC in upstream of splicing site |
| U31 | ATAATT | motif ATAATT in upstream of splicing site |
| U32 | AACAG | motif AACAG in upstream of splicing site |
| U33 | CATTTG | motif CATTTG in upstream of splicing site |
| U34 | TTTCAG | motif TTTCAG in upstream of splicing site |
| U35 | TTAGCA | motif TTAGCA in upstream of splicing site |
| U36 | TTGCCT | motif TTGCCT in upstream of splicing site |
| U37 | CAAAT | motif CAAAT in upstream of splicing site |
| U38 | TTTAAC | motif TTTAAC in upstream of splicing site |
| U39 | TAATG | motif TAATG in upstream of splicing site |
| U40 | AGAAAT | motif AGAAAT in upstream of splicing site |
| U41 | TTTTGAT | motif TTTTGAT in upstream of splicing site |
| U42 | TAACT | motif TAACT in upstream of splicing site |
| U43 | TAAAAT | motif TAAAAT in upstream of splicing site |
| U44 | AATTACA | motif AATTACA in upstream of splicing site |
| U45 | TTCAAAA | motif TTCAAAA in upstream of splicing site |
| U46 | TTTATA | motif TTTATA in upstream of splicing site |
| U47 | CTTGTC | motif CTTGTC in upstream of splicing site |
| U48 | TTTACAG | motif TTTACAG in upstream of splicing site |
| U49 | TGGATTT | motif TGGATTT in upstream of splicing site |
| U50 | TTGCATT | motif TTGCATT in upstream of splicing site |
| U51 | TTAAG | motif TTAAG in upstream of splicing site |
| U52 | TTGGT | motif TTGGT in upstream of splicing site |
| U53 | CATAAA | motif CATAAA in upstream of splicing site |
| U54 | TGATTA | motif TGATTA in upstream of splicing site |
| U55 | GCTTTGC | motif GCTTTGC in upstream of splicing site |
| U56 | ATTAG | motif ATTAG in upstream of splicing site |
| U57 | TTTTAAA | motif TTTTAAA in upstream of splicing site |
| U58 | ACTAAT | motif ACTAAT in upstream of splicing site |
| U59 | CTGACT | motif CTGACT in upstream of splicing site |
| U60 | ATATTT | motif ATATTT in upstream of splicing site |
| U61 | CATTTA | motif CATTTA in upstream of splicing site |
| U62 | AAATCT | motif AAATCT in upstream of splicing site |
| U63 | TTTTGGC | motif TTTTGGC in upstream of splicing site |
| U64 | TCTTT | motif TCTTT in upstream of splicing site |
| U65 | TTATTGA | motif TTATTGA in upstream of splicing site |
| U66 | TCAGA | motif TCAGA in upstream of splicing site |
| U67 | TAAGT | motif TAAGT in upstream of splicing site |
| U68 | CTCTG | motif CTCTG in upstream of splicing site |
| U69 | TCTGG | motif TCTGG in upstream of splicing site |
| U70 | AATTC | motif AATTC in upstream of splicing site |
| U71 | TTTTCC | motif TTTTCC in upstream of splicing site |
| U72 | TTTTGC | motif TTTTGC in upstream of splicing site |
| U73 | GTGAG | motif GTGAG in upstream of splicing site |
| U74 | TCCATTT | motif TCCATTT in upstream of splicing site |
| U75 | AATTTT | motif AATTTT in upstream of splicing site |
| U76 | CTTGATT | motif CTTGATT in upstream of splicing site |
| U77 | GTAAG | motif GTAAG in upstream of splicing site |
| U78 | ATGAAA | motif ATGAAA in upstream of splicing site |
| U79 | AGAAAA | motif AGAAAA in upstream of splicing site |
| U80 | TGGCTT | motif TGGCTT in upstream of splicing site |
| U81 | CTCAG | motif CTCAG in upstream of splicing site |
| U82 | TGGAAAT | motif TGGAAAT in upstream of splicing site |
| U83 | AATTAT | motif AATTAT in upstream of splicing site |
| U84 | AATAAT | motif AATAAT in upstream of splicing site |
| U85 | TCCTAG | motif TCCTAG in upstream of splicing site |
| U86 | CCACAG | motif CCACAG in upstream of splicing site |
| U87 | TCATTTC | motif TCATTTC in upstream of splicing site |
| U88 | AAAGCA | motif AAAGCA in upstream of splicing site |
| U89 | AAATGA | motif AAATGA in upstream of splicing site |
| U90 | TTTATAG | motif TTTATAG in upstream of splicing site |
| U91 | ATTAAAT | motif ATTAAAT in upstream of splicing site |
| U92 | CCTGCAG | motif CCTGCAG in upstream of splicing site |
| U93 | TTACAG | motif TTACAG in upstream of splicing site |
| U94 | AAATGT | motif AAATGT in upstream of splicing site |
| U95 | TGCAT | motif TGCAT in upstream of splicing site |
| U96 | CTTCT | motif CTTCT in upstream of splicing site |
| U97 | TTAGAA | motif TTAGAA in upstream of splicing site |
| U98 | TGTTTC | motif TGTTTC in upstream of splicing site |
| U99 | TTTAC | motif TTTAC in upstream of splicing site |
| U100 | GTTTT | motif GTTTT in upstream of splicing site |
| U101 | CTTCCA | motif CTTCCA in upstream of splicing site |
| U102 | TTCTAG | motif TTCTAG in upstream of splicing site |
| U103 | AAATT | motif AAATT in upstream of splicing site |
| U104 | TTAAAC | motif TTAAAC in upstream of splicing site |
| U105 | TGAGAA | motif TGAGAA in upstream of splicing site |
| U106 | TTTGTAG | motif TTTGTAG in upstream of splicing site |
| U107 | GTCAGT | motif GTCAGT in upstream of splicing site |
| U108 | TAAGA | motif TAAGA in upstream of splicing site |
| U109 | AAATCA | motif AAATCA in upstream of splicing site |
| U110 | TGTTGA | motif TGTTGA in upstream of splicing site |
| U111 | CTTGC | motif CTTGC in upstream of splicing site |
| U112 | TAATTTG | motif TAATTTG in upstream of splicing site |
| U113 | CCTCT | motif CCTCT in upstream of splicing site |
| U114 | TGGTTT | motif TGGTTT in upstream of splicing site |
| U115 | TGATTTC | motif TGATTTC in upstream of splicing site |
| U116 | TGTTAA | motif TGTTAA in upstream of splicing site |
| U117 | TGTGTC | motif TGTGTC in upstream of splicing site |
| U118 | TCTCT | motif TCTCT in upstream of splicing site |
| U119 | TTAACA | motif TTAACA in upstream of splicing site |
| U120 | TTTGGT | motif TTTGGT in upstream of splicing site |
| U121 | TGTCT | motif TGTCT in upstream of splicing site |
| U122 | TTCCTT | motif TTCCTT in upstream of splicing site |
| U123 | TGAATT | motif TGAATT in upstream of splicing site |
| U124 | AATTTA | motif AATTTA in upstream of splicing site |
| U125 | GTTTCT | motif GTTTCT in upstream of splicing site |
| U126 | TGCTAA | motif TGCTAA in upstream of splicing site |
| U127 | AGATTT | motif AGATTT in upstream of splicing site |
| U128 | GAAAAT | motif GAAAAT in upstream of splicing site |
| U129 | GTTTAAT | motif GTTTAAT in upstream of splicing site |
| U130 | TTTGACT | motif TTTGACT in upstream of splicing site |
| U131 | TCTGA | motif TCTGA in upstream of splicing site |
| U132 | TCTGTT | motif TCTGTT in upstream of splicing site |
| U133 | TTTATTC | motif TTTATTC in upstream of splicing site |
| U134 | TGAAAG | motif TGAAAG in upstream of splicing site |
| U135 | TGTTCT | motif TGTTCT in upstream of splicing site |
| U136 | CTTTT | motif CTTTT in upstream of splicing site |
| U137 | ATTTGT | motif ATTTGT in upstream of splicing site |
| U138 | TGAGT | motif TGAGT in upstream of splicing site |
| U139 | CCCCAG | motif CCCCAG in upstream of splicing site |
| U140 | TTGCAG | motif TTGCAG in upstream of splicing site |
| U141 | CTGAT | motif CTGAT in upstream of splicing site |
| U142 | TAATA | motif TAATA in upstream of splicing site |
| U143 | TCTTA | motif TCTTA in upstream of splicing site |
| U144 | ATTCT | motif ATTCT in upstream of splicing site |
| U145 | ATCAAA | motif ATCAAA in upstream of splicing site |
| U146 | CTTTA | motif CTTTA in upstream of splicing site |
| U147 | TTTAG | motif TTTAG in upstream of splicing site |
| U148 | TTGCTG | motif TTGCTG in upstream of splicing site |
| U149 | TTTCAT | motif TTTCAT in upstream of splicing site |
| U150 | CTTTCA | motif CTTTCA in upstream of splicing site |
| U151 | TTCTC | motif TTCTC in upstream of splicing site |
| U152 | TGCTT | motif TGCTT in upstream of splicing site |
| U153 | TCTGC | motif TCTGC in upstream of splicing site |
| U154 | CTGAA | motif CTGAA in upstream of splicing site |
| U155 | GTAGGT | motif GTAGGT in upstream of splicing site |
| U156 | CTAAA | motif CTAAA in upstream of splicing site |

Table S4 Features used by mkcDBGAS

| No. | Features | Description |
| --- | --- | --- |
| 1 | length_of_as | Length of AS region |
| 2 | length_of_all_sequence | Length of upstream region + AS region + downstream region |
| 3 | DmotifGTAAC | Is there this motif in downstream of splicing site: GTAAC (1 for yes, 0 for no) |
| 4 | DmotifAAGTGT | Is there this motif in downstream of splicing site: AAGTGT (1 for yes, 0 for no) |
| 5 | DmotifGTTTGT | Is there this motif in downstream of splicing site: GTTTGT (1 for yes, 0 for no) |
| 6 | DmotifATTAACA | Is there this motif in downstream of splicing site: ATTAACA (1 for yes, 0 for no) |
| 7 | DmotifTGAAG | Is there this motif in downstream of splicing site: TGAAG (1 for yes, 0 for no) |
| 8 | DmotifTAACC | Is there this motif in downstream of splicing site: TAACC (1 for yes, 0 for no) |
| 9 | DmotifTTGAAAT | Is there this motif in downstream of splicing site: TTGAAAT (1 for yes, 0 for no) |
| 10 | DmotifAATTG | Is there this motif in downstream of splicing site: AATTG (1 for yes, 0 for no) |
| 11 | DmotifCTGCT | Is there this motif in downstream of splicing site: CTGCT (1 for yes, 0 for no) |
| 12 | DmotifTTTATG | Is there this motif in downstream of splicing site: TTTATG (1 for yes, 0 for no) |
| 13 | DmotifTGATAAA | Is there this motif in downstream of splicing site: TGATAAA (1 for yes, 0 for no) |
| 14 | DmotifATGTTT | Is there this motif in downstream of splicing site: ATGTTT (1 for yes, 0 for no) |
| 15 | DmotifTTTCCAA | Is there this motif in downstream of splicing site: TTTCCAA (1 for yes, 0 for no) |
| 16 | DmotifAAGTC | Is there this motif in downstream of splicing site: AAGTC (1 for yes, 0 for no) |
| 17 | DmotifAAAGA | Is there this motif in downstream of splicing site: AAAGA (1 for yes, 0 for no) |
| 18 | DmotifGTACGT | Is there this motif in downstream of splicing site: GTACGT (1 for yes, 0 for no) |
| 19 | DmotifGTTAAA | Is there this motif in downstream of splicing site: GTTAAA (1 for yes, 0 for no) |
| 20 | DmotifGAGCTG | Is there this motif in downstream of splicing site: GAGCTG (1 for yes, 0 for no) |
| 21 | DmotifTCATTTT | Is there this motif in downstream of splicing site: TCATTTT (1 for yes, 0 for no) |
| 22 | DmotifTGCATG | Is there this motif in downstream of splicing site: TGCATG (1 for yes, 0 for no) |
| 23 | DmotifTTCTT | Is there this motif in downstream of splicing site: TTCTT (1 for yes, 0 for no) |
| 24 | DmotifTTTATC | Is there this motif in downstream of splicing site: TTTATC (1 for yes, 0 for no) |
| 25 | DmotifACATTT | Is there this motif in downstream of splicing site: ACATTT (1 for yes, 0 for no) |
| 26 | DmotifTGCCAGC | Is there this motif in downstream of splicing site: TGCCAGC (1 for yes, 0 for no) |
| 27 | DmotifATAATT | Is there this motif in downstream of splicing site: ATAATT (1 for yes, 0 for no) |
| 28 | DmotifGTAGG | Is there this motif in downstream of splicing site: GTAGG (1 for yes, 0 for no) |
| 29 | DmotifGTATCCT | Is there this motif in downstream of splicing site: GTATCCT (1 for yes, 0 for no) |
| 30 | DmotifCATTTG | Is there this motif in downstream of splicing site: CATTTG (1 for yes, 0 for no) |
| 31 | DmotifACTAAC | Is there this motif in downstream of splicing site: ACTAAC (1 for yes, 0 for no) |
| 32 | DmotifTTTCAG | Is there this motif in downstream of splicing site: TTTCAG (1 for yes, 0 for no) |
| 33 | DmotifAATTGA | Is there this motif in downstream of splicing site: AATTGA (1 for yes, 0 for no) |
| 34 | DmotifTTAGCA | Is there this motif in downstream of splicing site: TTAGCA (1 for yes, 0 for no) |
| 35 | DmotifCAAAT | Is there this motif in downstream of splicing site: CAAAT (1 for yes, 0 for no) |
| 36 | DmotifTAATG | Is there this motif in downstream of splicing site: TAATG (1 for yes, 0 for no) |
| 37 | DmotifTTTTGAT | Is there this motif in downstream of splicing site: TTTTGAT (1 for yes, 0 for no) |
| 38 | DmotifAGAAAT | Is there this motif in downstream of splicing site: AGAAAT (1 for yes, 0 for no) |
| 39 | DmotifTTTCTA | Is there this motif in downstream of splicing site: TTTCTA (1 for yes, 0 for no) |
| 40 | DmotifTATTTC | Is there this motif in downstream of splicing site: TATTTC (1 for yes, 0 for no) |
| 41 | DmotifTAACT | Is there this motif in downstream of splicing site: TAACT (1 for yes, 0 for no) |
| 42 | DmotifTGAGG | Is there this motif in downstream of splicing site: TGAGG (1 for yes, 0 for no) |
| 43 | DmotifTAAAAT | Is there this motif in downstream of splicing site: TAAAAT (1 for yes, 0 for no) |
| 44 | DmotifTTTATA | Is there this motif in downstream of splicing site: TTTATA (1 for yes, 0 for no) |
| 45 | DmotifTATCCT | Is there this motif in downstream of splicing site: TATCCT (1 for yes, 0 for no) |
| 46 | DmotifGTTAGT | Is there this motif in downstream of splicing site: GTTAGT (1 for yes, 0 for no) |
| 47 | DmotifTTTACAG | Is there this motif in downstream of splicing site: TTTACAG (1 for yes, 0 for no) |
| 48 | DmotifTATTTG | Is there this motif in downstream of splicing site: TATTTG (1 for yes, 0 for no) |
| 49 | DmotifGTACTGT | Is there this motif in downstream of splicing site: GTACTGT (1 for yes, 0 for no) |
| 50 | DmotifTTAAG | Is there this motif in downstream of splicing site: TTAAG (1 for yes, 0 for no) |
| 51 | DmotifCATAAA | Is there this motif in downstream of splicing site: CATAAA (1 for yes, 0 for no) |
| 52 | DmotifGCATG | Is there this motif in downstream of splicing site: GCATG (1 for yes, 0 for no) |
| 53 | DmotifTGATTA | Is there this motif in downstream of splicing site: TGATTA (1 for yes, 0 for no) |
| 54 | DmotifTTTTAAA | Is there this motif in downstream of splicing site: TTTTAAA (1 for yes, 0 for no) |
| 55 | DmotifCTGACT | Is there this motif in downstream of splicing site: CTGACT (1 for yes, 0 for no) |
| 56 | DmotifACTAAT | Is there this motif in downstream of splicing site: ACTAAT (1 for yes, 0 for no) |
| 57 | DmotifGAGTA | Is there this motif in downstream of splicing site: GAGTA (1 for yes, 0 for no) |
| 58 | DmotifTCTTAA | Is there this motif in downstream of splicing site: TCTTAA (1 for yes, 0 for no) |
| 59 | DmotifTTGGTT | Is there this motif in downstream of splicing site: TTGGTT (1 for yes, 0 for no) |
| 60 | DmotifATATTT | Is there this motif in downstream of splicing site: ATATTT (1 for yes, 0 for no) |
| 61 | DmotifAGAGCCA | Is there this motif in downstream of splicing site: AGAGCCA (1 for yes, 0 for no) |
| 62 | DmotifTCTTT | Is there this motif in downstream of splicing site: TCTTT (1 for yes, 0 for no) |
| 63 | DmotifAGTTTT | Is there this motif in downstream of splicing site: AGTTTT (1 for yes, 0 for no) |
| 64 | DmotifGTATTT | Is there this motif in downstream of splicing site: GTATTT (1 for yes, 0 for no) |
| 65 | DmotifTCAGA | Is there this motif in downstream of splicing site: TCAGA (1 for yes, 0 for no) |
| 66 | DmotifTAAGT | Is there this motif in downstream of splicing site: TAAGT (1 for yes, 0 for no) |
| 67 | DmotifAAGCA | Is there this motif in downstream of splicing site: AAGCA (1 for yes, 0 for no) |
| 68 | DmotifTTCACAG | Is there this motif in downstream of splicing site: TTCACAG (1 for yes, 0 for no) |
| 69 | DmotifAGTAA | Is there this motif in downstream of splicing site: AGTAA (1 for yes, 0 for no) |
| 70 | DmotifTCTGG | Is there this motif in downstream of splicing site: TCTGG (1 for yes, 0 for no) |
| 71 | DmotifAGCTTT | Is there this motif in downstream of splicing site: AGCTTT (1 for yes, 0 for no) |
| 72 | DmotifTGATTTG | Is there this motif in downstream of splicing site: TGATTTG (1 for yes, 0 for no) |
| 73 | DmotifTTTTGC | Is there this motif in downstream of splicing site: TTTTGC (1 for yes, 0 for no) |
| 74 | DmotifTAGAAA | Is there this motif in downstream of splicing site: TAGAAA (1 for yes, 0 for no) |
| 75 | DmotifGTGAG | Is there this motif in downstream of splicing site: GTGAG (1 for yes, 0 for no) |
| 76 | DmotifTTCTGT | Is there this motif in downstream of splicing site: TTCTGT (1 for yes, 0 for no) |
| 77 | DmotifGTAAG | Is there this motif in downstream of splicing site: GTAAG (1 for yes, 0 for no) |
| 78 | DmotifATGAAA | Is there this motif in downstream of splicing site: ATGAAA (1 for yes, 0 for no) |
| 79 | DmotifAGAAAA | Is there this motif in downstream of splicing site: AGAAAA (1 for yes, 0 for no) |
| 80 | DmotifTGAGC | Is there this motif in downstream of splicing site: TGAGC (1 for yes, 0 for no) |
| 81 | DmotifTGGCTT | Is there this motif in downstream of splicing site: TGGCTT (1 for yes, 0 for no) |
| 82 | DmotifTTAATCT | Is there this motif in downstream of splicing site: TTAATCT (1 for yes, 0 for no) |
| 83 | DmotifAATTAT | Is there this motif in downstream of splicing site: AATTAT (1 for yes, 0 for no) |
| 84 | DmotifTGGAAAT | Is there this motif in downstream of splicing site: TGGAAAT (1 for yes, 0 for no) |
| 85 | DmotifCCACAG | Is there this motif in downstream of splicing site: CCACAG (1 for yes, 0 for no) |
| 86 | DmotifAAATGA | Is there this motif in downstream of splicing site: AAATGA (1 for yes, 0 for no) |
| 87 | DmotifGCAAGT | Is there this motif in downstream of splicing site: GCAAGT (1 for yes, 0 for no) |
| 88 | DmotifGTAAAA | Is there this motif in downstream of splicing site: GTAAAA (1 for yes, 0 for no) |
| 89 | DmotifGTCTG | Is there this motif in downstream of splicing site: GTCTG (1 for yes, 0 for no) |
| 90 | DmotifAAATGT | Is there this motif in downstream of splicing site: AAATGT (1 for yes, 0 for no) |
| 91 | DmotifTGCAT | Is there this motif in downstream of splicing site: TGCAT (1 for yes, 0 for no) |
| 92 | DmotifGAGAAA | Is there this motif in downstream of splicing site: GAGAAA (1 for yes, 0 for no) |
| 93 | DmotifTTAGA | Is there this motif in downstream of splicing site: TTAGA (1 for yes, 0 for no) |
| 94 | DmotifTTTATAA | Is there this motif in downstream of splicing site: TTTATAA (1 for yes, 0 for no) |
| 95 | DmotifGTTTT | Is there this motif in downstream of splicing site: GTTTT (1 for yes, 0 for no) |
| 96 | DmotifGCTTGGC | Is there this motif in downstream of splicing site: GCTTGGC (1 for yes, 0 for no) |
| 97 | DmotifTAAGC | Is there this motif in downstream of splicing site: TAAGC (1 for yes, 0 for no) |
| 98 | DmotifGTATG | Is there this motif in downstream of splicing site: GTATG (1 for yes, 0 for no) |
| 99 | DmotifAAATT | Is there this motif in downstream of splicing site: AAATT (1 for yes, 0 for no) |
| 100 | DmotifGTAAT | Is there this motif in downstream of splicing site: GTAAT (1 for yes, 0 for no) |
| 101 | DmotifTTCTCT | Is there this motif in downstream of splicing site: TTCTCT (1 for yes, 0 for no) |
| 102 | DmotifTGAGAA | Is there this motif in downstream of splicing site: TGAGAA (1 for yes, 0 for no) |
| 103 | DmotifTTAGTT | Is there this motif in downstream of splicing site: TTAGTT (1 for yes, 0 for no) |
| 104 | DmotifTAAGG | Is there this motif in downstream of splicing site: TAAGG (1 for yes, 0 for no) |
| 105 | DmotifTGTTTAA | Is there this motif in downstream of splicing site: TGTTTAA (1 for yes, 0 for no) |
| 106 | DmotifGTCAGT | Is there this motif in downstream of splicing site: GTCAGT (1 for yes, 0 for no) |
| 107 | DmotifAGAATT | Is there this motif in downstream of splicing site: AGAATT (1 for yes, 0 for no) |
| 108 | DmotifTAAATG | Is there this motif in downstream of splicing site: TAAATG (1 for yes, 0 for no) |
| 109 | DmotifAATTCA | Is there this motif in downstream of splicing site: AATTCA (1 for yes, 0 for no) |
| 110 | DmotifTCCTTT | Is there this motif in downstream of splicing site: TCCTTT (1 for yes, 0 for no) |
| 111 | DmotifTAAGA | Is there this motif in downstream of splicing site: TAAGA (1 for yes, 0 for no) |
| 112 | DmotifAAATCA | Is there this motif in downstream of splicing site: AAATCA (1 for yes, 0 for no) |
| 113 | DmotifTAATTTG | Is there this motif in downstream of splicing site: TAATTTG (1 for yes, 0 for no) |
| 114 | DmotifGAAATA | Is there this motif in downstream of splicing site: GAAATA (1 for yes, 0 for no) |
| 115 | DmotifTGGTTT | Is there this motif in downstream of splicing site: TGGTTT (1 for yes, 0 for no) |
| 116 | DmotifTGTTAA | Is there this motif in downstream of splicing site: TGTTAA (1 for yes, 0 for no) |
| 117 | DmotifTGTCT | Is there this motif in downstream of splicing site: TGTCT (1 for yes, 0 for no) |
| 118 | DmotifGTTGGT | Is there this motif in downstream of splicing site: GTTGGT (1 for yes, 0 for no) |
| 119 | DmotifTGAATT | Is there this motif in downstream of splicing site: TGAATT (1 for yes, 0 for no) |
| 120 | DmotifAATTTA | Is there this motif in downstream of splicing site: AATTTA (1 for yes, 0 for no) |
| 121 | DmotifTATGT | Is there this motif in downstream of splicing site: TATGT (1 for yes, 0 for no) |
| 122 | DmotifGCATTT | Is there this motif in downstream of splicing site: GCATTT (1 for yes, 0 for no) |
| 123 | DmotifAAGTA | Is there this motif in downstream of splicing site: AAGTA (1 for yes, 0 for no) |
| 124 | DmotifGCTTCT | Is there this motif in downstream of splicing site: GCTTCT (1 for yes, 0 for no) |
| 125 | DmotifTTCTAA | Is there this motif in downstream of splicing site: TTCTAA (1 for yes, 0 for no) |
| 126 | DmotifGTTTCT | Is there this motif in downstream of splicing site: GTTTCT (1 for yes, 0 for no) |
| 127 | DmotifAGATTT | Is there this motif in downstream of splicing site: AGATTT (1 for yes, 0 for no) |
| 128 | DmotifGAAAAT | Is there this motif in downstream of splicing site: GAAAAT (1 for yes, 0 for no) |
| 129 | DmotifTGCTAA | Is there this motif in downstream of splicing site: TGCTAA (1 for yes, 0 for no) |
| 130 | DmotifAAGCT | Is there this motif in downstream of splicing site: AAGCT (1 for yes, 0 for no) |
| 131 | DmotifCTTTGCT | Is there this motif in downstream of splicing site: CTTTGCT (1 for yes, 0 for no) |
| 132 | DmotifTCTGA | Is there this motif in downstream of splicing site: TCTGA (1 for yes, 0 for no) |
| 133 | DmotifTTTCTC | Is there this motif in downstream of splicing site: TTTCTC (1 for yes, 0 for no) |
| 134 | DmotifTTTATTC | Is there this motif in downstream of splicing site: TTTATTC (1 for yes, 0 for no) |
| 135 | DmotifTTTGCC | Is there this motif in downstream of splicing site: TTTGCC (1 for yes, 0 for no) |
| 136 | DmotifTGAAAG | Is there this motif in downstream of splicing site: TGAAAG (1 for yes, 0 for no) |
| 137 | DmotifTGTTCT | Is there this motif in downstream of splicing site: TGTTCT (1 for yes, 0 for no) |
| 138 | DmotifCTTTT | Is there this motif in downstream of splicing site: CTTTT (1 for yes, 0 for no) |
| 139 | DmotifTTTTCTG | Is there this motif in downstream of splicing site: TTTTCTG (1 for yes, 0 for no) |
| 140 | DmotifTGAGT | Is there this motif in downstream of splicing site: TGAGT (1 for yes, 0 for no) |
| 141 | DmotifTTGCAG | Is there this motif in downstream of splicing site: TTGCAG (1 for yes, 0 for no) |
| 142 | DmotifTAATA | Is there this motif in downstream of splicing site: TAATA (1 for yes, 0 for no) |
| 143 | DmotifAGTAT | Is there this motif in downstream of splicing site: AGTAT (1 for yes, 0 for no) |
| 144 | DmotifATTCT | Is there this motif in downstream of splicing site: ATTCT (1 for yes, 0 for no) |
| 145 | DmotifTGCCTTT | Is there this motif in downstream of splicing site: TGCCTTT (1 for yes, 0 for no) |
| 146 | DmotifATCAAA | Is there this motif in downstream of splicing site: ATCAAA (1 for yes, 0 for no) |
| 147 | DmotifGAGTG | Is there this motif in downstream of splicing site: GAGTG (1 for yes, 0 for no) |
| 148 | DmotifTAGGT | Is there this motif in downstream of splicing site: TAGGT (1 for yes, 0 for no) |
| 149 | DmotifCTTTA | Is there this motif in downstream of splicing site: CTTTA (1 for yes, 0 for no) |
| 150 | DmotifTTTAG | Is there this motif in downstream of splicing site: TTTAG (1 for yes, 0 for no) |
| 151 | DmotifTGATTTT | Is there this motif in downstream of splicing site: TGATTTT (1 for yes, 0 for no) |
| 152 | DmotifTTTCAT | Is there this motif in downstream of splicing site: TTTCAT (1 for yes, 0 for no) |
| 153 | DmotifCTTTCA | Is there this motif in downstream of splicing site: CTTTCA (1 for yes, 0 for no) |
| 154 | DmotifAAGAT | Is there this motif in downstream of splicing site: AAGAT (1 for yes, 0 for no) |
| 155 | DmotifTGCTT | Is there this motif in downstream of splicing site: TGCTT (1 for yes, 0 for no) |
| 156 | DmotifGTGGGT | Is there this motif in downstream of splicing site: GTGGGT (1 for yes, 0 for no) |
| 157 | DmotifGTAAAG | Is there this motif in downstream of splicing site: GTAAAG (1 for yes, 0 for no) |
| 158 | DmotifCTGAA | Is there this motif in downstream of splicing site: CTGAA (1 for yes, 0 for no) |
| 159 | DmotifTCTGC | Is there this motif in downstream of splicing site: TCTGC (1 for yes, 0 for no) |
| 160 | DmotifCTAAA | Is there this motif in downstream of splicing site: CTAAA (1 for yes, 0 for no) |
| 161 | UmotifGTTTGT | Is there this motif in upstream of splicing site: GTTTGT (1 for yes, 0 for no) |
| 162 | UmotifTCTCC | Is there this motif in upstream of splicing site: TCTCC (1 for yes, 0 for no) |
| 163 | UmotifGATTTT | Is there this motif in upstream of splicing site: GATTTT (1 for yes, 0 for no) |
| 164 | UmotifTTTTTC | Is there this motif in upstream of splicing site: TTTTTC (1 for yes, 0 for no) |
| 165 | UmotifTAACC | Is there this motif in upstream of splicing site: TAACC (1 for yes, 0 for no) |
| 166 | UmotifTTGAAAT | Is there this motif in upstream of splicing site: TTGAAAT (1 for yes, 0 for no) |
| 167 | UmotifAAGCCA | Is there this motif in upstream of splicing site: AAGCCA (1 for yes, 0 for no) |
| 168 | UmotifAATTG | Is there this motif in upstream of splicing site: AATTG (1 for yes, 0 for no) |
| 169 | UmotifCTGCT | Is there this motif in upstream of splicing site: CTGCT (1 for yes, 0 for no) |
| 170 | UmotifTTTATG | Is there this motif in upstream of splicing site: TTTATG (1 for yes, 0 for no) |
| 171 | UmotifTTCACA | Is there this motif in upstream of splicing site: TTCACA (1 for yes, 0 for no) |
| 172 | UmotifTGATAA | Is there this motif in upstream of splicing site: TGATAA (1 for yes, 0 for no) |
| 173 | UmotifATGTTT | Is there this motif in upstream of splicing site: ATGTTT (1 for yes, 0 for no) |
| 174 | UmotifTCCAG | Is there this motif in upstream of splicing site: TCCAG (1 for yes, 0 for no) |
| 175 | UmotifTTTCCAA | Is there this motif in upstream of splicing site: TTTCCAA (1 for yes, 0 for no) |
| 176 | UmotifTTATTTC | Is there this motif in upstream of splicing site: TTATTTC (1 for yes, 0 for no) |
| 177 | UmotifTGTGTT | Is there this motif in upstream of splicing site: TGTGTT (1 for yes, 0 for no) |
| 178 | UmotifTCTTG | Is there this motif in upstream of splicing site: TCTTG (1 for yes, 0 for no) |
| 179 | UmotifTTGTAA | Is there this motif in upstream of splicing site: TTGTAA (1 for yes, 0 for no) |
| 180 | UmotifCTTGAC | Is there this motif in upstream of splicing site: CTTGAC (1 for yes, 0 for no) |
| 181 | UmotifTTAAAAC | Is there this motif in upstream of splicing site: TTAAAAC (1 for yes, 0 for no) |
| 182 | UmotifCTAAC | Is there this motif in upstream of splicing site: CTAAC (1 for yes, 0 for no) |
| 183 | UmotifAAAGCT | Is there this motif in upstream of splicing site: AAAGCT (1 for yes, 0 for no) |
| 184 | UmotifTCTTC | Is there this motif in upstream of splicing site: TCTTC (1 for yes, 0 for no) |
| 185 | UmotifTGCATG | Is there this motif in upstream of splicing site: TGCATG (1 for yes, 0 for no) |
| 186 | UmotifTTCTT | Is there this motif in upstream of splicing site: TTCTT (1 for yes, 0 for no) |
| 187 | UmotifACATTT | Is there this motif in upstream of splicing site: ACATTT (1 for yes, 0 for no) |
| 188 | UmotifTTTATC | Is there this motif in upstream of splicing site: TTTATC (1 for yes, 0 for no) |
| 189 | UmotifATTTTCT | Is there this motif in upstream of splicing site: ATTTTCT (1 for yes, 0 for no) |
| 190 | UmotifGCTGACC | Is there this motif in upstream of splicing site: GCTGACC (1 for yes, 0 for no) |
| 191 | UmotifATAATT | Is there this motif in upstream of splicing site: ATAATT (1 for yes, 0 for no) |
| 192 | UmotifAACAG | Is there this motif in upstream of splicing site: AACAG (1 for yes, 0 for no) |
| 193 | UmotifCATTTG | Is there this motif in upstream of splicing site: CATTTG (1 for yes, 0 for no) |
| 194 | UmotifTTTCAG | Is there this motif in upstream of splicing site: TTTCAG (1 for yes, 0 for no) |
| 195 | UmotifTTAGCA | Is there this motif in upstream of splicing site: TTAGCA (1 for yes, 0 for no) |
| 196 | UmotifTTGCCT | Is there this motif in upstream of splicing site: TTGCCT (1 for yes, 0 for no) |
| 197 | UmotifCAAAT | Is there this motif in upstream of splicing site: CAAAT (1 for yes, 0 for no) |
| 198 | UmotifTTTAAC | Is there this motif in upstream of splicing site: TTTAAC (1 for yes, 0 for no) |
| 199 | UmotifTAATG | Is there this motif in upstream of splicing site: TAATG (1 for yes, 0 for no) |
| 200 | UmotifAGAAAT | Is there this motif in upstream of splicing site: AGAAAT (1 for yes, 0 for no) |
| 201 | UmotifTTTTGAT | Is there this motif in upstream of splicing site: TTTTGAT (1 for yes, 0 for no) |
| 202 | UmotifTAACT | Is there this motif in upstream of splicing site: TAACT (1 for yes, 0 for no) |
| 203 | UmotifTAAAAT | Is there this motif in upstream of splicing site: TAAAAT (1 for yes, 0 for no) |
| 204 | UmotifAATTACA | Is there this motif in upstream of splicing site: AATTACA (1 for yes, 0 for no) |
| 205 | UmotifTTCAAAA | Is there this motif in upstream of splicing site: TTCAAAA (1 for yes, 0 for no) |
| 206 | UmotifTTTATA | Is there this motif in upstream of splicing site: TTTATA (1 for yes, 0 for no) |
| 207 | UmotifCTTGTC | Is there this motif in upstream of splicing site: CTTGTC (1 for yes, 0 for no) |
| 208 | UmotifTTTACAG | Is there this motif in upstream of splicing site: TTTACAG (1 for yes, 0 for no) |
| 209 | UmotifTGGATTT | Is there this motif in upstream of splicing site: TGGATTT (1 for yes, 0 for no) |
| 210 | UmotifTTGCATT | Is there this motif in upstream of splicing site: TTGCATT (1 for yes, 0 for no) |
| 211 | UmotifTTAAG | Is there this motif in upstream of splicing site: TTAAG (1 for yes, 0 for no) |
| 212 | UmotifTTGGT | Is there this motif in upstream of splicing site: TTGGT (1 for yes, 0 for no) |
| 213 | UmotifCATAAA | Is there this motif in upstream of splicing site: CATAAA (1 for yes, 0 for no) |
| 214 | UmotifTGATTA | Is there this motif in upstream of splicing site: TGATTA (1 for yes, 0 for no) |
| 215 | UmotifGCTTTGC | Is there this motif in upstream of splicing site: GCTTTGC (1 for yes, 0 for no) |
| 216 | UmotifATTAG | Is there this motif in upstream of splicing site: ATTAG (1 for yes, 0 for no) |
| 217 | UmotifTTTTAAA | Is there this motif in upstream of splicing site: TTTTAAA (1 for yes, 0 for no) |
| 218 | UmotifACTAAT | Is there this motif in upstream of splicing site: ACTAAT (1 for yes, 0 for no) |
| 219 | UmotifCTGACT | Is there this motif in upstream of splicing site: CTGACT (1 for yes, 0 for no) |
| 220 | UmotifATATTT | Is there this motif in upstream of splicing site: ATATTT (1 for yes, 0 for no) |
| 221 | UmotifCATTTA | Is there this motif in upstream of splicing site: CATTTA (1 for yes, 0 for no) |
| 222 | UmotifAAATCT | Is there this motif in upstream of splicing site: AAATCT (1 for yes, 0 for no) |
| 223 | UmotifTTTTGGC | Is there this motif in upstream of splicing site: TTTTGGC (1 for yes, 0 for no) |
| 224 | UmotifTCTTT | Is there this motif in upstream of splicing site: TCTTT (1 for yes, 0 for no) |
| 225 | UmotifTTATTGA | Is there this motif in upstream of splicing site: TTATTGA (1 for yes, 0 for no) |
| 226 | UmotifTCAGA | Is there this motif in upstream of splicing site: TCAGA (1 for yes, 0 for no) |
| 227 | UmotifTAAGT | Is there this motif in upstream of splicing site: TAAGT (1 for yes, 0 for no) |
| 228 | UmotifCTCTG | Is there this motif in upstream of splicing site: CTCTG (1 for yes, 0 for no) |
| 229 | UmotifTCTGG | Is there this motif in upstream of splicing site: TCTGG (1 for yes, 0 for no) |
| 230 | UmotifAATTC | Is there this motif in upstream of splicing site: AATTC (1 for yes, 0 for no) |
| 231 | UmotifTTTTCC | Is there this motif in upstream of splicing site: TTTTCC (1 for yes, 0 for no) |
| 232 | UmotifTTTTGC | Is there this motif in upstream of splicing site: TTTTGC (1 for yes, 0 for no) |
| 233 | UmotifGTGAG | Is there this motif in upstream of splicing site: GTGAG (1 for yes, 0 for no) |
| 234 | UmotifTCCATTT | Is there this motif in upstream of splicing site: TCCATTT (1 for yes, 0 for no) |
| 235 | UmotifAATTTT | Is there this motif in upstream of splicing site: AATTTT (1 for yes, 0 for no) |
| 236 | UmotifCTTGATT | Is there this motif in upstream of splicing site: CTTGATT (1 for yes, 0 for no) |
| 237 | UmotifGTAAG | Is there this motif in upstream of splicing site: GTAAG (1 for yes, 0 for no) |
| 238 | UmotifATGAAA | Is there this motif in upstream of splicing site: ATGAAA (1 for yes, 0 for no) |
| 239 | UmotifAGAAAA | Is there this motif in upstream of splicing site: AGAAAA (1 for yes, 0 for no) |
| 240 | UmotifTGGCTT | Is there this motif in upstream of splicing site: TGGCTT (1 for yes, 0 for no) |
| 241 | UmotifCTCAG | Is there this motif in upstream of splicing site: CTCAG (1 for yes, 0 for no) |
| 242 | UmotifTGGAAAT | Is there this motif in upstream of splicing site: TGGAAAT (1 for yes, 0 for no) |
| 243 | UmotifAATTAT | Is there this motif in upstream of splicing site: AATTAT (1 for yes, 0 for no) |
| 244 | UmotifAATAAT | Is there this motif in upstream of splicing site: AATAAT (1 for yes, 0 for no) |
| 245 | UmotifTCCTAG | Is there this motif in upstream of splicing site: TCCTAG (1 for yes, 0 for no) |
| 246 | UmotifCCACAG | Is there this motif in upstream of splicing site: CCACAG (1 for yes, 0 for no) |
| 247 | UmotifTCATTTC | Is there this motif in upstream of splicing site: TCATTTC (1 for yes, 0 for no) |
| 248 | UmotifAAAGCA | Is there this motif in upstream of splicing site: AAAGCA (1 for yes, 0 for no) |
| 249 | UmotifAAATGA | Is there this motif in upstream of splicing site: AAATGA (1 for yes, 0 for no) |
| 250 | UmotifTTTATAG | Is there this motif in upstream of splicing site: TTTATAG (1 for yes, 0 for no) |
| 251 | UmotifATTAAAT | Is there this motif in upstream of splicing site: ATTAAAT (1 for yes, 0 for no) |
| 252 | UmotifCCTGCAG | Is there this motif in upstream of splicing site: CCTGCAG (1 for yes, 0 for no) |
| 253 | UmotifTTACAG | Is there this motif in upstream of splicing site: TTACAG (1 for yes, 0 for no) |
| 254 | UmotifAAATGT | Is there this motif in upstream of splicing site: AAATGT (1 for yes, 0 for no) |
| 255 | UmotifTGCAT | Is there this motif in upstream of splicing site: TGCAT (1 for yes, 0 for no) |
| 256 | UmotifCTTCT | Is there this motif in upstream of splicing site: CTTCT (1 for yes, 0 for no) |
| 257 | UmotifTTAGAA | Is there this motif in upstream of splicing site: TTAGAA (1 for yes, 0 for no) |
| 258 | UmotifTGTTTC | Is there this motif in upstream of splicing site: TGTTTC (1 for yes, 0 for no) |
| 259 | UmotifTTTAC | Is there this motif in upstream of splicing site: TTTAC (1 for yes, 0 for no) |
| 260 | UmotifGTTTT | Is there this motif in upstream of splicing site: GTTTT (1 for yes, 0 for no) |
| 261 | UmotifCTTCCA | Is there this motif in upstream of splicing site: CTTCCA (1 for yes, 0 for no) |
| 262 | UmotifTTCTAG | Is there this motif in upstream of splicing site: TTCTAG (1 for yes, 0 for no) |
| 263 | UmotifAAATT | Is there this motif in upstream of splicing site: AAATT (1 for yes, 0 for no) |
| 264 | UmotifTTAAAC | Is there this motif in upstream of splicing site: TTAAAC (1 for yes, 0 for no) |
| 265 | UmotifTGAGAA | Is there this motif in upstream of splicing site: TGAGAA (1 for yes, 0 for no) |
| 266 | UmotifTTTGTAG | Is there this motif in upstream of splicing site: TTTGTAG (1 for yes, 0 for no) |
| 267 | UmotifGTCAGT | Is there this motif in upstream of splicing site: GTCAGT (1 for yes, 0 for no) |
| 268 | UmotifTAAGA | Is there this motif in upstream of splicing site: TAAGA (1 for yes, 0 for no) |
| 269 | UmotifAAATCA | Is there this motif in upstream of splicing site: AAATCA (1 for yes, 0 for no) |
| 270 | UmotifTGTTGA | Is there this motif in upstream of splicing site: TGTTGA (1 for yes, 0 for no) |
| 271 | UmotifCTTGC | Is there this motif in upstream of splicing site: CTTGC (1 for yes, 0 for no) |
| 272 | UmotifTAATTTG | Is there this motif in upstream of splicing site: TAATTTG (1 for yes, 0 for no) |
| 273 | UmotifCCTCT | Is there this motif in upstream of splicing site: CCTCT (1 for yes, 0 for no) |
| 274 | UmotifTGGTTT | Is there this motif in upstream of splicing site: TGGTTT (1 for yes, 0 for no) |
| 275 | UmotifTGATTTC | Is there this motif in upstream of splicing site: TGATTTC (1 for yes, 0 for no) |
| 276 | UmotifTGTTAA | Is there this motif in upstream of splicing site: TGTTAA (1 for yes, 0 for no) |
| 277 | UmotifTGTGTC | Is there this motif in upstream of splicing site: TGTGTC (1 for yes, 0 for no) |
| 278 | UmotifTCTCT | Is there this motif in upstream of splicing site: TCTCT (1 for yes, 0 for no) |
| 279 | UmotifTTAACA | Is there this motif in upstream of splicing site: TTAACA (1 for yes, 0 for no) |
| 280 | UmotifTTTGGT | Is there this motif in upstream of splicing site: TTTGGT (1 for yes, 0 for no) |
| 281 | UmotifTGTCT | Is there this motif in upstream of splicing site: TGTCT (1 for yes, 0 for no) |
| 282 | UmotifTTCCTT | Is there this motif in upstream of splicing site: TTCCTT (1 for yes, 0 for no) |
| 283 | UmotifTGAATT | Is there this motif in upstream of splicing site: TGAATT (1 for yes, 0 for no) |
| 284 | UmotifAATTTA | Is there this motif in upstream of splicing site: AATTTA (1 for yes, 0 for no) |
| 285 | UmotifGTTTCT | Is there this motif in upstream of splicing site: GTTTCT (1 for yes, 0 for no) |
| 286 | UmotifTGCTAA | Is there this motif in upstream of splicing site: TGCTAA (1 for yes, 0 for no) |
| 287 | UmotifAGATTT | Is there this motif in upstream of splicing site: AGATTT (1 for yes, 0 for no) |
| 288 | UmotifGAAAAT | Is there this motif in upstream of splicing site: GAAAAT (1 for yes, 0 for no) |
| 289 | UmotifGTTTAAT | Is there this motif in upstream of splicing site: GTTTAAT (1 for yes, 0 for no) |
| 290 | UmotifTTTGACT | Is there this motif in upstream of splicing site: TTTGACT (1 for yes, 0 for no) |
| 291 | UmotifTCTGA | Is there this motif in upstream of splicing site: TCTGA (1 for yes, 0 for no) |
| 292 | UmotifTCTGTT | Is there this motif in upstream of splicing site: TCTGTT (1 for yes, 0 for no) |
| 293 | UmotifTTTATTC | Is there this motif in upstream of splicing site: TTTATTC (1 for yes, 0 for no) |
| 294 | UmotifTGAAAG | Is there this motif in upstream of splicing site: TGAAAG (1 for yes, 0 for no) |
| 295 | UmotifTGTTCT | Is there this motif in upstream of splicing site: TGTTCT (1 for yes, 0 for no) |
| 296 | UmotifCTTTT | Is there this motif in upstream of splicing site: CTTTT (1 for yes, 0 for no) |
| 297 | UmotifATTTGT | Is there this motif in upstream of splicing site: ATTTGT (1 for yes, 0 for no) |
| 298 | UmotifTGAGT | Is there this motif in upstream of splicing site: TGAGT (1 for yes, 0 for no) |
| 299 | UmotifCCCCAG | Is there this motif in upstream of splicing site: CCCCAG (1 for yes, 0 for no) |
| 300 | UmotifTTGCAG | Is there this motif in upstream of splicing site: TTGCAG (1 for yes, 0 for no) |
| 301 | UmotifCTGAT | Is there this motif in upstream of splicing site: CTGAT (1 for yes, 0 for no) |
| 302 | UmotifTAATA | Is there this motif in upstream of splicing site: TAATA (1 for yes, 0 for no) |
| 303 | UmotifTCTTA | Is there this motif in upstream of splicing site: TCTTA (1 for yes, 0 for no) |
| 304 | UmotifATTCT | Is there this motif in upstream of splicing site: ATTCT (1 for yes, 0 for no) |
| 305 | UmotifATCAAA | Is there this motif in upstream of splicing site: ATCAAA (1 for yes, 0 for no) |
| 306 | UmotifCTTTA | Is there this motif in upstream of splicing site: CTTTA (1 for yes, 0 for no) |
| 307 | UmotifTTTAG | Is there this motif in upstream of splicing site: TTTAG (1 for yes, 0 for no) |
| 308 | UmotifTTGCTG | Is there this motif in upstream of splicing site: TTGCTG (1 for yes, 0 for no) |
| 309 | UmotifTTTCAT | Is there this motif in upstream of splicing site: TTTCAT (1 for yes, 0 for no) |
| 310 | UmotifCTTTCA | Is there this motif in upstream of splicing site: CTTTCA (1 for yes, 0 for no) |
| 311 | UmotifTTCTC | Is there this motif in upstream of splicing site: TTCTC (1 for yes, 0 for no) |
| 312 | UmotifTGCTT | Is there this motif in upstream of splicing site: TGCTT (1 for yes, 0 for no) |
| 313 | UmotifTCTGC | Is there this motif in upstream of splicing site: TCTGC (1 for yes, 0 for no) |
| 314 | UmotifCTGAA | Is there this motif in upstream of splicing site: CTGAA (1 for yes, 0 for no) |
| 315 | UmotifGTAGGT | Is there this motif in upstream of splicing site: GTAGGT (1 for yes, 0 for no) |
| 316 | UmotifCTAAA | Is there this motif in upstream of splicing site: CTAAA (1 for yes, 0 for no) |
| 317 | allseqif%3 | Whether the length of all sequence divisible by three |
| 318 | allseqGC | The GC contant of all sequence |
| 319 | allseqnumberTAA | The number of stopdocon TAA in all sequence |
| 320 | allseqnumberTAG | The number of stopdocon TAA in all sequence |
| 321 | allseqnumberTGA | The number of stopdocon TAA in all sequence |
| 322 | allseqfrequencyA | The frequency of A in all sequence |
| 323 | allseqfrequencyAA | The frequency of AA in all sequence |
| 324 | allseqfrequencyAAA | The frequency of AAA in all sequence |
| 325 | allseqfrequencyAAT | The frequency of AAT in all sequence |
| 326 | allseqfrequencyAAC | The frequency of AAC in all sequence |
| 327 | allseqfrequencyAAG | The frequency of AAG in all sequence |
| 328 | allseqfrequencyAT | The frequency of AT in all sequence |
| 329 | allseqfrequencyATA | The frequency of ATA in all sequence |
| 330 | allseqfrequencyATT | The frequency of ATT in all sequence |
| 331 | allseqfrequencyATC | The frequency of ATC in all sequence |
| 332 | allseqfrequencyATG | The frequency of ATG in all sequence |
| 333 | allseqfrequencyAC | The frequency of AC in all sequence |
| 334 | allseqfrequencyACA | The frequency of ACA in all sequence |
| 335 | allseqfrequencyACT | The frequency of ACT in all sequence |
| 336 | allseqfrequencyACC | The frequency of ACC in all sequence |
| 337 | allseqfrequencyACG | The frequency of ACG in all sequence |
| 338 | allseqfrequencyAG | The frequency of AG in all sequence |
| 339 | allseqfrequencyAGA | The frequency of AGA in all sequence |
| 340 | allseqfrequencyAGT | The frequency of AGT in all sequence |
| 341 | allseqfrequencyAGC | The frequency of AGC in all sequence |
| 342 | allseqfrequencyAGG | The frequency of AGG in all sequence |
| 343 | allseqfrequencyT | The frequency of T in all sequence |
| 344 | allseqfrequencyTA | The frequency of TA in all sequence |
| 345 | allseqfrequencyTAA | The frequency of TAA in all sequence |
| 346 | allseqfrequencyTAT | The frequency of TAT in all sequence |
| 347 | allseqfrequencyTAC | The frequency of TAC in all sequence |
| 348 | allseqfrequencyTAG | The frequency of TAG in all sequence |
| 349 | allseqfrequencyTT | The frequency of TT in all sequence |
| 350 | allseqfrequencyTTA | The frequency of TTA in all sequence |
| 351 | allseqfrequencyTTT | The frequency of TTT in all sequence |
| 352 | allseqfrequencyTTC | The frequency of TTC in all sequence |
| 353 | allseqfrequencyTTG | The frequency of TTG in all sequence |
| 354 | allseqfrequencyTC | The frequency of TC in all sequence |
| 355 | allseqfrequencyTCA | The frequency of TCA in all sequence |
| 356 | allseqfrequencyTCT | The frequency of TCT in all sequence |
| 357 | allseqfrequencyTCC | The frequency of TCC in all sequence |
| 358 | allseqfrequencyTCG | The frequency of TCG in all sequence |
| 359 | allseqfrequencyTG | The frequency of TG in all sequence |
| 360 | allseqfrequencyTGA | The frequency of TGA in all sequence |
| 361 | allseqfrequencyTGT | The frequency of TGT in all sequence |
| 362 | allseqfrequencyTGC | The frequency of TGC in all sequence |
| 363 | allseqfrequencyTGG | The frequency of TGG in all sequence |
| 364 | allseqfrequencyC | The frequency of C in all sequence |
| 365 | allseqfrequencyCA | The frequency of CA in all sequence |
| 366 | allseqfrequencyCAA | The frequency of CAA in all sequence |
| 367 | allseqfrequencyCAT | The frequency of CAT in all sequence |
| 368 | allseqfrequencyCAC | The frequency of CAC in all sequence |
| 369 | allseqfrequencyCAG | The frequency of CAG in all sequence |
| 370 | allseqfrequencyCT | The frequency of CT in all sequence |
| 371 | allseqfrequencyCTA | The frequency of CTA in all sequence |
| 372 | allseqfrequencyCTT | The frequency of CTT in all sequence |
| 373 | allseqfrequencyCTC | The frequency of CTC in all sequence |
| 374 | allseqfrequencyCTG | The frequency of CTG in all sequence |
| 375 | allseqfrequencyCC | The frequency of CC in all sequence |
| 376 | allseqfrequencyCCA | The frequency of CCA in all sequence |
| 377 | allseqfrequencyCCT | The frequency of CCT in all sequence |
| 378 | allseqfrequencyCCC | The frequency of CCC in all sequence |
| 379 | allseqfrequencyCCG | The frequency of CCG in all sequence |
| 380 | allseqfrequencyCG | The frequency of CG in all sequence |
| 381 | allseqfrequencyCGA | The frequency of CGA in all sequence |
| 382 | allseqfrequencyCGT | The frequency of CGT in all sequence |
| 383 | allseqfrequencyCGC | The frequency of CGC in all sequence |
| 384 | allseqfrequencyCGG | The frequency of CGG in all sequence |
| 385 | allseqfrequencyG | The frequency of G in all sequence |
| 386 | allseqfrequencyGA | The frequency of GA in all sequence |
| 387 | allseqfrequencyGAA | The frequency of GAA in all sequence |
| 388 | allseqfrequencyGAT | The frequency of GAT in all sequence |
| 389 | allseqfrequencyGAC | The frequency of GAC in all sequence |
| 390 | allseqfrequencyGAG | The frequency of GAG in all sequence |
| 391 | allseqfrequencyGT | The frequency of GT in all sequence |
| 392 | allseqfrequencyGTA | The frequency of GTA in all sequence |
| 393 | allseqfrequencyGTT | The frequency of GTT in all sequence |
| 394 | allseqfrequencyGTC | The frequency of GTC in all sequence |
| 395 | allseqfrequencyGTG | The frequency of GTG in all sequence |
| 396 | allseqfrequencyGC | The frequency of GC in all sequence |
| 397 | allseqfrequencyGCA | The frequency of GCA in all sequence |
| 398 | allseqfrequencyGCT | The frequency of GCT in all sequence |
| 399 | allseqfrequencyGCC | The frequency of GCC in all sequence |
| 400 | allseqfrequencyGCG | The frequency of GCG in all sequence |
| 401 | allseqfrequencyGG | The frequency of GG in all sequence |
| 402 | allseqfrequencyGGA | The frequency of GGA in all sequence |
| 403 | allseqfrequencyGGT | The frequency of GGT in all sequence |
| 404 | allseqfrequencyGGC | The frequency of GGC in all sequence |
| 405 | allseqfrequencyGGG | The frequency of GGG in all sequence |
| 406 | allseqdistr1%A | The distribution(position/length) of 1% A in all sequence |
| 407 | allseqdistr25%A | The distribution(position/length) of 25% A in all sequence |
| 408 | allseqdistr50%A | The distribution(position/length) of 50% A in all sequence |
| 409 | allseqdistr75%A | The distribution(position/length) of 75% A in all sequence |
| 410 | allseqdistr100%A | The distribution(position/length) of 100% A in all sequence |
| 411 | allseqdistr1%T | The distribution(position/length) of 1% T in all sequence |
| 412 | allseqdistr25%T | The distribution(position/length) of 25% T in all sequence |
| 413 | allseqdistr50%T | The distribution(position/length) of 50% T in all sequence |
| 414 | allseqdistr75%T | The distribution(position/length) of 75% T in all sequence |
| 415 | allseqdistr100%T | The distribution(position/length) of 100% T in all sequence |
| 416 | allseqdistr1%C | The distribution(position/length) of 1% C in all sequence |
| 417 | allseqdistr25%C | The distribution(position/length) of 25% C in all sequence |
| 418 | allseqdistr50%C | The distribution(position/length) of 50% C in all sequence |
| 419 | allseqdistr75%C | The distribution(position/length) of 75% C in all sequence |
| 420 | allseqdistr100%C | The distribution(position/length) of 100% C in all sequence |
| 421 | allseqdistr1%G | The distribution(position/length) of 1% G in all sequence |
| 422 | allseqdistr25%G | The distribution(position/length) of 25% G in all sequence |
| 423 | allseqdistr50%G | The distribution(position/length) of 50% G in all sequence |
| 424 | allseqdistr75%G | The distribution(position/length) of 75% G in all sequence |
| 425 | allseqdistr100%G | The distribution(position/length) of 100% G in all sequence |
| 426 | allseqdonerGT | Is there GT in doner of all sequence (1 for yes, 0 for no) |
| 427 | allseqacceptorGT | Is there GT in acceptor of all sequence (1 for yes, 0 for no) |
| 428 | allseqdonerGC | Is there GC in doner of all sequence (1 for yes, 0 for no) |
| 429 | allseqacceptorGC | Is there GC in acceptor of all sequence (1 for yes, 0 for no) |
| 430 | allseqdonerAT | Is there AT in doner of all sequence (1 for yes, 0 for no) |
| 431 | allseqacceptorAT | Is there AT in acceptor of all sequence (1 for yes, 0 for no) |
| 432 | allseqdonerAG | Is there AG in doner of all sequence (1 for yes, 0 for no) |
| 433 | allseqacceptorAG | Is there AG in acceptor of all sequence (1 for yes, 0 for no) |
| 434 | allseqdonerAC | Is there AC in doner of all sequence (1 for yes, 0 for no) |
| 435 | allseqacceptorAC | Is there AC in acceptor of all sequence (1 for yes, 0 for no) |
| 436 | asseqif%3 | Whether the length of AS region sequence divisible by three |
| 437 | asseqGC | The GC contant of AS region sequence |
| 438 | asseqnumberTAA | The number of stopdocon TAA in AS region sequence |
| 439 | asseqnumberTAG | The number of stopdocon TAA in AS region sequence |
| 440 | asseqnumberTGA | The number of stopdocon TAA in AS region sequence |
| 441 | asseqfrequencyA | The frequency of A in AS region sequence |
| 442 | asseqfrequencyAA | The frequency of AA in AS region sequence |
| 443 | asseqfrequencyAAA | The frequency of AAA in AS region sequence |
| 444 | asseqfrequencyAAT | The frequency of AAT in AS region sequence |
| 445 | asseqfrequencyAAC | The frequency of AAC in AS region sequence |
| 446 | asseqfrequencyAAG | The frequency of AAG in AS region sequence |
| 447 | asseqfrequencyAT | The frequency of AT in AS region sequence |
| 448 | asseqfrequencyATA | The frequency of ATA in AS region sequence |
| 449 | asseqfrequencyATT | The frequency of ATT in AS region sequence |
| 450 | asseqfrequencyATC | The frequency of ATC in AS region sequence |
| 451 | asseqfrequencyATG | The frequency of ATG in AS region sequence |
| 452 | asseqfrequencyAC | The frequency of AC in AS region sequence |
| 453 | asseqfrequencyACA | The frequency of ACA in AS region sequence |
| 454 | asseqfrequencyACT | The frequency of ACT in AS region sequence |
| 455 | asseqfrequencyACC | The frequency of ACC in AS region sequence |
| 456 | asseqfrequencyACG | The frequency of ACG in AS region sequence |
| 457 | asseqfrequencyAG | The frequency of AG in AS region sequence |
| 458 | asseqfrequencyAGA | The frequency of AGA in AS region sequence |
| 459 | asseqfrequencyAGT | The frequency of AGT in AS region sequence |
| 460 | asseqfrequencyAGC | The frequency of AGC in AS region sequence |
| 461 | asseqfrequencyAGG | The frequency of AGG in AS region sequence |
| 462 | asseqfrequencyT | The frequency of T in AS region sequence |
| 463 | asseqfrequencyTA | The frequency of TA in AS region sequence |
| 464 | asseqfrequencyTAA | The frequency of TAA in AS region sequence |
| 465 | asseqfrequencyTAT | The frequency of TAT in AS region sequence |
| 466 | asseqfrequencyTAC | The frequency of TAC in AS region sequence |
| 467 | asseqfrequencyTAG | The frequency of TAG in AS region sequence |
| 468 | asseqfrequencyTT | The frequency of TT in AS region sequence |
| 469 | asseqfrequencyTTA | The frequency of TTA in AS region sequence |
| 470 | asseqfrequencyTTT | The frequency of TTT in AS region sequence |
| 471 | asseqfrequencyTTC | The frequency of TTC in AS region sequence |
| 472 | asseqfrequencyTTG | The frequency of TTG in AS region sequence |
| 473 | asseqfrequencyTC | The frequency of TC in AS region sequence |
| 474 | asseqfrequencyTCA | The frequency of TCA in AS region sequence |
| 475 | asseqfrequencyTCT | The frequency of TCT in AS region sequence |
| 476 | asseqfrequencyTCC | The frequency of TCC in AS region sequence |
| 477 | asseqfrequencyTCG | The frequency of TCG in AS region sequence |
| 478 | asseqfrequencyTG | The frequency of TG in AS region sequence |
| 479 | asseqfrequencyTGA | The frequency of TGA in AS region sequence |
| 480 | asseqfrequencyTGT | The frequency of TGT in AS region sequence |
| 481 | asseqfrequencyTGC | The frequency of TGC in AS region sequence |
| 482 | asseqfrequencyTGG | The frequency of TGG in AS region sequence |
| 483 | asseqfrequencyC | The frequency of C in AS region sequence |
| 484 | asseqfrequencyCA | The frequency of CA in AS region sequence |
| 485 | asseqfrequencyCAA | The frequency of CAA in AS region sequence |
| 486 | asseqfrequencyCAT | The frequency of CAT in AS region sequence |
| 487 | asseqfrequencyCAC | The frequency of CAC in AS region sequence |
| 488 | asseqfrequencyCAG | The frequency of CAG in AS region sequence |
| 489 | asseqfrequencyCT | The frequency of CT in AS region sequence |
| 490 | asseqfrequencyCTA | The frequency of CTA in AS region sequence |
| 491 | asseqfrequencyCTT | The frequency of CTT in AS region sequence |
| 492 | asseqfrequencyCTC | The frequency of CTC in AS region sequence |
| 493 | asseqfrequencyCTG | The frequency of CTG in AS region sequence |
| 494 | asseqfrequencyCC | The frequency of CC in AS region sequence |
| 495 | asseqfrequencyCCA | The frequency of CCA in AS region sequence |
| 496 | asseqfrequencyCCT | The frequency of CCT in AS region sequence |
| 497 | asseqfrequencyCCC | The frequency of CCC in AS region sequence |
| 498 | asseqfrequencyCCG | The frequency of CCG in AS region sequence |
| 499 | asseqfrequencyCG | The frequency of CG in AS region sequence |
| 500 | asseqfrequencyCGA | The frequency of CGA in AS region sequence |
| 501 | asseqfrequencyCGT | The frequency of CGT in AS region sequence |
| 502 | asseqfrequencyCGC | The frequency of CGC in AS region sequence |
| 503 | asseqfrequencyCGG | The frequency of CGG in AS region sequence |
| 504 | asseqfrequencyG | The frequency of G in AS region sequence |
| 505 | asseqfrequencyGA | The frequency of GA in AS region sequence |
| 506 | asseqfrequencyGAA | The frequency of GAA in AS region sequence |
| 507 | asseqfrequencyGAT | The frequency of GAT in AS region sequence |
| 508 | asseqfrequencyGAC | The frequency of GAC in AS region sequence |
| 509 | asseqfrequencyGAG | The frequency of GAG in AS region sequence |
| 510 | asseqfrequencyGT | The frequency of GT in AS region sequence |
| 511 | asseqfrequencyGTA | The frequency of GTA in AS region sequence |
| 512 | asseqfrequencyGTT | The frequency of GTT in AS region sequence |
| 513 | asseqfrequencyGTC | The frequency of GTC in AS region sequence |
| 514 | asseqfrequencyGTG | The frequency of GTG in AS region sequence |
| 515 | asseqfrequencyGC | The frequency of GC in AS region sequence |
| 516 | asseqfrequencyGCA | The frequency of GCA in AS region sequence |
| 517 | asseqfrequencyGCT | The frequency of GCT in AS region sequence |
| 518 | asseqfrequencyGCC | The frequency of GCC in AS region sequence |
| 519 | asseqfrequencyGCG | The frequency of GCG in AS region sequence |
| 520 | asseqfrequencyGG | The frequency of GG in AS region sequence |
| 521 | asseqfrequencyGGA | The frequency of GGA in AS region sequence |
| 522 | asseqfrequencyGGT | The frequency of GGT in AS region sequence |
| 523 | asseqfrequencyGGC | The frequency of GGC in AS region sequence |
| 524 | asseqfrequencyGGG | The frequency of GGG in AS region sequence |
| 525 | asseqdistr1%A | The distribution(position/length) of 1% A in AS region sequence |
| 526 | asseqdistr25%A | The distribution(position/length) of 25% A in AS region sequence |
| 527 | asseqdistr50%A | The distribution(position/length) of 50% A in AS region sequence |
| 528 | asseqdistr75%A | The distribution(position/length) of 75% A in AS region sequence |
| 529 | asseqdistr100%A | The distribution(position/length) of 100% A in AS region sequence |
| 530 | asseqdistr1%T | The distribution(position/length) of 1% T in AS region sequence |
| 531 | asseqdistr25%T | The distribution(position/length) of 25% T in AS region sequence |
| 532 | asseqdistr50%T | The distribution(position/length) of 50% T in AS region sequence |
| 533 | asseqdistr75%T | The distribution(position/length) of 75% T in AS region sequence |
| 534 | asseqdistr100%T | The distribution(position/length) of 100% T in AS region sequence |
| 535 | asseqdistr1%C | The distribution(position/length) of 1% C in AS region sequence |
| 536 | asseqdistr25%C | The distribution(position/length) of 25% C in AS region sequence |
| 537 | asseqdistr50%C | The distribution(position/length) of 50% C in AS region sequence |
| 538 | asseqdistr75%C | The distribution(position/length) of 75% C in AS region sequence |
| 539 | asseqdistr100%C | The distribution(position/length) of 100% C in AS region sequence |
| 540 | asseqdistr1%G | The distribution(position/length) of 1% G in AS region sequence |
| 541 | asseqdistr25%G | The distribution(position/length) of 25% G in AS region sequence |
| 542 | asseqdistr50%G | The distribution(position/length) of 50% G in AS region sequence |
| 543 | asseqdistr75%G | The distribution(position/length) of 75% G in AS region sequence |
| 544 | asseqdistr100%G | The distribution(position/length) of 100% G in AS region sequence |
| 545 | asseqdonerGT | Is there GT in doner of AS region sequence (1 for yes, 0 for no) |
| 546 | asseqacceptorGT | Is there GT in acceptor of AS region sequence (1 for yes, 0 for no) |
| 547 | asseqdonerGC | Is there GC in doner of AS region sequence (1 for yes, 0 for no) |
| 548 | asseqacceptorGC | Is there GC in acceptor of AS region sequence (1 for yes, 0 for no) |
| 549 | asseqdonerAT | Is there AT in doner of AS region sequence (1 for yes, 0 for no) |
| 550 | asseqacceptorAT | Is there AT in acceptor of AS region sequence (1 for yes, 0 for no) |
| 551 | asseqdonerAG | Is there AG in doner of AS region sequence (1 for yes, 0 for no) |
| 552 | asseqacceptorAG | Is there AG in acceptor of AS region sequence (1 for yes, 0 for no) |
| 553 | asseqdonerAC | Is there AC in doner of AS region sequence (1 for yes, 0 for no) |
| 554 | asseqacceptorAC | Is there AC in acceptor of AS region sequence (1 for yes, 0 for no) |
| 555 | upif%3 | Whether the length of upstream sequence divisible by three |
| 556 | upGC | The GC contant of upstream sequence |
| 557 | upnumberTAA | The number of stopdocon TAA in upstream sequence |
| 558 | upnumberTAG | The number of stopdocon TAA in upstream sequence |
| 559 | upnumberTGA | The number of stopdocon TAA in upstream sequence |
| 560 | upfrequencyA | The frequency of A in upstream sequence |
| 561 | upfrequencyAA | The frequency of AA in upstream sequence |
| 562 | upfrequencyAAA | The frequency of AAA in upstream sequence |
| 563 | upfrequencyAAT | The frequency of AAT in upstream sequence |
| 564 | upfrequencyAAC | The frequency of AAC in upstream sequence |
| 565 | upfrequencyAAG | The frequency of AAG in upstream sequence |
| 566 | upfrequencyAT | The frequency of AT in upstream sequence |
| 567 | upfrequencyATA | The frequency of ATA in upstream sequence |
| 568 | upfrequencyATT | The frequency of ATT in upstream sequence |
| 569 | upfrequencyATC | The frequency of ATC in upstream sequence |
| 570 | upfrequencyATG | The frequency of ATG in upstream sequence |
| 571 | upfrequencyAC | The frequency of AC in upstream sequence |
| 572 | upfrequencyACA | The frequency of ACA in upstream sequence |
| 573 | upfrequencyACT | The frequency of ACT in upstream sequence |
| 574 | upfrequencyACC | The frequency of ACC in upstream sequence |
| 575 | upfrequencyACG | The frequency of ACG in upstream sequence |
| 576 | upfrequencyAG | The frequency of AG in upstream sequence |
| 577 | upfrequencyAGA | The frequency of AGA in upstream sequence |
| 578 | upfrequencyAGT | The frequency of AGT in upstream sequence |
| 579 | upfrequencyAGC | The frequency of AGC in upstream sequence |
| 580 | upfrequencyAGG | The frequency of AGG in upstream sequence |
| 581 | upfrequencyT | The frequency of T in upstream sequence |
| 582 | upfrequencyTA | The frequency of TA in upstream sequence |
| 583 | upfrequencyTAA | The frequency of TAA in upstream sequence |
| 584 | upfrequencyTAT | The frequency of TAT in upstream sequence |
| 585 | upfrequencyTAC | The frequency of TAC in upstream sequence |
| 586 | upfrequencyTAG | The frequency of TAG in upstream sequence |
| 587 | upfrequencyTT | The frequency of TT in upstream sequence |
| 588 | upfrequencyTTA | The frequency of TTA in upstream sequence |
| 589 | upfrequencyTTT | The frequency of TTT in upstream sequence |
| 590 | upfrequencyTTC | The frequency of TTC in upstream sequence |
| 591 | upfrequencyTTG | The frequency of TTG in upstream sequence |
| 592 | upfrequencyTC | The frequency of TC in upstream sequence |
| 593 | upfrequencyTCA | The frequency of TCA in upstream sequence |
| 594 | upfrequencyTCT | The frequency of TCT in upstream sequence |
| 595 | upfrequencyTCC | The frequency of TCC in upstream sequence |
| 596 | upfrequencyTCG | The frequency of TCG in upstream sequence |
| 597 | upfrequencyTG | The frequency of TG in upstream sequence |
| 598 | upfrequencyTGA | The frequency of TGA in upstream sequence |
| 599 | upfrequencyTGT | The frequency of TGT in upstream sequence |
| 600 | upfrequencyTGC | The frequency of TGC in upstream sequence |
| 601 | upfrequencyTGG | The frequency of TGG in upstream sequence |
| 602 | upfrequencyC | The frequency of C in upstream sequence |
| 603 | upfrequencyCA | The frequency of CA in upstream sequence |
| 604 | upfrequencyCAA | The frequency of CAA in upstream sequence |
| 605 | upfrequencyCAT | The frequency of CAT in upstream sequence |
| 606 | upfrequencyCAC | The frequency of CAC in upstream sequence |
| 607 | upfrequencyCAG | The frequency of CAG in upstream sequence |
| 608 | upfrequencyCT | The frequency of CT in upstream sequence |
| 609 | upfrequencyCTA | The frequency of CTA in upstream sequence |
| 610 | upfrequencyCTT | The frequency of CTT in upstream sequence |
| 611 | upfrequencyCTC | The frequency of CTC in upstream sequence |
| 612 | upfrequencyCTG | The frequency of CTG in upstream sequence |
| 613 | upfrequencyCC | The frequency of CC in upstream sequence |
| 614 | upfrequencyCCA | The frequency of CCA in upstream sequence |
| 615 | upfrequencyCCT | The frequency of CCT in upstream sequence |
| 616 | upfrequencyCCC | The frequency of CCC in upstream sequence |
| 617 | upfrequencyCCG | The frequency of CCG in upstream sequence |
| 618 | upfrequencyCG | The frequency of CG in upstream sequence |
| 619 | upfrequencyCGA | The frequency of CGA in upstream sequence |
| 620 | upfrequencyCGT | The frequency of CGT in upstream sequence |
| 621 | upfrequencyCGC | The frequency of CGC in upstream sequence |
| 622 | upfrequencyCGG | The frequency of CGG in upstream sequence |
| 623 | upfrequencyG | The frequency of G in upstream sequence |
| 624 | upfrequencyGA | The frequency of GA in upstream sequence |
| 625 | upfrequencyGAA | The frequency of GAA in upstream sequence |
| 626 | upfrequencyGAT | The frequency of GAT in upstream sequence |
| 627 | upfrequencyGAC | The frequency of GAC in upstream sequence |
| 628 | upfrequencyGAG | The frequency of GAG in upstream sequence |
| 629 | upfrequencyGT | The frequency of GT in upstream sequence |
| 630 | upfrequencyGTA | The frequency of GTA in upstream sequence |
| 631 | upfrequencyGTT | The frequency of GTT in upstream sequence |
| 632 | upfrequencyGTC | The frequency of GTC in upstream sequence |
| 633 | upfrequencyGTG | The frequency of GTG in upstream sequence |
| 634 | upfrequencyGC | The frequency of GC in upstream sequence |
| 635 | upfrequencyGCA | The frequency of GCA in upstream sequence |
| 636 | upfrequencyGCT | The frequency of GCT in upstream sequence |
| 637 | upfrequencyGCC | The frequency of GCC in upstream sequence |
| 638 | upfrequencyGCG | The frequency of GCG in upstream sequence |
| 639 | upfrequencyGG | The frequency of GG in upstream sequence |
| 640 | upfrequencyGGA | The frequency of GGA in upstream sequence |
| 641 | upfrequencyGGT | The frequency of GGT in upstream sequence |
| 642 | upfrequencyGGC | The frequency of GGC in upstream sequence |
| 643 | upfrequencyGGG | The frequency of GGG in upstream sequence |
| 644 | updistr1%A | The distribution(position/length) of 1% A in upstream sequence |
| 645 | updistr25%A | The distribution(position/length) of 25% A in upstream sequence |
| 646 | updistr50%A | The distribution(position/length) of 50% A in upstream sequence |
| 647 | updistr75%A | The distribution(position/length) of 75% A in upstream sequence |
| 648 | updistr100%A | The distribution(position/length) of 100% A in upstream sequence |
| 649 | updistr1%T | The distribution(position/length) of 1% T in upstream sequence |
| 650 | updistr25%T | The distribution(position/length) of 25% T in upstream sequence |
| 651 | updistr50%T | The distribution(position/length) of 50% T in upstream sequence |
| 652 | updistr75%T | The distribution(position/length) of 75% T in upstream sequence |
| 653 | updistr100%T | The distribution(position/length) of 100% T in upstream sequence |
| 654 | updistr1%C | The distribution(position/length) of 1% C in upstream sequence |
| 655 | updistr25%C | The distribution(position/length) of 25% C in upstream sequence |
| 656 | updistr50%C | The distribution(position/length) of 50% C in upstream sequence |
| 657 | updistr75%C | The distribution(position/length) of 75% C in upstream sequence |
| 658 | updistr100%C | The distribution(position/length) of 100% C in upstream sequence |
| 659 | updistr1%G | The distribution(position/length) of 1% G in upstream sequence |
| 660 | updistr25%G | The distribution(position/length) of 25% G in upstream sequence |
| 661 | updistr50%G | The distribution(position/length) of 50% G in upstream sequence |
| 662 | updistr75%G | The distribution(position/length) of 75% G in upstream sequence |
| 663 | updistr100%G | The distribution(position/length) of 100% G in upstream sequence |
| 664 | updonerGT | Is there GT in doner of upstream sequence (1 for yes, 0 for no) |
| 665 | upacceptorGT | Is there GT in acceptor of upstream sequence (1 for yes, 0 for no) |
| 666 | updonerGC | Is there GC in doner of upstream sequence (1 for yes, 0 for no) |
| 667 | upacceptorGC | Is there GC in acceptor of upstream sequence (1 for yes, 0 for no) |
| 668 | updonerAT | Is there AT in doner of upstream sequence (1 for yes, 0 for no) |
| 669 | upacceptorAT | Is there AT in acceptor of upstream sequence (1 for yes, 0 for no) |
| 670 | updonerAG | Is there AG in doner of upstream sequence (1 for yes, 0 for no) |
| 671 | upacceptorAG | Is there AG in acceptor of upstream sequence (1 for yes, 0 for no) |
| 672 | updonerAC | Is there AC in doner of upstream sequence (1 for yes, 0 for no) |
| 673 | upacceptorAC | Is there AC in acceptor of upstream sequence (1 for yes, 0 for no) |
| 674 | downif%3 | Whether the length of downstream sequence divisible by three |
| 675 | downGC | The GC contant of downstream sequence |
| 676 | downnumberTAA | The number of stopdocon TAA in downstream sequence |
| 677 | downnumberTAG | The number of stopdocon TAA in downstream sequence |
| 678 | downnumberTGA | The number of stopdocon TAA in downstream sequence |
| 679 | downfrequencyA | The frequency of A in downstream sequence |
| 680 | downfrequencyAA | The frequency of AA in downstream sequence |
| 681 | downfrequencyAAA | The frequency of AAA in downstream sequence |
| 682 | downfrequencyAAT | The frequency of AAT in downstream sequence |
| 683 | downfrequencyAAC | The frequency of AAC in downstream sequence |
| 684 | downfrequencyAAG | The frequency of AAG in downstream sequence |
| 685 | downfrequencyAT | The frequency of AT in downstream sequence |
| 686 | downfrequencyATA | The frequency of ATA in downstream sequence |
| 687 | downfrequencyATT | The frequency of ATT in downstream sequence |
| 688 | downfrequencyATC | The frequency of ATC in downstream sequence |
| 689 | downfrequencyATG | The frequency of ATG in downstream sequence |
| 690 | downfrequencyAC | The frequency of AC in downstream sequence |
| 691 | downfrequencyACA | The frequency of ACA in downstream sequence |
| 692 | downfrequencyACT | The frequency of ACT in downstream sequence |
| 693 | downfrequencyACC | The frequency of ACC in downstream sequence |
| 694 | downfrequencyACG | The frequency of ACG in downstream sequence |
| 695 | downfrequencyAG | The frequency of AG in downstream sequence |
| 696 | downfrequencyAGA | The frequency of AGA in downstream sequence |
| 697 | downfrequencyAGT | The frequency of AGT in downstream sequence |
| 698 | downfrequencyAGC | The frequency of AGC in downstream sequence |
| 699 | downfrequencyAGG | The frequency of AGG in downstream sequence |
| 700 | downfrequencyT | The frequency of T in downstream sequence |
| 701 | downfrequencyTA | The frequency of TA in downstream sequence |
| 702 | downfrequencyTAA | The frequency of TAA in downstream sequence |
| 703 | downfrequencyTAT | The frequency of TAT in downstream sequence |
| 704 | downfrequencyTAC | The frequency of TAC in downstream sequence |
| 705 | downfrequencyTAG | The frequency of TAG in downstream sequence |
| 706 | downfrequencyTT | The frequency of TT in downstream sequence |
| 707 | downfrequencyTTA | The frequency of TTA in downstream sequence |
| 708 | downfrequencyTTT | The frequency of TTT in downstream sequence |
| 709 | downfrequencyTTC | The frequency of TTC in downstream sequence |
| 710 | downfrequencyTTG | The frequency of TTG in downstream sequence |
| 711 | downfrequencyTC | The frequency of TC in downstream sequence |
| 712 | downfrequencyTCA | The frequency of TCA in downstream sequence |
| 713 | downfrequencyTCT | The frequency of TCT in downstream sequence |
| 714 | downfrequencyTCC | The frequency of TCC in downstream sequence |
| 715 | downfrequencyTCG | The frequency of TCG in downstream sequence |
| 716 | downfrequencyTG | The frequency of TG in downstream sequence |
| 717 | downfrequencyTGA | The frequency of TGA in downstream sequence |
| 718 | downfrequencyTGT | The frequency of TGT in downstream sequence |
| 719 | downfrequencyTGC | The frequency of TGC in downstream sequence |
| 720 | downfrequencyTGG | The frequency of TGG in downstream sequence |
| 721 | downfrequencyC | The frequency of C in downstream sequence |
| 722 | downfrequencyCA | The frequency of CA in downstream sequence |
| 723 | downfrequencyCAA | The frequency of CAA in downstream sequence |
| 724 | downfrequencyCAT | The frequency of CAT in downstream sequence |
| 725 | downfrequencyCAC | The frequency of CAC in downstream sequence |
| 726 | downfrequencyCAG | The frequency of CAG in downstream sequence |
| 727 | downfrequencyCT | The frequency of CT in downstream sequence |
| 728 | downfrequencyCTA | The frequency of CTA in downstream sequence |
| 729 | downfrequencyCTT | The frequency of CTT in downstream sequence |
| 730 | downfrequencyCTC | The frequency of CTC in downstream sequence |
| 731 | downfrequencyCTG | The frequency of CTG in downstream sequence |
| 732 | downfrequencyCC | The frequency of CC in downstream sequence |
| 733 | downfrequencyCCA | The frequency of CCA in downstream sequence |
| 734 | downfrequencyCCT | The frequency of CCT in downstream sequence |
| 735 | downfrequencyCCC | The frequency of CCC in downstream sequence |
| 736 | downfrequencyCCG | The frequency of CCG in downstream sequence |
| 737 | downfrequencyCG | The frequency of CG in downstream sequence |
| 738 | downfrequencyCGA | The frequency of CGA in downstream sequence |
| 739 | downfrequencyCGT | The frequency of CGT in downstream sequence |
| 740 | downfrequencyCGC | The frequency of CGC in downstream sequence |
| 741 | downfrequencyCGG | The frequency of CGG in downstream sequence |
| 742 | downfrequencyG | The frequency of G in downstream sequence |
| 743 | downfrequencyGA | The frequency of GA in downstream sequence |
| 744 | downfrequencyGAA | The frequency of GAA in downstream sequence |
| 745 | downfrequencyGAT | The frequency of GAT in downstream sequence |
| 746 | downfrequencyGAC | The frequency of GAC in downstream sequence |
| 747 | downfrequencyGAG | The frequency of GAG in downstream sequence |
| 748 | downfrequencyGT | The frequency of GT in downstream sequence |
| 749 | downfrequencyGTA | The frequency of GTA in downstream sequence |
| 750 | downfrequencyGTT | The frequency of GTT in downstream sequence |
| 751 | downfrequencyGTC | The frequency of GTC in downstream sequence |
| 752 | downfrequencyGTG | The frequency of GTG in downstream sequence |
| 753 | downfrequencyGC | The frequency of GC in downstream sequence |
| 754 | downfrequencyGCA | The frequency of GCA in downstream sequence |
| 755 | downfrequencyGCT | The frequency of GCT in downstream sequence |
| 756 | downfrequencyGCC | The frequency of GCC in downstream sequence |
| 757 | downfrequencyGCG | The frequency of GCG in downstream sequence |
| 758 | downfrequencyGG | The frequency of GG in downstream sequence |
| 759 | downfrequencyGGA | The frequency of GGA in downstream sequence |
| 760 | downfrequencyGGT | The frequency of GGT in downstream sequence |
| 761 | downfrequencyGGC | The frequency of GGC in downstream sequence |
| 762 | downfrequencyGGG | The frequency of GGG in downstream sequence |
| 763 | downdistr1%A | The distribution(position/length) of 1% A in downstream sequence |
| 764 | downdistr25%A | The distribution(position/length) of 25% A in downstream sequence |
| 765 | downdistr50%A | The distribution(position/length) of 50% A in downstream sequence |
| 766 | downdistr75%A | The distribution(position/length) of 75% A in downstream sequence |
| 767 | downdistr100%A | The distribution(position/length) of 100% A in downstream sequence |
| 768 | downdistr1%T | The distribution(position/length) of 1% T in downstream sequence |
| 769 | downdistr25%T | The distribution(position/length) of 25% T in downstream sequence |
| 770 | downdistr50%T | The distribution(position/length) of 50% T in downstream sequence |
| 771 | downdistr75%T | The distribution(position/length) of 75% T in downstream sequence |
| 772 | downdistr100%T | The distribution(position/length) of 100% T in downstream sequence |
| 773 | downdistr1%C | The distribution(position/length) of 1% C in downstream sequence |
| 774 | downdistr25%C | The distribution(position/length) of 25% C in downstream sequence |
| 775 | downdistr50%C | The distribution(position/length) of 50% C in downstream sequence |
| 776 | downdistr75%C | The distribution(position/length) of 75% C in downstream sequence |
| 777 | downdistr100%C | The distribution(position/length) of 100% C in downstream sequence |
| 778 | downdistr1%G | The distribution(position/length) of 1% G in downstream sequence |
| 779 | downdistr25%G | The distribution(position/length) of 25% G in downstream sequence |
| 780 | downdistr50%G | The distribution(position/length) of 50% G in downstream sequence |
| 781 | downdistr75%G | The distribution(position/length) of 75% G in downstream sequence |
| 782 | downdistr100%G | The distribution(position/length) of 100% G in downstream sequence |
| 783 | downdonerGT | Is there GT in doner of downstream sequence (1 for yes, 0 for no) |
| 784 | downacceptorGT | Is there GT in acceptor of downstream sequence (1 for yes, 0 for no) |
| 785 | downdonerGC | Is there GC in doner of downstream sequence (1 for yes, 0 for no) |
| 786 | downacceptorGC | Is there GC in acceptor of downstream sequence (1 for yes, 0 for no) |
| 787 | downdonerAT | Is there AT in doner of downstream sequence (1 for yes, 0 for no) |
| 788 | downacceptorAT | Is there AT in acceptor of downstream sequence (1 for yes, 0 for no) |
| 789 | downdonerAG | Is there AG in doner of downstream sequence (1 for yes, 0 for no) |
| 790 | downacceptorAG | Is there AG in acceptor of downstream sequence (1 for yes, 0 for no) |
| 791 | downdonerAC | Is there AC in doner of downstream sequence (1 for yes, 0 for no) |
| 792 | downacceptorAC | Is there AC in acceptor of downstream sequence (1 for yes, 0 for no) |
| 793 | up30as30if%3 | Whether the length of upstream30 + AS 30bp sequence divisible by three |
| 794 | up30as30GC | The GC contant of upstream30 + AS 30bp sequence |
| 795 | up30as30numberTAA | The number of stopdocon TAA in upstream30 + AS 30bp sequence |
| 796 | up30as30numberTAG | The number of stopdocon TAA in upstream30 + AS 30bp sequence |
| 797 | up30as30numberTGA | The number of stopdocon TAA in upstream30 + AS 30bp sequence |
| 798 | up30as30frequencyA | The frequency of A in upstream30 + AS 30bp sequence |
| 799 | up30as30frequencyAA | The frequency of AA in upstream30 + AS 30bp sequence |
| 800 | up30as30frequencyAAA | The frequency of AAA in upstream30 + AS 30bp sequence |
| 801 | up30as30frequencyAAT | The frequency of AAT in upstream30 + AS 30bp sequence |
| 802 | up30as30frequencyAAC | The frequency of AAC in upstream30 + AS 30bp sequence |
| 803 | up30as30frequencyAAG | The frequency of AAG in upstream30 + AS 30bp sequence |
| 804 | up30as30frequencyAT | The frequency of AT in upstream30 + AS 30bp sequence |
| 805 | up30as30frequencyATA | The frequency of ATA in upstream30 + AS 30bp sequence |
| 806 | up30as30frequencyATT | The frequency of ATT in upstream30 + AS 30bp sequence |
| 807 | up30as30frequencyATC | The frequency of ATC in upstream30 + AS 30bp sequence |
| 808 | up30as30frequencyATG | The frequency of ATG in upstream30 + AS 30bp sequence |
| 809 | up30as30frequencyAC | The frequency of AC in upstream30 + AS 30bp sequence |
| 810 | up30as30frequencyACA | The frequency of ACA in upstream30 + AS 30bp sequence |
| 811 | up30as30frequencyACT | The frequency of ACT in upstream30 + AS 30bp sequence |
| 812 | up30as30frequencyACC | The frequency of ACC in upstream30 + AS 30bp sequence |
| 813 | up30as30frequencyACG | The frequency of ACG in upstream30 + AS 30bp sequence |
| 814 | up30as30frequencyAG | The frequency of AG in upstream30 + AS 30bp sequence |
| 815 | up30as30frequencyAGA | The frequency of AGA in upstream30 + AS 30bp sequence |
| 816 | up30as30frequencyAGT | The frequency of AGT in upstream30 + AS 30bp sequence |
| 817 | up30as30frequencyAGC | The frequency of AGC in upstream30 + AS 30bp sequence |
| 818 | up30as30frequencyAGG | The frequency of AGG in upstream30 + AS 30bp sequence |
| 819 | up30as30frequencyT | The frequency of T in upstream30 + AS 30bp sequence |
| 820 | up30as30frequencyTA | The frequency of TA in upstream30 + AS 30bp sequence |
| 821 | up30as30frequencyTAA | The frequency of TAA in upstream30 + AS 30bp sequence |
| 822 | up30as30frequencyTAT | The frequency of TAT in upstream30 + AS 30bp sequence |
| 823 | up30as30frequencyTAC | The frequency of TAC in upstream30 + AS 30bp sequence |
| 824 | up30as30frequencyTAG | The frequency of TAG in upstream30 + AS 30bp sequence |
| 825 | up30as30frequencyTT | The frequency of TT in upstream30 + AS 30bp sequence |
| 826 | up30as30frequencyTTA | The frequency of TTA in upstream30 + AS 30bp sequence |
| 827 | up30as30frequencyTTT | The frequency of TTT in upstream30 + AS 30bp sequence |
| 828 | up30as30frequencyTTC | The frequency of TTC in upstream30 + AS 30bp sequence |
| 829 | up30as30frequencyTTG | The frequency of TTG in upstream30 + AS 30bp sequence |
| 830 | up30as30frequencyTC | The frequency of TC in upstream30 + AS 30bp sequence |
| 831 | up30as30frequencyTCA | The frequency of TCA in upstream30 + AS 30bp sequence |
| 832 | up30as30frequencyTCT | The frequency of TCT in upstream30 + AS 30bp sequence |
| 833 | up30as30frequencyTCC | The frequency of TCC in upstream30 + AS 30bp sequence |
| 834 | up30as30frequencyTCG | The frequency of TCG in upstream30 + AS 30bp sequence |
| 835 | up30as30frequencyTG | The frequency of TG in upstream30 + AS 30bp sequence |
| 836 | up30as30frequencyTGA | The frequency of TGA in upstream30 + AS 30bp sequence |
| 837 | up30as30frequencyTGT | The frequency of TGT in upstream30 + AS 30bp sequence |
| 838 | up30as30frequencyTGC | The frequency of TGC in upstream30 + AS 30bp sequence |
| 839 | up30as30frequencyTGG | The frequency of TGG in upstream30 + AS 30bp sequence |
| 840 | up30as30frequencyC | The frequency of C in upstream30 + AS 30bp sequence |
| 841 | up30as30frequencyCA | The frequency of CA in upstream30 + AS 30bp sequence |
| 842 | up30as30frequencyCAA | The frequency of CAA in upstream30 + AS 30bp sequence |
| 843 | up30as30frequencyCAT | The frequency of CAT in upstream30 + AS 30bp sequence |
| 844 | up30as30frequencyCAC | The frequency of CAC in upstream30 + AS 30bp sequence |
| 845 | up30as30frequencyCAG | The frequency of CAG in upstream30 + AS 30bp sequence |
| 846 | up30as30frequencyCT | The frequency of CT in upstream30 + AS 30bp sequence |
| 847 | up30as30frequencyCTA | The frequency of CTA in upstream30 + AS 30bp sequence |
| 848 | up30as30frequencyCTT | The frequency of CTT in upstream30 + AS 30bp sequence |
| 849 | up30as30frequencyCTC | The frequency of CTC in upstream30 + AS 30bp sequence |
| 850 | up30as30frequencyCTG | The frequency of CTG in upstream30 + AS 30bp sequence |
| 851 | up30as30frequencyCC | The frequency of CC in upstream30 + AS 30bp sequence |
| 852 | up30as30frequencyCCA | The frequency of CCA in upstream30 + AS 30bp sequence |
| 853 | up30as30frequencyCCT | The frequency of CCT in upstream30 + AS 30bp sequence |
| 854 | up30as30frequencyCCC | The frequency of CCC in upstream30 + AS 30bp sequence |
| 855 | up30as30frequencyCCG | The frequency of CCG in upstream30 + AS 30bp sequence |
| 856 | up30as30frequencyCG | The frequency of CG in upstream30 + AS 30bp sequence |
| 857 | up30as30frequencyCGA | The frequency of CGA in upstream30 + AS 30bp sequence |
| 858 | up30as30frequencyCGT | The frequency of CGT in upstream30 + AS 30bp sequence |
| 859 | up30as30frequencyCGC | The frequency of CGC in upstream30 + AS 30bp sequence |
| 860 | up30as30frequencyCGG | The frequency of CGG in upstream30 + AS 30bp sequence |
| 861 | up30as30frequencyG | The frequency of G in upstream30 + AS 30bp sequence |
| 862 | up30as30frequencyGA | The frequency of GA in upstream30 + AS 30bp sequence |
| 863 | up30as30frequencyGAA | The frequency of GAA in upstream30 + AS 30bp sequence |
| 864 | up30as30frequencyGAT | The frequency of GAT in upstream30 + AS 30bp sequence |
| 865 | up30as30frequencyGAC | The frequency of GAC in upstream30 + AS 30bp sequence |
| 866 | up30as30frequencyGAG | The frequency of GAG in upstream30 + AS 30bp sequence |
| 867 | up30as30frequencyGT | The frequency of GT in upstream30 + AS 30bp sequence |
| 868 | up30as30frequencyGTA | The frequency of GTA in upstream30 + AS 30bp sequence |
| 869 | up30as30frequencyGTT | The frequency of GTT in upstream30 + AS 30bp sequence |
| 870 | up30as30frequencyGTC | The frequency of GTC in upstream30 + AS 30bp sequence |
| 871 | up30as30frequencyGTG | The frequency of GTG in upstream30 + AS 30bp sequence |
| 872 | up30as30frequencyGC | The frequency of GC in upstream30 + AS 30bp sequence |
| 873 | up30as30frequencyGCA | The frequency of GCA in upstream30 + AS 30bp sequence |
| 874 | up30as30frequencyGCT | The frequency of GCT in upstream30 + AS 30bp sequence |
| 875 | up30as30frequencyGCC | The frequency of GCC in upstream30 + AS 30bp sequence |
| 876 | up30as30frequencyGCG | The frequency of GCG in upstream30 + AS 30bp sequence |
| 877 | up30as30frequencyGG | The frequency of GG in upstream30 + AS 30bp sequence |
| 878 | up30as30frequencyGGA | The frequency of GGA in upstream30 + AS 30bp sequence |
| 879 | up30as30frequencyGGT | The frequency of GGT in upstream30 + AS 30bp sequence |
| 880 | up30as30frequencyGGC | The frequency of GGC in upstream30 + AS 30bp sequence |
| 881 | up30as30frequencyGGG | The frequency of GGG in upstream30 + AS 30bp sequence |
| 882 | up30as30distr1%A | The distribution(position/length) of 1% A in upstream30 + AS 30bp sequence |
| 883 | up30as30distr25%A | The distribution(position/length) of 25% A in upstream30 + AS 30bp sequence |
| 884 | up30as30distr50%A | The distribution(position/length) of 50% A in upstream30 + AS 30bp sequence |
| 885 | up30as30distr75%A | The distribution(position/length) of 75% A in upstream30 + AS 30bp sequence |
| 886 | up30as30distr100%A | The distribution(position/length) of 100% A in upstream30 + AS 30bp sequence |
| 887 | up30as30distr1%T | The distribution(position/length) of 1% T in upstream30 + AS 30bp sequence |
| 888 | up30as30distr25%T | The distribution(position/length) of 25% T in upstream30 + AS 30bp sequence |
| 889 | up30as30distr50%T | The distribution(position/length) of 50% T in upstream30 + AS 30bp sequence |
| 890 | up30as30distr75%T | The distribution(position/length) of 75% T in upstream30 + AS 30bp sequence |
| 891 | up30as30distr100%T | The distribution(position/length) of 100% T in upstream30 + AS 30bp sequence |
| 892 | up30as30distr1%C | The distribution(position/length) of 1% C in upstream30 + AS 30bp sequence |
| 893 | up30as30distr25%C | The distribution(position/length) of 25% C in upstream30 + AS 30bp sequence |
| 894 | up30as30distr50%C | The distribution(position/length) of 50% C in upstream30 + AS 30bp sequence |
| 895 | up30as30distr75%C | The distribution(position/length) of 75% C in upstream30 + AS 30bp sequence |
| 896 | up30as30distr100%C | The distribution(position/length) of 100% C in upstream30 + AS 30bp sequence |
| 897 | up30as30distr1%G | The distribution(position/length) of 1% G in upstream30 + AS 30bp sequence |
| 898 | up30as30distr25%G | The distribution(position/length) of 25% G in upstream30 + AS 30bp sequence |
| 899 | up30as30distr50%G | The distribution(position/length) of 50% G in upstream30 + AS 30bp sequence |
| 900 | up30as30distr75%G | The distribution(position/length) of 75% G in upstream30 + AS 30bp sequence |
| 901 | up30as30distr100%G | The distribution(position/length) of 100% G in upstream30 + AS 30bp sequence |
| 902 | up30as30donerGT | Is there GT in doner of upstream30 + AS 30bp sequence (1 for yes, 0 for no) |
| 903 | up30as30acceptorGT | Is there GT in acceptor of upstream30 + AS 30bp sequence (1 for yes, 0 for no) |
| 904 | up30as30donerGC | Is there GC in doner of upstream30 + AS 30bp sequence (1 for yes, 0 for no) |
| 905 | up30as30acceptorGC | Is there GC in acceptor of upstream30 + AS 30bp sequence (1 for yes, 0 for no) |
| 906 | up30as30donerAT | Is there AT in doner of upstream30 + AS 30bp sequence (1 for yes, 0 for no) |
| 907 | up30as30acceptorAT | Is there AT in acceptor of upstream30 + AS 30bp sequence (1 for yes, 0 for no) |
| 908 | up30as30donerAG | Is there AG in doner of upstream30 + AS 30bp sequence (1 for yes, 0 for no) |
| 909 | up30as30acceptorAG | Is there AG in acceptor of upstream30 + AS 30bp sequence (1 for yes, 0 for no) |
| 910 | up30as30donerAC | Is there AC in doner of upstream30 + AS 30bp sequence (1 for yes, 0 for no) |
| 911 | up30as30acceptorAC | Is there AC in acceptor of upstream30 + AS 30bp sequence (1 for yes, 0 for no) |
| 912 | up30down30if%3 | Whether the length of upstream30 + downstream30 sequence divisible by three |
| 913 | up30down30GC | The GC contant of upstream30 + downstream30 sequence |
| 914 | up30down30numberTAA | The number of stopdocon TAA in upstream30 + downstream30 sequence |
| 915 | up30down30numberTAG | The number of stopdocon TAA in upstream30 + downstream30 sequence |
| 916 | up30down30numberTGA | The number of stopdocon TAA in upstream30 + downstream30 sequence |
| 917 | up30down30frequencyA | The frequency of A in upstream30 + downstream30 sequence |
| 918 | up30down30frequencyAA | The frequency of AA in upstream30 + downstream30 sequence |
| 919 | up30down30frequencyAAA | The frequency of AAA in upstream30 + downstream30 sequence |
| 920 | up30down30frequencyAAT | The frequency of AAT in upstream30 + downstream30 sequence |
| 921 | up30down30frequencyAAC | The frequency of AAC in upstream30 + downstream30 sequence |
| 922 | up30down30frequencyAAG | The frequency of AAG in upstream30 + downstream30 sequence |
| 923 | up30down30frequencyAT | The frequency of AT in upstream30 + downstream30 sequence |
| 924 | up30down30frequencyATA | The frequency of ATA in upstream30 + downstream30 sequence |
| 925 | up30down30frequencyATT | The frequency of ATT in upstream30 + downstream30 sequence |
| 926 | up30down30frequencyATC | The frequency of ATC in upstream30 + downstream30 sequence |
| 927 | up30down30frequencyATG | The frequency of ATG in upstream30 + downstream30 sequence |
| 928 | up30down30frequencyAC | The frequency of AC in upstream30 + downstream30 sequence |
| 929 | up30down30frequencyACA | The frequency of ACA in upstream30 + downstream30 sequence |
| 930 | up30down30frequencyACT | The frequency of ACT in upstream30 + downstream30 sequence |
| 931 | up30down30frequencyACC | The frequency of ACC in upstream30 + downstream30 sequence |
| 932 | up30down30frequencyACG | The frequency of ACG in upstream30 + downstream30 sequence |
| 933 | up30down30frequencyAG | The frequency of AG in upstream30 + downstream30 sequence |
| 934 | up30down30frequencyAGA | The frequency of AGA in upstream30 + downstream30 sequence |
| 935 | up30down30frequencyAGT | The frequency of AGT in upstream30 + downstream30 sequence |
| 936 | up30down30frequencyAGC | The frequency of AGC in upstream30 + downstream30 sequence |
| 937 | up30down30frequencyAGG | The frequency of AGG in upstream30 + downstream30 sequence |
| 938 | up30down30frequencyT | The frequency of T in upstream30 + downstream30 sequence |
| 939 | up30down30frequencyTA | The frequency of TA in upstream30 + downstream30 sequence |
| 940 | up30down30frequencyTAA | The frequency of TAA in upstream30 + downstream30 sequence |
| 941 | up30down30frequencyTAT | The frequency of TAT in upstream30 + downstream30 sequence |
| 942 | up30down30frequencyTAC | The frequency of TAC in upstream30 + downstream30 sequence |
| 943 | up30down30frequencyTAG | The frequency of TAG in upstream30 + downstream30 sequence |
| 944 | up30down30frequencyTT | The frequency of TT in upstream30 + downstream30 sequence |
| 945 | up30down30frequencyTTA | The frequency of TTA in upstream30 + downstream30 sequence |
| 946 | up30down30frequencyTTT | The frequency of TTT in upstream30 + downstream30 sequence |
| 947 | up30down30frequencyTTC | The frequency of TTC in upstream30 + downstream30 sequence |
| 948 | up30down30frequencyTTG | The frequency of TTG in upstream30 + downstream30 sequence |
| 949 | up30down30frequencyTC | The frequency of TC in upstream30 + downstream30 sequence |
| 950 | up30down30frequencyTCA | The frequency of TCA in upstream30 + downstream30 sequence |
| 951 | up30down30frequencyTCT | The frequency of TCT in upstream30 + downstream30 sequence |
| 952 | up30down30frequencyTCC | The frequency of TCC in upstream30 + downstream30 sequence |
| 953 | up30down30frequencyTCG | The frequency of TCG in upstream30 + downstream30 sequence |
| 954 | up30down30frequencyTG | The frequency of TG in upstream30 + downstream30 sequence |
| 955 | up30down30frequencyTGA | The frequency of TGA in upstream30 + downstream30 sequence |
| 956 | up30down30frequencyTGT | The frequency of TGT in upstream30 + downstream30 sequence |
| 957 | up30down30frequencyTGC | The frequency of TGC in upstream30 + downstream30 sequence |
| 958 | up30down30frequencyTGG | The frequency of TGG in upstream30 + downstream30 sequence |
| 959 | up30down30frequencyC | The frequency of C in upstream30 + downstream30 sequence |
| 960 | up30down30frequencyCA | The frequency of CA in upstream30 + downstream30 sequence |
| 961 | up30down30frequencyCAA | The frequency of CAA in upstream30 + downstream30 sequence |
| 962 | up30down30frequencyCAT | The frequency of CAT in upstream30 + downstream30 sequence |
| 963 | up30down30frequencyCAC | The frequency of CAC in upstream30 + downstream30 sequence |
| 964 | up30down30frequencyCAG | The frequency of CAG in upstream30 + downstream30 sequence |
| 965 | up30down30frequencyCT | The frequency of CT in upstream30 + downstream30 sequence |
| 966 | up30down30frequencyCTA | The frequency of CTA in upstream30 + downstream30 sequence |
| 967 | up30down30frequencyCTT | The frequency of CTT in upstream30 + downstream30 sequence |
| 968 | up30down30frequencyCTC | The frequency of CTC in upstream30 + downstream30 sequence |
| 969 | up30down30frequencyCTG | The frequency of CTG in upstream30 + downstream30 sequence |
| 970 | up30down30frequencyCC | The frequency of CC in upstream30 + downstream30 sequence |
| 971 | up30down30frequencyCCA | The frequency of CCA in upstream30 + downstream30 sequence |
| 972 | up30down30frequencyCCT | The frequency of CCT in upstream30 + downstream30 sequence |
| 973 | up30down30frequencyCCC | The frequency of CCC in upstream30 + downstream30 sequence |
| 974 | up30down30frequencyCCG | The frequency of CCG in upstream30 + downstream30 sequence |
| 975 | up30down30frequencyCG | The frequency of CG in upstream30 + downstream30 sequence |
| 976 | up30down30frequencyCGA | The frequency of CGA in upstream30 + downstream30 sequence |
| 977 | up30down30frequencyCGT | The frequency of CGT in upstream30 + downstream30 sequence |
| 978 | up30down30frequencyCGC | The frequency of CGC in upstream30 + downstream30 sequence |
| 979 | up30down30frequencyCGG | The frequency of CGG in upstream30 + downstream30 sequence |
| 980 | up30down30frequencyG | The frequency of G in upstream30 + downstream30 sequence |
| 981 | up30down30frequencyGA | The frequency of GA in upstream30 + downstream30 sequence |
| 982 | up30down30frequencyGAA | The frequency of GAA in upstream30 + downstream30 sequence |
| 983 | up30down30frequencyGAT | The frequency of GAT in upstream30 + downstream30 sequence |
| 984 | up30down30frequencyGAC | The frequency of GAC in upstream30 + downstream30 sequence |
| 985 | up30down30frequencyGAG | The frequency of GAG in upstream30 + downstream30 sequence |
| 986 | up30down30frequencyGT | The frequency of GT in upstream30 + downstream30 sequence |
| 987 | up30down30frequencyGTA | The frequency of GTA in upstream30 + downstream30 sequence |
| 988 | up30down30frequencyGTT | The frequency of GTT in upstream30 + downstream30 sequence |
| 989 | up30down30frequencyGTC | The frequency of GTC in upstream30 + downstream30 sequence |
| 990 | up30down30frequencyGTG | The frequency of GTG in upstream30 + downstream30 sequence |
| 991 | up30down30frequencyGC | The frequency of GC in upstream30 + downstream30 sequence |
| 992 | up30down30frequencyGCA | The frequency of GCA in upstream30 + downstream30 sequence |
| 993 | up30down30frequencyGCT | The frequency of GCT in upstream30 + downstream30 sequence |
| 994 | up30down30frequencyGCC | The frequency of GCC in upstream30 + downstream30 sequence |
| 995 | up30down30frequencyGCG | The frequency of GCG in upstream30 + downstream30 sequence |
| 996 | up30down30frequencyGG | The frequency of GG in upstream30 + downstream30 sequence |
| 997 | up30down30frequencyGGA | The frequency of GGA in upstream30 + downstream30 sequence |
| 998 | up30down30frequencyGGT | The frequency of GGT in upstream30 + downstream30 sequence |
| 999 | up30down30frequencyGGC | The frequency of GGC in upstream30 + downstream30 sequence |
| 1000 | up30down30frequencyGGG | The frequency of GGG in upstream30 + downstream30 sequence |
| 1001 | up30down30distr1%A | The distribution(position/length) of 1% A in upstream30 + downstream30 sequence |
| 1002 | up30down30distr25%A | The distribution(position/length) of 25% A in upstream30 + downstream30 sequence |
| 1003 | up30down30distr50%A | The distribution(position/length) of 50% A in upstream30 + downstream30 sequence |
| 1004 | up30down30distr75%A | The distribution(position/length) of 75% A in upstream30 + downstream30 sequence |
| 1005 | up30down30distr100%A | The distribution(position/length) of 100% A in upstream30 + downstream30 sequence |
| 1006 | up30down30distr1%T | The distribution(position/length) of 1% T in upstream30 + downstream30 sequence |
| 1007 | up30down30distr25%T | The distribution(position/length) of 25% T in upstream30 + downstream30 sequence |
| 1008 | up30down30distr50%T | The distribution(position/length) of 50% T in upstream30 + downstream30 sequence |
| 1009 | up30down30distr75%T | The distribution(position/length) of 75% T in upstream30 + downstream30 sequence |
| 1010 | up30down30distr100%T | The distribution(position/length) of 100% T in upstream30 + downstream30 sequence |
| 1011 | up30down30distr1%C | The distribution(position/length) of 1% C in upstream30 + downstream30 sequence |
| 1012 | up30down30distr25%C | The distribution(position/length) of 25% C in upstream30 + downstream30 sequence |
| 1013 | up30down30distr50%C | The distribution(position/length) of 50% C in upstream30 + downstream30 sequence |
| 1014 | up30down30distr75%C | The distribution(position/length) of 75% C in upstream30 + downstream30 sequence |
| 1015 | up30down30distr100%C | The distribution(position/length) of 100% C in upstream30 + downstream30 sequence |
| 1016 | up30down30distr1%G | The distribution(position/length) of 1% G in upstream30 + downstream30 sequence |
| 1017 | up30down30distr25%G | The distribution(position/length) of 25% G in upstream30 + downstream30 sequence |
| 1018 | up30down30distr50%G | The distribution(position/length) of 50% G in upstream30 + downstream30 sequence |
| 1019 | up30down30distr75%G | The distribution(position/length) of 75% G in upstream30 + downstream30 sequence |
| 1020 | up30down30distr100%G | The distribution(position/length) of 100% G in upstream30 + downstream30 sequence |
| 1021 | up30down30donerGT | Is there GT in doner of upstream30 + downstream30 sequence (1 for yes, 0 for no) |
| 1022 | up30down30acceptorGT | Is there GT in acceptor of upstream30 + downstream30 sequence (1 for yes, 0 for no) |
| 1023 | up30down30donerGC | Is there GC in doner of upstream30 + downstream30 sequence (1 for yes, 0 for no) |
| 1024 | up30down30acceptorGC | Is there GC in acceptor of upstream30 + downstream30 sequence (1 for yes, 0 for no) |
| 1025 | up30down30donerAT | Is there AT in doner of upstream30 + downstream30 sequence (1 for yes, 0 for no) |
| 1026 | up30down30acceptorAT | Is there AT in acceptor of upstream30 + downstream30 sequence (1 for yes, 0 for no) |
| 1027 | up30down30donerAG | Is there AG in doner of upstream30 + downstream30 sequence (1 for yes, 0 for no) |
| 1028 | up30down30acceptorAG | Is there AG in acceptor of upstream30 + downstream30 sequence (1 for yes, 0 for no) |
| 1029 | up30down30donerAC | Is there AC in doner of upstream30 + downstream30 sequence (1 for yes, 0 for no) |
| 1030 | up30down30acceptorAC | Is there AC in acceptor of upstream30 + downstream30 sequence (1 for yes, 0 for no) |
| 1031 | as30down30if%3 | Whether the length of AS 30bp + downstream 30bp sequence divisible by three |
| 1032 | as30down30GC | The GC contant of AS 30bp + downstream30bp sequence |
| 1033 | as30down30numberTAA | The number of stopdocon TAA in AS 30bp + downstream30bp sequence |
| 1034 | as30down30numberTAG | The number of stopdocon TAA in AS 30bp + downstream30bp sequence |
| 1035 | as30down30numberTGA | The number of stopdocon TAA in AS 30bp + downstream30bp sequence |
| 1036 | as30down30frequencyA | The frequency of A in AS 30bp + downstream30bp sequence |
| 1037 | as30down30frequencyAA | The frequency of AA in AS 30bp + downstream30bp sequence |
| 1038 | as30down30frequencyAAA | The frequency of AAA in AS 30bp + downstream30bp sequence |
| 1039 | as30down30frequencyAAT | The frequency of AAT in AS 30bp + downstream30bp sequence |
| 1040 | as30down30frequencyAAC | The frequency of AAC in AS 30bp + downstream30bp sequence |
| 1041 | as30down30frequencyAAG | The frequency of AAG in AS 30bp + downstream30bp sequence |
| 1042 | as30down30frequencyAT | The frequency of AT in AS 30bp + downstream30bp sequence |
| 1043 | as30down30frequencyATA | The frequency of ATA in AS 30bp + downstream30bp sequence |
| 1044 | as30down30frequencyATT | The frequency of ATT in AS 30bp + downstream30bp sequence |
| 1045 | as30down30frequencyATC | The frequency of ATC in AS 30bp + downstream30bp sequence |
| 1046 | as30down30frequencyATG | The frequency of ATG in AS 30bp + downstream30bp sequence |
| 1047 | as30down30frequencyAC | The frequency of AC in AS 30bp + downstream30bp sequence |
| 1048 | as30down30frequencyACA | The frequency of ACA in AS 30bp + downstream30bp sequence |
| 1049 | as30down30frequencyACT | The frequency of ACT in AS 30bp + downstream30bp sequence |
| 1050 | as30down30frequencyACC | The frequency of ACC in AS 30bp + downstream30bp sequence |
| 1051 | as30down30frequencyACG | The frequency of ACG in AS 30bp + downstream30bp sequence |
| 1052 | as30down30frequencyAG | The frequency of AG in AS 30bp + downstream30bp sequence |
| 1053 | as30down30frequencyAGA | The frequency of AGA in AS 30bp + downstream30bp sequence |
| 1054 | as30down30frequencyAGT | The frequency of AGT in AS 30bp + downstream30bp sequence |
| 1055 | as30down30frequencyAGC | The frequency of AGC in AS 30bp + downstream30bp sequence |
| 1056 | as30down30frequencyAGG | The frequency of AGG in AS 30bp + downstream30bp sequence |
| 1057 | as30down30frequencyT | The frequency of T in AS 30bp + downstream30bp sequence |
| 1058 | as30down30frequencyTA | The frequency of TA in AS 30bp + downstream30bp sequence |
| 1059 | as30down30frequencyTAA | The frequency of TAA in AS 30bp + downstream30bp sequence |
| 1060 | as30down30frequencyTAT | The frequency of TAT in AS 30bp + downstream30bp sequence |
| 1061 | as30down30frequencyTAC | The frequency of TAC in AS 30bp + downstream30bp sequence |
| 1062 | as30down30frequencyTAG | The frequency of TAG in AS 30bp + downstream30bp sequence |
| 1063 | as30down30frequencyTT | The frequency of TT in AS 30bp + downstream30bp sequence |
| 1064 | as30down30frequencyTTA | The frequency of TTA in AS 30bp + downstream30bp sequence |
| 1065 | as30down30frequencyTTT | The frequency of TTT in AS 30bp + downstream30bp sequence |
| 1066 | as30down30frequencyTTC | The frequency of TTC in AS 30bp + downstream30bp sequence |
| 1067 | as30down30frequencyTTG | The frequency of TTG in AS 30bp + downstream30bp sequence |
| 1068 | as30down30frequencyTC | The frequency of TC in AS 30bp + downstream30bp sequence |
| 1069 | as30down30frequencyTCA | The frequency of TCA in AS 30bp + downstream30bp sequence |
| 1070 | as30down30frequencyTCT | The frequency of TCT in AS 30bp + downstream30bp sequence |
| 1071 | as30down30frequencyTCC | The frequency of TCC in AS 30bp + downstream30bp sequence |
| 1072 | as30down30frequencyTCG | The frequency of TCG in AS 30bp + downstream30bp sequence |
| 1073 | as30down30frequencyTG | The frequency of TG in AS 30bp + downstream30bp sequence |
| 1074 | as30down30frequencyTGA | The frequency of TGA in AS 30bp + downstream30bp sequence |
| 1075 | as30down30frequencyTGT | The frequency of TGT in AS 30bp + downstream30bp sequence |
| 1076 | as30down30frequencyTGC | The frequency of TGC in AS 30bp + downstream30bp sequence |
| 1077 | as30down30frequencyTGG | The frequency of TGG in AS 30bp + downstream30bp sequence |
| 1078 | as30down30frequencyC | The frequency of C in AS 30bp + downstream30bp sequence |
| 1079 | as30down30frequencyCA | The frequency of CA in AS 30bp + downstream30bp sequence |
| 1080 | as30down30frequencyCAA | The frequency of CAA in AS 30bp + downstream30bp sequence |
| 1081 | as30down30frequencyCAT | The frequency of CAT in AS 30bp + downstream30bp sequence |
| 1082 | as30down30frequencyCAC | The frequency of CAC in AS 30bp + downstream30bp sequence |
| 1083 | as30down30frequencyCAG | The frequency of CAG in AS 30bp + downstream30bp sequence |
| 1084 | as30down30frequencyCT | The frequency of CT in AS 30bp + downstream30bp sequence |
| 1085 | as30down30frequencyCTA | The frequency of CTA in AS 30bp + downstream30bp sequence |
| 1086 | as30down30frequencyCTT | The frequency of CTT in AS 30bp + downstream30bp sequence |
| 1087 | as30down30frequencyCTC | The frequency of CTC in AS 30bp + downstream30bp sequence |
| 1088 | as30down30frequencyCTG | The frequency of CTG in AS 30bp + downstream30bp sequence |
| 1089 | as30down30frequencyCC | The frequency of CC in AS 30bp + downstream30bp sequence |
| 1090 | as30down30frequencyCCA | The frequency of CCA in AS 30bp + downstream30bp sequence |
| 1091 | as30down30frequencyCCT | The frequency of CCT in AS 30bp + downstream30bp sequence |
| 1092 | as30down30frequencyCCC | The frequency of CCC in AS 30bp + downstream30bp sequence |
| 1093 | as30down30frequencyCCG | The frequency of CCG in AS 30bp + downstream30bp sequence |
| 1094 | as30down30frequencyCG | The frequency of CG in AS 30bp + downstream30bp sequence |
| 1095 | as30down30frequencyCGA | The frequency of CGA in AS 30bp + downstream30bp sequence |
| 1096 | as30down30frequencyCGT | The frequency of CGT in AS 30bp + downstream30bp sequence |
| 1097 | as30down30frequencyCGC | The frequency of CGC in AS 30bp + downstream30bp sequence |
| 1098 | as30down30frequencyCGG | The frequency of CGG in AS 30bp + downstream30bp sequence |
| 1099 | as30down30frequencyG | The frequency of G in AS 30bp + downstream30bp sequence |
| 1100 | as30down30frequencyGA | The frequency of GA in AS 30bp + downstream30bp sequence |
| 1101 | as30down30frequencyGAA | The frequency of GAA in AS 30bp + downstream30bp sequence |
| 1102 | as30down30frequencyGAT | The frequency of GAT in AS 30bp + downstream30bp sequence |
| 1103 | as30down30frequencyGAC | The frequency of GAC in AS 30bp + downstream30bp sequence |
| 1104 | as30down30frequencyGAG | The frequency of GAG in AS 30bp + downstream30bp sequence |
| 1105 | as30down30frequencyGT | The frequency of GT in AS 30bp + downstream30bp sequence |
| 1106 | as30down30frequencyGTA | The frequency of GTA in AS 30bp + downstream30bp sequence |
| 1107 | as30down30frequencyGTT | The frequency of GTT in AS 30bp + downstream30bp sequence |
| 1108 | as30down30frequencyGTC | The frequency of GTC in AS 30bp + downstream30bp sequence |
| 1109 | as30down30frequencyGTG | The frequency of GTG in AS 30bp + downstream30bp sequence |
| 1110 | as30down30frequencyGC | The frequency of GC in AS 30bp + downstream30bp sequence |
| 1111 | as30down30frequencyGCA | The frequency of GCA in AS 30bp + downstream30bp sequence |
| 1112 | as30down30frequencyGCT | The frequency of GCT in AS 30bp + downstream30bp sequence |
| 1113 | as30down30frequencyGCC | The frequency of GCC in AS 30bp + downstream30bp sequence |
| 1114 | as30down30frequencyGCG | The frequency of GCG in AS 30bp + downstream30bp sequence |
| 1115 | as30down30frequencyGG | The frequency of GG in AS 30bp + downstream30bp sequence |
| 1116 | as30down30frequencyGGA | The frequency of GGA in AS 30bp + downstream30bp sequence |
| 1117 | as30down30frequencyGGT | The frequency of GGT in AS 30bp + downstream30bp sequence |
| 1118 | as30down30frequencyGGC | The frequency of GGC in AS 30bp + downstream30bp sequence |
| 1119 | as30down30frequencyGGG | The frequency of GGG in AS 30bp + downstream30bp sequence |
| 1120 | as30down30distr1%A | The distribution(position/length) of 1% A in AS 30bp + downstream30bp sequence |
| 1121 | as30down30distr25%A | The distribution(position/length) of 25% A in AS 30bp + downstream30bp sequence |
| 1122 | as30down30distr50%A | The distribution(position/length) of 50% A in AS 30bp + downstream30bp sequence |
| 1123 | as30down30distr75%A | The distribution(position/length) of 75% A in AS 30bp + downstream30bp sequence |
| 1124 | as30down30distr100%A | The distribution(position/length) of 100% A in AS 30bp + downstream30bp sequence |
| 1125 | as30down30distr1%T | The distribution(position/length) of 1% T in AS 30bp + downstream30bp sequence |
| 1126 | as30down30distr25%T | The distribution(position/length) of 25% T in AS 30bp + downstream30bp sequence |
| 1127 | as30down30distr50%T | The distribution(position/length) of 50% T in AS 30bp + downstream30bp sequence |
| 1128 | as30down30distr75%T | The distribution(position/length) of 75% T in AS 30bp + downstream30bp sequence |
| 1129 | as30down30distr100%T | The distribution(position/length) of 100% T in AS 30bp + downstream30bp sequence |
| 1130 | as30down30distr1%C | The distribution(position/length) of 1% C in AS 30bp + downstream30bp sequence |
| 1131 | as30down30distr25%C | The distribution(position/length) of 25% C in AS 30bp + downstream30bp sequence |
| 1132 | as30down30distr50%C | The distribution(position/length) of 50% C in AS 30bp + downstream30bp sequence |
| 1133 | as30down30distr75%C | The distribution(position/length) of 75% C in AS 30bp + downstream30bp sequence |
| 1134 | as30down30distr100%C | The distribution(position/length) of 100% C in AS 30bp + downstream30bp sequence |
| 1135 | as30down30distr1%G | The distribution(position/length) of 1% G in AS 30bp + downstream30bp sequence |
| 1136 | as30down30distr25%G | The distribution(position/length) of 25% G in AS 30bp + downstream30bp sequence |
| 1137 | as30down30distr50%G | The distribution(position/length) of 50% G in AS 30bp + downstream30bp sequence |
| 1138 | as30down30distr75%G | The distribution(position/length) of 75% G in AS 30bp + downstream30bp sequence |
| 1139 | as30down30distr100%G | The distribution(position/length) of 100% G in AS 30bp + downstream30bp sequence |
| 1140 | as30down30donerGT | Is there GT in doner of AS 30bp + downstream30bp sequence (1 for yes, 0 for no) |
| 1141 | as30down30acceptorGT | Is there GT in acceptor of AS 30bp + downstream30bp sequence (1 for yes, 0 for no) |
| 1142 | as30down30donerGC | Is there GC in doner of AS 30bp + downstream30bp sequence (1 for yes, 0 for no) |
| 1143 | as30down30acceptorGC | Is there GC in acceptor of AS 30bp + downstream30bp sequence (1 for yes, 0 for no) |
| 1144 | as30down30donerAT | Is there AT in doner of AS 30bp + downstream30bp sequence (1 for yes, 0 for no) |
| 1145 | as30down30acceptorAT | Is there AT in acceptor of AS 30bp + downstream30bp sequence (1 for yes, 0 for no) |
| 1146 | as30down30donerAG | Is there AG in doner of AS 30bp + downstream30bp sequence (1 for yes, 0 for no) |
| 1147 | as30down30acceptorAG | Is there AG in acceptor of AS 30bp + downstream30bp sequence (1 for yes, 0 for no) |
| 1148 | as30down30donerAC | Is there AC in doner of AS 30bp + downstream30bp sequence (1 for yes, 0 for no) |
| 1149 | as30down30acceptorAC | Is there AC in acceptor of AS 30bp + downstream30bp sequence (1 for yes, 0 for no) |
| 1150 | up50as50if%3 | Whether the length of upstream50 + AS 50bp sequence divisible by three |
| 1151 | up50as50GC | The GC contant of upstream50 + AS 50bp sequence |
| 1152 | up50as50numberTAA | The number of stopdocon TAA in upstream50 + AS 50bp sequence |
| 1153 | up50as50numberTAG | The number of stopdocon TAA in upstream50 + AS 50bp sequence |
| 1154 | up50as50numberTGA | The number of stopdocon TAA in upstream50 + AS 50bp sequence |
| 1155 | up50as50frequencyA | The frequency of A in upstream50 + AS 50bp sequence |
| 1156 | up50as50frequencyAA | The frequency of AA in upstream50 + AS 50bp sequence |
| 1157 | up50as50frequencyAAA | The frequency of AAA in upstream50 + AS 50bp sequence |
| 1158 | up50as50frequencyAAT | The frequency of AAT in upstream50 + AS 50bp sequence |
| 1159 | up50as50frequencyAAC | The frequency of AAC in upstream50 + AS 50bp sequence |
| 1160 | up50as50frequencyAAG | The frequency of AAG in upstream50 + AS 50bp sequence |
| 1161 | up50as50frequencyAT | The frequency of AT in upstream50 + AS 50bp sequence |
| 1162 | up50as50frequencyATA | The frequency of ATA in upstream50 + AS 50bp sequence |
| 1163 | up50as50frequencyATT | The frequency of ATT in upstream50 + AS 50bp sequence |
| 1164 | up50as50frequencyATC | The frequency of ATC in upstream50 + AS 50bp sequence |
| 1165 | up50as50frequencyATG | The frequency of ATG in upstream50 + AS 50bp sequence |
| 1166 | up50as50frequencyAC | The frequency of AC in upstream50 + AS 50bp sequence |
| 1167 | up50as50frequencyACA | The frequency of ACA in upstream50 + AS 50bp sequence |
| 1168 | up50as50frequencyACT | The frequency of ACT in upstream50 + AS 50bp sequence |
| 1169 | up50as50frequencyACC | The frequency of ACC in upstream50 + AS 50bp sequence |
| 1170 | up50as50frequencyACG | The frequency of ACG in upstream50 + AS 50bp sequence |
| 1171 | up50as50frequencyAG | The frequency of AG in upstream50 + AS 50bp sequence |
| 1172 | up50as50frequencyAGA | The frequency of AGA in upstream50 + AS 50bp sequence |
| 1173 | up50as50frequencyAGT | The frequency of AGT in upstream50 + AS 50bp sequence |
| 1174 | up50as50frequencyAGC | The frequency of AGC in upstream50 + AS 50bp sequence |
| 1175 | up50as50frequencyAGG | The frequency of AGG in upstream50 + AS 50bp sequence |
| 1176 | up50as50frequencyT | The frequency of T in upstream50 + AS 50bp sequence |
| 1177 | up50as50frequencyTA | The frequency of TA in upstream50 + AS 50bp sequence |
| 1178 | up50as50frequencyTAA | The frequency of TAA in upstream50 + AS 50bp sequence |
| 1179 | up50as50frequencyTAT | The frequency of TAT in upstream50 + AS 50bp sequence |
| 1180 | up50as50frequencyTAC | The frequency of TAC in upstream50 + AS 50bp sequence |
| 1181 | up50as50frequencyTAG | The frequency of TAG in upstream50 + AS 50bp sequence |
| 1182 | up50as50frequencyTT | The frequency of TT in upstream50 + AS 50bp sequence |
| 1183 | up50as50frequencyTTA | The frequency of TTA in upstream50 + AS 50bp sequence |
| 1184 | up50as50frequencyTTT | The frequency of TTT in upstream50 + AS 50bp sequence |
| 1185 | up50as50frequencyTTC | The frequency of TTC in upstream50 + AS 50bp sequence |
| 1186 | up50as50frequencyTTG | The frequency of TTG in upstream50 + AS 50bp sequence |
| 1187 | up50as50frequencyTC | The frequency of TC in upstream50 + AS 50bp sequence |
| 1188 | up50as50frequencyTCA | The frequency of TCA in upstream50 + AS 50bp sequence |
| 1189 | up50as50frequencyTCT | The frequency of TCT in upstream50 + AS 50bp sequence |
| 1190 | up50as50frequencyTCC | The frequency of TCC in upstream50 + AS 50bp sequence |
| 1191 | up50as50frequencyTCG | The frequency of TCG in upstream50 + AS 50bp sequence |
| 1192 | up50as50frequencyTG | The frequency of TG in upstream50 + AS 50bp sequence |
| 1193 | up50as50frequencyTGA | The frequency of TGA in upstream50 + AS 50bp sequence |
| 1194 | up50as50frequencyTGT | The frequency of TGT in upstream50 + AS 50bp sequence |
| 1195 | up50as50frequencyTGC | The frequency of TGC in upstream50 + AS 50bp sequence |
| 1196 | up50as50frequencyTGG | The frequency of TGG in upstream50 + AS 50bp sequence |
| 1197 | up50as50frequencyC | The frequency of C in upstream50 + AS 50bp sequence |
| 1198 | up50as50frequencyCA | The frequency of CA in upstream50 + AS 50bp sequence |
| 1199 | up50as50frequencyCAA | The frequency of CAA in upstream50 + AS 50bp sequence |
| 1200 | up50as50frequencyCAT | The frequency of CAT in upstream50 + AS 50bp sequence |
| 1201 | up50as50frequencyCAC | The frequency of CAC in upstream50 + AS 50bp sequence |
| 1202 | up50as50frequencyCAG | The frequency of CAG in upstream50 + AS 50bp sequence |
| 1203 | up50as50frequencyCT | The frequency of CT in upstream50 + AS 50bp sequence |
| 1204 | up50as50frequencyCTA | The frequency of CTA in upstream50 + AS 50bp sequence |
| 1205 | up50as50frequencyCTT | The frequency of CTT in upstream50 + AS 50bp sequence |
| 1206 | up50as50frequencyCTC | The frequency of CTC in upstream50 + AS 50bp sequence |
| 1207 | up50as50frequencyCTG | The frequency of CTG in upstream50 + AS 50bp sequence |
| 1208 | up50as50frequencyCC | The frequency of CC in upstream50 + AS 50bp sequence |
| 1209 | up50as50frequencyCCA | The frequency of CCA in upstream50 + AS 50bp sequence |
| 1210 | up50as50frequencyCCT | The frequency of CCT in upstream50 + AS 50bp sequence |
| 1211 | up50as50frequencyCCC | The frequency of CCC in upstream50 + AS 50bp sequence |
| 1212 | up50as50frequencyCCG | The frequency of CCG in upstream50 + AS 50bp sequence |
| 1213 | up50as50frequencyCG | The frequency of CG in upstream50 + AS 50bp sequence |
| 1214 | up50as50frequencyCGA | The frequency of CGA in upstream50 + AS 50bp sequence |
| 1215 | up50as50frequencyCGT | The frequency of CGT in upstream50 + AS 50bp sequence |
| 1216 | up50as50frequencyCGC | The frequency of CGC in upstream50 + AS 50bp sequence |
| 1217 | up50as50frequencyCGG | The frequency of CGG in upstream50 + AS 50bp sequence |
| 1218 | up50as50frequencyG | The frequency of G in upstream50 + AS 50bp sequence |
| 1219 | up50as50frequencyGA | The frequency of GA in upstream50 + AS 50bp sequence |
| 1220 | up50as50frequencyGAA | The frequency of GAA in upstream50 + AS 50bp sequence |
| 1221 | up50as50frequencyGAT | The frequency of GAT in upstream50 + AS 50bp sequence |
| 1222 | up50as50frequencyGAC | The frequency of GAC in upstream50 + AS 50bp sequence |
| 1223 | up50as50frequencyGAG | The frequency of GAG in upstream50 + AS 50bp sequence |
| 1224 | up50as50frequencyGT | The frequency of GT in upstream50 + AS 50bp sequence |
| 1225 | up50as50frequencyGTA | The frequency of GTA in upstream50 + AS 50bp sequence |
| 1226 | up50as50frequencyGTT | The frequency of GTT in upstream50 + AS 50bp sequence |
| 1227 | up50as50frequencyGTC | The frequency of GTC in upstream50 + AS 50bp sequence |
| 1228 | up50as50frequencyGTG | The frequency of GTG in upstream50 + AS 50bp sequence |
| 1229 | up50as50frequencyGC | The frequency of GC in upstream50 + AS 50bp sequence |
| 1230 | up50as50frequencyGCA | The frequency of GCA in upstream50 + AS 50bp sequence |
| 1231 | up50as50frequencyGCT | The frequency of GCT in upstream50 + AS 50bp sequence |
| 1232 | up50as50frequencyGCC | The frequency of GCC in upstream50 + AS 50bp sequence |
| 1233 | up50as50frequencyGCG | The frequency of GCG in upstream50 + AS 50bp sequence |
| 1234 | up50as50frequencyGG | The frequency of GG in upstream50 + AS 50bp sequence |
| 1235 | up50as50frequencyGGA | The frequency of GGA in upstream50 + AS 50bp sequence |
| 1236 | up50as50frequencyGGT | The frequency of GGT in upstream50 + AS 50bp sequence |
| 1237 | up50as50frequencyGGC | The frequency of GGC in upstream50 + AS 50bp sequence |
| 1238 | up50as50frequencyGGG | The frequency of GGG in upstream50 + AS 50bp sequence |
| 1239 | up50as50distr1%A | The distribution(position/length) of 1% A in upstream50 + AS 50bp sequence |
| 1240 | up50as50distr25%A | The distribution(position/length) of 25% A in upstream50 + AS 50bp sequence |
| 1241 | up50as50distr50%A | The distribution(position/length) of 50% A in upstream50 + AS 50bp sequence |
| 1242 | up50as50distr75%A | The distribution(position/length) of 75% A in upstream50 + AS 50bp sequence |
| 1243 | up50as50distr100%A | The distribution(position/length) of 100% A in upstream50 + AS 50bp sequence |
| 1244 | up50as50distr1%T | The distribution(position/length) of 1% T in upstream50 + AS 50bp sequence |
| 1245 | up50as50distr25%T | The distribution(position/length) of 25% T in upstream50 + AS 50bp sequence |
| 1246 | up50as50distr50%T | The distribution(position/length) of 50% T in upstream50 + AS 50bp sequence |
| 1247 | up50as50distr75%T | The distribution(position/length) of 75% T in upstream50 + AS 50bp sequence |
| 1248 | up50as50distr100%T | The distribution(position/length) of 100% T in upstream50 + AS 50bp sequence |
| 1249 | up50as50distr1%C | The distribution(position/length) of 1% C in upstream50 + AS 50bp sequence |
| 1250 | up50as50distr25%C | The distribution(position/length) of 25% C in upstream50 + AS 50bp sequence |
| 1251 | up50as50distr50%C | The distribution(position/length) of 50% C in upstream50 + AS 50bp sequence |
| 1252 | up50as50distr75%C | The distribution(position/length) of 75% C in upstream50 + AS 50bp sequence |
| 1253 | up50as50distr100%C | The distribution(position/length) of 100% C in upstream50 + AS 50bp sequence |
| 1254 | up50as50distr1%G | The distribution(position/length) of 1% G in upstream50 + AS 50bp sequence |
| 1255 | up50as50distr25%G | The distribution(position/length) of 25% G in upstream50 + AS 50bp sequence |
| 1256 | up50as50distr50%G | The distribution(position/length) of 50% G in upstream50 + AS 50bp sequence |
| 1257 | up50as50distr75%G | The distribution(position/length) of 75% G in upstream50 + AS 50bp sequence |
| 1258 | up50as50distr100%G | The distribution(position/length) of 100% G in upstream50 + AS 50bp sequence |
| 1259 | up50as50donerGT | Is there GT in doner of upstream50 + AS 50bp sequence (1 for yes, 0 for no) |
| 1260 | up50as50acceptorGT | Is there GT in acceptor of upstream50 + AS 50bp sequence (1 for yes, 0 for no) |
| 1261 | up50as50donerGC | Is there GC in doner of upstream50 + AS 50bp sequence (1 for yes, 0 for no) |
| 1262 | up50as50acceptorGC | Is there GC in acceptor of upstream50 + AS 50bp sequence (1 for yes, 0 for no) |
| 1263 | up50as50donerAT | Is there AT in doner of upstream50 + AS 50bp sequence (1 for yes, 0 for no) |
| 1264 | up50as50acceptorAT | Is there AT in acceptor of upstream50 + AS 50bp sequence (1 for yes, 0 for no) |
| 1265 | up50as50donerAG | Is there AG in doner of upstream50 + AS 50bp sequence (1 for yes, 0 for no) |
| 1266 | up50as50acceptorAG | Is there AG in acceptor of upstream50 + AS 50bp sequence (1 for yes, 0 for no) |
| 1267 | up50as50donerAC | Is there AC in doner of upstream50 + AS 50bp sequence (1 for yes, 0 for no) |
| 1268 | up50as50acceptorAC | Is there AC in acceptor of upstream50 + AS 50bp sequence (1 for yes, 0 for no) |
| 1269 | up50down50if%3 | Whether the length of upstream50 + downstream50 sequence divisible by three |
| 1270 | up50down50GC | The GC contant of upstream50 + downstream50 sequence |
| 1271 | up50down50numberTAA | The number of stopdocon TAA in upstream50 + downstream50 sequence |
| 1272 | up50down50numberTAG | The number of stopdocon TAA in upstream50 + downstream50 sequence |
| 1273 | up50down50numberTGA | The number of stopdocon TAA in upstream50 + downstream50 sequence |
| 1274 | up50down50frequencyA | The frequency of A in upstream50 + downstream50 sequence |
| 1275 | up50down50frequencyAA | The frequency of AA in upstream50 + downstream50 sequence |
| 1276 | up50down50frequencyAAA | The frequency of AAA in upstream50 + downstream50 sequence |
| 1277 | up50down50frequencyAAT | The frequency of AAT in upstream50 + downstream50 sequence |
| 1278 | up50down50frequencyAAC | The frequency of AAC in upstream50 + downstream50 sequence |
| 1279 | up50down50frequencyAAG | The frequency of AAG in upstream50 + downstream50 sequence |
| 1280 | up50down50frequencyAT | The frequency of AT in upstream50 + downstream50 sequence |
| 1281 | up50down50frequencyATA | The frequency of ATA in upstream50 + downstream50 sequence |
| 1282 | up50down50frequencyATT | The frequency of ATT in upstream50 + downstream50 sequence |
| 1283 | up50down50frequencyATC | The frequency of ATC in upstream50 + downstream50 sequence |
| 1284 | up50down50frequencyATG | The frequency of ATG in upstream50 + downstream50 sequence |
| 1285 | up50down50frequencyAC | The frequency of AC in upstream50 + downstream50 sequence |
| 1286 | up50down50frequencyACA | The frequency of ACA in upstream50 + downstream50 sequence |
| 1287 | up50down50frequencyACT | The frequency of ACT in upstream50 + downstream50 sequence |
| 1288 | up50down50frequencyACC | The frequency of ACC in upstream50 + downstream50 sequence |
| 1289 | up50down50frequencyACG | The frequency of ACG in upstream50 + downstream50 sequence |
| 1290 | up50down50frequencyAG | The frequency of AG in upstream50 + downstream50 sequence |
| 1291 | up50down50frequencyAGA | The frequency of AGA in upstream50 + downstream50 sequence |
| 1292 | up50down50frequencyAGT | The frequency of AGT in upstream50 + downstream50 sequence |
| 1293 | up50down50frequencyAGC | The frequency of AGC in upstream50 + downstream50 sequence |
| 1294 | up50down50frequencyAGG | The frequency of AGG in upstream50 + downstream50 sequence |
| 1295 | up50down50frequencyT | The frequency of T in upstream50 + downstream50 sequence |
| 1296 | up50down50frequencyTA | The frequency of TA in upstream50 + downstream50 sequence |
| 1297 | up50down50frequencyTAA | The frequency of TAA in upstream50 + downstream50 sequence |
| 1298 | up50down50frequencyTAT | The frequency of TAT in upstream50 + downstream50 sequence |
| 1299 | up50down50frequencyTAC | The frequency of TAC in upstream50 + downstream50 sequence |
| 1300 | up50down50frequencyTAG | The frequency of TAG in upstream50 + downstream50 sequence |
| 1301 | up50down50frequencyTT | The frequency of TT in upstream50 + downstream50 sequence |
| 1302 | up50down50frequencyTTA | The frequency of TTA in upstream50 + downstream50 sequence |
| 1303 | up50down50frequencyTTT | The frequency of TTT in upstream50 + downstream50 sequence |
| 1304 | up50down50frequencyTTC | The frequency of TTC in upstream50 + downstream50 sequence |
| 1305 | up50down50frequencyTTG | The frequency of TTG in upstream50 + downstream50 sequence |
| 1306 | up50down50frequencyTC | The frequency of TC in upstream50 + downstream50 sequence |
| 1307 | up50down50frequencyTCA | The frequency of TCA in upstream50 + downstream50 sequence |
| 1308 | up50down50frequencyTCT | The frequency of TCT in upstream50 + downstream50 sequence |
| 1309 | up50down50frequencyTCC | The frequency of TCC in upstream50 + downstream50 sequence |
| 1310 | up50down50frequencyTCG | The frequency of TCG in upstream50 + downstream50 sequence |
| 1311 | up50down50frequencyTG | The frequency of TG in upstream50 + downstream50 sequence |
| 1312 | up50down50frequencyTGA | The frequency of TGA in upstream50 + downstream50 sequence |
| 1313 | up50down50frequencyTGT | The frequency of TGT in upstream50 + downstream50 sequence |
| 1314 | up50down50frequencyTGC | The frequency of TGC in upstream50 + downstream50 sequence |
| 1315 | up50down50frequencyTGG | The frequency of TGG in upstream50 + downstream50 sequence |
| 1316 | up50down50frequencyC | The frequency of C in upstream50 + downstream50 sequence |
| 1317 | up50down50frequencyCA | The frequency of CA in upstream50 + downstream50 sequence |
| 1318 | up50down50frequencyCAA | The frequency of CAA in upstream50 + downstream50 sequence |
| 1319 | up50down50frequencyCAT | The frequency of CAT in upstream50 + downstream50 sequence |
| 1320 | up50down50frequencyCAC | The frequency of CAC in upstream50 + downstream50 sequence |
| 1321 | up50down50frequencyCAG | The frequency of CAG in upstream50 + downstream50 sequence |
| 1322 | up50down50frequencyCT | The frequency of CT in upstream50 + downstream50 sequence |
| 1323 | up50down50frequencyCTA | The frequency of CTA in upstream50 + downstream50 sequence |
| 1324 | up50down50frequencyCTT | The frequency of CTT in upstream50 + downstream50 sequence |
| 1325 | up50down50frequencyCTC | The frequency of CTC in upstream50 + downstream50 sequence |
| 1326 | up50down50frequencyCTG | The frequency of CTG in upstream50 + downstream50 sequence |
| 1327 | up50down50frequencyCC | The frequency of CC in upstream50 + downstream50 sequence |
| 1328 | up50down50frequencyCCA | The frequency of CCA in upstream50 + downstream50 sequence |
| 1329 | up50down50frequencyCCT | The frequency of CCT in upstream50 + downstream50 sequence |
| 1330 | up50down50frequencyCCC | The frequency of CCC in upstream50 + downstream50 sequence |
| 1331 | up50down50frequencyCCG | The frequency of CCG in upstream50 + downstream50 sequence |
| 1332 | up50down50frequencyCG | The frequency of CG in upstream50 + downstream50 sequence |
| 1333 | up50down50frequencyCGA | The frequency of CGA in upstream50 + downstream50 sequence |
| 1334 | up50down50frequencyCGT | The frequency of CGT in upstream50 + downstream50 sequence |
| 1335 | up50down50frequencyCGC | The frequency of CGC in upstream50 + downstream50 sequence |
| 1336 | up50down50frequencyCGG | The frequency of CGG in upstream50 + downstream50 sequence |
| 1337 | up50down50frequencyG | The frequency of G in upstream50 + downstream50 sequence |
| 1338 | up50down50frequencyGA | The frequency of GA in upstream50 + downstream50 sequence |
| 1339 | up50down50frequencyGAA | The frequency of GAA in upstream50 + downstream50 sequence |
| 1340 | up50down50frequencyGAT | The frequency of GAT in upstream50 + downstream50 sequence |
| 1341 | up50down50frequencyGAC | The frequency of GAC in upstream50 + downstream50 sequence |
| 1342 | up50down50frequencyGAG | The frequency of GAG in upstream50 + downstream50 sequence |
| 1343 | up50down50frequencyGT | The frequency of GT in upstream50 + downstream50 sequence |
| 1344 | up50down50frequencyGTA | The frequency of GTA in upstream50 + downstream50 sequence |
| 1345 | up50down50frequencyGTT | The frequency of GTT in upstream50 + downstream50 sequence |
| 1346 | up50down50frequencyGTC | The frequency of GTC in upstream50 + downstream50 sequence |
| 1347 | up50down50frequencyGTG | The frequency of GTG in upstream50 + downstream50 sequence |
| 1348 | up50down50frequencyGC | The frequency of GC in upstream50 + downstream50 sequence |
| 1349 | up50down50frequencyGCA | The frequency of GCA in upstream50 + downstream50 sequence |
| 1350 | up50down50frequencyGCT | The frequency of GCT in upstream50 + downstream50 sequence |
| 1351 | up50down50frequencyGCC | The frequency of GCC in upstream50 + downstream50 sequence |
| 1352 | up50down50frequencyGCG | The frequency of GCG in upstream50 + downstream50 sequence |
| 1353 | up50down50frequencyGG | The frequency of GG in upstream50 + downstream50 sequence |
| 1354 | up50down50frequencyGGA | The frequency of GGA in upstream50 + downstream50 sequence |
| 1355 | up50down50frequencyGGT | The frequency of GGT in upstream50 + downstream50 sequence |
| 1356 | up50down50frequencyGGC | The frequency of GGC in upstream50 + downstream50 sequence |
| 1357 | up50down50frequencyGGG | The frequency of GGG in upstream50 + downstream50 sequence |
| 1358 | up50down50distr1%A | The distribution(position/length) of 1% A in upstream50 + downstream50 sequence |
| 1359 | up50down50distr25%A | The distribution(position/length) of 25% A in upstream50 + downstream50 sequence |
| 1360 | up50down50distr50%A | The distribution(position/length) of 50% A in upstream50 + downstream50 sequence |
| 1361 | up50down50distr75%A | The distribution(position/length) of 75% A in upstream50 + downstream50 sequence |
| 1362 | up50down50distr100%A | The distribution(position/length) of 100% A in upstream50 + downstream50 sequence |
| 1363 | up50down50distr1%T | The distribution(position/length) of 1% T in upstream50 + downstream50 sequence |
| 1364 | up50down50distr25%T | The distribution(position/length) of 25% T in upstream50 + downstream50 sequence |
| 1365 | up50down50distr50%T | The distribution(position/length) of 50% T in upstream50 + downstream50 sequence |
| 1366 | up50down50distr75%T | The distribution(position/length) of 75% T in upstream50 + downstream50 sequence |
| 1367 | up50down50distr100%T | The distribution(position/length) of 100% T in upstream50 + downstream50 sequence |
| 1368 | up50down50distr1%C | The distribution(position/length) of 1% C in upstream50 + downstream50 sequence |
| 1369 | up50down50distr25%C | The distribution(position/length) of 25% C in upstream50 + downstream50 sequence |
| 1370 | up50down50distr50%C | The distribution(position/length) of 50% C in upstream50 + downstream50 sequence |
| 1371 | up50down50distr75%C | The distribution(position/length) of 75% C in upstream50 + downstream50 sequence |
| 1372 | up50down50distr100%C | The distribution(position/length) of 100% C in upstream50 + downstream50 sequence |
| 1373 | up50down50distr1%G | The distribution(position/length) of 1% G in upstream50 + downstream50 sequence |
| 1374 | up50down50distr25%G | The distribution(position/length) of 25% G in upstream50 + downstream50 sequence |
| 1375 | up50down50distr50%G | The distribution(position/length) of 50% G in upstream50 + downstream50 sequence |
| 1376 | up50down50distr75%G | The distribution(position/length) of 75% G in upstream50 + downstream50 sequence |
| 1377 | up50down50distr100%G | The distribution(position/length) of 100% G in upstream50 + downstream50 sequence |
| 1378 | up50down50donerGT | Is there GT in doner of upstream50 + downstream50 sequence (1 for yes, 0 for no) |
| 1379 | up50down50acceptorGT | Is there GT in acceptor of upstream50 + downstream50 sequence (1 for yes, 0 for no) |
| 1380 | up50down50donerGC | Is there GC in doner of upstream50 + downstream50 sequence (1 for yes, 0 for no) |
| 1381 | up50down50acceptorGC | Is there GC in acceptor of upstream50 + downstream50 sequence (1 for yes, 0 for no) |
| 1382 | up50down50donerAT | Is there AT in doner of upstream50 + downstream50 sequence (1 for yes, 0 for no) |
| 1383 | up50down50acceptorAT | Is there AT in acceptor of upstream50 + downstream50 sequence (1 for yes, 0 for no) |
| 1384 | up50down50donerAG | Is there AG in doner of upstream50 + downstream50 sequence (1 for yes, 0 for no) |
| 1385 | up50down50acceptorAG | Is there AG in acceptor of upstream50 + downstream50 sequence (1 for yes, 0 for no) |
| 1386 | up50down50donerAC | Is there AC in doner of upstream50 + downstream50 sequence (1 for yes, 0 for no) |
| 1387 | up50down50acceptorAC | Is there AC in acceptor of upstream50 + downstream50 sequence (1 for yes, 0 for no) |
| 1388 | as50down50if%3 | Whether the length of AS 50bp + downstream50bp sequence divisible by three |
| 1389 | as50down50GC | The GC contant of AS 50bp + downstream50bp sequence |
| 1390 | as50down50numberTAA | The number of stopdocon TAA in AS 50bp + downstream50bp sequence |
| 1391 | as50down50numberTAG | The number of stopdocon TAA in AS 50bp + downstream50bp sequence |
| 1392 | as50down50numberTGA | The number of stopdocon TAA in AS 50bp + downstream50bp sequence |
| 1393 | as50down50frequencyA | The frequency of A in AS 50bp + downstream50bp sequence |
| 1394 | as50down50frequencyAA | The frequency of AA in AS 50bp + downstream50bp sequence |
| 1395 | as50down50frequencyAAA | The frequency of AAA in AS 50bp + downstream50bp sequence |
| 1396 | as50down50frequencyAAT | The frequency of AAT in AS 50bp + downstream50bp sequence |
| 1397 | as50down50frequencyAAC | The frequency of AAC in AS 50bp + downstream50bp sequence |
| 1398 | as50down50frequencyAAG | The frequency of AAG in AS 50bp + downstream50bp sequence |
| 1399 | as50down50frequencyAT | The frequency of AT in AS 50bp + downstream50bp sequence |
| 1400 | as50down50frequencyATA | The frequency of ATA in AS 50bp + downstream50bp sequence |
| 1401 | as50down50frequencyATT | The frequency of ATT in AS 50bp + downstream50bp sequence |
| 1402 | as50down50frequencyATC | The frequency of ATC in AS 50bp + downstream50bp sequence |
| 1403 | as50down50frequencyATG | The frequency of ATG in AS 50bp + downstream50bp sequence |
| 1404 | as50down50frequencyAC | The frequency of AC in AS 50bp + downstream50bp sequence |
| 1405 | as50down50frequencyACA | The frequency of ACA in AS 50bp + downstream50bp sequence |
| 1406 | as50down50frequencyACT | The frequency of ACT in AS 50bp + downstream50bp sequence |
| 1407 | as50down50frequencyACC | The frequency of ACC in AS 50bp + downstream50bp sequence |
| 1408 | as50down50frequencyACG | The frequency of ACG in AS 50bp + downstream50bp sequence |
| 1409 | as50down50frequencyAG | The frequency of AG in AS 50bp + downstream50bp sequence |
| 1410 | as50down50frequencyAGA | The frequency of AGA in AS 50bp + downstream50bp sequence |
| 1411 | as50down50frequencyAGT | The frequency of AGT in AS 50bp + downstream50bp sequence |
| 1412 | as50down50frequencyAGC | The frequency of AGC in AS 50bp + downstream50bp sequence |
| 1413 | as50down50frequencyAGG | The frequency of AGG in AS 50bp + downstream50bp sequence |
| 1414 | as50down50frequencyT | The frequency of T in AS 50bp + downstream50bp sequence |
| 1415 | as50down50frequencyTA | The frequency of TA in AS 50bp + downstream50bp sequence |
| 1416 | as50down50frequencyTAA | The frequency of TAA in AS 50bp + downstream50bp sequence |
| 1417 | as50down50frequencyTAT | The frequency of TAT in AS 50bp + downstream50bp sequence |
| 1418 | as50down50frequencyTAC | The frequency of TAC in AS 50bp + downstream50bp sequence |
| 1419 | as50down50frequencyTAG | The frequency of TAG in AS 50bp + downstream50bp sequence |
| 1420 | as50down50frequencyTT | The frequency of TT in AS 50bp + downstream50bp sequence |
| 1421 | as50down50frequencyTTA | The frequency of TTA in AS 50bp + downstream50bp sequence |
| 1422 | as50down50frequencyTTT | The frequency of TTT in AS 50bp + downstream50bp sequence |
| 1423 | as50down50frequencyTTC | The frequency of TTC in AS 50bp + downstream50bp sequence |
| 1424 | as50down50frequencyTTG | The frequency of TTG in AS 50bp + downstream50bp sequence |
| 1425 | as50down50frequencyTC | The frequency of TC in AS 50bp + downstream50bp sequence |
| 1426 | as50down50frequencyTCA | The frequency of TCA in AS 50bp + downstream50bp sequence |
| 1427 | as50down50frequencyTCT | The frequency of TCT in AS 50bp + downstream50bp sequence |
| 1428 | as50down50frequencyTCC | The frequency of TCC in AS 50bp + downstream50bp sequence |
| 1429 | as50down50frequencyTCG | The frequency of TCG in AS 50bp + downstream50bp sequence |
| 1430 | as50down50frequencyTG | The frequency of TG in AS 50bp + downstream50bp sequence |
| 1431 | as50down50frequencyTGA | The frequency of TGA in AS 50bp + downstream50bp sequence |
| 1432 | as50down50frequencyTGT | The frequency of TGT in AS 50bp + downstream50bp sequence |
| 1433 | as50down50frequencyTGC | The frequency of TGC in AS 50bp + downstream50bp sequence |
| 1434 | as50down50frequencyTGG | The frequency of TGG in AS 50bp + downstream50bp sequence |
| 1435 | as50down50frequencyC | The frequency of C in AS 50bp + downstream50bp sequence |
| 1436 | as50down50frequencyCA | The frequency of CA in AS 50bp + downstream50bp sequence |
| 1437 | as50down50frequencyCAA | The frequency of CAA in AS 50bp + downstream50bp sequence |
| 1438 | as50down50frequencyCAT | The frequency of CAT in AS 50bp + downstream50bp sequence |
| 1439 | as50down50frequencyCAC | The frequency of CAC in AS 50bp + downstream50bp sequence |
| 1440 | as50down50frequencyCAG | The frequency of CAG in AS 50bp + downstream50bp sequence |
| 1441 | as50down50frequencyCT | The frequency of CT in AS 50bp + downstream50bp sequence |
| 1442 | as50down50frequencyCTA | The frequency of CTA in AS 50bp + downstream50bp sequence |
| 1443 | as50down50frequencyCTT | The frequency of CTT in AS 50bp + downstream50bp sequence |
| 1444 | as50down50frequencyCTC | The frequency of CTC in AS 50bp + downstream50bp sequence |
| 1445 | as50down50frequencyCTG | The frequency of CTG in AS 50bp + downstream50bp sequence |
| 1446 | as50down50frequencyCC | The frequency of CC in AS 50bp + downstream50bp sequence |
| 1447 | as50down50frequencyCCA | The frequency of CCA in AS 50bp + downstream50bp sequence |
| 1448 | as50down50frequencyCCT | The frequency of CCT in AS 50bp + downstream50bp sequence |
| 1449 | as50down50frequencyCCC | The frequency of CCC in AS 50bp + downstream50bp sequence |
| 1450 | as50down50frequencyCCG | The frequency of CCG in AS 50bp + downstream50bp sequence |
| 1451 | as50down50frequencyCG | The frequency of CG in AS 50bp + downstream50bp sequence |
| 1452 | as50down50frequencyCGA | The frequency of CGA in AS 50bp + downstream50bp sequence |
| 1453 | as50down50frequencyCGT | The frequency of CGT in AS 50bp + downstream50bp sequence |
| 1454 | as50down50frequencyCGC | The frequency of CGC in AS 50bp + downstream50bp sequence |
| 1455 | as50down50frequencyCGG | The frequency of CGG in AS 50bp + downstream50bp sequence |
| 1456 | as50down50frequencyG | The frequency of G in AS 50bp + downstream50bp sequence |
| 1457 | as50down50frequencyGA | The frequency of GA in AS 50bp + downstream50bp sequence |
| 1458 | as50down50frequencyGAA | The frequency of GAA in AS 50bp + downstream50bp sequence |
| 1459 | as50down50frequencyGAT | The frequency of GAT in AS 50bp + downstream50bp sequence |
| 1460 | as50down50frequencyGAC | The frequency of GAC in AS 50bp + downstream50bp sequence |
| 1461 | as50down50frequencyGAG | The frequency of GAG in AS 50bp + downstream50bp sequence |
| 1462 | as50down50frequencyGT | The frequency of GT in AS 50bp + downstream50bp sequence |
| 1463 | as50down50frequencyGTA | The frequency of GTA in AS 50bp + downstream50bp sequence |
| 1464 | as50down50frequencyGTT | The frequency of GTT in AS 50bp + downstream50bp sequence |
| 1465 | as50down50frequencyGTC | The frequency of GTC in AS 50bp + downstream50bp sequence |
| 1466 | as50down50frequencyGTG | The frequency of GTG in AS 50bp + downstream50bp sequence |
| 1467 | as50down50frequencyGC | The frequency of GC in AS 50bp + downstream50bp sequence |
| 1468 | as50down50frequencyGCA | The frequency of GCA in AS 50bp + downstream50bp sequence |
| 1469 | as50down50frequencyGCT | The frequency of GCT in AS 50bp + downstream50bp sequence |
| 1470 | as50down50frequencyGCC | The frequency of GCC in AS 50bp + downstream50bp sequence |
| 1471 | as50down50frequencyGCG | The frequency of GCG in AS 50bp + downstream50bp sequence |
| 1472 | as50down50frequencyGG | The frequency of GG in AS 50bp + downstream50bp sequence |
| 1473 | as50down50frequencyGGA | The frequency of GGA in AS 50bp + downstream50bp sequence |
| 1474 | as50down50frequencyGGT | The frequency of GGT in AS 50bp + downstream50bp sequence |
| 1475 | as50down50frequencyGGC | The frequency of GGC in AS 50bp + downstream50bp sequence |
| 1476 | as50down50frequencyGGG | The frequency of GGG in AS 50bp + downstream50bp sequence |
| 1477 | as50down50distr1%A | The distribution(position/length) of 1% A in AS 50bp + downstream50bp sequence |
| 1478 | as50down50distr25%A | The distribution(position/length) of 25% A in AS 50bp + downstream50bp sequence |
| 1479 | as50down50distr50%A | The distribution(position/length) of 50% A in AS 50bp + downstream50bp sequence |
| 1480 | as50down50distr75%A | The distribution(position/length) of 75% A in AS 50bp + downstream50bp sequence |
| 1481 | as50down50distr100%A | The distribution(position/length) of 100% A in AS 50bp + downstream50bp sequence |
| 1482 | as50down50distr1%T | The distribution(position/length) of 1% T in AS 50bp + downstream50bp sequence |
| 1483 | as50down50distr25%T | The distribution(position/length) of 25% T in AS 50bp + downstream50bp sequence |
| 1484 | as50down50distr50%T | The distribution(position/length) of 50% T in AS 50bp + downstream50bp sequence |
| 1485 | as50down50distr75%T | The distribution(position/length) of 75% T in AS 50bp + downstream50bp sequence |
| 1486 | as50down50distr100%T | The distribution(position/length) of 100% T in AS 50bp + downstream50bp sequence |
| 1487 | as50down50distr1%C | The distribution(position/length) of 1% C in AS 50bp + downstream50bp sequence |
| 1488 | as50down50distr25%C | The distribution(position/length) of 25% C in AS 50bp + downstream50bp sequence |
| 1489 | as50down50distr50%C | The distribution(position/length) of 50% C in AS 50bp + downstream50bp sequence |
| 1490 | as50down50distr75%C | The distribution(position/length) of 75% C in AS 50bp + downstream50bp sequence |
| 1491 | as50down50distr100%C | The distribution(position/length) of 100% C in AS 50bp + downstream50bp sequence |
| 1492 | as50down50distr1%G | The distribution(position/length) of 1% G in AS 50bp + downstream50bp sequence |
| 1493 | as50down50distr25%G | The distribution(position/length) of 25% G in AS 50bp + downstream50bp sequence |
| 1494 | as50down50distr50%G | The distribution(position/length) of 50% G in AS 50bp + downstream50bp sequence |
| 1495 | as50down50distr75%G | The distribution(position/length) of 75% G in AS 50bp + downstream50bp sequence |
| 1496 | as50down50distr100%G | The distribution(position/length) of 100% G in AS 50bp + downstream50bp sequence |
| 1497 | as50down50donerGT | Is there GT in doner of AS 50bp + downstream50bp sequence (1 for yes, 0 for no) |
| 1498 | as50down50acceptorGT | Is there GT in acceptor of AS 50bp + downstream50bp sequence (1 for yes, 0 for no) |
| 1499 | as50down50donerGC | Is there GC in doner of AS 50bp + downstream50bp sequence (1 for yes, 0 for no) |
| 1500 | as50down50acceptorGC | Is there GC in acceptor of AS 50bp + downstream50bp sequence (1 for yes, 0 for no) |
| 1501 | as50down50donerAT | Is there AT in doner of AS 50bp + downstream50bp sequence (1 for yes, 0 for no) |
| 1502 | as50down50acceptorAT | Is there AT in acceptor of AS 50bp + downstream50bp sequence (1 for yes, 0 for no) |
| 1503 | as50down50donerAG | Is there AG in doner of AS 50bp + downstream50bp sequence (1 for yes, 0 for no) |
| 1504 | as50down50acceptorAG | Is there AG in acceptor of AS 50bp + downstream50bp sequence (1 for yes, 0 for no) |
| 1505 | as50down50donerAC | Is there AC in doner of AS 50bp + downstream50bp sequence (1 for yes, 0 for no) |
| 1506 | as50down50acceptorAC | Is there AC in acceptor of AS 50bp + downstream50bp sequence (1 for yes, 0 for no) |
| 1507 | up10bpif%3 | Whether the length of ASASupstream 10bp sequence divisible by three |
| 1508 | up10bpGC | The GC contant of ASupstream 10bp sequence |
| 1509 | up10bpnumberTAA | The number of stopdocon TAA in ASupstream 10bp sequence |
| 1510 | up10bpnumberTAG | The number of stopdocon TAA in ASupstream 10bp sequence |
| 1511 | up10bpnumberTGA | The number of stopdocon TAA in ASupstream 10bp sequence |
| 1512 | up10bpfrequencyA | The frequency of A in ASupstream 10bp sequence |
| 1513 | up10bpfrequencyAA | The frequency of AA in ASupstream 10bp sequence |
| 1514 | up10bpfrequencyAAA | The frequency of AAA in ASupstream 10bp sequence |
| 1515 | up10bpfrequencyAAT | The frequency of AAT in ASupstream 10bp sequence |
| 1516 | up10bpfrequencyAAC | The frequency of AAC in ASupstream 10bp sequence |
| 1517 | up10bpfrequencyAAG | The frequency of AAG in ASupstream 10bp sequence |
| 1518 | up10bpfrequencyAT | The frequency of AT in ASupstream 10bp sequence |
| 1519 | up10bpfrequencyATA | The frequency of ATA in ASupstream 10bp sequence |
| 1520 | up10bpfrequencyATT | The frequency of ATT in ASupstream 10bp sequence |
| 1521 | up10bpfrequencyATC | The frequency of ATC in ASupstream 10bp sequence |
| 1522 | up10bpfrequencyATG | The frequency of ATG in ASupstream 10bp sequence |
| 1523 | up10bpfrequencyAC | The frequency of AC in ASupstream 10bp sequence |
| 1524 | up10bpfrequencyACA | The frequency of ACA in ASupstream 10bp sequence |
| 1525 | up10bpfrequencyACT | The frequency of ACT in ASupstream 10bp sequence |
| 1526 | up10bpfrequencyACC | The frequency of ACC in ASupstream 10bp sequence |
| 1527 | up10bpfrequencyACG | The frequency of ACG in ASupstream 10bp sequence |
| 1528 | up10bpfrequencyAG | The frequency of AG in ASupstream 10bp sequence |
| 1529 | up10bpfrequencyAGA | The frequency of AGA in ASupstream 10bp sequence |
| 1530 | up10bpfrequencyAGT | The frequency of AGT in ASupstream 10bp sequence |
| 1531 | up10bpfrequencyAGC | The frequency of AGC in ASupstream 10bp sequence |
| 1532 | up10bpfrequencyAGG | The frequency of AGG in ASupstream 10bp sequence |
| 1533 | up10bpfrequencyT | The frequency of T in ASupstream 10bp sequence |
| 1534 | up10bpfrequencyTA | The frequency of TA in ASupstream 10bp sequence |
| 1535 | up10bpfrequencyTAA | The frequency of TAA in ASupstream 10bp sequence |
| 1536 | up10bpfrequencyTAT | The frequency of TAT in ASupstream 10bp sequence |
| 1537 | up10bpfrequencyTAC | The frequency of TAC in ASupstream 10bp sequence |
| 1538 | up10bpfrequencyTAG | The frequency of TAG in ASupstream 10bp sequence |
| 1539 | up10bpfrequencyTT | The frequency of TT in ASupstream 10bp sequence |
| 1540 | up10bpfrequencyTTA | The frequency of TTA in ASupstream 10bp sequence |
| 1541 | up10bpfrequencyTTT | The frequency of TTT in ASupstream 10bp sequence |
| 1542 | up10bpfrequencyTTC | The frequency of TTC in ASupstream 10bp sequence |
| 1543 | up10bpfrequencyTTG | The frequency of TTG in ASupstream 10bp sequence |
| 1544 | up10bpfrequencyTC | The frequency of TC in ASupstream 10bp sequence |
| 1545 | up10bpfrequencyTCA | The frequency of TCA in ASupstream 10bp sequence |
| 1546 | up10bpfrequencyTCT | The frequency of TCT in ASupstream 10bp sequence |
| 1547 | up10bpfrequencyTCC | The frequency of TCC in ASupstream 10bp sequence |
| 1548 | up10bpfrequencyTCG | The frequency of TCG in ASupstream 10bp sequence |
| 1549 | up10bpfrequencyTG | The frequency of TG in ASupstream 10bp sequence |
| 1550 | up10bpfrequencyTGA | The frequency of TGA in ASupstream 10bp sequence |
| 1551 | up10bpfrequencyTGT | The frequency of TGT in ASupstream 10bp sequence |
| 1552 | up10bpfrequencyTGC | The frequency of TGC in ASupstream 10bp sequence |
| 1553 | up10bpfrequencyTGG | The frequency of TGG in ASupstream 10bp sequence |
| 1554 | up10bpfrequencyC | The frequency of C in ASupstream 10bp sequence |
| 1555 | up10bpfrequencyCA | The frequency of CA in ASupstream 10bp sequence |
| 1556 | up10bpfrequencyCAA | The frequency of CAA in ASupstream 10bp sequence |
| 1557 | up10bpfrequencyCAT | The frequency of CAT in ASupstream 10bp sequence |
| 1558 | up10bpfrequencyCAC | The frequency of CAC in ASupstream 10bp sequence |
| 1559 | up10bpfrequencyCAG | The frequency of CAG in ASupstream 10bp sequence |
| 1560 | up10bpfrequencyCT | The frequency of CT in ASupstream 10bp sequence |
| 1561 | up10bpfrequencyCTA | The frequency of CTA in ASupstream 10bp sequence |
| 1562 | up10bpfrequencyCTT | The frequency of CTT in ASupstream 10bp sequence |
| 1563 | up10bpfrequencyCTC | The frequency of CTC in ASupstream 10bp sequence |
| 1564 | up10bpfrequencyCTG | The frequency of CTG in ASupstream 10bp sequence |
| 1565 | up10bpfrequencyCC | The frequency of CC in ASupstream 10bp sequence |
| 1566 | up10bpfrequencyCCA | The frequency of CCA in ASupstream 10bp sequence |
| 1567 | up10bpfrequencyCCT | The frequency of CCT in ASupstream 10bp sequence |
| 1568 | up10bpfrequencyCCC | The frequency of CCC in ASupstream 10bp sequence |
| 1569 | up10bpfrequencyCCG | The frequency of CCG in ASupstream 10bp sequence |
| 1570 | up10bpfrequencyCG | The frequency of CG in ASupstream 10bp sequence |
| 1571 | up10bpfrequencyCGA | The frequency of CGA in ASupstream 10bp sequence |
| 1572 | up10bpfrequencyCGT | The frequency of CGT in ASupstream 10bp sequence |
| 1573 | up10bpfrequencyCGC | The frequency of CGC in ASupstream 10bp sequence |
| 1574 | up10bpfrequencyCGG | The frequency of CGG in ASupstream 10bp sequence |
| 1575 | up10bpfrequencyG | The frequency of G in ASupstream 10bp sequence |
| 1576 | up10bpfrequencyGA | The frequency of GA in ASupstream 10bp sequence |
| 1577 | up10bpfrequencyGAA | The frequency of GAA in ASupstream 10bp sequence |
| 1578 | up10bpfrequencyGAT | The frequency of GAT in ASupstream 10bp sequence |
| 1579 | up10bpfrequencyGAC | The frequency of GAC in ASupstream 10bp sequence |
| 1580 | up10bpfrequencyGAG | The frequency of GAG in ASupstream 10bp sequence |
| 1581 | up10bpfrequencyGT | The frequency of GT in ASupstream 10bp sequence |
| 1582 | up10bpfrequencyGTA | The frequency of GTA in ASupstream 10bp sequence |
| 1583 | up10bpfrequencyGTT | The frequency of GTT in ASupstream 10bp sequence |
| 1584 | up10bpfrequencyGTC | The frequency of GTC in ASupstream 10bp sequence |
| 1585 | up10bpfrequencyGTG | The frequency of GTG in ASupstream 10bp sequence |
| 1586 | up10bpfrequencyGC | The frequency of GC in ASupstream 10bp sequence |
| 1587 | up10bpfrequencyGCA | The frequency of GCA in ASupstream 10bp sequence |
| 1588 | up10bpfrequencyGCT | The frequency of GCT in ASupstream 10bp sequence |
| 1589 | up10bpfrequencyGCC | The frequency of GCC in ASupstream 10bp sequence |
| 1590 | up10bpfrequencyGCG | The frequency of GCG in ASupstream 10bp sequence |
| 1591 | up10bpfrequencyGG | The frequency of GG in ASupstream 10bp sequence |
| 1592 | up10bpfrequencyGGA | The frequency of GGA in ASupstream 10bp sequence |
| 1593 | up10bpfrequencyGGT | The frequency of GGT in ASupstream 10bp sequence |
| 1594 | up10bpfrequencyGGC | The frequency of GGC in ASupstream 10bp sequence |
| 1595 | up10bpfrequencyGGG | The frequency of GGG in ASupstream 10bp sequence |
| 1596 | up10bpdistr1%A | The distribution(position/length) of 1% A in ASupstream 10bp sequence |
| 1597 | up10bpdistr25%A | The distribution(position/length) of 25% A in ASupstream 10bp sequence |
| 1598 | up10bpdistr50%A | The distribution(position/length) of 50% A in ASupstream 10bp sequence |
| 1599 | up10bpdistr75%A | The distribution(position/length) of 75% A in ASupstream 10bp sequence |
| 1600 | up10bpdistr100%A | The distribution(position/length) of 100% A in ASupstream 10bp sequence |
| 1601 | up10bpdistr1%T | The distribution(position/length) of 1% T in ASupstream 10bp sequence |
| 1602 | up10bpdistr25%T | The distribution(position/length) of 25% T in ASupstream 10bp sequence |
| 1603 | up10bpdistr50%T | The distribution(position/length) of 50% T in ASupstream 10bp sequence |
| 1604 | up10bpdistr75%T | The distribution(position/length) of 75% T in ASupstream 10bp sequence |
| 1605 | up10bpdistr100%T | The distribution(position/length) of 100% T in ASupstream 10bp sequence |
| 1606 | up10bpdistr1%C | The distribution(position/length) of 1% C in ASupstream 10bp sequence |
| 1607 | up10bpdistr25%C | The distribution(position/length) of 25% C in ASupstream 10bp sequence |
| 1608 | up10bpdistr50%C | The distribution(position/length) of 50% C in ASupstream 10bp sequence |
| 1609 | up10bpdistr75%C | The distribution(position/length) of 75% C in ASupstream 10bp sequence |
| 1610 | up10bpdistr100%C | The distribution(position/length) of 100% C in ASupstream 10bp sequence |
| 1611 | up10bpdistr1%G | The distribution(position/length) of 1% G in ASupstream 10bp sequence |
| 1612 | up10bpdistr25%G | The distribution(position/length) of 25% G in ASupstream 10bp sequence |
| 1613 | up10bpdistr50%G | The distribution(position/length) of 50% G in ASupstream 10bp sequence |
| 1614 | up10bpdistr75%G | The distribution(position/length) of 75% G in ASupstream 10bp sequence |
| 1615 | up10bpdistr100%G | The distribution(position/length) of 100% G in ASupstream 10bp sequence |
| 1616 | up10bpdonerGT | Is there GT in doner of ASupstream 10bp sequence (1 for yes, 0 for no) |
| 1617 | up10bpacceptorGT | Is there GT in acceptor of ASupstream 10bp sequence (1 for yes, 0 for no) |
| 1618 | up10bpdonerGC | Is there GC in doner of ASupstream 10bp sequence (1 for yes, 0 for no) |
| 1619 | up10bpacceptorGC | Is there GC in acceptor of ASupstream 10bp sequence (1 for yes, 0 for no) |
| 1620 | up10bpdonerAT | Is there AT in doner of ASupstream 10bp sequence (1 for yes, 0 for no) |
| 1621 | up10bpacceptorAT | Is there AT in acceptor of ASupstream 10bp sequence (1 for yes, 0 for no) |
| 1622 | up10bpdonerAG | Is there AG in doner of ASupstream 10bp sequence (1 for yes, 0 for no) |
| 1623 | up10bpacceptorAG | Is there AG in acceptor of ASupstream 10bp sequence (1 for yes, 0 for no) |
| 1624 | up10bpdonerAC | Is there AC in doner of ASupstream 10bp sequence (1 for yes, 0 for no) |
| 1625 | up10bpacceptorAC | Is there AC in acceptor of ASupstream 10bp sequence (1 for yes, 0 for no) |
| 1626 | up20bpif%3 | Whether the length of ASupstream 20bp sequence divisible by three |
| 1627 | up20bpGC | The GC contant of ASupstream 20bp sequence |
| 1628 | up20bpnumberTAA | The number of stopdocon TAA in ASupstream 20bp sequence |
| 1629 | up20bpnumberTAG | The number of stopdocon TAA in ASupstream 20bp sequence |
| 1630 | up20bpnumberTGA | The number of stopdocon TAA in ASupstream 20bp sequence |
| 1631 | up20bpfrequencyA | The frequency of A in ASupstream 20bp sequence |
| 1632 | up20bpfrequencyAA | The frequency of AA in ASupstream 20bp sequence |
| 1633 | up20bpfrequencyAAA | The frequency of AAA in ASupstream 20bp sequence |
| 1634 | up20bpfrequencyAAT | The frequency of AAT in ASupstream 20bp sequence |
| 1635 | up20bpfrequencyAAC | The frequency of AAC in ASupstream 20bp sequence |
| 1636 | up20bpfrequencyAAG | The frequency of AAG in ASupstream 20bp sequence |
| 1637 | up20bpfrequencyAT | The frequency of AT in ASupstream 20bp sequence |
| 1638 | up20bpfrequencyATA | The frequency of ATA in ASupstream 20bp sequence |
| 1639 | up20bpfrequencyATT | The frequency of ATT in ASupstream 20bp sequence |
| 1640 | up20bpfrequencyATC | The frequency of ATC in ASupstream 20bp sequence |
| 1641 | up20bpfrequencyATG | The frequency of ATG in ASupstream 20bp sequence |
| 1642 | up20bpfrequencyAC | The frequency of AC in ASupstream 20bp sequence |
| 1643 | up20bpfrequencyACA | The frequency of ACA in ASupstream 20bp sequence |
| 1644 | up20bpfrequencyACT | The frequency of ACT in ASupstream 20bp sequence |
| 1645 | up20bpfrequencyACC | The frequency of ACC in ASupstream 20bp sequence |
| 1646 | up20bpfrequencyACG | The frequency of ACG in ASupstream 20bp sequence |
| 1647 | up20bpfrequencyAG | The frequency of AG in ASupstream 20bp sequence |
| 1648 | up20bpfrequencyAGA | The frequency of AGA in ASupstream 20bp sequence |
| 1649 | up20bpfrequencyAGT | The frequency of AGT in ASupstream 20bp sequence |
| 1650 | up20bpfrequencyAGC | The frequency of AGC in ASupstream 20bp sequence |
| 1651 | up20bpfrequencyAGG | The frequency of AGG in ASupstream 20bp sequence |
| 1652 | up20bpfrequencyT | The frequency of T in ASupstream 20bp sequence |
| 1653 | up20bpfrequencyTA | The frequency of TA in ASupstream 20bp sequence |
| 1654 | up20bpfrequencyTAA | The frequency of TAA in ASupstream 20bp sequence |
| 1655 | up20bpfrequencyTAT | The frequency of TAT in ASupstream 20bp sequence |
| 1656 | up20bpfrequencyTAC | The frequency of TAC in ASupstream 20bp sequence |
| 1657 | up20bpfrequencyTAG | The frequency of TAG in ASupstream 20bp sequence |
| 1658 | up20bpfrequencyTT | The frequency of TT in ASupstream 20bp sequence |
| 1659 | up20bpfrequencyTTA | The frequency of TTA in ASupstream 20bp sequence |
| 1660 | up20bpfrequencyTTT | The frequency of TTT in ASupstream 20bp sequence |
| 1661 | up20bpfrequencyTTC | The frequency of TTC in ASupstream 20bp sequence |
| 1662 | up20bpfrequencyTTG | The frequency of TTG in ASupstream 20bp sequence |
| 1663 | up20bpfrequencyTC | The frequency of TC in ASupstream 20bp sequence |
| 1664 | up20bpfrequencyTCA | The frequency of TCA in ASupstream 20bp sequence |
| 1665 | up20bpfrequencyTCT | The frequency of TCT in ASupstream 20bp sequence |
| 1666 | up20bpfrequencyTCC | The frequency of TCC in ASupstream 20bp sequence |
| 1667 | up20bpfrequencyTCG | The frequency of TCG in ASupstream 20bp sequence |
| 1668 | up20bpfrequencyTG | The frequency of TG in ASupstream 20bp sequence |
| 1669 | up20bpfrequencyTGA | The frequency of TGA in ASupstream 20bp sequence |
| 1670 | up20bpfrequencyTGT | The frequency of TGT in ASupstream 20bp sequence |
| 1671 | up20bpfrequencyTGC | The frequency of TGC in ASupstream 20bp sequence |
| 1672 | up20bpfrequencyTGG | The frequency of TGG in ASupstream 20bp sequence |
| 1673 | up20bpfrequencyC | The frequency of C in ASupstream 20bp sequence |
| 1674 | up20bpfrequencyCA | The frequency of CA in ASupstream 20bp sequence |
| 1675 | up20bpfrequencyCAA | The frequency of CAA in ASupstream 20bp sequence |
| 1676 | up20bpfrequencyCAT | The frequency of CAT in ASupstream 20bp sequence |
| 1677 | up20bpfrequencyCAC | The frequency of CAC in ASupstream 20bp sequence |
| 1678 | up20bpfrequencyCAG | The frequency of CAG in ASupstream 20bp sequence |
| 1679 | up20bpfrequencyCT | The frequency of CT in ASupstream 20bp sequence |
| 1680 | up20bpfrequencyCTA | The frequency of CTA in ASupstream 20bp sequence |
| 1681 | up20bpfrequencyCTT | The frequency of CTT in ASupstream 20bp sequence |
| 1682 | up20bpfrequencyCTC | The frequency of CTC in ASupstream 20bp sequence |
| 1683 | up20bpfrequencyCTG | The frequency of CTG in ASupstream 20bp sequence |
| 1684 | up20bpfrequencyCC | The frequency of CC in ASupstream 20bp sequence |
| 1685 | up20bpfrequencyCCA | The frequency of CCA in ASupstream 20bp sequence |
| 1686 | up20bpfrequencyCCT | The frequency of CCT in ASupstream 20bp sequence |
| 1687 | up20bpfrequencyCCC | The frequency of CCC in ASupstream 20bp sequence |
| 1688 | up20bpfrequencyCCG | The frequency of CCG in ASupstream 20bp sequence |
| 1689 | up20bpfrequencyCG | The frequency of CG in ASupstream 20bp sequence |
| 1690 | up20bpfrequencyCGA | The frequency of CGA in ASupstream 20bp sequence |
| 1691 | up20bpfrequencyCGT | The frequency of CGT in ASupstream 20bp sequence |
| 1692 | up20bpfrequencyCGC | The frequency of CGC in ASupstream 20bp sequence |
| 1693 | up20bpfrequencyCGG | The frequency of CGG in ASupstream 20bp sequence |
| 1694 | up20bpfrequencyG | The frequency of G in ASupstream 20bp sequence |
| 1695 | up20bpfrequencyGA | The frequency of GA in ASupstream 20bp sequence |
| 1696 | up20bpfrequencyGAA | The frequency of GAA in ASupstream 20bp sequence |
| 1697 | up20bpfrequencyGAT | The frequency of GAT in ASupstream 20bp sequence |
| 1698 | up20bpfrequencyGAC | The frequency of GAC in ASupstream 20bp sequence |
| 1699 | up20bpfrequencyGAG | The frequency of GAG in ASupstream 20bp sequence |
| 1700 | up20bpfrequencyGT | The frequency of GT in ASupstream 20bp sequence |
| 1701 | up20bpfrequencyGTA | The frequency of GTA in ASupstream 20bp sequence |
| 1702 | up20bpfrequencyGTT | The frequency of GTT in ASupstream 20bp sequence |
| 1703 | up20bpfrequencyGTC | The frequency of GTC in ASupstream 20bp sequence |
| 1704 | up20bpfrequencyGTG | The frequency of GTG in ASupstream 20bp sequence |
| 1705 | up20bpfrequencyGC | The frequency of GC in ASupstream 20bp sequence |
| 1706 | up20bpfrequencyGCA | The frequency of GCA in ASupstream 20bp sequence |
| 1707 | up20bpfrequencyGCT | The frequency of GCT in ASupstream 20bp sequence |
| 1708 | up20bpfrequencyGCC | The frequency of GCC in ASupstream 20bp sequence |
| 1709 | up20bpfrequencyGCG | The frequency of GCG in ASupstream 20bp sequence |
| 1710 | up20bpfrequencyGG | The frequency of GG in ASupstream 20bp sequence |
| 1711 | up20bpfrequencyGGA | The frequency of GGA in ASupstream 20bp sequence |
| 1712 | up20bpfrequencyGGT | The frequency of GGT in ASupstream 20bp sequence |
| 1713 | up20bpfrequencyGGC | The frequency of GGC in ASupstream 20bp sequence |
| 1714 | up20bpfrequencyGGG | The frequency of GGG in ASupstream 20bp sequence |
| 1715 | up20bpdistr1%A | The distribution(position/length) of 1% A in ASupstream 20bp sequence |
| 1716 | up20bpdistr25%A | The distribution(position/length) of 25% A in ASupstream 20bp sequence |
| 1717 | up20bpdistr50%A | The distribution(position/length) of 50% A in ASupstream 20bp sequence |
| 1718 | up20bpdistr75%A | The distribution(position/length) of 75% A in ASupstream 20bp sequence |
| 1719 | up20bpdistr100%A | The distribution(position/length) of 100% A in ASupstream 20bp sequence |
| 1720 | up20bpdistr1%T | The distribution(position/length) of 1% T in ASupstream 20bp sequence |
| 1721 | up20bpdistr25%T | The distribution(position/length) of 25% T in ASupstream 20bp sequence |
| 1722 | up20bpdistr50%T | The distribution(position/length) of 50% T in ASupstream 20bp sequence |
| 1723 | up20bpdistr75%T | The distribution(position/length) of 75% T in ASupstream 20bp sequence |
| 1724 | up20bpdistr100%T | The distribution(position/length) of 100% T in ASupstream 20bp sequence |
| 1725 | up20bpdistr1%C | The distribution(position/length) of 1% C in ASupstream 20bp sequence |
| 1726 | up20bpdistr25%C | The distribution(position/length) of 25% C in ASupstream 20bp sequence |
| 1727 | up20bpdistr50%C | The distribution(position/length) of 50% C in ASupstream 20bp sequence |
| 1728 | up20bpdistr75%C | The distribution(position/length) of 75% C in ASupstream 20bp sequence |
| 1729 | up20bpdistr100%C | The distribution(position/length) of 100% C in ASupstream 20bp sequence |
| 1730 | up20bpdistr1%G | The distribution(position/length) of 1% G in ASupstream 20bp sequence |
| 1731 | up20bpdistr25%G | The distribution(position/length) of 25% G in ASupstream 20bp sequence |
| 1732 | up20bpdistr50%G | The distribution(position/length) of 50% G in ASupstream 20bp sequence |
| 1733 | up20bpdistr75%G | The distribution(position/length) of 75% G in ASupstream 20bp sequence |
| 1734 | up20bpdistr100%G | The distribution(position/length) of 100% G in ASupstream 20bp sequence |
| 1735 | up20bpdonerGT | Is there GT in doner of ASupstream 20bp sequence (1 for yes, 0 for no) |
| 1736 | up20bpacceptorGT | Is there GT in acceptor of ASupstream 20bp sequence (1 for yes, 0 for no) |
| 1737 | up20bpdonerGC | Is there GC in doner of ASupstream 20bp sequence (1 for yes, 0 for no) |
| 1738 | up20bpacceptorGC | Is there GC in acceptor of ASupstream 20bp sequence (1 for yes, 0 for no) |
| 1739 | up20bpdonerAT | Is there AT in doner of ASupstream 20bp sequence (1 for yes, 0 for no) |
| 1740 | up20bpacceptorAT | Is there AT in acceptor of ASupstream 20bp sequence (1 for yes, 0 for no) |
| 1741 | up20bpdonerAG | Is there AG in doner of ASupstream 20bp sequence (1 for yes, 0 for no) |
| 1742 | up20bpacceptorAG | Is there AG in acceptor of ASupstream 20bp sequence (1 for yes, 0 for no) |
| 1743 | up20bpdonerAC | Is there AC in doner of ASupstream 20bp sequence (1 for yes, 0 for no) |
| 1744 | up20bpacceptorAC | Is there AC in acceptor of ASupstream 20bp sequence (1 for yes, 0 for no) |
| 1745 | down10bpif%3 | Whether the length of ASdownstream 10bp sequence divisible by three |
| 1746 | down10bpGC | The GC contant of ASdownstream 10bp sequence |
| 1747 | down10bpnumberTAA | The number of stopdocon TAA in ASdownstream 10bp sequence |
| 1748 | down10bpnumberTAG | The number of stopdocon TAA in ASdownstream 10bp sequence |
| 1749 | down10bpnumberTGA | The number of stopdocon TAA in ASdownstream 10bp sequence |
| 1750 | down10bpfrequencyA | The frequency of A in ASdownstream 10bp sequence |
| 1751 | down10bpfrequencyAA | The frequency of AA in ASdownstream 10bp sequence |
| 1752 | down10bpfrequencyAAA | The frequency of AAA in ASdownstream 10bp sequence |
| 1753 | down10bpfrequencyAAT | The frequency of AAT in ASdownstream 10bp sequence |
| 1754 | down10bpfrequencyAAC | The frequency of AAC in ASdownstream 10bp sequence |
| 1755 | down10bpfrequencyAAG | The frequency of AAG in ASdownstream 10bp sequence |
| 1756 | down10bpfrequencyAT | The frequency of AT in ASdownstream 10bp sequence |
| 1757 | down10bpfrequencyATA | The frequency of ATA in ASdownstream 10bp sequence |
| 1758 | down10bpfrequencyATT | The frequency of ATT in ASdownstream 10bp sequence |
| 1759 | down10bpfrequencyATC | The frequency of ATC in ASdownstream 10bp sequence |
| 1760 | down10bpfrequencyATG | The frequency of ATG in ASdownstream 10bp sequence |
| 1761 | down10bpfrequencyAC | The frequency of AC in ASdownstream 10bp sequence |
| 1762 | down10bpfrequencyACA | The frequency of ACA in ASdownstream 10bp sequence |
| 1763 | down10bpfrequencyACT | The frequency of ACT in ASdownstream 10bp sequence |
| 1764 | down10bpfrequencyACC | The frequency of ACC in ASdownstream 10bp sequence |
| 1765 | down10bpfrequencyACG | The frequency of ACG in ASdownstream 10bp sequence |
| 1766 | down10bpfrequencyAG | The frequency of AG in ASdownstream 10bp sequence |
| 1767 | down10bpfrequencyAGA | The frequency of AGA in ASdownstream 10bp sequence |
| 1768 | down10bpfrequencyAGT | The frequency of AGT in ASdownstream 10bp sequence |
| 1769 | down10bpfrequencyAGC | The frequency of AGC in ASdownstream 10bp sequence |
| 1770 | down10bpfrequencyAGG | The frequency of AGG in ASdownstream 10bp sequence |
| 1771 | down10bpfrequencyT | The frequency of T in ASdownstream 10bp sequence |
| 1772 | down10bpfrequencyTA | The frequency of TA in ASdownstream 10bp sequence |
| 1773 | down10bpfrequencyTAA | The frequency of TAA in ASdownstream 10bp sequence |
| 1774 | down10bpfrequencyTAT | The frequency of TAT in ASdownstream 10bp sequence |
| 1775 | down10bpfrequencyTAC | The frequency of TAC in ASdownstream 10bp sequence |
| 1776 | down10bpfrequencyTAG | The frequency of TAG in ASdownstream 10bp sequence |
| 1777 | down10bpfrequencyTT | The frequency of TT in ASdownstream 10bp sequence |
| 1778 | down10bpfrequencyTTA | The frequency of TTA in ASdownstream 10bp sequence |
| 1779 | down10bpfrequencyTTT | The frequency of TTT in ASdownstream 10bp sequence |
| 1780 | down10bpfrequencyTTC | The frequency of TTC in ASdownstream 10bp sequence |
| 1781 | down10bpfrequencyTTG | The frequency of TTG in ASdownstream 10bp sequence |
| 1782 | down10bpfrequencyTC | The frequency of TC in ASdownstream 10bp sequence |
| 1783 | down10bpfrequencyTCA | The frequency of TCA in ASdownstream 10bp sequence |
| 1784 | down10bpfrequencyTCT | The frequency of TCT in ASdownstream 10bp sequence |
| 1785 | down10bpfrequencyTCC | The frequency of TCC in ASdownstream 10bp sequence |
| 1786 | down10bpfrequencyTCG | The frequency of TCG in ASdownstream 10bp sequence |
| 1787 | down10bpfrequencyTG | The frequency of TG in ASdownstream 10bp sequence |
| 1788 | down10bpfrequencyTGA | The frequency of TGA in ASdownstream 10bp sequence |
| 1789 | down10bpfrequencyTGT | The frequency of TGT in ASdownstream 10bp sequence |
| 1790 | down10bpfrequencyTGC | The frequency of TGC in ASdownstream 10bp sequence |
| 1791 | down10bpfrequencyTGG | The frequency of TGG in ASdownstream 10bp sequence |
| 1792 | down10bpfrequencyC | The frequency of C in ASdownstream 10bp sequence |
| 1793 | down10bpfrequencyCA | The frequency of CA in ASdownstream 10bp sequence |
| 1794 | down10bpfrequencyCAA | The frequency of CAA in ASdownstream 10bp sequence |
| 1795 | down10bpfrequencyCAT | The frequency of CAT in ASdownstream 10bp sequence |
| 1796 | down10bpfrequencyCAC | The frequency of CAC in ASdownstream 10bp sequence |
| 1797 | down10bpfrequencyCAG | The frequency of CAG in ASdownstream 10bp sequence |
| 1798 | down10bpfrequencyCT | The frequency of CT in ASdownstream 10bp sequence |
| 1799 | down10bpfrequencyCTA | The frequency of CTA in ASdownstream 10bp sequence |
| 1800 | down10bpfrequencyCTT | The frequency of CTT in ASdownstream 10bp sequence |
| 1801 | down10bpfrequencyCTC | The frequency of CTC in ASdownstream 10bp sequence |
| 1802 | down10bpfrequencyCTG | The frequency of CTG in ASdownstream 10bp sequence |
| 1803 | down10bpfrequencyCC | The frequency of CC in ASdownstream 10bp sequence |
| 1804 | down10bpfrequencyCCA | The frequency of CCA in ASdownstream 10bp sequence |
| 1805 | down10bpfrequencyCCT | The frequency of CCT in ASdownstream 10bp sequence |
| 1806 | down10bpfrequencyCCC | The frequency of CCC in ASdownstream 10bp sequence |
| 1807 | down10bpfrequencyCCG | The frequency of CCG in ASdownstream 10bp sequence |
| 1808 | down10bpfrequencyCG | The frequency of CG in ASdownstream 10bp sequence |
| 1809 | down10bpfrequencyCGA | The frequency of CGA in ASdownstream 10bp sequence |
| 1810 | down10bpfrequencyCGT | The frequency of CGT in ASdownstream 10bp sequence |
| 1811 | down10bpfrequencyCGC | The frequency of CGC in ASdownstream 10bp sequence |
| 1812 | down10bpfrequencyCGG | The frequency of CGG in ASdownstream 10bp sequence |
| 1813 | down10bpfrequencyG | The frequency of G in ASdownstream 10bp sequence |
| 1814 | down10bpfrequencyGA | The frequency of GA in ASdownstream 10bp sequence |
| 1815 | down10bpfrequencyGAA | The frequency of GAA in ASdownstream 10bp sequence |
| 1816 | down10bpfrequencyGAT | The frequency of GAT in ASdownstream 10bp sequence |
| 1817 | down10bpfrequencyGAC | The frequency of GAC in ASdownstream 10bp sequence |
| 1818 | down10bpfrequencyGAG | The frequency of GAG in ASdownstream 10bp sequence |
| 1819 | down10bpfrequencyGT | The frequency of GT in ASdownstream 10bp sequence |
| 1820 | down10bpfrequencyGTA | The frequency of GTA in ASdownstream 10bp sequence |
| 1821 | down10bpfrequencyGTT | The frequency of GTT in ASdownstream 10bp sequence |
| 1822 | down10bpfrequencyGTC | The frequency of GTC in ASdownstream 10bp sequence |
| 1823 | down10bpfrequencyGTG | The frequency of GTG in ASdownstream 10bp sequence |
| 1824 | down10bpfrequencyGC | The frequency of GC in ASdownstream 10bp sequence |
| 1825 | down10bpfrequencyGCA | The frequency of GCA in ASdownstream 10bp sequence |
| 1826 | down10bpfrequencyGCT | The frequency of GCT in ASdownstream 10bp sequence |
| 1827 | down10bpfrequencyGCC | The frequency of GCC in ASdownstream 10bp sequence |
| 1828 | down10bpfrequencyGCG | The frequency of GCG in ASdownstream 10bp sequence |
| 1829 | down10bpfrequencyGG | The frequency of GG in ASdownstream 10bp sequence |
| 1830 | down10bpfrequencyGGA | The frequency of GGA in ASdownstream 10bp sequence |
| 1831 | down10bpfrequencyGGT | The frequency of GGT in ASdownstream 10bp sequence |
| 1832 | down10bpfrequencyGGC | The frequency of GGC in ASdownstream 10bp sequence |
| 1833 | down10bpfrequencyGGG | The frequency of GGG in ASdownstream 10bp sequence |
| 1834 | down10bpdistr1%A | The distribution(position/length) of 1% A in ASdownstream 10bp sequence |
| 1835 | down10bpdistr25%A | The distribution(position/length) of 25% A in ASdownstream 10bp sequence |
| 1836 | down10bpdistr50%A | The distribution(position/length) of 50% A in ASdownstream 10bp sequence |
| 1837 | down10bpdistr75%A | The distribution(position/length) of 75% A in ASdownstream 10bp sequence |
| 1838 | down10bpdistr100%A | The distribution(position/length) of 100% A in ASdownstream 10bp sequence |
| 1839 | down10bpdistr1%T | The distribution(position/length) of 1% T in ASdownstream 10bp sequence |
| 1840 | down10bpdistr25%T | The distribution(position/length) of 25% T in ASdownstream 10bp sequence |
| 1841 | down10bpdistr50%T | The distribution(position/length) of 50% T in ASdownstream 10bp sequence |
| 1842 | down10bpdistr75%T | The distribution(position/length) of 75% T in ASdownstream 10bp sequence |
| 1843 | down10bpdistr100%T | The distribution(position/length) of 100% T in ASdownstream 10bp sequence |
| 1844 | down10bpdistr1%C | The distribution(position/length) of 1% C in ASdownstream 10bp sequence |
| 1845 | down10bpdistr25%C | The distribution(position/length) of 25% C in ASdownstream 10bp sequence |
| 1846 | down10bpdistr50%C | The distribution(position/length) of 50% C in ASdownstream 10bp sequence |
| 1847 | down10bpdistr75%C | The distribution(position/length) of 75% C in ASdownstream 10bp sequence |
| 1848 | down10bpdistr100%C | The distribution(position/length) of 100% C in ASdownstream 10bp sequence |
| 1849 | down10bpdistr1%G | The distribution(position/length) of 1% G in ASdownstream 10bp sequence |
| 1850 | down10bpdistr25%G | The distribution(position/length) of 25% G in ASdownstream 10bp sequence |
| 1851 | down10bpdistr50%G | The distribution(position/length) of 50% G in ASdownstream 10bp sequence |
| 1852 | down10bpdistr75%G | The distribution(position/length) of 75% G in ASdownstream 10bp sequence |
| 1853 | down10bpdistr100%G | The distribution(position/length) of 100% G in ASdownstream 10bp sequence |
| 1854 | down10bpdonerGT | Is there GT in doner of ASdownstream 10bp sequence (1 for yes, 0 for no) |
| 1855 | down10bpacceptorGT | Is there GT in acceptor of ASdownstream 10bp sequence (1 for yes, 0 for no) |
| 1856 | down10bpdonerGC | Is there GC in doner of ASdownstream 10bp sequence (1 for yes, 0 for no) |
| 1857 | down10bpacceptorGC | Is there GC in acceptor of ASdownstream 10bp sequence (1 for yes, 0 for no) |
| 1858 | down10bpdonerAT | Is there AT in doner of ASdownstream 10bp sequence (1 for yes, 0 for no) |
| 1859 | down10bpacceptorAT | Is there AT in acceptor of ASdownstream 10bp sequence (1 for yes, 0 for no) |
| 1860 | down10bpdonerAG | Is there AG in doner of ASdownstream 10bp sequence (1 for yes, 0 for no) |
| 1861 | down10bpacceptorAG | Is there AG in acceptor of ASdownstream 10bp sequence (1 for yes, 0 for no) |
| 1862 | down10bpdonerAC | Is there AC in doner of ASdownstream 10bp sequence (1 for yes, 0 for no) |
| 1863 | down10bpacceptorAC | Is there AC in acceptor of ASdownstream 10bp sequence (1 for yes, 0 for no) |
| 1864 | down20bpif%3 | Whether the length of ASdownstream 20bp sequence divisible by three |
| 1865 | down20bpGC | The GC contant of ASdownstream 20bp sequence |
| 1866 | down20bpnumberTAA | The number of stopdocon TAA in ASdownstream 20bp sequence |
| 1867 | down20bpnumberTAG | The number of stopdocon TAA in ASdownstream 20bp sequence |
| 1868 | down20bpnumberTGA | The number of stopdocon TAA in ASdownstream 20bp sequence |
| 1869 | down20bpfrequencyA | The frequency of A in ASdownstream 20bp sequence |
| 1870 | down20bpfrequencyAA | The frequency of AA in ASdownstream 20bp sequence |
| 1871 | down20bpfrequencyAAA | The frequency of AAA in ASdownstream 20bp sequence |
| 1872 | down20bpfrequencyAAT | The frequency of AAT in ASdownstream 20bp sequence |
| 1873 | down20bpfrequencyAAC | The frequency of AAC in ASdownstream 20bp sequence |
| 1874 | down20bpfrequencyAAG | The frequency of AAG in ASdownstream 20bp sequence |
| 1875 | down20bpfrequencyAT | The frequency of AT in ASdownstream 20bp sequence |
| 1876 | down20bpfrequencyATA | The frequency of ATA in ASdownstream 20bp sequence |
| 1877 | down20bpfrequencyATT | The frequency of ATT in ASdownstream 20bp sequence |
| 1878 | down20bpfrequencyATC | The frequency of ATC in ASdownstream 20bp sequence |
| 1879 | down20bpfrequencyATG | The frequency of ATG in ASdownstream 20bp sequence |
| 1880 | down20bpfrequencyAC | The frequency of AC in ASdownstream 20bp sequence |
| 1881 | down20bpfrequencyACA | The frequency of ACA in ASdownstream 20bp sequence |
| 1882 | down20bpfrequencyACT | The frequency of ACT in ASdownstream 20bp sequence |
| 1883 | down20bpfrequencyACC | The frequency of ACC in ASdownstream 20bp sequence |
| 1884 | down20bpfrequencyACG | The frequency of ACG in ASdownstream 20bp sequence |
| 1885 | down20bpfrequencyAG | The frequency of AG in ASdownstream 20bp sequence |
| 1886 | down20bpfrequencyAGA | The frequency of AGA in ASdownstream 20bp sequence |
| 1887 | down20bpfrequencyAGT | The frequency of AGT in ASdownstream 20bp sequence |
| 1888 | down20bpfrequencyAGC | The frequency of AGC in ASdownstream 20bp sequence |
| 1889 | down20bpfrequencyAGG | The frequency of AGG in ASdownstream 20bp sequence |
| 1890 | down20bpfrequencyT | The frequency of T in ASdownstream 20bp sequence |
| 1891 | down20bpfrequencyTA | The frequency of TA in ASdownstream 20bp sequence |
| 1892 | down20bpfrequencyTAA | The frequency of TAA in ASdownstream 20bp sequence |
| 1893 | down20bpfrequencyTAT | The frequency of TAT in ASdownstream 20bp sequence |
| 1894 | down20bpfrequencyTAC | The frequency of TAC in ASdownstream 20bp sequence |
| 1895 | down20bpfrequencyTAG | The frequency of TAG in ASdownstream 20bp sequence |
| 1896 | down20bpfrequencyTT | The frequency of TT in ASdownstream 20bp sequence |
| 1897 | down20bpfrequencyTTA | The frequency of TTA in ASdownstream 20bp sequence |
| 1898 | down20bpfrequencyTTT | The frequency of TTT in ASdownstream 20bp sequence |
| 1899 | down20bpfrequencyTTC | The frequency of TTC in ASdownstream 20bp sequence |
| 1900 | down20bpfrequencyTTG | The frequency of TTG in ASdownstream 20bp sequence |
| 1901 | down20bpfrequencyTC | The frequency of TC in ASdownstream 20bp sequence |
| 1902 | down20bpfrequencyTCA | The frequency of TCA in ASdownstream 20bp sequence |
| 1903 | down20bpfrequencyTCT | The frequency of TCT in ASdownstream 20bp sequence |
| 1904 | down20bpfrequencyTCC | The frequency of TCC in ASdownstream 20bp sequence |
| 1905 | down20bpfrequencyTCG | The frequency of TCG in ASdownstream 20bp sequence |
| 1906 | down20bpfrequencyTG | The frequency of TG in ASdownstream 20bp sequence |
| 1907 | down20bpfrequencyTGA | The frequency of TGA in ASdownstream 20bp sequence |
| 1908 | down20bpfrequencyTGT | The frequency of TGT in ASdownstream 20bp sequence |
| 1909 | down20bpfrequencyTGC | The frequency of TGC in ASdownstream 20bp sequence |
| 1910 | down20bpfrequencyTGG | The frequency of TGG in ASdownstream 20bp sequence |
| 1911 | down20bpfrequencyC | The frequency of C in ASdownstream 20bp sequence |
| 1912 | down20bpfrequencyCA | The frequency of CA in ASdownstream 20bp sequence |
| 1913 | down20bpfrequencyCAA | The frequency of CAA in ASdownstream 20bp sequence |
| 1914 | down20bpfrequencyCAT | The frequency of CAT in ASdownstream 20bp sequence |
| 1915 | down20bpfrequencyCAC | The frequency of CAC in ASdownstream 20bp sequence |
| 1916 | down20bpfrequencyCAG | The frequency of CAG in ASdownstream 20bp sequence |
| 1917 | down20bpfrequencyCT | The frequency of CT in ASdownstream 20bp sequence |
| 1918 | down20bpfrequencyCTA | The frequency of CTA in ASdownstream 20bp sequence |
| 1919 | down20bpfrequencyCTT | The frequency of CTT in ASdownstream 20bp sequence |
| 1920 | down20bpfrequencyCTC | The frequency of CTC in ASdownstream 20bp sequence |
| 1921 | down20bpfrequencyCTG | The frequency of CTG in ASdownstream 20bp sequence |
| 1922 | down20bpfrequencyCC | The frequency of CC in ASdownstream 20bp sequence |
| 1923 | down20bpfrequencyCCA | The frequency of CCA in ASdownstream 20bp sequence |
| 1924 | down20bpfrequencyCCT | The frequency of CCT in ASdownstream 20bp sequence |
| 1925 | down20bpfrequencyCCC | The frequency of CCC in ASdownstream 20bp sequence |
| 1926 | down20bpfrequencyCCG | The frequency of CCG in ASdownstream 20bp sequence |
| 1927 | down20bpfrequencyCG | The frequency of CG in ASdownstream 20bp sequence |
| 1928 | down20bpfrequencyCGA | The frequency of CGA in ASdownstream 20bp sequence |
| 1929 | down20bpfrequencyCGT | The frequency of CGT in ASdownstream 20bp sequence |
| 1930 | down20bpfrequencyCGC | The frequency of CGC in ASdownstream 20bp sequence |
| 1931 | down20bpfrequencyCGG | The frequency of CGG in ASdownstream 20bp sequence |
| 1932 | down20bpfrequencyG | The frequency of G in ASdownstream 20bp sequence |
| 1933 | down20bpfrequencyGA | The frequency of GA in ASdownstream 20bp sequence |
| 1934 | down20bpfrequencyGAA | The frequency of GAA in ASdownstream 20bp sequence |
| 1935 | down20bpfrequencyGAT | The frequency of GAT in ASdownstream 20bp sequence |
| 1936 | down20bpfrequencyGAC | The frequency of GAC in ASdownstream 20bp sequence |
| 1937 | down20bpfrequencyGAG | The frequency of GAG in ASdownstream 20bp sequence |
| 1938 | down20bpfrequencyGT | The frequency of GT in ASdownstream 20bp sequence |
| 1939 | down20bpfrequencyGTA | The frequency of GTA in ASdownstream 20bp sequence |
| 1940 | down20bpfrequencyGTT | The frequency of GTT in ASdownstream 20bp sequence |
| 1941 | down20bpfrequencyGTC | The frequency of GTC in ASdownstream 20bp sequence |
| 1942 | down20bpfrequencyGTG | The frequency of GTG in ASdownstream 20bp sequence |
| 1943 | down20bpfrequencyGC | The frequency of GC in ASdownstream 20bp sequence |
| 1944 | down20bpfrequencyGCA | The frequency of GCA in ASdownstream 20bp sequence |
| 1945 | down20bpfrequencyGCT | The frequency of GCT in ASdownstream 20bp sequence |
| 1946 | down20bpfrequencyGCC | The frequency of GCC in ASdownstream 20bp sequence |
| 1947 | down20bpfrequencyGCG | The frequency of GCG in ASdownstream 20bp sequence |
| 1948 | down20bpfrequencyGG | The frequency of GG in ASdownstream 20bp sequence |
| 1949 | down20bpfrequencyGGA | The frequency of GGA in ASdownstream 20bp sequence |
| 1950 | down20bpfrequencyGGT | The frequency of GGT in ASdownstream 20bp sequence |
| 1951 | down20bpfrequencyGGC | The frequency of GGC in ASdownstream 20bp sequence |
| 1952 | down20bpfrequencyGGG | The frequency of GGG in ASdownstream 20bp sequence |
| 1953 | down20bpdistr1%A | The distribution(position/length) of 1% A in ASdownstream 20bp sequence |
| 1954 | down20bpdistr25%A | The distribution(position/length) of 25% A in ASdownstream 20bp sequence |
| 1955 | down20bpdistr50%A | The distribution(position/length) of 50% A in ASdownstream 20bp sequence |
| 1956 | down20bpdistr75%A | The distribution(position/length) of 75% A in ASdownstream 20bp sequence |
| 1957 | down20bpdistr100%A | The distribution(position/length) of 100% A in ASdownstream 20bp sequence |
| 1958 | down20bpdistr1%T | The distribution(position/length) of 1% T in ASdownstream 20bp sequence |
| 1959 | down20bpdistr25%T | The distribution(position/length) of 25% T in ASdownstream 20bp sequence |
| 1960 | down20bpdistr50%T | The distribution(position/length) of 50% T in ASdownstream 20bp sequence |
| 1961 | down20bpdistr75%T | The distribution(position/length) of 75% T in ASdownstream 20bp sequence |
| 1962 | down20bpdistr100%T | The distribution(position/length) of 100% T in ASdownstream 20bp sequence |
| 1963 | down20bpdistr1%C | The distribution(position/length) of 1% C in ASdownstream 20bp sequence |
| 1964 | down20bpdistr25%C | The distribution(position/length) of 25% C in ASdownstream 20bp sequence |
| 1965 | down20bpdistr50%C | The distribution(position/length) of 50% C in ASdownstream 20bp sequence |
| 1966 | down20bpdistr75%C | The distribution(position/length) of 75% C in ASdownstream 20bp sequence |
| 1967 | down20bpdistr100%C | The distribution(position/length) of 100% C in ASdownstream 20bp sequence |
| 1968 | down20bpdistr1%G | The distribution(position/length) of 1% G in ASdownstream 20bp sequence |
| 1969 | down20bpdistr25%G | The distribution(position/length) of 25% G in ASdownstream 20bp sequence |
| 1970 | down20bpdistr50%G | The distribution(position/length) of 50% G in ASdownstream 20bp sequence |
| 1971 | down20bpdistr75%G | The distribution(position/length) of 75% G in ASdownstream 20bp sequence |
| 1972 | down20bpdistr100%G | The distribution(position/length) of 100% G in ASdownstream 20bp sequence |
| 1973 | down20bpdonerGT | Is there GT in doner of ASdownstream 20bp sequence (1 for yes, 0 for no) |
| 1974 | down20bpacceptorGT | Is there GT in acceptor of ASdownstream 20bp sequence (1 for yes, 0 for no) |
| 1975 | down20bpdonerGC | Is there GC in doner of ASdownstream 20bp sequence (1 for yes, 0 for no) |
| 1976 | down20bpacceptorGC | Is there GC in acceptor of ASdownstream 20bp sequence (1 for yes, 0 for no) |
| 1977 | down20bpdonerAT | Is there AT in doner of ASdownstream 20bp sequence (1 for yes, 0 for no) |
| 1978 | down20bpacceptorAT | Is there AT in acceptor of ASdownstream 20bp sequence (1 for yes, 0 for no) |
| 1979 | down20bpdonerAG | Is there AG in doner of ASdownstream 20bp sequence (1 for yes, 0 for no) |
| 1980 | down20bpacceptorAG | Is there AG in acceptor of ASdownstream 20bp sequence (1 for yes, 0 for no) |
| 1981 | down20bpdonerAC | Is there AC in doner of ASdownstream 20bp sequence (1 for yes, 0 for no) |
| 1982 | down20bpacceptorAC | Is there AC in acceptor of ASdownstream 20bp sequence (1 for yes, 0 for no) |

Table S5 Performance of identifying AS transcript pairs among mkcDBGAS and existing methods taking the results as SUPPA2 as ground truth

| Methods | Metrics | Human | *Arabidopsis thaliana* |
| --- | --- | --- | --- |
| mkcDBGAS | Predicted AS transcript pairs | 223 848 | 24 163 |
|  | True positive | 220 983 | 23 968 |
|  | Precision (%) | 98.72 | 99.19 |
|  | Recall (%) | 94.88 | 96.01 |
|  | F1-score | 0.97 | 0.98 |
| DeepASmRNA | Predicted AS transcript pairs | 204 910 | 21 747 |
|  | True positive | 187 083 | 20 595 |
|  | Precision (%) | 91.30 | 94.70 |
|  | Recall (%) | 80.32 | 82.50 |
|  | F1-score | 0.85 | 0.88 |
| AStrap | Predicted AS transcript pairs | 44 085 | 9791 |
|  | True positive | 22 081 | 9373 |
|  | Precision (%) | 50.08 | 95.73 |
|  | Recall (%) | 9.48 | 37.55 |
|  | F1-score | 0.16 | 0.54 |
| IsoSplitter | Predicted AS transcript pairs | 259 915 | 20 376 |
|  | True positive | 125 276 | 16 641 |
|  | Precision (%) | 48.20 | 81.67 |
|  | Recall (%) | 53.79 | 66.66 |
|  | F1-score | 0.51 | 0.73 |

Table S6 Features of the human model

| No. | Features | Importance | Description |
| --- | --- | --- | --- |
| 1 | length_of_as | 0.00321023 | Length of AS region |
| 2 | DmotifAAAGA | 0.00051058 | Is there this motif in downstream of splicing site: AAAGA (1 for yes, 0 for no) |
| 3 | DmotifTTCTT | 0.00061618 | Is there this motif in downstream of splicing site: TTCTT (1 for yes, 0 for no) |
| 4 | DmotifGTAGG | 0.00266274 | Is there this motif in downstream of splicing site: GTAGG (1 for yes, 0 for no) |
| 5 | DmotifTGAGG | 0.00068823 | Is there this motif in downstream of splicing site: TGAGG (1 for yes, 0 for no) |
| 6 | DmotifTCTTT | 0.00087175 | Is there this motif in downstream of splicing site: TCTTT (1 for yes, 0 for no) |
| 7 | DmotifTAAGT | 0.00069154 | Is there this motif in downstream of splicing site: TAAGT (1 for yes, 0 for no) |
| 8 | DmotifGTGAG | 0.00479803 | Is there this motif in downstream of splicing site: GTGAG (1 for yes, 0 for no) |
| 9 | DmotifGTAAG | 0.00259515 | Is there this motif in downstream of splicing site: GTAAG (1 for yes, 0 for no) |
| 10 | DmotifGTCTG | 0.00071273 | Is there this motif in downstream of splicing site: GTCTG (1 for yes, 0 for no) |
| 11 | DmotifGTTTT | 0.00067621 | Is there this motif in downstream of splicing site: GTTTT (1 for yes, 0 for no) |
| 12 | DmotifTTCTCT | 0.00104417 | Is there this motif in downstream of splicing site: TTCTCT (1 for yes, 0 for no) |
| 13 | DmotifTAAGG | 0.00058586 | Is there this motif in downstream of splicing site: TAAGG (1 for yes, 0 for no) |
| 14 | DmotifTCCTTT | 0.00057234 | Is there this motif in downstream of splicing site: TCCTTT (1 for yes, 0 for no) |
| 15 | DmotifTGTCT | 0.00074713 | Is there this motif in downstream of splicing site: TGTCT (1 for yes, 0 for no) |
| 16 | DmotifTTTCTC | 0.00089086 | Is there this motif in downstream of splicing site: TTTCTC (1 for yes, 0 for no) |
| 17 | DmotifCTTTT | 0.00085476 | Is there this motif in downstream of splicing site: CTTTT (1 for yes, 0 for no) |
| 18 | DmotifTGAGT | 0.00124747 | Is there this motif in downstream of splicing site: TGAGT (1 for yes, 0 for no) |
| 19 | DmotifTAGGT | 0.00133475 | Is there this motif in downstream of splicing site: TAGGT (1 for yes, 0 for no) |
| 20 | DmotifCTTTA | 0.00052679 | Is there this motif in downstream of splicing site: CTTTA (1 for yes, 0 for no) |
| 21 | DmotifTTTAG | 0.00078008 | Is there this motif in downstream of splicing site: TTTAG (1 for yes, 0 for no) |
| 22 | DmotifTGCTT | 0.00070491 | Is there this motif in downstream of splicing site: TGCTT (1 for yes, 0 for no) |
| 23 | DmotifGTGGGT | 0.00094222 | Is there this motif in downstream of splicing site: GTGGGT (1 for yes, 0 for no) |
| 24 | DmotifTCTCC | 0.00068126 | Is there this motif in downstream of splicing site: TCTCC (1 for yes, 0 for no) |
| 25 | DmotifTTTTTC | 0.00139376 | Is there this motif in downstream of splicing site: TTTTTC (1 for yes, 0 for no) |
| 26 | UmotifTTCTT | 0.00054549 | Is there this motif in upstream of splicing site: TTCTT (1 for yes, 0 for no) |
| 27 | UmotifTCTTT | 0.00096571 | Is there this motif in upstream of splicing site: TCTTT (1 for yes, 0 for no) |
| 28 | UmotifTAAGT | 0.00066801 | Is there this motif in upstream of splicing site: TAAGT (1 for yes, 0 for no) |
| 29 | UmotifCTCTG | 0.00061516 | Is there this motif in upstream of splicing site: CTCTG (1 for yes, 0 for no) |
| 30 | UmotifTTTTCC | 0.0007502 | Is there this motif in upstream of splicing site: TTTTCC (1 for yes, 0 for no) |
| 31 | UmotifGTGAG | 0.00550866 | Is there this motif in upstream of splicing site: GTGAG (1 for yes, 0 for no) |
| 32 | UmotifGTAAG | 0.00238317 | Is there this motif in upstream of splicing site: GTAAG (1 for yes, 0 for no) |
| 33 | UmotifCTTCT | 0.00051282 | Is there this motif in upstream of splicing site: CTTCT (1 for yes, 0 for no) |
| 34 | UmotifGTTTT | 0.00054749 | Is there this motif in upstream of splicing site: GTTTT (1 for yes, 0 for no) |
| 35 | UmotifCCTCT | 0.00097555 | Is there this motif in upstream of splicing site: CCTCT (1 for yes, 0 for no) |
| 36 | UmotifTCTCT | 0.00117784 | Is there this motif in upstream of splicing site: TCTCT (1 for yes, 0 for no) |
| 37 | UmotifTGTCT | 0.00059122 | Is there this motif in upstream of splicing site: TGTCT (1 for yes, 0 for no) |
| 38 | UmotifTTCCTT | 0.0005197 | Is there this motif in upstream of splicing site: TTCCTT (1 for yes, 0 for no) |
| 39 | UmotifCTTTT | 0.00080732 | Is there this motif in upstream of splicing site: CTTTT (1 for yes, 0 for no) |
| 40 | UmotifTGAGT | 0.00114328 | Is there this motif in upstream of splicing site: TGAGT (1 for yes, 0 for no) |
| 41 | UmotifCCCCAG | 0.00096588 | Is there this motif in upstream of splicing site: CCCCAG (1 for yes, 0 for no) |
| 42 | UmotifTTTAG | 0.00064961 | Is there this motif in upstream of splicing site: TTTAG (1 for yes, 0 for no) |
| 43 | UmotifTTCTC | 0.00079194 | Is there this motif in upstream of splicing site: TTCTC (1 for yes, 0 for no) |
| 44 | UmotifTGCTT | 0.00068789 | Is there this motif in upstream of splicing site: TGCTT (1 for yes, 0 for no) |
| 45 | allseqGC | 0.00186719 | The GC contant of all sequence |
| 46 | allseqnumberTAA | 0.00085041 | The number of stopdocon TAA in all sequence |
| 47 | allseqnumberTAG | 0.00100172 | The number of stopdocon TAG in all sequence |
| 48 | allseqnumberTGA | 0.00072766 | The number of stopdocon TGA in all sequence |
| 49 | allseqfrequencyA | 0.00094488 | The frequency of A in all sequence |
| 50 | allseqfrequencyAA | 0.00091142 | The frequency of AA in all sequence |
| 51 | allseqfrequencyAAA | 0.00050729 | The frequency of AAA in all sequence |
| 52 | allseqfrequencyAT | 0.00101279 | The frequency of AT in all sequence |
| 53 | allseqfrequencyAC | 0.00053169 | The frequency of AC in all sequence |
| 54 | allseqfrequencyACA | 0.0005611 | The frequency of ACA in all sequence |
| 55 | allseqfrequencyAGG | 0.00052174 | The frequency of AGG in all sequence |
| 56 | allseqfrequencyTT | 0.00054358 | The frequency of TT in all sequence |
| 57 | allseqfrequencyTTT | 0.00056096 | The frequency of TTT in all sequence |
| 58 | allseqfrequencyC | 0.0006169 | The frequency of C in all sequence |
| 59 | allseqfrequencyCA | 0.00096057 | The frequency of CA in all sequence |
| 60 | allseqfrequencyCAA | 0.00084891 | The frequency of CAA in all sequence |
| 61 | allseqfrequencyCC | 0.00064007 | The frequency of CC in all sequence |
| 62 | allseqfrequencyCCT | 0.00065494 | The frequency of CCT in all sequence |
| 63 | allseqfrequencyCCC | 0.00098559 | The frequency of CCC in all sequence |
| 64 | allseqfrequencyCCG | 0.00073578 | The frequency of CCG in all sequence |
| 65 | allseqfrequencyCG | 0.00175652 | The frequency of CG in all sequence |
| 66 | allseqfrequencyCGC | 0.00078686 | The frequency of CGC in all sequence |
| 67 | allseqfrequencyCGG | 0.00133533 | The frequency of CGG in all sequence |
| 68 | allseqfrequencyG | 0.00172969 | The frequency of G in all sequence |
| 69 | allseqfrequencyGA | 0.00059723 | The frequency of GA in all sequence |
| 70 | allseqfrequencyGAA | 0.00061731 | The frequency of GAA in all sequence |
| 71 | allseqfrequencyGTA | 0.00053189 | The frequency of GTA in all sequence |
| 72 | allseqfrequencyGC | 0.00065331 | The frequency of GC in all sequence |
| 73 | allseqfrequencyGCG | 0.00089575 | The frequency of GCG in all sequence |
| 74 | allseqfrequencyGG | 0.00104378 | The frequency of GG in all sequence |
| 75 | allseqfrequencyGGT | 0.00074696 | The frequency of GGT in all sequence |
| 76 | allseqfrequencyGGC | 0.00052334 | The frequency of GGC in all sequence |
| 77 | allseqfrequencyGGG | 0.00212446 | The frequency of GGG in all sequence |
| 78 | allseqdistr50%A | 0.00057426 | The distribution(position/length) of 50%A in all sequence |
| 79 | allseqdistr100%G | 0.00077109 | The distribution(position/length) of 100%G in all sequence |
| 80 | allseqdonerAG | 0.00066435 | Is AG in doner of all sequence (1 for yes, 0 for no) |
| 81 | asseqGC | 0.00273557 | The GC contant of AS region sequence |
| 82 | asseqnumberTAA | 0.00125697 | The number of stopdocon TAA in AS region sequence |
| 83 | asseqnumberTAG | 0.00141324 | The number of stopdocon TAG in AS region sequence |
| 84 | asseqnumberTGA | 0.0011946 | The number of stopdocon TGA in AS region sequence |
| 85 | asseqfrequencyA | 0.00209854 | The frequency of A in AS region sequence |
| 86 | asseqfrequencyAA | 0.00189235 | The frequency of AA in AS region sequence |
| 87 | asseqfrequencyAAA | 0.00068023 | The frequency of AAA in AS region sequence |
| 88 | asseqfrequencyAAT | 0.00066627 | The frequency of AAT in AS region sequence |
| 89 | asseqfrequencyAAC | 0.00065229 | The frequency of AAC in AS region sequence |
| 90 | asseqfrequencyAAG | 0.00130101 | The frequency of AAG in AS region sequence |
| 91 | asseqfrequencyAT | 0.00158739 | The frequency of AT in AS region sequence |
| 92 | asseqfrequencyATA | 0.00056893 | The frequency of ATA in AS region sequence |
| 93 | asseqfrequencyATT | 0.0006019 | The frequency of ATT in AS region sequence |
| 94 | asseqfrequencyATC | 0.00076242 | The frequency of ATC in AS region sequence |
| 95 | asseqfrequencyATG | 0.00077056 | The frequency of ATG in AS region sequence |
| 96 | asseqfrequencyAC | 0.00118974 | The frequency of AC in AS region sequence |
| 97 | asseqfrequencyACA | 0.00096529 | The frequency of ACA in AS region sequence |
| 98 | asseqfrequencyACT | 0.00061111 | The frequency of ACT in AS region sequence |
| 99 | asseqfrequencyACC | 0.00053821 | The frequency of ACC in AS region sequence |
| 100 | asseqfrequencyACG | 0.00055502 | The frequency of ACG in AS region sequence |
| 101 | asseqfrequencyAG | 0.0028336 | The frequency of AG in AS region sequence |
| 102 | asseqfrequencyAGA | 0.00169715 | The frequency of AGA in AS region sequence |
| 103 | asseqfrequencyAGT | 0.00094007 | The frequency of AGT in AS region sequence |
| 104 | asseqfrequencyAGC | 0.00098379 | The frequency of AGC in AS region sequence |
| 105 | asseqfrequencyAGG | 0.0015495 | The frequency of AGG in AS region sequence |
| 106 | asseqfrequencyT | 0.00200717 | The frequency of T in AS region sequence |
| 107 | asseqfrequencyTA | 0.00125172 | The frequency of TA in AS region sequence |
| 108 | asseqfrequencyTAA | 0.00063381 | The frequency of TAA in AS region sequence |
| 109 | asseqfrequencyTAT | 0.00056674 | The frequency of TAT in AS region sequence |
| 110 | asseqfrequencyTAC | 0.00060683 | The frequency of TAC in AS region sequence |
| 111 | asseqfrequencyTAG | 0.00067511 | The frequency of TAG in AS region sequence |
| 112 | asseqfrequencyTT | 0.00147658 | The frequency of TT in AS region sequence |
| 113 | asseqfrequencyTTA | 0.00055822 | The frequency of TTA in AS region sequence |
| 114 | asseqfrequencyTTT | 0.0011774 | The frequency of TTT in AS region sequence |
| 115 | asseqfrequencyTTC | 0.00084713 | The frequency of TTC in AS region sequence |
| 116 | asseqfrequencyTTG | 0.00057696 | The frequency of TTG in AS region sequence |
| 117 | asseqfrequencyTC | 0.00113531 | The frequency of TC in AS region sequence |
| 118 | asseqfrequencyTCA | 0.0012888 | The frequency of TCA in AS region sequence |
| 119 | asseqfrequencyTCT | 0.000873 | The frequency of TCT in AS region sequence |
| 120 | asseqfrequencyTCC | 0.0008606 | The frequency of TCC in AS region sequence |
| 121 | asseqfrequencyTCG | 0.00052793 | The frequency of TCG in AS region sequence |
| 122 | asseqfrequencyTG | 0.00084508 | The frequency of TG in AS region sequence |
| 123 | asseqfrequencyTGA | 0.00085939 | The frequency of TGA in AS region sequence |
| 124 | asseqfrequencyTGT | 0.00097791 | The frequency of TGT in AS region sequence |
| 125 | asseqfrequencyTGC | 0.00076763 | The frequency of TGC in AS region sequence |
| 126 | asseqfrequencyTGG | 0.00084692 | The frequency of TGG in AS region sequence |
| 127 | asseqfrequencyC | 0.00157183 | The frequency of C in AS region sequence |
| 128 | asseqfrequencyCA | 0.00265721 | The frequency of CA in AS region sequence |
| 129 | asseqfrequencyCAA | 0.00124177 | The frequency of CAA in AS region sequence |
| 130 | asseqfrequencyCAT | 0.00069481 | The frequency of CAT in AS region sequence |
| 131 | asseqfrequencyCAC | 0.00064456 | The frequency of CAC in AS region sequence |
| 132 | asseqfrequencyCAG | 0.00254851 | The frequency of CAG in AS region sequence |
| 133 | asseqfrequencyCT | 0.00102412 | The frequency of CT in AS region sequence |
| 134 | asseqfrequencyCTT | 0.00082232 | The frequency of CTT in AS region sequence |
| 135 | asseqfrequencyCTC | 0.00065311 | The frequency of CTC in AS region sequence |
| 136 | asseqfrequencyCTG | 0.00069029 | The frequency of CTG in AS region sequence |
| 137 | asseqfrequencyCC | 0.00125255 | The frequency of CC in AS region sequence |
| 138 | asseqfrequencyCCA | 0.00066105 | The frequency of CCA in AS region sequence |
| 139 | asseqfrequencyCCT | 0.00098925 | The frequency of CCT in AS region sequence |
| 140 | asseqfrequencyCCC | 0.00127424 | The frequency of CCC in AS region sequence |
| 141 | asseqfrequencyCCG | 0.00081053 | The frequency of CCG in AS region sequence |
| 142 | asseqfrequencyCG | 0.00158243 | The frequency of CG in AS region sequence |
| 143 | asseqfrequencyCGA | 0.00062279 | The frequency of CGA in AS region sequence |
| 144 | asseqfrequencyCGC | 0.00062939 | The frequency of CGC in AS region sequence |
| 145 | asseqfrequencyCGG | 0.00114093 | The frequency of CGG in AS region sequence |
| 146 | asseqfrequencyG | 0.00352234 | The frequency of G in AS region sequence |
| 147 | asseqfrequencyGA | 0.00239326 | The frequency of GA in AS region sequence |
| 148 | asseqfrequencyGAA | 0.00104029 | The frequency of GAA in AS region sequence |
| 149 | asseqfrequencyGAT | 0.00075181 | The frequency of GAT in AS region sequence |
| 150 | asseqfrequencyGAC | 0.00066078 | The frequency of GAC in AS region sequence |
| 151 | asseqfrequencyGAG | 0.00106982 | The frequency of GAG in AS region sequence |
| 152 | asseqfrequencyGT | 0.00298577 | The frequency of GT in AS region sequence |
| 153 | asseqfrequencyGTA | 0.00115602 | The frequency of GTA in AS region sequence |
| 154 | asseqfrequencyGTT | 0.00055317 | The frequency of GTT in AS region sequence |
| 155 | asseqfrequencyGTC | 0.00056694 | The frequency of GTC in AS region sequence |
| 156 | asseqfrequencyGTG | 0.00132808 | The frequency of GTG in AS region sequence |
| 157 | asseqfrequencyGC | 0.00095184 | The frequency of GC in AS region sequence |
| 158 | asseqfrequencyGCA | 0.00073113 | The frequency of GCA in AS region sequence |
| 159 | asseqfrequencyGCT | 0.00055435 | The frequency of GCT in AS region sequence |
| 160 | asseqfrequencyGCC | 0.00066351 | The frequency of GCC in AS region sequence |
| 161 | asseqfrequencyGCG | 0.00093908 | The frequency of GCG in AS region sequence |
| 162 | asseqfrequencyGG | 0.00222462 | The frequency of GG in AS region sequence |
| 163 | asseqfrequencyGGA | 0.00122948 | The frequency of GGA in AS region sequence |
| 164 | asseqfrequencyGGT | 0.00081454 | The frequency of GGT in AS region sequence |
| 165 | asseqfrequencyGGC | 0.00117968 | The frequency of GGC in AS region sequence |
| 166 | asseqfrequencyGGG | 0.00267595 | The frequency of GGG in AS region sequence |
| 167 | asseqdistr1%A | 0.00411731 | The distribution(position/length) of 1%A in AS region sequence |
| 168 | asseqdistr25%A | 0.00606579 | The distribution(position/length) of 25%A in AS region sequence |
| 169 | asseqdistr50%A | 0.00183051 | The distribution(position/length) of 50%A in AS region sequence |
| 170 | asseqdistr75%A | 0.00264277 | The distribution(position/length) of 75%A in AS region sequence |
| 171 | asseqdistr100%A | 0.00284182 | The distribution(position/length) of 100%A in AS region sequence |
| 172 | asseqdistr1%T | 0.00188463 | The distribution(position/length) of 1%T in AS region sequence |
| 173 | asseqdistr25%T | 0.00151007 | The distribution(position/length) of 25%T in AS region sequence |
| 174 | asseqdistr50%T | 0.00237935 | The distribution(position/length) of 50%T in AS region sequence |
| 175 | asseqdistr75%T | 0.00290814 | The distribution(position/length) of 75%T in AS region sequence |
| 176 | asseqdistr100%T | 0.00607481 | The distribution(position/length) of 100%T in AS region sequence |
| 177 | asseqdistr1%C | 0.00244276 | The distribution(position/length) of 1%C in AS region sequence |
| 178 | asseqdistr25%C | 0.00142548 | The distribution(position/length) of 25%C in AS region sequence |
| 179 | asseqdistr50%C | 0.00135641 | The distribution(position/length) of 50%C in AS region sequence |
| 180 | asseqdistr75%C | 0.00293903 | The distribution(position/length) of 75%C in AS region sequence |
| 181 | asseqdistr100%C | 0.00404454 | The distribution(position/length) of 100%C in AS region sequence |
| 182 | asseqdistr1%G | 0.00910389 | The distribution(position/length) of 1%G in AS region sequence |
| 183 | asseqdistr25%G | 0.00879149 | The distribution(position/length) of 25%G in AS region sequence |
| 184 | asseqdistr50%G | 0.004301 | The distribution(position/length) of 50%G in AS region sequence |
| 185 | asseqdistr75%G | 0.00253368 | The distribution(position/length) of 75%G in AS region sequence |
| 186 | asseqdistr100%G | 0.00106038 | The distribution(position/length) of 100%G in AS region sequence |
| 187 | asseqdonerGT | 0.00077499 | Is there GT in doner of AS region sequence (1 for yes, 0 for no) |
| 188 | asseqacceptorGT | 0.0232506 | Is there GT in acceptor of AS region sequence (1 for yes, 0 for no) |
| 189 | asseqdonerGC | 0.00053073 | Is there GC in doner of AS region sequence (1 for yes, 0 for no) |
| 190 | asseqacceptorGC | 0.00103237 | Is there GC in acceptor of AS region sequence (1 for yes, 0 for no) |
| 191 | asseqdonerAT | 0.00087271 | Is there AT in doner of AS region sequence (1 for yes, 0 for no) |
| 192 | asseqacceptorAT | 0.0011296 | Is there AT in acceptor of AS region sequence (1 for yes, 0 for no) |
| 193 | asseqdonerAG | 0.00848124 | Is there AG in doner of AS region sequence (1 for yes, 0 for no) |
| 194 | asseqacceptorAG | 0.00450759 | Is there AG in acceptor of AS region sequence (1 for yes, 0 for no) |
| 195 | asseqacceptorAC | 0.00057082 | Is there AC in acceptor of AS region sequence (1 for yes, 0 for no) |
| 196 | updistr100%A | 0.00050752 | The distribution(position/length) of 100%A in upstream sequence |
| 197 | updistr100%G | 0.00156791 | The distribution(position/length) of 100%G in upstream sequence |
| 198 | updonerAG | 0.00112393 | Is AG in doner of upstream sequence (1 for yes, 0 for no) |
| 199 | downGC | 0.00129222 | The GC contant of downstream sequence |
| 200 | downfrequencyAT | 0.00075052 | The frequency of AT in downstream sequence |
| 201 | downfrequencyT | 0.00055035 | The frequency of T in downstream sequence |
| 202 | downfrequencyCCG | 0.00058042 | The frequency of CCG in downstream sequence |
| 203 | downfrequencyCG | 0.00116404 | The frequency of CG in downstream sequence |
| 204 | downfrequencyCGC | 0.00057317 | The frequency of CGC in downstream sequence |
| 205 | downfrequencyCGG | 0.00059605 | The frequency of CGG in downstream sequence |
| 206 | downfrequencyG | 0.00052572 | The frequency of G in downstream sequence |
| 207 | downdistr100%G | 0.00063384 | The distribution(position/length) of 100%G in downstream sequence |
| 208 | downdonerAG | 0.00062139 | Is there AG in doner of downstream sequence (1 for yes, 0 for no) |
| 209 | up30as30if%3 | 0.00474754 | Whethere the length of upstream30 + AS 30bp sequence divisible by three |
| 210 | up30as30GC | 0.00097856 | The GC contant of upstream30AS 30bp + AS 30bp sequence |
| 211 | up30as30frequencyAT | 0.00059268 | The frequency of AT in upstream 30 bp + AS 30bp sequence |
| 212 | up30as30frequencyAGG | 0.00050739 | The frequency of AGG in upstream 30 bp + AS 30bp sequence |
| 213 | up30as30frequencyTAA | 0.00051832 | The frequency of TAA in upstream 30 bp + AS 30bp sequence |
| 214 | up30as30frequencyCA | 0.00054439 | The frequency of CA in upstream 30 bp + AS 30bp sequence |
| 215 | up30as30frequencyCG | 0.0005249 | The frequency of CG in upstream 30 bp + AS 30bp sequence |
| 216 | up30as30frequencyG | 0.00090055 | The frequency of G in upstream 30 bp + AS 30bp sequence |
| 217 | up30as30frequencyGT | 0.00100878 | The frequency of GT in upstream 30 bp + AS 30bp sequence |
| 218 | up30as30frequencyGTA | 0.00186856 | The frequency of GTA in upstream 30 bp + AS 30bp sequence |
| 219 | up30as30frequencyGTG | 0.00104536 | The frequency of GTG in upstream 30 bp + AS 30bp sequence |
| 220 | up30as30frequencyGG | 0.00068261 | The frequency of GG in upstream 30 bp + AS 30bp sequence |
| 221 | up30as30frequencyGGT | 0.00203348 | The frequency of GGT in upstream 30 bp + AS 30bp sequence |
| 222 | up30as30frequencyGGG | 0.00101225 | The frequency of GGG in upstream 30 bp + AS 30bp sequence |
| 223 | up30as30distr50%A | 0.00057693 | The distribution(position/length) of 50%A in upstream 30 bp + AS 30bp sequence |
| 224 | up30as30distr75%A | 0.00065496 | The distribution(position/length) of 75%A in upstream 30 bp + AS 30bp sequence |
| 225 | up30as30distr100%A | 0.00079364 | The distribution(position/length) of 100%A in upstream 30 bp + AS 30bp sequence |
| 226 | up30as30distr75%T | 0.00076725 | The distribution(position/length) of 75%T in upstream 30 bp + AS 30bp sequence |
| 227 | up30as30distr100%T | 0.00055656 | The distribution(position/length) of 100%T in upstream 30 bp + AS 30bp sequence |
| 228 | up30as30distr50%G | 0.00061018 | The distribution(position/length) of 50%G in upstream 30 bp + AS 30bp sequence |
| 229 | up30as30distr75%G | 0.00056901 | The distribution(position/length) of 75%G in upstream 30 bp + AS 30bp sequence |
| 230 | up30as30distr100%G | 0.00069336 | The distribution(position/length) of 100%G in upstream 30 bp + AS 30bp sequence |
| 231 | up30as30donerGT | 0.00055622 | Is there GT in doner of upstream 30 bp + AS 30bp sequence (1 for yes, 0 for no) |
| 232 | up30as30donerAG | 0.00164825 | Is there AG in doner of upstream 30 bp + AS 30bp sequence (1 for yes, 0 for no) |
| 233 | up30down30GC | 0.00052924 | The GC contant of upstream 30 + downstream 30GC sequence |
| 234 | as30down30if%3 | 0.00650359 | Whethere the length of AS 30bp + downstream 30bp sequence divisible by three |
| 235 | as30down30GC | 0.00137061 | The GC contant of AS 30bp + downstream 30bp sequence |
| 236 | as30down30frequencyA | 0.00248632 | The frequency of A in AS 30bp + downstream 30bp sequence |
| 237 | as30down30frequencyAA | 0.00118563 | The frequency of AA in AS 30bp + downstream 30bp sequence |
| 238 | as30down30frequencyAAA | 0.00069696 | The frequency of AAA in AS 30bp + downstream 30bp sequence |
| 239 | as30down30frequencyAAT | 0.00051676 | The frequency of AAT in AS 30bp + downstream 30bp sequence |
| 240 | as30down30frequencyAAG | 0.00085099 | The frequency of AAG in AS 30bp + downstream 30bp sequence |
| 241 | as30down30frequencyAT | 0.00061518 | The frequency of AT in AS 30bp + downstream 30bp sequence |
| 242 | as30down30frequencyAG | 0.0007348 | The frequency of AG in AS 30bp + downstream 30bp sequence |
| 243 | as30down30frequencyT | 0.00096321 | The frequency of T in AS 30bp + downstream 30bp sequence |
| 244 | as30down30frequencyTT | 0.00078277 | The frequency of TT in AS 30bp + downstream 30bp sequence |
| 245 | as30down30frequencyTTT | 0.00072603 | The frequency of TTT in AS 30bp + downstream 30bp sequence |
| 246 | as30down30frequencyTTC | 0.00069752 | The frequency of TTC in AS 30bp + downstream 30bp sequence |
| 247 | as30down30frequencyTC | 0.00114611 | The frequency of TC in AS 30bp + downstream 30bp sequence |
| 248 | as30down30frequencyTCT | 0.00088733 | The frequency of TCT in AS 30bp + downstream 30bp sequence |
| 249 | as30down30frequencyTCC | 0.00068028 | The frequency of TCC in AS 30bp + downstream 30bp sequence |
| 250 | as30down30frequencyTGT | 0.00050917 | The frequency of TGT in AS 30bp + downstream 30bp sequence |
| 251 | as30down30frequencyC | 0.0015108 | The frequency of C in AS 30bp + downstream 30bp sequence |
| 252 | as30down30frequencyCAA | 0.00057239 | The frequency of CAA in AS 30bp + downstream 30bp sequence |
| 253 | as30down30frequencyCT | 0.00100102 | The frequency of CT in AS 30bp + downstream 30bp sequence |
| 254 | as30down30frequencyCTT | 0.00062108 | The frequency of CTT in AS 30bp + downstream 30bp sequence |
| 255 | as30down30frequencyCTC | 0.00068795 | The frequency of CTC in AS 30bp + downstream 30bp sequence |
| 256 | as30down30frequencyCC | 0.00124744 | The frequency of CC in AS 30bp + downstream 30bp sequence |
| 257 | as30down30frequencyCCT | 0.00069083 | The frequency of CCT in AS 30bp + downstream 30bp sequence |
| 258 | as30down30frequencyCCC | 0.00112537 | The frequency of CCC in AS 30bp + downstream 30bp sequence |
| 259 | as30down30frequencyCG | 0.00112878 | The frequency of CG in AS 30bp + downstream 30bp sequence |
| 260 | as30down30frequencyCGC | 0.00057624 | The frequency of CGC in AS 30bp + downstream 30bp sequence |
| 261 | as30down30frequencyCGG | 0.00063691 | The frequency of CGG in AS 30bp + downstream 30bp sequence |
| 262 | as30down30frequencyG | 0.00119789 | The frequency of G in AS 30bp + downstream 30bp sequence |
| 263 | as30down30frequencyGA | 0.0007453 | The frequency of GA in AS 30bp + downstream 30bp sequence |
| 264 | as30down30frequencyGAA | 0.000782 | The frequency of GAA in AS 30bp + downstream 30bp sequence |
| 265 | as30down30frequencyGAG | 0.00055691 | The frequency of GAG in AS 30bp + downstream 30bp sequence |
| 266 | as30down30frequencyGC | 0.00059211 | The frequency of GC in AS 30bp + downstream 30bp sequence |
| 267 | as30down30frequencyGCG | 0.00084407 | The frequency of GCG in AS 30bp + downstream 30bp sequence |
| 268 | as30down30frequencyGG | 0.00070159 | The frequency of GG in AS 30bp + downstream 30bp sequence |
| 269 | as30down30frequencyGGA | 0.00054978 | The frequency of GGA in AS 30bp + downstream 30bp sequence |
| 270 | as30down30frequencyGGC | 0.0005576 | The frequency of GGC in AS 30bp + downstream 30bp sequence |
| 271 | as30down30frequencyGGG | 0.00068282 | The frequency of GGG in AS 30bp + downstream 30bp sequence |
| 272 | as30down30distr1%A | 0.00066735 | The distribution(position/length) of 1%A in AS 30bp + downstream 30bp sequence |
| 273 | as30down30distr25%A | 0.00076062 | The distribution(position/length) of 25%A in AS 30bp + downstream 30bp sequence |
| 274 | as30down30distr50%A | 0.0007277 | The distribution(position/length) of 50%A in AS 30bp + downstream 30bp sequence |
| 275 | as30down30distr75%A | 0.00051167 | The distribution(position/length) of 75%A in AS 30bp + downstream 30bp sequence |
| 276 | as30down30distr1%T | 0.00071621 | The distribution(position/length) of 1%T in AS 30bp + downstream 30bp sequence |
| 277 | as30down30distr50%T | 0.00263898 | The distribution(position/length) of 50%T in AS 30bp + downstream 30bp sequence |
| 278 | as30down30distr75%T | 0.0010547 | The distribution(position/length) of 75%T in AS 30bp + downstream 30bp sequence |
| 279 | as30down30distr1%C | 0.00056924 | The distribution(position/length) of 1%C in AS 30bp + downstream 30bp sequence |
| 280 | as30down30distr25%C | 0.00051178 | The distribution(position/length) of 25%C in AS 30bp + downstream 30bp sequence |
| 281 | as30down30distr50%C | 0.00052881 | The distribution(position/length) of 50%C in AS 30bp + downstream 30bp sequence |
| 282 | as30down30distr1%G | 0.00118134 | The distribution(position/length) of 1%G in AS 30bp + downstream 30bp sequence |
| 283 | as30down30distr25%G | 0.00084032 | The distribution(position/length) of 25%G in AS 30bp + downstream 30bp sequence |
| 284 | as30down30distr50%G | 0.00153808 | The distribution(position/length) of 50%G in AS 30bp + downstream 30bp sequence |
| 285 | as30down30distr75%G | 0.00058458 | The distribution(position/length) of 75%G in AS 30bp + downstream 30bp sequence |
| 286 | as30down30acceptorGT | 0.00180244 | Is there GT in acceptor of AS 30bp + downstream 30bp sequence (1 for yes, 0 for no) |
| 287 | up50as50if%3 | 0.00217258 | Whethere the length of upstream 50bp + AS 50bp sequence divisible by three |
| 288 | up50as50GC | 0.00161372 | The GC contant of upstream 50bp + AS 50bp sequence |
| 289 | up50as50numberTGA | 0.00051969 | The numberof stopdocon TGA in upstream 50bp + AS 50bp sequence |
| 290 | up50as50frequencyA | 0.00073539 | The frequency of A in upstream 50bp + AS 50bp sequence |
| 291 | up50as50frequencyAA | 0.0005253 | The frequency of AA in upstream 50bp + AS 50bp sequence |
| 292 | up50as50frequencyAAA | 0.00054986 | The frequency of AAA in upstream 50bp + AS 50bp sequence |
| 293 | up50as50frequencyAT | 0.00050509 | The frequency of AT in upstream 50bp + AS 50bp sequence |
| 294 | up50as50frequencyAGG | 0.00053668 | The frequency of AGG in upstream 50bp + AS 50bp sequence |
| 295 | up50as50frequencyCA | 0.00063607 | The frequency of CA in upstream 50bp + AS 50bp sequence |
| 296 | up50as50frequencyCC | 0.00054102 | The frequency of CC in upstream 50bp + AS 50bp sequence |
| 297 | up50as50frequencyCCT | 0.0005109 | The frequency of CCT in upstream 50bp + AS 50bp sequence |
| 298 | up50as50frequencyCCC | 0.00054381 | The frequency of CCC in upstream 50bp + AS 50bp sequence |
| 299 | up50as50frequencyCG | 0.0006102 | The frequency of CG in upstream 50bp + AS 50bp sequence |
| 300 | up50as50frequencyG | 0.0008402 | The frequency of G in upstream 50bp + AS 50bp sequence |
| 301 | up50as50frequencyGT | 0.00064936 | The frequency of GT in upstream 50bp + AS 50bp sequence |
| 302 | up50as50frequencyGTA | 0.00108651 | The frequency of GTA in upstream 50bp + AS 50bp sequence |
| 303 | up50as50frequencyGTG | 0.00062282 | The frequency of GTG in upstream 50bp + AS 50bp sequence |
| 304 | up50as50frequencyGG | 0.00088383 | The frequency of GG in upstream 50bp + AS 50bp sequence |
| 305 | up50as50frequencyGGT | 0.00109505 | The frequency of GGT in upstream 50bp + AS 50bp sequence |
| 306 | up50as50frequencyGGG | 0.00142172 | The frequency of GGG in upstream 50bp + AS 50bp sequence |
| 307 | up50as50distr25%A | 0.00052063 | The distribution(position/length) of 25%A in upstream 50bp + AS 50bp sequence |
| 308 | up50as50distr50%A | 0.00074426 | The distribution(position/length) of 50%A in upstream 50bp + AS 50bp sequence |
| 309 | up50as50distr75%A | 0.00075071 | The distribution(position/length) of 75%A in upstream 50bp + AS 50bp sequence |
| 310 | up50as50distr100%A | 0.00089748 | The distribution(position/length) of 100%A in upstream 50bp + AS 50bp sequence |
| 311 | up50as50distr50%T | 0.00061052 | The distribution(position/length) of 50%T in upstream 50bp + AS 50bp sequence |
| 312 | up50as50distr75%T | 0.00067725 | The distribution(position/length) of 75%T in upstream 50bp + AS 50bp sequence |
| 313 | up50as50distr100%T | 0.00060157 | The distribution(position/length) of 100%T in upstream 50bp + AS 50bp sequence |
| 314 | up50as50distr100%C | 0.00062694 | The distribution(position/length) of 100%C in upstream 50bp + AS 50bp sequence |
| 315 | up50as50distr25%G | 0.00051214 | The distribution(position/length) of 25%G in upstream 50bp + AS 50bp sequence |
| 316 | up50as50distr50%G | 0.00074642 | The distribution(position/length) of 50%G in upstream 50bp + AS 50bp sequence |
| 317 | up50as50distr75%G | 0.00067369 | The distribution(position/length) of 75%G in upstream 50bp + AS 50bp sequence |
| 318 | up50as50distr100%G | 0.0008374 | The distribution(position/length) of 100%G in upstream 50bp + AS 50bp sequence |
| 319 | up50as50donerGT | 0.00059529 | Is there GT in doner of upstream 50bp + AS 50bp sequence (1 for yes, 0 for no) |
| 320 | up50as50donerAG | 0.00252162 | Is there AG in doner of upstream 50bp + AS 50bp sequence (1 for yes, 0 for no) |
| 321 | up50down50GC | 0.00054293 | The GC contant upstream 50bp + + downstream 50bp sequence |
| 322 | up50down50distr100%G | 0.00068183 | The distribution(position/length) of 100%G in upstream 50bp + downstream 50bp sequence |
| 323 | up50down50donerAG | 0.00064568 | Is there AG in doner of upstream 50bp + downstream 50bp sequence (1 for yes, 0 for no) |
| 324 | as50down50if%3 | 0.00219184 | Whethere the length of AS 50bp + downstream 50bp sequence divisible by three |
| 325 | as50down50GC | 0.00183917 | The GC contant of GC in AS 50bp + downstream 50bp sequence |
| 326 | as50down50numberTAA | 0.00050581 | The number of stopdocon TAA in AS 50bp + downstream 50bp sequence |
| 327 | as50down50numberTGA | 0.0006772 | The number of stopdocon TGA in AS 50bp + downstream 50bp sequence |
| 328 | as50down50frequencyA | 0.00170553 | The frequency of A in AS 50bp + downstream 50bp sequence |
| 329 | as50down50frequencyAA | 0.00095836 | The frequency of AA in AS 50bp + downstream 50bp sequence |
| 330 | as50down50frequencyAAA | 0.00094627 | The frequency of AAA in AS 50bp + downstream 50bp sequence |
| 331 | as50down50frequencyAAG | 0.00079716 | The frequency of AAG in AS 50bp + downstream 50bp sequence |
| 332 | as50down50frequencyAT | 0.00081654 | The frequency of AT in AS 50bp + downstream 50bp sequence |
| 333 | as50down50frequencyATT | 0.00055137 | The frequency of ATT in AS 50bp + downstream 50bp sequence |
| 334 | as50down50frequencyAG | 0.00058994 | The frequency of AG in AS 50bp + downstream 50bp sequence |
| 335 | as50down50frequencyAGA | 0.00052927 | The frequency of AGA in AS 50bp + downstream 50bp sequence |
| 336 | as50down50frequencyT | 0.00079415 | The frequency of T in AS 50bp + downstream 50bp sequence |
| 337 | as50down50frequencyTT | 0.00059693 | The frequency of TT in AS 50bp + downstream 50bp sequence |
| 338 | as50down50frequencyTTT | 0.00068869 | The frequency of TTT in AS 50bp + downstream 50bp sequence |
| 339 | as50down50frequencyTTC | 0.00052754 | The frequency of TTC in AS 50bp + downstream 50bp sequence |
| 340 | as50down50frequencyTC | 0.00062893 | The frequency of TC in AS 50bp + downstream 50bp sequence |
| 341 | as50down50frequencyTCT | 0.00061056 | The frequency of TCT in AS 50bp + downstream 50bp sequence |
| 342 | as50down50frequencyTCC | 0.00058486 | The frequency of TCC in AS 50bp + downstream 50bp sequence |
| 343 | as50down50frequencyC | 0.00108165 | The frequency of C in AS 50bp + downstream 50bp sequence |
| 344 | as50down50frequencyCAA | 0.00084815 | The frequency of CAA in AS 50bp + downstream 50bp sequence |
| 345 | as50down50frequencyCT | 0.000901 | The frequency of CT in AS 50bp + downstream 50bp sequence |
| 346 | as50down50frequencyCTT | 0.000519 | The frequency of CTT in AS 50bp + downstream 50bp sequence |
| 347 | as50down50frequencyCTC | 0.00057286 | The frequency of CTC in AS 50bp + downstream 50bp sequence |
| 348 | as50down50frequencyCC | 0.0008822 | The frequency of CC in AS 50bp + downstream 50bp sequence |
| 349 | as50down50frequencyCCT | 0.00066117 | The frequency of CCT in AS 50bp + downstream 50bp sequence |
| 350 | as50down50frequencyCCC | 0.00123427 | The frequency of CCC in AS 50bp + downstream 50bp sequence |
| 351 | as50down50frequencyCCG | 0.00064359 | The frequency of CCG in AS 50bp + downstream 50bp sequence |
| 352 | as50down50frequencyCG | 0.00149152 | The frequency of CG in AS 50bp + downstream 50bp sequence |
| 353 | as50down50frequencyCGC | 0.00062161 | The frequency of CGC in AS 50bp + downstream 50bp sequence |
| 354 | as50down50frequencyCGG | 0.0009457 | The frequency of CGG in AS 50bp + downstream 50bp sequence |
| 355 | as50down50frequencyG | 0.00090804 | The frequency of G in AS 50bp + downstream 50bp sequence |
| 356 | as50down50frequencyGA | 0.0008211 | The frequency of GA in AS 50bp + downstream 50bp sequence |
| 357 | as50down50frequencyGAA | 0.00077229 | The frequency of GAA in AS 50bp + downstream 50bp sequence |
| 358 | as50down50frequencyGC | 0.00057992 | The frequency of GC in AS 50bp + downstream 50bp sequence |
| 359 | as50down50frequencyGCG | 0.00090251 | The frequency of GCG in AS 50bp + downstream 50bp sequence |
| 360 | as50down50frequencyGG | 0.00058702 | The frequency of GG in AS 50bp + downstream 50bp sequence |
| 361 | as50down50frequencyGGG | 0.00083036 | The frequency of GGG in AS 50bp + downstream 50bp sequence |
| 362 | as50down50distr1%A | 0.00076284 | The distribution(position/length) of 1%A in AS 50bp + downstream 50bp sequence |
| 363 | as50down50distr50%A | 0.00066968 | The distribution(position/length) of 50%A in AS 50bp + downstream 50bp sequence |
| 364 | as50down50distr1%T | 0.00078609 | The distribution(position/length) of 1%T in AS 50bp + downstream 50bp sequence |
| 365 | as50down50distr50%T | 0.00128706 | The distribution(position/length) of 50%T in AS 50bp + downstream 50bp sequence |
| 366 | as50down50distr75%T | 0.00079896 | The distribution(position/length) of 75%T in AS 50bp + downstream 50bp sequence |
| 367 | as50down50distr1%C | 0.00070529 | The distribution(position/length) of 1%C in AS 50bp + downstream 50bp sequence |
| 368 | as50down50distr1%G | 0.00118752 | The distribution(position/length) of 1%G in AS 50bp + downstream 50bp sequence |
| 369 | as50down50distr25%G | 0.00074848 | The distribution(position/length) of 25%G in AS 50bp + downstream 50bp sequence |
| 370 | as50down50distr50%G | 0.00079151 | The distribution(position/length) of 50%G in AS 50bp + downstream 50bp sequence |
| 371 | as50down50distr100%G | 0.00058932 | The distribution(position/length) of 100%G in AS 50bp + downstream 50bp sequence |
| 372 | as50down50acceptorGT | 0.00320036 | Is there GT in acceptor of AS 50bp + downstream 50bp sequence (1 for yes, 0 for no) |
| 373 | as50down50donerAG | 0.00065088 | Is there AG in doner of AS 50bp + downstream 50bp sequence (1 for yes, 0 for no) |
| 374 | as50down50acceptorAG | 0.00065056 | Is there AG in acceptor of AS 50bp + downstream 50bp sequence (1 for yes, 0 for no) |
| 375 | down20bpGC | 0.00064652 | The GC contant of downstream 20bp sequence |
| 376 | down20bpfrequencyCG | 0.00064071 | The frequency of CG in downstream 20bp sequence |

Table S7 Features of the *Arabidopsis thaliana* model

| No. | Features | Importance | Description |
| --- | --- | --- | --- |
| 1 | length_of_as | 0.0031287 | Length of AS region |
| 2 | DmotifTTCTT | 0.00090826 | Is there this motif in downstream of splicing site: TTCTT (1 for yes, 0 for no) |
| 3 | DmotifTAACT | 0.0005252 | Is there this motif in downstream of splicing site: TAACT (1 for yes, 0 for no) |
| 4 | DmotifTCTTT | 0.00086944 | Is there this motif in downstream of splicing site: TCTTT (1 for yes, 0 for no) |
| 5 | DmotifTCTGG | 0.00050688 | Is there this motif in downstream of splicing site: TCTGG (1 for yes, 0 for no) |
| 6 | DmotifGTAAG | 0.00074236 | Is there this motif in downstream of splicing site: GTAAG (1 for yes, 0 for no) |
| 7 | DmotifGTTTT | 0.00120458 | Is there this motif in downstream of splicing site: GTTTT (1 for yes, 0 for no) |
| 8 | DmotifGTAAT | 0.00055544 | Is there this motif in downstream of splicing site: GTAAT (1 for yes, 0 for no) |
| 9 | DmotifTTCTCT | 0.00052963 | Is there this motif in downstream of splicing site: TTCTCT (1 for yes, 0 for no) |
| 10 | DmotifTATGT | 0.00129761 | Is there this motif in downstream of splicing site: TATGT (1 for yes, 0 for no) |
| 11 | DmotifTTTCTC | 0.00052936 | Is there this motif in downstream of splicing site: TTTCTC (1 for yes, 0 for no) |
| 12 | DmotifCTTTT | 0.00175439 | Is there this motif in downstream of splicing site: CTTTT (1 for yes, 0 for no) |
| 13 | DmotifTTTAG | 0.0006886 | Is there this motif in downstream of splicing site: TTTAG (1 for yes, 0 for no) |
| 14 | DmotifTTTTTC | 0.00077446 | Is there this motif in downstream of splicing site: TTTTTC (1 for yes, 0 for no) |
| 15 | DmotifTCTTG | 0.00056064 | Is there this motif in downstream of splicing site: TCTTG (1 for yes, 0 for no) |
| 16 | DmotifTCTTC | 0.00055469 | Is there this motif in downstream of splicing site: TCTTC (1 for yes, 0 for no) |
| 17 | UmotifTTCTT | 0.00099871 | Is there this motif in upstream of splicing site: TTCTT (1 for yes, 0 for no) |
| 18 | UmotifTCTTT | 0.00168493 | Is there this motif in upstream of splicing site: TCTTT (1 for yes, 0 for no) |
| 19 | UmotifCTCTG | 0.00054215 | Is there this motif in upstream of splicing site: CTCTG (1 for yes, 0 for no) |
| 20 | UmotifGTAAG | 0.00091884 | Is there this motif in upstream of splicing site: GTAAG (1 for yes, 0 for no) |
| 21 | UmotifGTTTT | 0.00129736 | Is there this motif in upstream of splicing site: GTTTT (1 for yes, 0 for no) |
| 22 | UmotifAAATT | 0.00053055 | Is there this motif in upstream of splicing site: AAATT (1 for yes, 0 for no) |
| 23 | UmotifTCTCT | 0.00069679 | Is there this motif in upstream of splicing site: TCTCT (1 for yes, 0 for no) |
| 24 | UmotifCTTTT | 0.00125088 | Is there this motif in upstream of splicing site: CTTTT (1 for yes, 0 for no) |
| 25 | UmotifTTCTC | 0.00050946 | Is there this motif in upstream of splicing site: TTCTC (1 for yes, 0 for no) |
| 26 | allseqGC | 0.00197553 | The GC contant of all sequence |
| 27 | allseqnumberTAA | 0.00093801 | The number of stopdocon TAA in all sequence |
| 28 | allseqnumberTAG | 0.00051914 | The number of stopdocon TAG in all sequence |
| 29 | allseqnumberTGA | 0.00079594 | The number of stopdocon TGA in all sequence |
| 30 | allseqfrequencyATA | 0.00054714 | The frequency of ATA in all sequence |
| 31 | allseqfrequencyAGA | 0.00056293 | The frequency of AGA in all sequence |
| 32 | allseqfrequencyT | 0.00120963 | The frequency of T in all sequence |
| 33 | allseqfrequencyTA | 0.00144838 | The frequency of TA in all sequence |
| 34 | allseqfrequencyTAA | 0.00086404 | The frequency of TAA in all sequence |
| 35 | allseqfrequencyTAT | 0.00055247 | The frequency of TAT in all sequence |
| 36 | allseqfrequencyTT | 0.00068956 | The frequency of TT in all sequence |
| 37 | allseqfrequencyTTA | 0.00065315 | The frequency of TTA in all sequence |
| 38 | allseqfrequencyTTT | 0.00102308 | The frequency of TTT in all sequence |
| 39 | allseqfrequencyCAG | 0.00080081 | The frequency of CAG in all sequence |
| 40 | allseqfrequencyCG | 0.00050537 | The frequency of CG in all sequence |
| 41 | allseqfrequencyG | 0.00064716 | The frequency of G in all sequence |
| 42 | allseqfrequencyGA | 0.00090113 | The frequency of GA in all sequence |
| 43 | allseqfrequencyGTA | 0.00082611 | The frequency of GTA in all sequence |
| 44 | allseqfrequencyGC | 0.00056062 | The frequency of GC in all sequence |
| 45 | allseqfrequencyGGA | 0.00072377 | The frequency of GGA in all sequence |
| 46 | allseqdistr1%A | 0.00050853 | The distribution(position/length) of 1% A in all sequence |
| 47 | allseqdistr25%T | 0.00075153 | The distribution(position/length) of 25% T in all sequence |
| 48 | allseqdistr50%T | 0.00061404 | The distribution(position/length) of 50% T in all sequence |
| 49 | allseqdistr75%T | 0.00062282 | The distribution(position/length) of 75% T in all sequence |
| 50 | allseqdistr25%C | 0.00053424 | The distribution(position/length) of 25% C in all sequence |
| 51 | allseqdistr50%C | 0.00078249 | The distribution(position/length) of 50% C in all sequence |
| 52 | allseqdistr1%G | 0.00053172 | The distribution(position/length) of 1% G in all sequence |
| 53 | allseqdistr25%G | 0.00054524 | The distribution(position/length) of 25% G in all sequence |
| 54 | allseqdistr50%G | 0.00058275 | The distribution(position/length) of 50% G in all sequence |
| 55 | allseqdistr75%G | 0.00100307 | The distribution(position/length) of 75% G in all sequence |
| 56 | allseqdistr100%G | 0.00058178 | The distribution(position/length) of 100% G in all sequence |
| 57 | asseqif%3 | 0.00075603 | Whethere the length of AS region sequence divisible by three |
| 58 | asseqGC | 0.00267662 | The GC contant of AS region sequence |
| 59 | asseqnumberTAA | 0.00154151 | The number of stopdocon TAA in AS region sequence |
| 60 | asseqnumberTAG | 0.00105079 | The number of stopdocon TAG in AS region sequence |
| 61 | asseqnumberTGA | 0.0013909 | The number of stopdocon TGA in AS region sequence |
| 62 | asseqfrequencyA | 0.0013278 | The frequency of A in AS region sequence |
| 63 | asseqfrequencyAA | 0.00189244 | The frequency of AA in AS region sequence |
| 64 | asseqfrequencyAAA | 0.00073439 | The frequency of AAA in AS region sequence |
| 65 | asseqfrequencyAAT | 0.0006585 | The frequency of AAT in AS region sequence |
| 66 | asseqfrequencyAAC | 0.00072175 | The frequency of AAC in AS region sequence |
| 67 | asseqfrequencyAAG | 0.00102058 | The frequency of AAG in AS region sequence |
| 68 | asseqfrequencyAT | 0.00071711 | The frequency of AT in AS region sequence |
| 69 | asseqfrequencyATA | 0.00074491 | The frequency of ATA in AS region sequence |
| 70 | asseqfrequencyATT | 0.00098504 | The frequency of ATT in AS region sequence |
| 71 | asseqfrequencyATC | 0.0007468 | The frequency of ATC in AS region sequence |
| 72 | asseqfrequencyATG | 0.00079964 | The frequency of ATG in AS region sequence |
| 73 | asseqfrequencyAC | 0.00132906 | The frequency of AC in AS region sequence |
| 74 | asseqfrequencyACA | 0.00084261 | The frequency of ACA in AS region sequence |
| 75 | asseqfrequencyACT | 0.00096637 | The frequency of ACT in AS region sequence |
| 76 | asseqfrequencyACG | 0.0005231 | The frequency of ACG in AS region sequence |
| 77 | asseqfrequencyAG | 0.00310413 | The frequency of AG in AS region sequence |
| 78 | asseqfrequencyAGA | 0.00105 | The frequency of AGA in AS region sequence |
| 79 | asseqfrequencyAGT | 0.00071315 | The frequency of AGT in AS region sequence |
| 80 | asseqfrequencyAGC | 0.00061105 | The frequency of AGC in AS region sequence |
| 81 | asseqfrequencyAGG | 0.00095123 | The frequency of AGG in AS region sequence |
| 82 | asseqfrequencyT | 0.0015526 | The frequency of T in AS region sequence |
| 83 | asseqfrequencyTA | 0.00210749 | The frequency of TA in AS region sequence |
| 84 | asseqfrequencyTAA | 0.00279647 | The frequency of TAA in AS region sequence |
| 85 | asseqfrequencyTAT | 0.00092955 | The frequency of TAT in AS region sequence |
| 86 | asseqfrequencyTAC | 0.00071859 | The frequency of TAC in AS region sequence |
| 87 | asseqfrequencyTAG | 0.00100277 | The frequency of TAG in AS region sequence |
| 88 | asseqfrequencyTT | 0.00325989 | The frequency of TT in AS region sequence |
| 89 | asseqfrequencyTTA | 0.00210937 | The frequency of TTA in AS region sequence |
| 90 | asseqfrequencyTTT | 0.00232819 | The frequency of TTT in AS region sequence |
| 91 | asseqfrequencyTTC | 0.00114105 | The frequency of TTC in AS region sequence |
| 92 | asseqfrequencyTTG | 0.00223335 | The frequency of TTG in AS region sequence |
| 93 | asseqfrequencyTC | 0.00106129 | The frequency of TC in AS region sequence |
| 94 | asseqfrequencyTCA | 0.00068795 | The frequency of TCA in AS region sequence |
| 95 | asseqfrequencyTCT | 0.00123544 | The frequency of TCT in AS region sequence |
| 96 | asseqfrequencyTCC | 0.00077003 | The frequency of TCC in AS region sequence |
| 97 | asseqfrequencyTCG | 0.00078591 | The frequency of TCG in AS region sequence |
| 98 | asseqfrequencyTG | 0.00172659 | The frequency of TG in AS region sequence |
| 99 | asseqfrequencyTGA | 0.00067501 | The frequency of TGA in AS region sequence |
| 100 | asseqfrequencyTGT | 0.00217143 | The frequency of TGT in AS region sequence |
| 101 | asseqfrequencyTGC | 0.00131095 | The frequency of TGC in AS region sequence |
| 102 | asseqfrequencyTGG | 0.00078633 | The frequency of TGG in AS region sequence |
| 103 | asseqfrequencyC | 0.00094733 | The frequency of C in AS region sequence |
| 104 | asseqfrequencyCA | 0.00177709 | The frequency of CA in AS region sequence |
| 105 | asseqfrequencyCAA | 0.00201401 | The frequency of CAA in AS region sequence |
| 106 | asseqfrequencyCAT | 0.00066235 | The frequency of CAT in AS region sequence |
| 107 | asseqfrequencyCAG | 0.00379374 | The frequency of CAG in AS region sequence |
| 108 | asseqfrequencyCT | 0.00460841 | The frequency of CT in AS region sequence |
| 109 | asseqfrequencyCTA | 0.00052012 | The frequency of CTA in AS region sequence |
| 110 | asseqfrequencyCTT | 0.00092103 | The frequency of CTT in AS region sequence |
| 111 | asseqfrequencyCTC | 0.00079791 | The frequency of CTC in AS region sequence |
| 112 | asseqfrequencyCTG | 0.00055332 | The frequency of CTG in AS region sequence |
| 113 | asseqfrequencyCC | 0.00059355 | The frequency of CC in AS region sequence |
| 114 | asseqfrequencyCCA | 0.00054019 | The frequency of CCA in AS region sequence |
| 115 | asseqfrequencyCCT | 0.00051827 | The frequency of CCT in AS region sequence |
| 116 | asseqfrequencyCG | 0.00099069 | The frequency of CG in AS region sequence |
| 117 | asseqfrequencyCGA | 0.00087573 | The frequency of CGA in AS region sequence |
| 118 | asseqfrequencyG | 0.0013486 | The frequency of G in AS region sequence |
| 119 | asseqfrequencyGA | 0.00160472 | The frequency of GA in AS region sequence |
| 120 | asseqfrequencyGAA | 0.00073121 | The frequency of GAA in AS region sequence |
| 121 | asseqfrequencyGAT | 0.00075458 | The frequency of GAT in AS region sequence |
| 122 | asseqfrequencyGAC | 0.00051875 | The frequency of GAC in AS region sequence |
| 123 | asseqfrequencyGAG | 0.00086106 | The frequency of GAG in AS region sequence |
| 124 | asseqfrequencyGT | 0.00295507 | The frequency of GT in AS region sequence |
| 125 | asseqfrequencyGTA | 0.00190334 | The frequency of GTA in AS region sequence |
| 126 | asseqfrequencyGTT | 0.00073332 | The frequency of GTT in AS region sequence |
| 127 | asseqfrequencyGTC | 0.00063169 | The frequency of GTC in AS region sequence |
| 128 | asseqfrequencyGTG | 0.00068685 | The frequency of GTG in AS region sequence |
| 129 | asseqfrequencyGC | 0.00111047 | The frequency of GC in AS region sequence |
| 130 | asseqfrequencyGCA | 0.00112206 | The frequency of GCA in AS region sequence |
| 131 | asseqfrequencyGCT | 0.00070271 | The frequency of GCT in AS region sequence |
| 132 | asseqfrequencyGG | 0.00135013 | The frequency of GG in AS region sequence |
| 133 | asseqfrequencyGGA | 0.00124547 | The frequency of GGA in AS region sequence |
| 134 | asseqfrequencyGGT | 0.00070964 | The frequency of GGT in AS region sequence |
| 135 | asseqfrequencyGGC | 0.0006017 | The frequency of GGC in AS region sequence |
| 136 | asseqfrequencyGGG | 0.00068444 | The frequency of GGG in AS region sequence |
| 137 | asseqdistr1%A | 0.00726205 | The distribution(position/length) of 1% A in AS region sequence |
| 138 | asseqdistr25%A | 0.00679882 | The distribution(position/length) of 25% A in AS region sequence |
| 139 | asseqdistr50%A | 0.00161645 | The distribution(position/length) of 50% A in AS region sequence |
| 140 | asseqdistr75%A | 0.00142922 | The distribution(position/length) of 75% A in AS region sequence |
| 141 | asseqdistr100%A | 0.0075252 | The distribution(position/length) of 100% A in AS region sequence |
| 142 | asseqdistr1%T | 0.00601582 | The distribution(position/length) of 1% T in AS region sequence |
| 143 | asseqdistr25%T | 0.0024819 | The distribution(position/length) of 25% T in AS region sequence |
| 144 | asseqdistr50%T | 0.00409853 | The distribution(position/length) of 50% T in AS region sequence |
| 145 | asseqdistr75%T | 0.004453 | The distribution(position/length) of 75% T in AS region sequence |
| 146 | asseqdistr100%T | 0.00978825 | The distribution(position/length) of 100% T in AS region sequence |
| 147 | asseqdistr1%C | 0.00352705 | The distribution(position/length) of 1% C in AS region sequence |
| 148 | asseqdistr25%C | 0.0019445 | The distribution(position/length) of 25% C in AS region sequence |
| 149 | asseqdistr50%C | 0.00090629 | The distribution(position/length) of 50% C in AS region sequence |
| 150 | asseqdistr75%C | 0.00240864 | The distribution(position/length) of 75% C in AS region sequence |
| 151 | asseqdistr100%C | 0.00857147 | The distribution(position/length) of 100% C in AS region sequence |
| 152 | asseqdistr1%G | 0.01593786 | The distribution(position/length) of 1% G in AS region sequence |
| 153 | asseqdistr25%G | 0.01090831 | The distribution(position/length) of 25% G in AS region sequence |
| 154 | asseqdistr50%G | 0.00478994 | The distribution(position/length) of 50% G in AS region sequence |
| 155 | asseqdistr75%G | 0.00422594 | The distribution(position/length) of 75% G in AS region sequence |
| 156 | asseqdistr100%G | 0.00723644 | The distribution(position/length) of 100% G in AS region sequence |
| 157 | asseqdonerGT | 0.00131268 | Is there GT in doner of AS region sequence (1 for yes, 0 for no) |
| 158 | asseqacceptorGT | 0.02884584 | Is there GT in acceptor of AS region sequence (1 for yes, 0 for no) |
| 159 | asseqdonerGC | 0.00222911 | Is there GC in doner of AS region sequence (1 for yes, 0 for no) |
| 160 | asseqacceptorGC | 0.00105832 | Is there GC in acceptor of AS region sequence (1 for yes, 0 for no) |
| 161 | asseqdonerAT | 0.00205132 | Is there AT in doner of AS region sequence (1 for yes, 0 for no) |
| 162 | asseqacceptorAT | 0.00089301 | Is there AT in acceptor of AS region sequence (1 for yes, 0 for no) |
| 163 | asseqdonerAG | 0.0279886 | Is there AG in doner of AS region sequence (1 for yes, 0 for no) |
| 164 | asseqacceptorAG | 0.00086802 | Is there AG in acceptor of AS region sequence (1 for yes, 0 for no) |
| 165 | upfrequencyTC | 0.00054334 | The frequency of TC in upstream sequence |
| 166 | upfrequencyC | 0.00057141 | The frequency of C in upstream sequence |
| 167 | updistr100%G | 0.00087752 | The distribution(position/length) of 100% G in upstream sequence |
| 168 | updonerGT | 0.00056503 | Is there GT in doner of upstream sequence (1 for yes, 0 for no) |
| 169 | updonerAG | 0.0010452 | Is there AG in doner of upstream sequence (1 for yes, 0 for no) |
| 170 | downfrequencyTC | 0.00063755 | The frequency of TC in downstream sequence |
| 171 | downfrequencyTCT | 0.00057412 | The frequency of TCT in downstream sequence |
| 172 | downfrequencyCT | 0.00065043 | The frequency of CT in downstream sequence |
| 173 | downdistr100%G | 0.00058024 | The distribution(position/length) of 100% G in downstream sequence |
| 174 | downdonerAG | 0.00055697 | Is there AG in doner of in downstream sequence (1 for yes, 0 for no) |
| 175 | up30as30if%3 | 0.00526169 | Whethere the length of upstream30 + AS 30bp sequence divisible by three |
| 176 | up30as30GC | 0.00102788 | The GC contant of upstream30AS 30bp + AS 30bp sequence |
| 177 | up30as30numberTAA | 0.00062303 | The number of stopdocon TAA in upstream30AS 30bp + AS 30bp sequence |
| 178 | up30as30frequencyAG | 0.00083881 | The frequency of AG in upstream30AS 30bp + AS 30bp sequence |
| 179 | up30as30frequencyAGG | 0.00063295 | The frequency of AGG in upstream30AS 30bp + AS 30bp sequence |
| 180 | up30as30frequencyTA | 0.00071584 | The frequency of TA in upstream30AS 30bp + AS 30bp sequence |
| 181 | up30as30frequencyTAA | 0.00066461 | The frequency of TAA in upstream30AS 30bp + AS 30bp sequence |
| 182 | up30as30frequencyTTT | 0.00054375 | The frequency of TTT in upstream30AS 30bp + AS 30bp sequence |
| 183 | up30as30frequencyCAG | 0.00129916 | The frequency of CAG in upstream30AS 30bp + AS 30bp sequence |
| 184 | up30as30frequencyG | 0.00057868 | The frequency of G in upstream30AS 30bp + AS 30bp sequence |
| 185 | up30as30frequencyGA | 0.00090161 | The frequency of GA in upstream30AS 30bp + AS 30bp sequence |
| 186 | up30as30frequencyGAT | 0.0005604 | The frequency of GAT in upstream30AS 30bp + AS 30bp sequence |
| 187 | up30as30frequencyGTA | 0.00224494 | The frequency of GTA in upstream30AS 30bp + AS 30bp sequence |
| 188 | up30as30frequencyGGA | 0.00056531 | The frequency of GGA in upstream30AS 30bp + AS 30bp sequence |
| 189 | up30as30frequencyGGT | 0.00241256 | The frequency of GGT in upstream30AS 30bp + AS 30bp sequence |
| 190 | up30as30frequencyGGC | 0.00053686 | The frequency of GGC in upstream30AS 30bp + AS 30bp sequence |
| 191 | up30as30distr100%A | 0.00146039 | The distribution(position/length) of 100% A in upstream30AS 30bp + AS 30bp sequence |
| 192 | up30as30distr50%T | 0.00124887 | The distribution(position/length) of 50% T in upstream30AS 30bp + AS 30bp sequence |
| 193 | up30as30distr75%T | 0.00116163 | The distribution(position/length) of 75% T in upstream30AS 30bp + AS 30bp sequence |
| 194 | up30as30distr100%T | 0.00142157 | The distribution(position/length) of 100% T in upstream30AS 30bp + AS 30bp sequence |
| 195 | up30as30distr50%C | 0.00056688 | The distribution(position/length) of 50% C in upstream30AS 30bp + AS 30bp sequence |
| 196 | up30as30distr75%C | 0.00078279 | The distribution(position/length) of 75% C in upstream30AS 30bp + AS 30bp sequence |
| 197 | up30as30distr25%G | 0.00052417 | The distribution(position/length) of 25% G in upstream30AS 30bp + AS 30bp sequence |
| 198 | up30as30distr50%G | 0.00143519 | The distribution(position/length) of 50% G in upstream30AS 30bp + AS 30bp sequence |
| 199 | up30as30distr75%G | 0.00368218 | The distribution(position/length) of 75% G in upstream30AS 30bp + AS 30bp sequence |
| 200 | up30as30distr100%G | 0.00300135 | The distribution(position/length) of 100% G in upstream30AS 30bp + AS 30bp sequence |
| 201 | up30as30donerGT | 0.00075511 | Is there GT in doner of upstream30AS 30bp + AS 30bp sequence (1 for yes, 0 for no) |
| 202 | up30as30donerGC | 0.00082364 | Is there GC in doner of upstream30AS 30bp + AS 30bp sequence (1 for yes, 0 for no) |
| 203 | up30as30donerAT | 0.00057632 | Is there AT in doner of upstream30AS 30bp + AS 30bp sequence (1 for yes, 0 for no) |
| 204 | up30as30donerAG | 0.00882961 | Is there AG in doner of upstream30AS 30bp + AS 30bp sequence (1 for yes, 0 for no) |
| 205 | up30down30distr50%G | 0.00066765 | The distribution(position/length) of 50% G in upstream 30 + downstream 30 sequence |
| 206 | up30down30distr75%G | 0.00055362 | The distribution(position/length) of 75% G in upstream 30 + downstream 30 sequence |
| 207 | as30down30if%3 | 0.00412092 | Whethere the length of AS 30bp + downstream 30bp sequence divisible by three |
| 208 | as30down30GC | 0.00102468 | The GC contant of AS 30bp + downstream 30bp sequence |
| 209 | as30down30frequencyAAG | 0.00055681 | The frequency of AAG in AS 30bp + downstream 30bp sequence |
| 210 | as30down30frequencyAG | 0.00066375 | The frequency of AG in AS 30bp + downstream 30bp sequence |
| 211 | as30down30frequencyAGG | 0.00057326 | The frequency of AGG in AS 30bp + downstream 30bp sequence |
| 212 | as30down30frequencyT | 0.00060534 | The frequency of T in AS 30bp + downstream 30bp sequence |
| 213 | as30down30frequencyTTT | 0.00064802 | The frequency of TTT in AS 30bp + downstream 30bp sequence |
| 214 | as30down30frequencyTC | 0.00051054 | The frequency of TC in AS 30bp + downstream 30bp sequence |
| 215 | as30down30frequencyTG | 0.00051856 | The frequency of TG in AS 30bp + downstream 30bp sequence |
| 216 | as30down30frequencyC | 0.00052519 | The frequency of C in AS 30bp + downstream 30bp sequence |
| 217 | as30down30frequencyCAG | 0.00161718 | The frequency of CAG in AS 30bp + downstream 30bp sequence |
| 218 | as30down30frequencyGAG | 0.00060058 | The frequency of GAG in AS 30bp + downstream 30bp sequence |
| 219 | as30down30distr1%A | 0.00071615 | The distribution(position/length) of 1% A in AS 30bp + downstream 30bp sequence |
| 220 | as30down30distr1%T | 0.00065785 | The distribution(position/length) of 1% T in AS 30bp + downstream 30bp sequence |
| 221 | as30down30distr25%T | 0.00051918 | The distribution(position/length) of 25% T in AS 30bp + downstream 30bp sequence |
| 222 | as30down30distr50%T | 0.00121401 | The distribution(position/length) of 50% T in AS 30bp + downstream 30bp sequence |
| 223 | as30down30distr75%T | 0.00051052 | The distribution(position/length) of 75% T in AS 30bp + downstream 30bp sequence |
| 224 | as30down30distr1%G | 0.00161531 | The distribution(position/length) of 1% G in AS 30bp + downstream 30bp sequence |
| 225 | as30down30distr25%G | 0.00073252 | The distribution(position/length) of 25% G in AS 30bp + downstream 30bp sequence |
| 226 | as30down30distr50%G | 0.00137949 | The distribution(position/length) of 50% G in AS 30bp + downstream 30bp sequence |
| 227 | as30down30distr75%G | 0.00054911 | The distribution(position/length) of 75% G in AS 30bp + downstream 30bp sequence |
| 228 | as30down30acceptorGT | 0.00237995 | Is there GT in acceptor of AS 30bp + downstream 30bp sequence (1 for yes, 0 for no) |
| 229 | as30down30acceptorAG | 0.00103183 | Is there AG in acceptor of AS 30bp + downstream 30bp sequence (1 for yes, 0 for no) |
| 230 | up50as50if%3 | 0.00313826 | Whethere the length of upstream 50bp + AS 50bp sequence divisible by three |
| 231 | up50as50GC | 0.00248468 | The GC contant of upstream 50bp + AS 50bp sequence |
| 232 | up50as50numberTAA | 0.00090647 | The numberof stopdocon TAA in upstream 50bp + AS 50bp sequence |
| 233 | up50as50frequencyAG | 0.00066188 | The frequency of AG in upstream 50bp + AS 50bp sequence |
| 234 | up50as50frequencyAGG | 0.00052156 | The frequency of AGG in upstream 50bp + AS 50bp sequence |
| 235 | up50as50frequencyT | 0.00055684 | The frequency of T in upstream 50bp + AS 50bp sequence |
| 236 | up50as50frequencyTA | 0.00076884 | The frequency of TA in upstream 50bp + AS 50bp sequence |
| 237 | up50as50frequencyTAA | 0.00107979 | The frequency of TAA in upstream 50bp + AS 50bp sequence |
| 238 | up50as50frequencyTTA | 0.00061682 | The frequency of TTA in upstream 50bp + AS 50bp sequence |
| 239 | up50as50frequencyCAG | 0.00110433 | The frequency of CAG in upstream 50bp + AS 50bp sequence |
| 240 | up50as50frequencyG | 0.00076794 | The frequency of G in upstream 50bp + AS 50bp sequence |
| 241 | up50as50frequencyGA | 0.00070365 | The frequency of GA in upstream 50bp + AS 50bp sequence |
| 242 | up50as50frequencyGAG | 0.00051534 | The frequency of GAG in upstream 50bp + AS 50bp sequence |
| 243 | up50as50frequencyGTA | 0.00156303 | The frequency of GTA in upstream 50bp + AS 50bp sequence |
| 244 | up50as50frequencyGGA | 0.00060143 | The frequency of GGA in upstream 50bp + AS 50bp sequence |
| 245 | up50as50frequencyGGT | 0.00100631 | The frequency of GGT in upstream 50bp + AS 50bp sequence |
| 246 | up50as50distr100%A | 0.0013585 | The distribution(position/length) of 100% A in upstream 50bp + AS 50bp sequence |
| 247 | up50as50distr50%T | 0.00222866 | The distribution(position/length) of 50% T in upstream 50bp + AS 50bp sequence |
| 248 | up50as50distr75%T | 0.00060388 | The distribution(position/length) of 75% T in upstream 50bp + AS 50bp sequence |
| 249 | up50as50distr100%T | 0.00220456 | The distribution(position/length) of 100% T in upstream 50bp + AS 50bp sequence |
| 250 | up50as50distr50%C | 0.00052123 | The distribution(position/length) of 50% C in upstream 50bp + AS 50bp sequence |
| 251 | up50as50distr75%C | 0.00059727 | The distribution(position/length) of 75% C in upstream 50bp + AS 50bp sequence |
| 252 | up50as50distr100%C | 0.00066252 | The distribution(position/length) of 100% C in upstream 50bp + AS 50bp sequence |
| 253 | up50as50distr1%G | 0.0005147 | The distribution(position/length) of 1% G in upstream 50bp + AS 50bp sequence |
| 254 | up50as50distr25%G | 0.00066241 | The distribution(position/length) of 25% G in upstream 50bp + AS 50bp sequence |
| 255 | up50as50distr50%G | 0.00175489 | The distribution(position/length) of 50% G in upstream 50bp + AS 50bp sequence |
| 256 | up50as50distr75%G | 0.00172491 | The distribution(position/length) of 75% G in upstream 50bp + AS 50bp sequence |
| 257 | up50as50distr100%G | 0.00265711 | The distribution(position/length) of 100% G in upstream 50bp + AS 50bp sequence |
| 258 | up50as50donerGT | 0.00080078 | Is there GT in doner of upstream 50bp + AS 50bp sequence (1 for yes, 0 for no) |
| 259 | up50as50donerGC | 0.00072178 | Is there GC in doner of upstream 50bp + AS 50bp sequence (1 for yes, 0 for no) |
| 260 | up50as50donerAT | 0.00075531 | Is there AT in doner of upstream 50bp + AS 50bp sequence (1 for yes, 0 for no) |
| 261 | up50as50donerAG | 0.00973815 | Is there AG in doner of upstream 50bp + AS 50bp sequence (1 for yes, 0 for no) |
| 262 | up50down50distr25%C | 0.00056377 | The distribution(position/length) of 25% C in upstream 50bp + downstream 50bp sequence (1 for yes, 0 for no) |
| 263 | up50down50distr50%C | 0.00064309 | The distribution(position/length) of 50% C in upstream 50bp + downstream 50bp sequence (1 for yes, 0 for no) |
| 264 | up50down50distr50%G | 0.00066223 | The distribution(position/length) of 50% G in upstream 50bp + downstream 50bp sequence (1 for yes, 0 for no) |
| 265 | up50down50distr75%G | 0.00055767 | The distribution(position/length) of 75% G in upstream 50bp + downstream 50bp sequence (1 for yes, 0 for no) |
| 266 | up50down50distr100%G | 0.00054658 | The distribution(position/length) of 100% G in upstream 50bp + downstream 50bp sequence (1 for yes, 0 for no) |
| 267 | as50down50if%3 | 0.00369669 | Whethere the length of AS 50bp + downstream 50bp sequence divisible by three |
| 268 | as50down50GC | 0.00349382 | The GC contant of GC in AS 50bp + downstream 50bp sequence |
| 269 | as50down50numberTAA | 0.0008039 | The number of stopdocon TAA in AS 50bp + downstream 50bp sequence |
| 270 | as50down50frequencyAAG | 0.00053626 | The frequency of AAG in AS 50bp + downstream 50bp sequence |
| 271 | as50down50frequencyAG | 0.000601 | The frequency of AG in AS 50bp + downstream 50bp sequence |
| 272 | as50down50frequencyAGA | 0.00051576 | The frequency of AGA in AS 50bp + downstream 50bp sequence |
| 273 | as50down50frequencyAGG | 0.00060834 | The frequency of AGG in AS 50bp + downstream 50bp sequence |
| 274 | as50down50frequencyT | 0.00096353 | The frequency of T in AS 50bp + downstream 50bp sequence |
| 275 | as50down50frequencyTAA | 0.00052375 | The frequency of TAA in AS 50bp + downstream 50bp sequence |
| 276 | as50down50frequencyTT | 0.00064601 | The frequency of TT in AS 50bp + downstream 50bp sequence |
| 277 | as50down50frequencyTTA | 0.00061379 | The frequency of TTA in AS 50bp + downstream 50bp sequence |
| 278 | as50down50frequencyTC | 0.00060552 | The frequency of TC in AS 50bp + downstream 50bp sequence |
| 279 | as50down50frequencyTCG | 0.0005862 | The frequency of TCG in AS 50bp + downstream 50bp sequence |
| 280 | as50down50frequencyTGT | 0.0007312 | The frequency of TGT in AS 50bp + downstream 50bp sequence |
| 281 | as50down50frequencyC | 0.00053764 | The frequency of C in AS 50bp + downstream 50bp sequence |
| 282 | as50down50frequencyCAG | 0.00073511 | The frequency of CAG in AS 50bp + downstream 50bp sequence |
| 283 | as50down50frequencyCTC | 0.00056771 | The frequency of CTC in AS 50bp + downstream 50bp sequence |
| 284 | as50down50frequencyCG | 0.00056719 | The frequency of CG in AS 50bp + downstream 50bp sequence |
| 285 | as50down50frequencyCGA | 0.00051527 | The frequency of CGA in AS 50bp + downstream 50bp sequence |
| 286 | as50down50frequencyG | 0.00068001 | The frequency of G in AS 50bp + downstream 50bp sequence |
| 287 | as50down50distr1%A | 0.0008114 | The distribution(position/length) of 1% A in AS 50bp + downstream 50bp sequence |
| 288 | as50down50distr1%T | 0.00154536 | The distribution(position/length) of 1% T in AS 50bp + downstream 50bp sequence |
| 289 | as50down50distr50%T | 0.00121784 | The distribution(position/length) of 50% T in AS 50bp + downstream 50bp sequence |
| 290 | as50down50distr1%C | 0.00053517 | The distribution(position/length) of 1% C in AS 50bp + downstream 50bp sequence |
| 291 | as50down50distr1%G | 0.00166606 | The distribution(position/length) of 1% G in AS 50bp + downstream 50bp sequence |
| 292 | as50down50distr25%G | 0.00117026 | The distribution(position/length) of 25% G in AS 50bp + downstream 50bp sequence |
| 293 | as50down50distr50%G | 0.00155005 | The distribution(position/length) of 50% G in AS 50bp + downstream 50bp sequence |
| 294 | as50down50distr75%G | 0.00051549 | The distribution(position/length) of 75% G in AS 50bp + downstream 50bp sequence |
| 295 | as50down50distr100%G | 0.00052391 | The distribution(position/length) of 100% G in AS 50bp + downstream 50bp sequence |
| 296 | as50down50acceptorGT | 0.00348638 | Is there GT in acceptor of AS 50bp + downstream 50bp sequence (1 for yes, 0 for no) |
| 297 | as50down50acceptorAG | 0.00065119 | Is there AG in acceptor of AS 50bp + downstream 50bp sequence (1 for yes, 0 for no) |

Table S8 Comparison of XGBoost in balanced and imbalanced dataset

| Models | Datasets | Metrics | ES | AA | AD | IR | Overall |
| --- | --- | --- | --- | --- | --- | --- | --- |
| Human | Balance dataset | Accuracy (%) |  |  |  |  | 92.31 |
|  |  | Precision (%) | 90.12 | 91.57 | 92.47 | 91.33 |  |
|  |  | Recall (%) | 91.89 | 87.22 | 90.49 | 95.8 |  |
|  |  | F1-score | 0.91 | 0.89 | 0.91 | 0.94 |  |
|  |  | True positive | 10784 | 10905 | 11199 | 11033 | 43921 |
|  | Imbalance dataset | Accuracy (%) |  |  |  |  | 87.61 |
|  |  | Precision (%) | 89.39 | 85.46 | 88.36 | 80.94 |  |
|  |  | Recall (%) | 92.69 | 78.9 | 85.35 | 84.98 |  |
|  |  | F1-score | 0.91 | 0.82 | 0.87 | 0.83 |  |
|  |  | True positive | 10672 | 4386 | 4030 | 1752 | 20840 |
| *Arabidopsis thaliana* | Balance dataset | Accuracy (%) |  |  |  |  | 92.89 |
|  |  | Precision (%) | 95.65 | 94.91 | 93.88 | 90.41 |  |
|  |  | Recall (%) | 94.33 | 90.37 | 91.83 | 97.91 |  |
|  |  | F1-score | 0.95 | 0.93 | 0.93 | 0.94 |  |
|  |  | True positive | 1671 | 1744 | 1677 | 1645 | 6738 |
|  | Imbalance dataset | Accuracy (%) |  |  |  |  | 90.17 |
|  |  | Precision (%) | 81.53 | 90.33 | 92.86 | 89.92 |  |
|  |  | Recall (%) | 61.15 | 92.48 | 82.12 | 97.38 |  |
|  |  | F1-score | 0.70 | 0.91 | 0.87 | 0.94 |  |
|  |  | True positive | 241 | 1141 | 867 | 1647 | 3897 |

Table S9 The grid search of training the optimal hyperparameter combinations of the human model

| Hyperparameters | | | | | Accuracy（%） |
| --- | --- | --- | --- | --- | --- |
| colsample_bytree | learning_rate | max_depth | min_child_weight | subsample |  |
| 0.5 | 0.1 | 7 | 3 | 0.6 | 92.73 |
| 0.5 | 0.1 | 7 | 3 | 0.7 | 92.95 |
| 0.5 | 0.1 | 7 | 3 | 0.8 | 91.06 |
| 0.5 | 0.1 | 7 | 4 | 0.6 | 93.36 |
| 0.5 | 0.1 | 7 | 4 | 0.7 | 92.32 |
| 0.5 | 0.1 | 7 | 4 | 0.8 | 91.19 |
| 0.5 | 0.1 | 7 | 5 | 0.6 | 92.24 |
| 0.5 | 0.1 | 7 | 5 | 0.7 | 90.58 |
| 0.5 | 0.1 | 7 | 5 | 0.8 | 91.85 |
| 0.5 | 0.1 | 8 | 3 | 0.6 | 92.03 |
| 0.5 | 0.1 | 8 | 3 | 0.7 | 92.09 |
| 0.5 | 0.1 | 8 | 3 | 0.8 | 93.70 |
| 0.5 | 0.1 | 8 | 4 | 0.6 | 92.74 |
| 0.5 | 0.1 | 8 | 4 | 0.7 | 90.93 |
| 0.5 | 0.1 | 8 | 4 | 0.8 | 92.88 |
| 0.5 | 0.1 | 8 | 5 | 0.6 | 93.97 |
| 0.5 | 0.1 | 8 | 5 | 0.7 | 93.43 |
| 0.5 | 0.1 | 8 | 5 | 0.8 | 92.55 |
| 0.5 | 0.1 | 9 | 3 | 0.6 | 90.63 |
| 0.5 | 0.1 | 9 | 3 | 0.7 | 92.98 |
| 0.5 | 0.1 | 9 | 3 | 0.8 | 93.91 |
| 0.5 | 0.1 | 9 | 4 | 0.6 | 91.55 |
| 0.5 | 0.1 | 9 | 4 | 0.7 | 91.26 |
| 0.5 | 0.1 | 9 | 4 | 0.8 | 92.46 |
| 0.5 | 0.1 | 9 | 5 | 0.6 | 91.70 |
| 0.5 | 0.1 | 9 | 5 | 0.7 | 92.80 |
| 0.5 | 0.1 | 9 | 5 | 0.8 | 91.54 |
| 0.5 | 0.2 | 7 | 3 | 0.6 | 90.77 |
| 0.5 | 0.2 | 7 | 3 | 0.7 | 90.53 |
| 0.5 | 0.2 | 7 | 3 | 0.8 | 90.62 |
| 0.5 | 0.2 | 7 | 4 | 0.6 | 92.70 |
| 0.5 | 0.2 | 7 | 4 | 0.7 | 93.82 |
| 0.5 | 0.2 | 7 | 4 | 0.8 | 92.81 |
| 0.5 | 0.2 | 7 | 5 | 0.6 | 92.24 |
| 0.5 | 0.2 | 7 | 5 | 0.7 | 92.43 |
| 0.5 | 0.2 | 7 | 5 | 0.8 | 93.84 |
| 0.5 | 0.2 | 8 | 3 | 0.6 | 90.51 |
| 0.5 | 0.2 | 8 | 3 | 0.7 | 91.82 |
| 0.5 | 0.2 | 8 | 3 | 0.8 | 93.76 |
| 0.5 | 0.2 | 8 | 4 | 0.6 | 91.77 |
| 0.5 | 0.2 | 8 | 4 | 0.7 | 93.46 |
| 0.5 | 0.2 | 8 | 4 | 0.8 | 93.39 |
| 0.5 | 0.2 | 8 | 5 | 0.6 | 92.90 |
| 0.5 | 0.2 | 8 | 5 | 0.7 | 93.81 |
| 0.5 | 0.2 | 8 | 5 | 0.8 | 93.80 |
| 0.5 | 0.2 | 9 | 3 | 0.6 | 93.41 |
| 0.5 | 0.2 | 9 | 3 | 0.7 | 94.01 |
| 0.5 | 0.2 | 9 | 3 | 0.8 | 91.33 |
| 0.5 | 0.2 | 9 | 4 | 0.6 | 92.28 |
| 0.5 | 0.2 | 9 | 4 | 0.7 | 94.09 |
| 0.5 | 0.2 | 9 | 4 | 0.8 | 90.57 |
| 0.5 | 0.2 | 9 | 5 | 0.6 | 92.69 |
| 0.5 | 0.2 | 9 | 5 | 0.7 | 93.05 |
| 0.5 | 0.2 | 9 | 5 | 0.8 | 92.15 |
| 0.5 | 0.3 | 7 | 3 | 0.6 | 92.48 |
| 0.5 | 0.3 | 7 | 3 | 0.7 | 91.37 |
| 0.5 | 0.3 | 7 | 3 | 0.8 | 92.32 |
| 0.5 | 0.3 | 7 | 4 | 0.6 | 93.73 |
| 0.5 | 0.3 | 7 | 4 | 0.7 | 91.01 |
| 0.5 | 0.3 | 7 | 4 | 0.8 | 90.70 |
| 0.5 | 0.3 | 7 | 5 | 0.6 | 92.86 |
| 0.5 | 0.3 | 7 | 5 | 0.7 | 91.92 |
| 0.5 | 0.3 | 7 | 5 | 0.8 | 93.67 |
| 0.5 | 0.3 | 8 | 3 | 0.6 | 94.07 |
| 0.5 | 0.3 | 8 | 3 | 0.7 | 91.22 |
| 0.5 | 0.3 | 8 | 3 | 0.8 | 92.41 |
| 0.5 | 0.3 | 8 | 4 | 0.6 | 91.16 |
| 0.5 | 0.3 | 8 | 4 | 0.7 | 92.16 |
| 0.5 | 0.3 | 8 | 4 | 0.8 | 90.75 |
| 0.5 | 0.3 | 8 | 5 | 0.6 | 92.69 |
| 0.5 | 0.3 | 8 | 5 | 0.7 | 91.22 |
| 0.5 | 0.3 | 8 | 5 | 0.8 | 91.00 |
| 0.5 | 0.3 | 9 | 3 | 0.6 | 93.07 |
| 0.5 | 0.3 | 9 | 3 | 0.7 | 93.08 |
| 0.5 | 0.3 | 9 | 3 | 0.8 | 91.64 |
| 0.5 | 0.3 | 9 | 4 | 0.6 | 93.53 |
| 0.5 | 0.3 | 9 | 4 | 0.7 | 91.80 |
| 0.5 | 0.3 | 9 | 4 | 0.8 | 93.03 |
| 0.5 | 0.3 | 9 | 5 | 0.6 | 90.96 |
| 0.5 | 0.3 | 9 | 5 | 0.7 | 91.19 |
| 0.5 | 0.3 | 9 | 5 | 0.8 | 93.21 |
| 0.6 | 0.1 | 7 | 3 | 0.6 | 91.76 |
| 0.6 | 0.1 | 7 | 3 | 0.7 | 93.39 |
| 0.6 | 0.1 | 7 | 3 | 0.8 | 91.62 |
| 0.6 | 0.1 | 7 | 4 | 0.6 | 91.20 |
| 0.6 | 0.1 | 7 | 4 | 0.7 | 93.12 |
| 0.6 | 0.1 | 7 | 4 | 0.8 | 91.24 |
| 0.6 | 0.1 | 7 | 5 | 0.6 | 93.48 |
| 0.6 | 0.1 | 7 | 5 | 0.7 | 93.81 |
| 0.6 | 0.1 | 7 | 5 | 0.8 | 92.56 |
| 0.6 | 0.1 | 8 | 3 | 0.6 | 92.42 |
| 0.6 | 0.1 | 8 | 3 | 0.7 | 93.92 |
| 0.6 | 0.1 | 8 | 3 | 0.8 | 91.28 |
| 0.6 | 0.1 | 8 | 4 | 0.6 | 90.83 |
| 0.6 | 0.1 | 8 | 4 | 0.7 | 92.86 |
| 0.6 | 0.1 | 8 | 4 | 0.8 | 91.65 |
| 0.6 | 0.1 | 8 | 5 | 0.6 | 92.76 |
| 0.6 | 0.1 | 8 | 5 | 0.7 | 91.48 |
| 0.6 | 0.1 | 8 | 5 | 0.8 | 93.41 |
| 0.6 | 0.1 | 9 | 3 | 0.6 | 93.95 |
| 0.6 | 0.1 | 9 | 3 | 0.7 | 93.95 |
| 0.6 | 0.1 | 9 | 3 | 0.8 | 93.08 |
| 0.6 | 0.1 | 9 | 4 | 0.6 | 92.46 |
| 0.6 | 0.1 | 9 | 4 | 0.7 | 93.48 |
| 0.6 | 0.1 | 9 | 4 | 0.8 | 92.04 |
| 0.6 | 0.1 | 9 | 5 | 0.6 | 92.99 |
| 0.6 | 0.1 | 9 | 5 | 0.7 | 92.13 |
| 0.6 | 0.1 | 9 | 5 | 0.8 | 92.09 |
| 0.6 | 0.2 | 7 | 3 | 0.6 | 90.64 |
| 0.6 | 0.2 | 7 | 3 | 0.7 | 92.09 |
| 0.6 | 0.2 | 7 | 3 | 0.8 | 92.70 |
| 0.6 | 0.2 | 7 | 4 | 0.6 | 93.95 |
| 0.6 | 0.2 | 7 | 4 | 0.7 | 90.72 |
| 0.6 | 0.2 | 7 | 4 | 0.8 | 91.44 |
| 0.6 | 0.2 | 7 | 5 | 0.6 | 92.22 |
| 0.6 | 0.2 | 7 | 5 | 0.7 | 93.57 |
| 0.6 | 0.2 | 7 | 5 | 0.8 | 93.71 |
| 0.6 | 0.2 | 8 | 3 | 0.6 | 93.55 |
| 0.6 | 0.2 | 8 | 3 | 0.7 | 91.27 |
| 0.6 | 0.2 | 8 | 3 | 0.8 | 91.93 |
| 0.6 | 0.2 | 8 | 4 | 0.6 | 91.22 |
| 0.6 | 0.2 | 8 | 4 | 0.7 | 93.98 |
| 0.6 | 0.2 | 8 | 4 | 0.8 | 93.09 |
| 0.6 | 0.2 | 8 | 5 | 0.6 | 91.83 |
| 0.6 | 0.2 | 8 | 5 | 0.7 | 90.73 |
| 0.6 | 0.2 | 8 | 5 | 0.8 | 93.67 |
| 0.6 | 0.2 | 9 | 3 | 0.6 | 92.40 |
| 0.6 | 0.2 | 9 | 3 | 0.7 | 92.55 |
| 0.6 | 0.2 | 9 | 3 | 0.8 | 91.93 |
| 0.6 | 0.2 | 9 | 4 | 0.6 | 92.17 |
| 0.6 | 0.2 | 9 | 4 | 0.7 | 91.11 |
| 0.6 | 0.2 | 9 | 4 | 0.8 | 91.10 |
| 0.6 | 0.2 | 9 | 5 | 0.6 | 92.85 |
| 0.6 | 0.2 | 9 | 5 | 0.7 | 90.81 |
| 0.6 | 0.2 | 9 | 5 | 0.8 | 93.97 |
| 0.6 | 0.3 | 7 | 3 | 0.6 | 92.31 |
| 0.6 | 0.3 | 7 | 3 | 0.7 | 90.68 |
| 0.6 | 0.3 | 7 | 3 | 0.8 | 91.16 |
| 0.6 | 0.3 | 7 | 4 | 0.6 | 92.27 |
| 0.6 | 0.3 | 7 | 4 | 0.7 | 93.98 |
| 0.6 | 0.3 | 7 | 4 | 0.8 | 92.13 |
| 0.6 | 0.3 | 7 | 5 | 0.6 | 93.82 |
| 0.6 | 0.3 | 7 | 5 | 0.7 | 90.71 |
| 0.6 | 0.3 | 7 | 5 | 0.8 | 91.25 |
| 0.6 | 0.3 | 8 | 3 | 0.6 | 91.76 |
| 0.6 | 0.3 | 8 | 3 | 0.7 | 91.65 |
| 0.6 | 0.3 | 8 | 3 | 0.8 | 93.60 |
| 0.6 | 0.3 | 8 | 4 | 0.6 | 92.58 |
| 0.6 | 0.3 | 8 | 4 | 0.7 | 90.76 |
| 0.6 | 0.3 | 8 | 4 | 0.8 | 90.86 |
| 0.6 | 0.3 | 8 | 5 | 0.6 | 90.97 |
| 0.6 | 0.3 | 8 | 5 | 0.7 | 91.70 |
| 0.6 | 0.3 | 8 | 5 | 0.8 | 92.39 |
| 0.6 | 0.3 | 9 | 3 | 0.6 | 90.94 |
| 0.6 | 0.3 | 9 | 3 | 0.7 | 93.72 |
| 0.6 | 0.3 | 9 | 3 | 0.8 | 90.88 |
| 0.6 | 0.3 | 9 | 4 | 0.6 | 94.07 |
| 0.6 | 0.3 | 9 | 4 | 0.7 | 90.99 |
| 0.6 | 0.3 | 9 | 4 | 0.8 | 92.07 |
| 0.6 | 0.3 | 9 | 5 | 0.6 | 93.99 |
| 0.6 | 0.3 | 9 | 5 | 0.7 | 92.58 |
| 0.6 | 0.3 | 9 | 5 | 0.8 | 91.22 |
| 0.7 | 0.1 | 7 | 3 | 0.6 | 92.72 |
| 0.7 | 0.1 | 7 | 3 | 0.7 | 90.93 |
| 0.7 | 0.1 | 7 | 3 | 0.8 | 92.70 |
| 0.7 | 0.1 | 7 | 4 | 0.6 | 93.52 |
| 0.7 | 0.1 | 7 | 4 | 0.7 | 90.75 |
| 0.7 | 0.1 | 7 | 4 | 0.8 | 92.37 |
| 0.7 | 0.1 | 7 | 5 | 0.6 | 93.02 |
| 0.7 | 0.1 | 7 | 5 | 0.7 | 90.94 |
| 0.7 | 0.1 | 7 | 5 | 0.8 | 93.92 |
| 0.7 | 0.1 | 8 | 3 | 0.6 | 91.76 |
| 0.7 | 0.1 | 8 | 3 | 0.7 | 93.54 |
| 0.7 | 0.1 | 8 | 3 | 0.8 | 93.69 |
| 0.7 | 0.1 | 8 | 4 | 0.6 | 93.46 |
| 0.7 | 0.1 | 8 | 4 | 0.7 | 93.14 |
| 0.7 | 0.1 | 8 | 4 | 0.8 | 90.66 |
| 0.7 | 0.1 | 8 | 5 | 0.6 | 92.11 |
| 0.7 | 0.1 | 8 | 5 | 0.7 | 92.30 |
| 0.7 | 0.1 | 8 | 5 | 0.8 | 93.26 |
| 0.7 | 0.1 | 9 | 3 | 0.6 | 92.69 |
| 0.7 | 0.1 | 9 | 3 | 0.7 | 93.45 |
| 0.7 | 0.1 | 9 | 3 | 0.8 | 91.74 |
| 0.7 | 0.1 | 9 | 4 | 0.6 | 91.01 |
| 0.7 | 0.1 | 9 | 4 | 0.7 | 90.93 |
| 0.7 | 0.1 | 9 | 4 | 0.8 | 92.02 |
| 0.7 | 0.1 | 9 | 5 | 0.6 | 92.42 |
| 0.7 | 0.1 | 9 | 5 | 0.7 | 91.21 |
| 0.7 | 0.1 | 9 | 5 | 0.8 | 93.19 |
| 0.7 | 0.2 | 7 | 3 | 0.6 | 93.73 |
| 0.7 | 0.2 | 7 | 3 | 0.7 | 93.06 |
| 0.7 | 0.2 | 7 | 3 | 0.8 | 90.86 |
| 0.7 | 0.2 | 7 | 4 | 0.6 | 93.11 |
| 0.7 | 0.2 | 7 | 4 | 0.7 | 93.26 |
| 0.7 | 0.2 | 7 | 4 | 0.8 | 91.07 |
| 0.7 | 0.2 | 7 | 5 | 0.6 | 91.66 |
| 0.7 | 0.2 | 7 | 5 | 0.7 | 90.62 |
| 0.7 | 0.2 | 7 | 5 | 0.8 | 90.52 |
| 0.7 | 0.2 | 8 | 3 | 0.6 | 92.59 |
| 0.7 | 0.2 | 8 | 3 | 0.7 | 92.08 |
| 0.7 | 0.2 | 8 | 3 | 0.8 | 93.52 |
| 0.7 | 0.2 | 8 | 4 | 0.6 | 93.82 |
| 0.7 | 0.2 | 8 | 4 | 0.7 | 90.97 |
| 0.7 | 0.2 | 8 | 4 | 0.8 | 91.24 |
| 0.7 | 0.2 | 8 | 5 | 0.6 | 90.80 |
| 0.7 | 0.2 | 8 | 5 | 0.7 | 93.52 |
| 0.7 | 0.2 | 8 | 5 | 0.8 | 92.70 |
| 0.7 | 0.2 | 9 | 3 | 0.6 | 91.68 |
| 0.7 | 0.2 | 9 | 3 | 0.7 | 90.62 |
| 0.7 | 0.2 | 9 | 3 | 0.8 | 92.57 |
| 0.7 | 0.2 | 9 | 4 | 0.6 | 93.69 |
| 0.7 | 0.2 | 9 | 4 | 0.7 | 93.32 |
| 0.7 | 0.2 | 9 | 4 | 0.8 | 92.21 |
| 0.7 | 0.2 | 9 | 5 | 0.6 | 92.75 |
| 0.7 | 0.2 | 9 | 5 | 0.7 | 90.72 |
| 0.7 | 0.2 | 9 | 5 | 0.8 | 92.69 |
| 0.7 | 0.3 | 7 | 3 | 0.6 | 91.46 |
| 0.7 | 0.3 | 7 | 3 | 0.7 | 92.97 |
| 0.7 | 0.3 | 7 | 3 | 0.8 | 91.22 |
| 0.7 | 0.3 | 7 | 4 | 0.6 | 92.70 |
| 0.7 | 0.3 | 7 | 4 | 0.7 | 91.98 |
| 0.7 | 0.3 | 7 | 4 | 0.8 | 93.96 |
| 0.7 | 0.3 | 7 | 5 | 0.6 | 93.87 |
| 0.7 | 0.3 | 7 | 5 | 0.7 | 90.83 |
| 0.7 | 0.3 | 7 | 5 | 0.8 | 91.28 |
| 0.7 | 0.3 | 8 | 3 | 0.6 | 90.76 |
| 0.7 | 0.3 | 8 | 3 | 0.7 | 93.25 |
| 0.7 | 0.3 | 8 | 3 | 0.8 | 92.94 |
| 0.7 | 0.3 | 8 | 4 | 0.6 | 93.63 |
| 0.7 | 0.3 | 8 | 4 | 0.7 | 92.04 |
| 0.7 | 0.3 | 8 | 4 | 0.8 | 92.09 |
| 0.7 | 0.3 | 8 | 5 | 0.6 | 92.36 |
| 0.7 | 0.3 | 8 | 5 | 0.7 | 93.10 |
| 0.7 | 0.3 | 8 | 5 | 0.8 | 92.73 |
| 0.7 | 0.3 | 9 | 3 | 0.6 | 91.16 |
| 0.7 | 0.3 | 9 | 3 | 0.7 | 92.19 |
| 0.7 | 0.3 | 9 | 3 | 0.8 | 91.47 |
| 0.7 | 0.3 | 9 | 4 | 0.6 | 92.57 |
| 0.7 | 0.3 | 9 | 4 | 0.7 | 90.81 |
| 0.7 | 0.3 | 9 | 4 | 0.8 | 91.26 |
| 0.7 | 0.3 | 9 | 5 | 0.6 | 91.77 |
| 0.7 | 0.3 | 9 | 5 | 0.7 | 91.40 |
| 0.7 | 0.3 | 9 | 5 | 0.8 | 94.03 |

Table S10 The grid search of training the optimal hyperparameter combinations of the *Arabidopsis thaliana* model

| Hyperparameters | | | | | Accuracy（%） |
| --- | --- | --- | --- | --- | --- |
| colsample_bytree | learning_rate | max_depth | min_child_weight | subsample |  |
| 0.5 | 0.1 | 7 | 3 | 0.6 | 92.45 |
| 0.5 | 0.1 | 7 | 3 | 0.7 | 92.09 |
| 0.5 | 0.1 | 7 | 3 | 0.8 | 91.91 |
| 0.5 | 0.1 | 7 | 4 | 0.6 | 90.12 |
| 0.5 | 0.1 | 7 | 4 | 0.7 | 90.68 |
| 0.5 | 0.1 | 7 | 4 | 0.8 | 92.94 |
| 0.5 | 0.1 | 7 | 5 | 0.6 | 90.54 |
| 0.5 | 0.1 | 7 | 5 | 0.7 | 90.75 |
| 0.5 | 0.1 | 7 | 5 | 0.8 | 91.00 |
| 0.5 | 0.1 | 8 | 3 | 0.6 | 91.78 |
| 0.5 | 0.1 | 8 | 3 | 0.7 | 90.61 |
| 0.5 | 0.1 | 8 | 3 | 0.8 | 92.61 |
| 0.5 | 0.1 | 8 | 4 | 0.6 | 91.32 |
| 0.5 | 0.1 | 8 | 4 | 0.7 | 90.22 |
| 0.5 | 0.1 | 8 | 4 | 0.8 | 90.28 |
| 0.5 | 0.1 | 8 | 5 | 0.6 | 92.69 |
| 0.5 | 0.1 | 8 | 5 | 0.7 | 92.83 |
| 0.5 | 0.1 | 8 | 5 | 0.8 | 93.35 |
| 0.5 | 0.1 | 9 | 3 | 0.6 | 93.37 |
| 0.5 | 0.1 | 9 | 3 | 0.7 | 91.70 |
| 0.5 | 0.1 | 9 | 3 | 0.8 | 93.35 |
| 0.5 | 0.1 | 9 | 4 | 0.6 | 91.34 |
| 0.5 | 0.1 | 9 | 4 | 0.7 | 90.95 |
| 0.5 | 0.1 | 9 | 4 | 0.8 | 91.18 |
| 0.5 | 0.1 | 9 | 5 | 0.6 | 90.75 |
| 0.5 | 0.1 | 9 | 5 | 0.7 | 90.23 |
| 0.5 | 0.1 | 9 | 5 | 0.8 | 93.07 |
| 0.5 | 0.2 | 7 | 3 | 0.6 | 93.27 |
| 0.5 | 0.2 | 7 | 3 | 0.7 | 91.18 |
| 0.5 | 0.2 | 7 | 3 | 0.8 | 91.51 |
| 0.5 | 0.2 | 7 | 4 | 0.6 | 91.17 |
| 0.5 | 0.2 | 7 | 4 | 0.7 | 90.44 |
| 0.5 | 0.2 | 7 | 4 | 0.8 | 91.45 |
| 0.5 | 0.2 | 7 | 5 | 0.6 | 90.56 |
| 0.5 | 0.2 | 7 | 5 | 0.7 | 90.47 |
| 0.5 | 0.2 | 7 | 5 | 0.8 | 90.37 |
| 0.5 | 0.2 | 8 | 3 | 0.6 | 91.92 |
| 0.5 | 0.2 | 8 | 3 | 0.7 | 93.12 |
| 0.5 | 0.2 | 8 | 3 | 0.8 | 91.03 |
| 0.5 | 0.2 | 8 | 4 | 0.6 | 90.87 |
| 0.5 | 0.2 | 8 | 4 | 0.7 | 93.27 |
| 0.5 | 0.2 | 8 | 4 | 0.8 | 93.00 |
| 0.5 | 0.2 | 8 | 5 | 0.6 | 90.41 |
| 0.5 | 0.2 | 8 | 5 | 0.7 | 90.94 |
| 0.5 | 0.2 | 8 | 5 | 0.8 | 91.19 |
| 0.5 | 0.2 | 9 | 3 | 0.6 | 92.13 |
| 0.5 | 0.2 | 9 | 3 | 0.7 | 91.47 |
| 0.5 | 0.2 | 9 | 3 | 0.8 | 90.83 |
| 0.5 | 0.2 | 9 | 4 | 0.6 | 90.92 |
| 0.5 | 0.2 | 9 | 4 | 0.7 | 92.14 |
| 0.5 | 0.2 | 9 | 4 | 0.8 | 90.94 |
| 0.5 | 0.2 | 9 | 5 | 0.6 | 91.78 |
| 0.5 | 0.2 | 9 | 5 | 0.7 | 92.62 |
| 0.5 | 0.2 | 9 | 5 | 0.8 | 93.32 |
| 0.5 | 0.3 | 7 | 3 | 0.6 | 91.92 |
| 0.5 | 0.3 | 7 | 3 | 0.7 | 90.62 |
| 0.5 | 0.3 | 7 | 3 | 0.8 | 90.99 |
| 0.5 | 0.3 | 7 | 4 | 0.6 | 92.58 |
| 0.5 | 0.3 | 7 | 4 | 0.7 | 92.07 |
| 0.5 | 0.3 | 7 | 4 | 0.8 | 90.98 |
| 0.5 | 0.3 | 7 | 5 | 0.6 | 93.25 |
| 0.5 | 0.3 | 7 | 5 | 0.7 | 92.13 |
| 0.5 | 0.3 | 7 | 5 | 0.8 | 92.23 |
| 0.5 | 0.3 | 8 | 3 | 0.6 | 90.28 |
| 0.5 | 0.3 | 8 | 3 | 0.7 | 91.67 |
| 0.5 | 0.3 | 8 | 3 | 0.8 | 91.21 |
| 0.5 | 0.3 | 8 | 4 | 0.6 | 91.93 |
| 0.5 | 0.3 | 8 | 4 | 0.7 | 91.47 |
| 0.5 | 0.3 | 8 | 4 | 0.8 | 92.85 |
| 0.5 | 0.3 | 8 | 5 | 0.6 | 92.38 |
| 0.5 | 0.3 | 8 | 5 | 0.7 | 91.10 |
| 0.5 | 0.3 | 8 | 5 | 0.8 | 91.47 |
| 0.5 | 0.3 | 9 | 3 | 0.6 | 92.93 |
| 0.5 | 0.3 | 9 | 3 | 0.7 | 93.09 |
| 0.5 | 0.3 | 9 | 3 | 0.8 | 92.56 |
| 0.5 | 0.3 | 9 | 4 | 0.6 | 92.82 |
| 0.5 | 0.3 | 9 | 4 | 0.7 | 92.99 |
| 0.5 | 0.3 | 9 | 4 | 0.8 | 91.46 |
| 0.5 | 0.3 | 9 | 5 | 0.6 | 91.63 |
| 0.5 | 0.3 | 9 | 5 | 0.7 | 92.34 |
| 0.5 | 0.3 | 9 | 5 | 0.8 | 91.42 |
| 0.6 | 0.1 | 7 | 3 | 0.6 | 92.43 |
| 0.6 | 0.1 | 7 | 3 | 0.7 | 92.06 |
| 0.6 | 0.1 | 7 | 3 | 0.8 | 91.31 |
| 0.6 | 0.1 | 7 | 4 | 0.6 | 91.40 |
| 0.6 | 0.1 | 7 | 4 | 0.7 | 91.71 |
| 0.6 | 0.1 | 7 | 4 | 0.8 | 92.76 |
| 0.6 | 0.1 | 7 | 5 | 0.6 | 90.33 |
| 0.6 | 0.1 | 7 | 5 | 0.7 | 92.07 |
| 0.6 | 0.1 | 7 | 5 | 0.8 | 91.81 |
| 0.6 | 0.1 | 8 | 3 | 0.6 | 92.18 |
| 0.6 | 0.1 | 8 | 3 | 0.7 | 91.29 |
| 0.6 | 0.1 | 8 | 3 | 0.8 | 92.72 |
| 0.6 | 0.1 | 8 | 4 | 0.6 | 91.88 |
| 0.6 | 0.1 | 8 | 4 | 0.7 | 93.32 |
| 0.6 | 0.1 | 8 | 4 | 0.8 | 93.40 |
| 0.6 | 0.1 | 8 | 5 | 0.6 | 92.82 |
| 0.6 | 0.1 | 8 | 5 | 0.7 | 90.08 |
| 0.6 | 0.1 | 8 | 5 | 0.8 | 91.10 |
| 0.6 | 0.1 | 9 | 3 | 0.6 | 90.60 |
| 0.6 | 0.1 | 9 | 3 | 0.7 | 90.95 |
| 0.6 | 0.1 | 9 | 3 | 0.8 | 91.14 |
| 0.6 | 0.1 | 9 | 4 | 0.6 | 93.38 |
| 0.6 | 0.1 | 9 | 4 | 0.7 | 92.61 |
| 0.6 | 0.1 | 9 | 4 | 0.8 | 91.89 |
| 0.6 | 0.1 | 9 | 5 | 0.6 | 92.73 |
| 0.6 | 0.1 | 9 | 5 | 0.7 | 92.37 |
| 0.6 | 0.1 | 9 | 5 | 0.8 | 93.00 |
| 0.6 | 0.2 | 7 | 3 | 0.6 | 92.20 |
| 0.6 | 0.2 | 7 | 3 | 0.7 | 91.63 |
| 0.6 | 0.2 | 7 | 3 | 0.8 | 92.31 |
| 0.6 | 0.2 | 7 | 4 | 0.6 | 92.80 |
| 0.6 | 0.2 | 7 | 4 | 0.7 | 92.58 |
| 0.6 | 0.2 | 7 | 4 | 0.8 | 92.48 |
| 0.6 | 0.2 | 7 | 5 | 0.6 | 92.46 |
| 0.6 | 0.2 | 7 | 5 | 0.7 | 93.20 |
| 0.6 | 0.2 | 7 | 5 | 0.8 | 91.06 |
| 0.6 | 0.2 | 8 | 3 | 0.6 | 92.98 |
| 0.6 | 0.2 | 8 | 3 | 0.7 | 91.00 |
| 0.6 | 0.2 | 8 | 3 | 0.8 | 90.06 |
| 0.6 | 0.2 | 8 | 4 | 0.6 | 90.81 |
| 0.6 | 0.2 | 8 | 4 | 0.7 | 90.77 |
| 0.6 | 0.2 | 8 | 4 | 0.8 | 93.44 |
| 0.6 | 0.2 | 8 | 5 | 0.6 | 90.90 |
| 0.6 | 0.2 | 8 | 5 | 0.7 | 92.38 |
| 0.6 | 0.2 | 8 | 5 | 0.8 | 90.24 |
| 0.6 | 0.2 | 9 | 3 | 0.6 | 92.03 |
| 0.6 | 0.2 | 9 | 3 | 0.7 | 92.06 |
| 0.6 | 0.2 | 9 | 3 | 0.8 | 90.15 |
| 0.6 | 0.2 | 9 | 4 | 0.6 | 90.65 |
| 0.6 | 0.2 | 9 | 4 | 0.7 | 91.76 |
| 0.6 | 0.2 | 9 | 4 | 0.8 | 91.80 |
| 0.6 | 0.2 | 9 | 5 | 0.6 | 90.44 |
| 0.6 | 0.2 | 9 | 5 | 0.7 | 90.68 |
| 0.6 | 0.2 | 9 | 5 | 0.8 | 91.50 |
| 0.6 | 0.3 | 7 | 3 | 0.6 | 90.85 |
| 0.6 | 0.3 | 7 | 3 | 0.7 | 91.79 |
| 0.6 | 0.3 | 7 | 3 | 0.8 | 93.37 |
| 0.6 | 0.3 | 7 | 4 | 0.6 | 91.80 |
| 0.6 | 0.3 | 7 | 4 | 0.7 | 91.39 |
| 0.6 | 0.3 | 7 | 4 | 0.8 | 90.30 |
| 0.6 | 0.3 | 7 | 5 | 0.6 | 91.29 |
| 0.6 | 0.3 | 7 | 5 | 0.7 | 92.21 |
| 0.6 | 0.3 | 7 | 5 | 0.8 | 92.87 |
| 0.6 | 0.3 | 8 | 3 | 0.6 | 91.25 |
| 0.6 | 0.3 | 8 | 3 | 0.7 | 90.20 |
| 0.6 | 0.3 | 8 | 3 | 0.8 | 93.08 |
| 0.6 | 0.3 | 8 | 4 | 0.6 | 90.04 |
| 0.6 | 0.3 | 8 | 4 | 0.7 | 90.03 |
| 0.6 | 0.3 | 8 | 4 | 0.8 | 92.45 |
| 0.6 | 0.3 | 8 | 5 | 0.6 | 93.20 |
| 0.6 | 0.3 | 8 | 5 | 0.7 | 90.73 |
| 0.6 | 0.3 | 8 | 5 | 0.8 | 91.85 |
| 0.6 | 0.3 | 9 | 3 | 0.6 | 92.58 |
| 0.6 | 0.3 | 9 | 3 | 0.7 | 90.61 |
| 0.6 | 0.3 | 9 | 3 | 0.8 | 91.52 |
| 0.6 | 0.3 | 9 | 4 | 0.6 | 92.66 |
| 0.6 | 0.3 | 9 | 4 | 0.7 | 90.67 |
| 0.6 | 0.3 | 9 | 4 | 0.8 | 93.31 |
| 0.6 | 0.3 | 9 | 5 | 0.6 | 93.30 |
| 0.6 | 0.3 | 9 | 5 | 0.7 | 90.99 |
| 0.6 | 0.3 | 9 | 5 | 0.8 | 92.17 |
| 0.7 | 0.1 | 7 | 3 | 0.6 | 92.68 |
| 0.7 | 0.1 | 7 | 3 | 0.7 | 91.96 |
| 0.7 | 0.1 | 7 | 3 | 0.8 | 93.23 |
| 0.7 | 0.1 | 7 | 4 | 0.6 | 90.62 |
| 0.7 | 0.1 | 7 | 4 | 0.7 | 90.30 |
| 0.7 | 0.1 | 7 | 4 | 0.8 | 90.43 |
| 0.7 | 0.1 | 7 | 5 | 0.6 | 92.19 |
| 0.7 | 0.1 | 7 | 5 | 0.7 | 92.07 |
| 0.7 | 0.1 | 7 | 5 | 0.8 | 92.24 |
| 0.7 | 0.1 | 8 | 3 | 0.6 | 90.62 |
| 0.7 | 0.1 | 8 | 3 | 0.7 | 91.70 |
| 0.7 | 0.1 | 8 | 3 | 0.8 | 93.07 |
| 0.7 | 0.1 | 8 | 4 | 0.6 | 92.47 |
| 0.7 | 0.1 | 8 | 4 | 0.7 | 90.22 |
| 0.7 | 0.1 | 8 | 4 | 0.8 | 92.59 |
| 0.7 | 0.1 | 8 | 5 | 0.6 | 90.49 |
| 0.7 | 0.1 | 8 | 5 | 0.7 | 90.55 |
| 0.7 | 0.1 | 8 | 5 | 0.8 | 93.30 |
| 0.7 | 0.1 | 9 | 3 | 0.6 | 90.95 |
| 0.7 | 0.1 | 9 | 3 | 0.7 | 91.69 |
| 0.7 | 0.1 | 9 | 3 | 0.8 | 92.30 |
| 0.7 | 0.1 | 9 | 4 | 0.6 | 90.71 |
| 0.7 | 0.1 | 9 | 4 | 0.7 | 93.21 |
| 0.7 | 0.1 | 9 | 4 | 0.8 | 92.82 |
| 0.7 | 0.1 | 9 | 5 | 0.6 | 93.34 |
| 0.7 | 0.1 | 9 | 5 | 0.7 | 93.36 |
| 0.7 | 0.1 | 9 | 5 | 0.8 | 91.83 |
| 0.7 | 0.2 | 7 | 3 | 0.6 | 93.26 |
| 0.7 | 0.2 | 7 | 3 | 0.7 | 93.23 |
| 0.7 | 0.2 | 7 | 3 | 0.8 | 91.69 |
| 0.7 | 0.2 | 7 | 4 | 0.6 | 90.48 |
| 0.7 | 0.2 | 7 | 4 | 0.7 | 92.75 |
| 0.7 | 0.2 | 7 | 4 | 0.8 | 92.35 |
| 0.7 | 0.2 | 7 | 5 | 0.6 | 91.40 |
| 0.7 | 0.2 | 7 | 5 | 0.7 | 93.01 |
| 0.7 | 0.2 | 7 | 5 | 0.8 | 91.15 |
| 0.7 | 0.2 | 8 | 3 | 0.6 | 90.36 |
| 0.7 | 0.2 | 8 | 3 | 0.7 | 91.29 |
| 0.7 | 0.2 | 8 | 3 | 0.8 | 91.76 |
| 0.7 | 0.2 | 8 | 4 | 0.6 | 92.55 |
| 0.7 | 0.2 | 8 | 4 | 0.7 | 91.34 |
| 0.7 | 0.2 | 8 | 4 | 0.8 | 90.43 |
| 0.7 | 0.2 | 8 | 5 | 0.6 | 93.24 |
| 0.7 | 0.2 | 8 | 5 | 0.7 | 92.24 |
| 0.7 | 0.2 | 8 | 5 | 0.8 | 91.81 |
| 0.7 | 0.2 | 9 | 3 | 0.6 | 90.25 |
| 0.7 | 0.2 | 9 | 3 | 0.7 | 91.36 |
| 0.7 | 0.2 | 9 | 3 | 0.8 | 93.24 |
| 0.7 | 0.2 | 9 | 4 | 0.6 | 90.40 |
| 0.7 | 0.2 | 9 | 4 | 0.7 | 93.22 |
| 0.7 | 0.2 | 9 | 4 | 0.8 | 91.46 |
| 0.7 | 0.2 | 9 | 5 | 0.6 | 90.72 |
| 0.7 | 0.2 | 9 | 5 | 0.7 | 90.93 |
| 0.7 | 0.2 | 9 | 5 | 0.8 | 90.77 |
| 0.7 | 0.3 | 7 | 3 | 0.6 | 91.69 |
| 0.7 | 0.3 | 7 | 3 | 0.7 | 92.07 |
| 0.7 | 0.3 | 7 | 3 | 0.8 | 91.39 |
| 0.7 | 0.3 | 7 | 4 | 0.6 | 92.64 |
| 0.7 | 0.3 | 7 | 4 | 0.7 | 92.18 |
| 0.7 | 0.3 | 7 | 4 | 0.8 | 92.01 |
| 0.7 | 0.3 | 7 | 5 | 0.6 | 91.36 |
| 0.7 | 0.3 | 7 | 5 | 0.7 | 92.72 |
| 0.7 | 0.3 | 7 | 5 | 0.8 | 91.07 |
| 0.7 | 0.3 | 8 | 3 | 0.6 | 91.64 |
| 0.7 | 0.3 | 8 | 3 | 0.7 | 91.13 |
| 0.7 | 0.3 | 8 | 3 | 0.8 | 92.29 |
| 0.7 | 0.3 | 8 | 4 | 0.6 | 92.40 |
| 0.7 | 0.3 | 8 | 4 | 0.7 | 92.03 |
| 0.7 | 0.3 | 8 | 4 | 0.8 | 93.11 |
| 0.7 | 0.3 | 8 | 5 | 0.6 | 93.22 |
| 0.7 | 0.3 | 8 | 5 | 0.7 | 92.70 |
| 0.7 | 0.3 | 8 | 5 | 0.8 | 91.21 |
| 0.7 | 0.3 | 9 | 3 | 0.6 | 91.48 |
| 0.7 | 0.3 | 9 | 3 | 0.7 | 91.93 |
| 0.7 | 0.3 | 9 | 3 | 0.8 | 92.84 |
| 0.7 | 0.3 | 9 | 4 | 0.6 | 91.79 |
| 0.7 | 0.3 | 9 | 4 | 0.7 | 92.47 |
| 0.7 | 0.3 | 9 | 4 | 0.8 | 92.21 |
| 0.7 | 0.3 | 9 | 5 | 0.6 | 92.52 |
| 0.7 | 0.3 | 9 | 5 | 0.7 | 92.97 |
| 0.7 | 0.3 | 9 | 5 | 0.8 | 90.51 |

Table S11 Comparison of XGBoost and other widely used machine learning models

| Species | Models | Accuracy (%) | Precision (%) | Recall (%) | F1-score |
| --- | --- | --- | --- | --- | --- |
| Human | XGBoost (optimal parameters) | 93.44 | 93.44 | 93.43 | 0.93 |
|  | XGBoost (default parameters) | 92.31 | 92.14 | 92.09 | 0.92 |
|  | Nearest Neighbors | 73.44 | 79.44 | 73.35 | 0.67 |
|  | AdaBoost | 84.86 | 84.91 | 84.85 | 0.85 |
|  | SVM (linear kenrel) | 80.73 | 80.90 | 80.70 | 0.81 |
|  | SVM (rbf kenrel) | 92.23 | 92.21 | 92.22 | 0.92 |
|  | DecisionTree | 84.57 | 84.53 | 84.55 | 0.85 |
|  | SGD | 80.80 | 80.79 | 80.78 | 0.81 |
|  | Random Forest | 90.53 | 90.56 | 93.51 | 0.90 |
| *Arabidopsis thaliana* | XGBoost (optimal parameters) | 94.09 | 93.46 | 93.35 | 0.93 |
|  | XGBoost (default parameters) | 92.89 | 92.98 | 92.89 | 0.93 |
|  | Nearest Neighbors | 58.64 | 71.14 | 57.63 | 0.50 |
|  | AdaBoost | 90.10 | 90.32 | 90.01 | 0.90 |
|  | SVM (linear kenrel) | 85.02 | 85.08 | 84.98 | 0.85 |
|  | SVM (rbf kenrel) | 91.49 | 91.61 | 91.52 | 0.92 |
|  | DecisionTree | 83.51 | 83.46 | 83.44 | 0.83 |
|  | SGD | 85.10 | 85.09 | 85.07 | 0.85 |
|  | Random Forest | 90.71 | 90.90 | 90.73 | 0.91 |

Table S12 Comparison of AS classification among mkcDBGAS, DeepASmRNA, and AStrap in three datasets

| Datasets | Methods | Models | Accuracy (%) |
| --- | --- | --- | --- |
| The human test dataset in mkcDBGAS | mkcDBGAS | Human | 93.44 |
|  | AStrap | Human | 83.34 |
|  | DeepASmRNA | Human | 88.49 |
| The rice test dataset in DeepASmRNA | mkcDBGAS | *Arabidopsis thaliana* | 94.09 |
|  | AStrap | Rice | 90.11 |
|  | DeepASmRNA | Rice | 90.02 |
| The rice benchmark dataset in AStrap | mkcDBGAS | *Arabidopsis thaliana* | 92.17 |
|  | AStrap | Rice | 88.30 |
|  | DeepASmRNA | Rice | 91.17 |

Table S13 Experimental validation using 34 ES events obtained by RT-PCR from the PC3E cell line in the previous research [1]

| No. | Gene ID | Chr | Strand | Start in the genome | End in the genome | Start in the transcript | End in the transcript | Validation |
| --- | --- | --- | --- | --- | --- | --- | --- | --- |
| 1 | ENSG00000131473 | chr17 | - | 40052872 | 40052902 | 2750 | 2779 | Y |
| 2 | ENSG00000127914 | chr7 | + | 91671359 | 91671500 | 5151 | 5291 | Y |
| 3 | ENSG00000084234 | chr11 | + | 129993506 | 129993674 | 995 | 1162 | Y |
| 4 | ENSG00000140750 | chr16 | - | 24950684 | 24950918 | 1664 | 1897 | Y |
| 5 | ENSG00000196914 | chr11 | + | 120280102 | 120280159 | 309 | 365 | Y |
| 6 | ENSG00000074410 | chr15 | - | 63620296 | 63620329 | 5066 | 5098 | Y |
| 7 | ENSG00000196924 | chrX | - | 153585618 | 153585642 | 3301 | 3324 | Y |
| 8 | ENSG00000115414 | chr2 | - | 216257653 | 216257926 | 4374 | 4646 | Y |
| 9 | ENSG00000134317 | chr2 | + | 10102583 | 10102660 | 670 | 746 | Y |
| 10 | ENSG00000136231 | chr7 | - | 23383336 | 23383472 | 2700 | 2835 | Y |
| 11 | ENSG00000171105 | chr19 | - | 7150507 | 7150543 | 6684 | 6719 | Y |
| 12 | ENSG00000091409 | chr2 | + | 173366499 | 173366629 | 2971 | 3100 | Y |
| 13 | ENSG00000133703 | chr12 | - | 25368370 | 25368494 | 482 | 605 | Y |
| 14 | ENSG00000110514 | chr11 | + | 47310518 | 47310578 | 2819 | 2878 | Y |
| 15 | ENSG00000135341 | chr6 | - | 91254270 | 91254351 |  |  | N |
| 16 | ENSG00000071054 | chr2 | + | 102487955 | 102488147 | 2762 | 2953 | Y |
| 17 | ENSG00000196586 | chr6 | + | 76621388 | 76621415 | 3722 | 3748 | Y |
| 18 | ENSG00000138119 | chr10 | - | 95152673 | 95152712 | 5264 | 5302 | Y |
| 19 | ENSG00000196498 | chr12 | - | 124858958 | 124859009 | 6315 | 6365 | Y |
| 20 | ENSG00000166579 | chr17 | + | 8366637 | 8366672 | 1142 | 1176 | Y |
| 21 | ENSG00000001167 | chr6 | + | 41048549 | 41048636 |  |  | N |
| 22 | ENSG00000070882 | chr7 | - | 24902818 | 24902911 | 5439 | 5531 | Y |
| 23 | ENSG00000197461 | chr7 | - | 540067 | 540136 | 1864 | 1968 | Y |
| 24 | ENSG00000075651 | chr3 | - | 171404474 | 171404588 | 3611 | 3724 | Y |
| 25 | ENSG00000005175 | chr12 | - | 48073275 | 48073377 | 930 | 1031 | Y |
| 26 | ENSG00000065613 | chr10 | + | 105770573 | 105770666 | 3330 | 3422 | Y |
| 27 | ENSG00000135317 | chr6 | - | 86248555 | 86248582 | 1673 | 1699 | Y |
| 28 | ENSG00000008294 | chr17 | - | 49053223 | 49053262 | 1641 | 1679 | Y |
| 29 | ENSG00000163482 | chr2 | + | 219553113 | 219553216 | 276 | 378 | Y |
| 30 | ENSG00000135111 | chr12 | - | 115117717 | 115117777 | 3102 | 3161 | Y |
| 31 | ENSG00000124496 | chr6 | - | 42231057 | 42231306 | 5134 | 5442 | Y |
| 32 | ENSG00000108395 | chr17 | - | 57078958 | 57079075 |  |  | N |
| 33 | ENSG00000138768 | chr4 | + | 76716488 | 76716509 | 1459 | 1479 | Y |
| 34 | ENSG00000152818 | chr6 | + | 145148483 | 145148522 | 9495 | 9533 | Y |

Table S14 Performance of XGBoost using or without 314 motif features

| Model | Dataset |  | ES | AA | AD | IR | Overall |
| --- | --- | --- | --- | --- | --- | --- | --- |
| Human | With motif | Accuracy (%) |  |  |  |  | 93.44 |
|  |  | Precision (%) | 90.80 | 93.28 | 95.22 | 94.47 |  |
|  |  | Recall (%) | 92.01 | 90.54 | 93.45 | 97.71 |  |
|  |  | F1-score | 0.91 | 0.92 | 0.94 | 0.96 |  |
|  |  | True positive | 10865 | 11109 | 11532 | 11412 | 44918 |
|  | Without motif | Accuracy (%) |  |  |  |  | 90.60 |
|  |  | Precision (%) | 83.82 | 92.24 | 93.88 | 93.27 |  |
|  |  | Recall (%) | 91.94 | 85.47 | 89.48 | 95.45 |  |
|  |  | F1-score | 87.69 | 88.73 | 91.63 | 94.35 |  |
|  |  | True positive | 10030 | 10985 | 11370 | 11267 | 43652 |
| *Arabidopsis thaliana* | With motif | Accuracy (%) |  |  |  |  | 94.09 |
|  |  | Precision (%) | 96.63 | 95.32 | 94.23 | 90.60 |  |
|  |  | Recall (%) | 95.24 | 92.16 | 91.43 | 97.52 |  |
|  |  | F1-score | 0.96 | 0.94 | 0.93 | 0.94 |  |
|  |  | True positive | 1688 | 1752 | 1683 | 1649 | 6772 |
|  | Without motif | Accuracy (%) |  |  |  |  | 90.81 |
|  |  | Precision (%) | 93.03 | 92.57 | 92.66 | 85.88 |  |
|  |  | Recall (%) | 93.99 | 87.49 | 85.55 | 96.26 |  |
|  |  | F1-score | 93.51 | 89.96 | 88.97 | 90.78 |  |
|  |  | True positive | 1625 | 1701 | 1655 | 1563 | 6544 |

Table S15 The top 20 ΔPSI with *p*_value less than 0.05 obtained by MkcDBGAS between the PC3E and GS689 cell line

| Num. | Transcript ID | ΔPSI | *p*-value |
| --- | --- | --- | --- |
| 1 | ENST00000370660.3 | 0.83176303 | 0.000504496 |
| 2 | ENST00000621269.4 | 0.779329422 | 0 |
| 3 | ENST00000449475.6 | 0.588613883 | 0.002017982 |
| 4 | ENST00000378784.8 | 0.569803472 | 0.002017982 |
| 5 | ENST00000679056.1 | 0.332930729 | 0 |
| 6 | ENST00000629496.3 | 0.323488907 | 0.002017982 |
| 7 | ENST00000642669.1 | 0.318853019 | 0.001008991 |
| 8 | ENST00000676489.1 | 0.313201862 | 0.00454046 |
| 9 | ENST00000353676.9 | 0.306454975 | 0 |
| 10 | ENST00000439661.5 | 0.296430256 | 0.001008991 |
| 11 | ENST00000374006.1 | 0.283079087 | 0.018161838 |
| 12 | ENST00000680063.1 | 0.280508921 | 0.016143856 |
| 13 | ENST00000603808.5 | 0.265465111 | 0.014125874 |
| 14 | ENST00000565385.5 | 0.264696713 | 0.005044955 |
| 15 | ENST00000592894.5 | 0.26228015 | 0.091818182 |
| 16 | ENST00000256379.10 | 0.251917319 | 0.005549451 |
| 17 | ENST00000426173.6 | 0.241598286 | 0.013116883 |
| 18 | ENST00000615182.5 | 0.241408474 | 0.001008991 |
| 19 | ENST00000379850.7 | 0.230464727 | 0.005044955 |
| 20 | ENST00000357008.7 | 0.226803983 | 0.001513487 |

Reference

1. Shen S, Park JW, Lu ZX et al. rMATS: robust and flexible detection of differential alternative splicing from replicate RNA-Seq data, Proc Natl Acad Sci U S A 2014;111:E5593-5601.
