# Supplementary material for: MkcDBGAS: a reference-free approach to identify comprehensive alternative splicing events in a transcriptome
Source: Brief Bioinform. 2023 Oct 13;24(6):bbad367. doi: 10.1093/bib/bbad367 (PMC10576019; doi:10.1093/bib/bbad367)
Supplement: Supplementary_Methods_bbad367 [file supplementary_methods_bbad367.docx]

**MkcDBGAS: a reference-free approach to identify comprehensive alternative splicing events in a transcriptome**

Quanbao Zhang^1^, Lei Cao^1^, Hongtao Song^1^, Kui Lin^1^ and Erli Pang^1*^

^1^ MOE Key Laboratory for Biodiversity Science and Ecological Engineering and Beijing Key Laboratory of Gene Resource and Molecular Development, College of Life Sciences, Beijing Normal University, Beijing 100875, China

*To whom correspondence should be addressed

Erli Pang, College of Life Sciences, Beijing Normal University, No 19 Xinjiekouwai Street, Beijing, 100875, China

Email: [pangerli@bnu.edu.cn](mailto:pangerli@bnu.edu.cn)

Supplementary Data Index

[SUPPLEMENTARY FIGURES 3](#_Toc134176256)

[SUPPLEMENTARY ALGORITHMS 4](#_Toc134176257)

[SUPPLEMENTARY METHODS 7](#_Toc134176258)

[REFERENCES 10](#_Toc134176259)

# Supplementary Figures


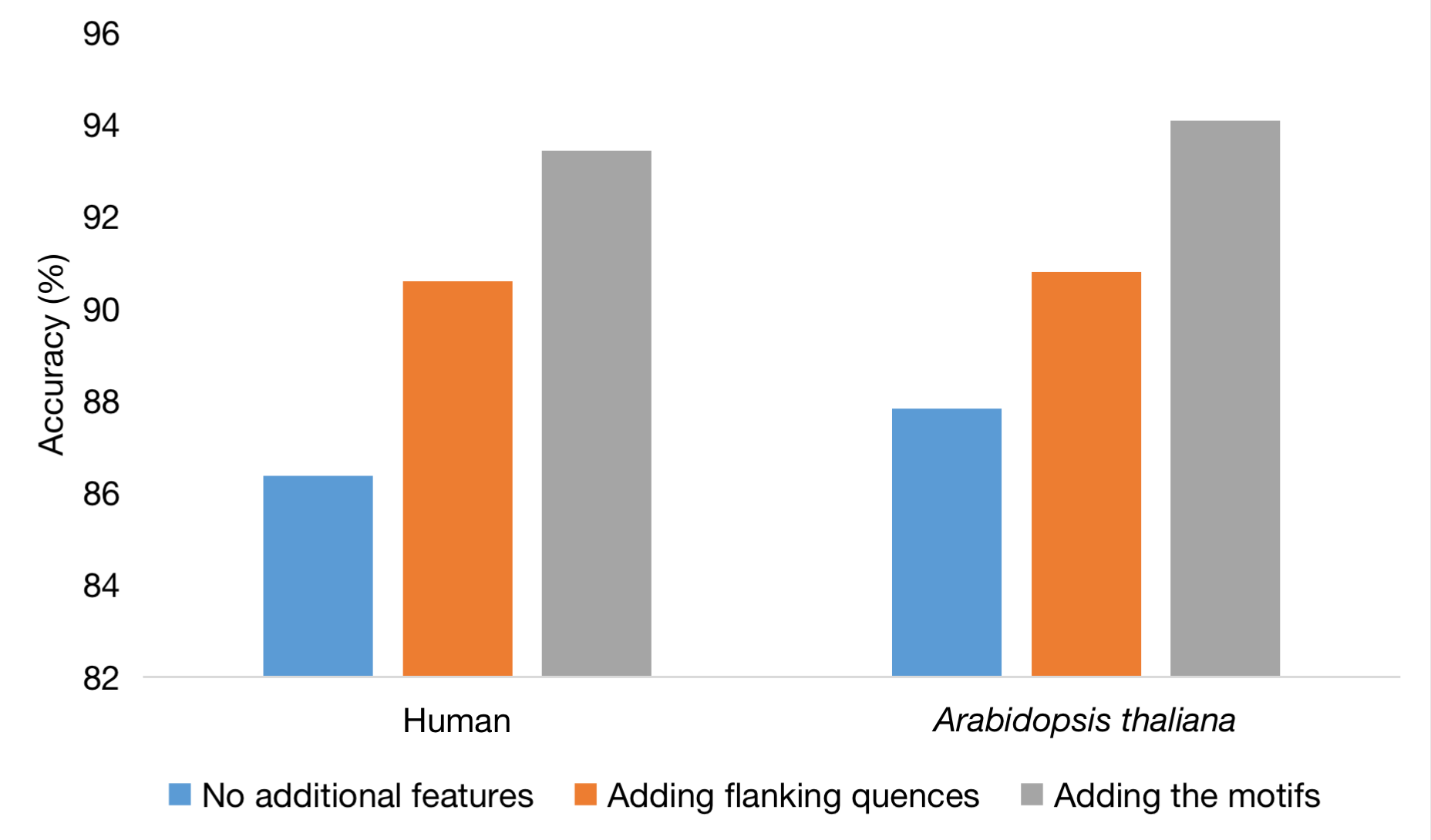


Figure S1 Performance comparison of MkcDBGAS before and after the addition of features. New features in MkcDBGAS mainly included: 1) flanking sequences (the upstream and downstream) of AS regions, and 2) motifs representing functional features.

# Supplementary Algorithms

## Algorithm S1: Obtaining the length of non-repeat sequences as k-mer

Instead of setting a fixed k value, we set the most suitable k value for each transcript sequence to construct colored de Bruijn graphs (cDBGs) [1]. The most suitable *k* was determined (Algorithm S1) as the length of the shortest nonrepeat sequences in the two transcript sequences to ensure that there was no loop in the cDBG.

| **Algorithm S1:** Obtaining the length of non-repeat sequences as *k*  **Input：** sequence  **Output：**k  **function min_k**(seq,k,flag,position)  1 pos <- position  2 **if** flag = 1 **then**  3 **for** i = pos to length(seq) **do** #From the position k  4 **if** seq.count(seq[i : i+k]) > 1 **then** #The *k*-mer started at i is not unique  5 flag <- 1  6 pos <- i  7 break  8 **else**  9 flag <- 0 #This *k*-mer is unique  10 **end for**  11 k = **min_k**(seq,k+1,flag,pos) #Start to iterate  12 **return** k  13 **else** #Flag is 0 until the whole sequence  14 **return** k |
| --- |

## Algorithm S2: Constructing cDBG for two transcript sequences

A cDBG was constructed using the *k-*mers by Algorithm S2. Meanwhile, the string "Q" with a length of *k* was added to the start, and the string "P" with a length of *k* was added to the end of the two transcript sequences to ensure that the cDBGs had a source node and a sink node.

| **Algorithm S2**: Constructing cDBG for two transcript sequences  **Input**： two transcript sequences  **Output**：cDBG: G={V,E,C}  **function construct**(seq1,seq2)  1 k1 <- **min_k**(seq1)  2 k2 <- **min_k**(seq2)  3 k <- **max**(k1, k2) -1 #Determining k  4 head = ”Q” * k  5 tail = ”P” * k  6 seq1 = head + seq1 +tail #Add head and tail to sequence  7 seq2 = head + seq2 +tail  8 **for** i in 1 : length(seq1)-k **do** #Decompose seq1 into *k*-mers  9 k-mer <- seq1[i:i+k]  10 V [k-mer] <- {position1: i,  length1:k,  sequence: k-mer,  color1: seq1}  11 E [k-mer] <- { position: i,  parent: last v,  son: next v}  12 C [k-mer] <- {color1: seq1  13 **end for**  **14 for** i in 0 : length(seq2)-k **do** #Decompose seq2 into *k*-mers  15 k-mer <- seq1[i:i+k]  16 V [k-mer] <- {position2: i,  length:k,  sequence: k-mer,  color2: seq1}  17 E [k-mer] <- { position2: i,  parent2: last v,  son2: next v}  18 C [k-mer] <- { position2: i,  color2: seq1}  19 **end for**  20 **return** G=(V, E, C) |
| --- |

## Algorithm S3: Calling other-induced bubbles and reconstructing sub-cDBGs with k'-mers

For each other-induced bubble, the two sequences of arms are used to reconstruct the sub-cDBG with *k'*-mers (Algorithm S3). Thus, the sub-cDBGs with *k'*-mers were obtained.

| **Algorithm S3**: Calling other-induced bubbles and reconstructing sub-cDBGs with *k'*-mers  **Input** G = (V, E, C)  **Out** G' = (V', K, E', C'),  initial SN is a set of supernodes  initial Bubble a is set of bubble  **function reconstruct**(G)  1 **for** *k*-mer **in** V **do** #Find suppernodes  2 **if** parent1≠ parent2 **then**  3 SN[*k*-mer]={ in_degree: 2,  type: source node}  4 **if** son1≠ son2 **then**  5 SN[*k*-mer]={ out_degree: 2,  type: sink node}  6 **for** a pair of source nodes and sink node **do** #Calling bubbles  7 **if** only two paths between the pair **then**  8 Bubble ={arm1_length: length of path1,  9 arm2_length: length of path2}  10 **for** each BB **in** Bubble **do**  11 **if** arm1_length > k and arm2_length >k **then**  12 seq1 = sequence in arm1  13 seq2 = sequence in arm2  14 sub_cDBG=**construct**(seq1seq2) #Applying Algorithm S2  15 replacing sub_cDBG with all nodes in BB  16 recording *k*-mer of sub_cDBG  17 **return** G' = (V', K, E', C') |
| --- |

## Algorithm S4: Identifying AS transcript pairs

To detect AS events, we tracked the cDBG and queried AS-induced bubbles in the graph (Algorithm S4). If there were AS-induced bubbles in the cDBG with mixed *k*-mers, we identified the transcript pair as alternatively spliced transcripts.

| **Algorithm S4**: Identifying AS transcript pairs  **Input** two transcript sequences  **Out** AS= a set of AS event  **function identifying**(seq, seq2)  1 G = **construct**(seq1, seq2) #Constructing cCDBG  2 G' = **reconstruct**(G) #Reconstructing sub_cCDBG  3 Bubbles =all bubbles in G' #Get all of the bubbles  4 **if** Bubbles number = 1 **then** #Identifying AF, AL, and MX  5 **if** arm1_length > k **and** arm2_length > k **then**  6 **if** length of arm > 30% length seq1 and seq2 **then**  7 **if** bubble at the start of seq1 and seq2 **then**  8 **return** AF  9  **if** bubble at the end of seq1 and seq2 **then**  10 **return** AL  11 **if** bubble at the end of seq1 and seq2 **then**  12 **return** MX  11 **for** Bubble **in** Bubbles **do**  12 snv_number = 0  13 as_number = 1  14 **if** arm1_length = k **and** arm2_length = k **then** #Identifying SNV  15 snv_number + 1  16 **if** arm1_length = k-1 **and** arm2_length > k+1 **then** #Identifying unclassified  17 AS[as_number] = { start position in seq1, four types AS event  end position in seq1,  start position in seq2,  end position in seq2}  18 **if** snv_number < 2 **then**  19 **return** AS |
| --- |

# Supplementary Methods

## S1.1 Calculating PSI values and analysis of differential splicing across multiple biological conditions

When RNA-Seq data is available, we further provide to analyze differential splcing. This module included three steps. First, transcripts that came from the same gene were clustered. AS transcript pairs were obtained by inputting the full-length transcript into MkcDBG. Based on transcript pairs, transcripts that came from the same gene were clustered. Second, the subfunction “psiPerIsoform” in SUPPA2 [2] was integrated to calculate and normalize percent-spliced-in (PSI) values of transcripts based on the expression levels of transcripts. Third, the subfunction “diffSplice” in SUPPA2 [2] was integrated to calculate the differential splicing. MkcDBG put out a dpsi (ΔPSI ) file, including a differential transcript between two conditions and their corresponding *p*-value.

## S1.2 Construct the feature matrix of AS classification model

To extract features, for each AS event, we named upstream n bp as “upnbp”, alternative region as “as”, and downstream n bp as “downnbp”. First, we obtained 14 different continuous sequences around the splicing site according to their coordinates in the transcripts: up50bp, as, down50bp, up50bp+as, up50bp+down50bp, as+down50bp, up50bp+as+down50bp, up30bp+as30bp, up30bp+down30bp, as30bp+down30bp, up10bp, up20bp, down10bp, and down20bp. Then, we calculated features of the 14 sequences, including sequence features such as GC content, whether the length of the sequence is divisible by three, frequency of nucleotides, frequency of dinucleotides, frequency of trinucleotides, and the number of the stop codons; structure features such as the length of the alternative region; whether there were patterns including GT, GC, AT, AG, and AC in the donors or acceptors; the distribution of each nucleotide (A, T, C, and G) [3]; and functional features such as information whether the sequence contained 314 motifs [4] including 156 distributed in the up50bp+as and 158 distributed in the as+down50bp (Supplementary Table S1).

## S1.3 XGBoost a novel sparsity-aware algorithm

XGBoost was adapted in mkcDBGAS to handle the motif features that are usually quite sparse. XGBoost is a novel sparsity-aware algorithm and gives state-of-the-art results on a wide range of problems including classification [5]. We used a tree ensemble model of XGBoost to build the classification models in mkcDBGAS, which used the feature matrix as input and obtained its best gradient regression tree through training. For a given training dataset with n samples and m features $D=\left\{ \left( x_{i},y_{i} \right) \right\} (\left| D \right|=n, x_{i} \in R^{m}, y_{i} \in R)$, XGBoost uses T decision trees, $f_{j}$represent the *j*-th decision tree, the feature vector is regarded as input$x_{i}$, and the predicted output $\hat{y}_{i}= \sum_{j=1}^{T} f_{j}\left( x_{i} \right)$, where T is the number of decision trees, and $f_{j}\left( x_{i} \right)$ is the prediction score of the *j*-th decision tree for the *i*-th sample. To avoid overfitting, the XGBoost adds a new function $\Omega\left( f_{j} \right)= \gamma T+ \frac{1}{2}ℷ\sum_{j=1}^{T} {\varpi_{j}}^{2}$ to control the complexity of the *j*-th subtree, where $\varpi_{j}$ is the score on the *j*-th leaf node, and $\gamma$ and $ℷ$ are the hyperparameters that control the complexity of the model.

## S1.4 The grid search approach in training classification models of AS events

To optimize the hyperparameters of the XGBoost classifier, a grid search approach was performed by the GridSearchCV function in scikit-learn [6]. We selected values from the following parameters: colsample bytree∈{0.5, 0.6, 0.7}, learning_rate∈{0.1, 0.2, 0.3}, max_depth∈{7, 8, 9}, min_child_weight∈{3, 4, 5}, subsample∈{0.6, 0.7, 0.8}. We traversed the combinations of the parameters and determined the hyperparameters by 10-fold inner cross-validation. The parameter selection was based on the accuracy of the model. The optimal combinations of the parameters for human and *Arabidopsis thaliana* were selected when the accuracy reached the largest values.

## S1.5 Full-length transcripts assembly

To apply the differential splicing module of MkcDBGAS, we used the RNA-Seq datasets from rMATS [7]. We downloaded the RNA-Seq datasets of PC3E and GS689 from rMATS [7], respectively.There were there replicates for each datastet. Trimmomatic [8] was used for trimming low-quality bases with default parameters.The high-quality reads were mapped to the human reference genome (GRCh38) using STAR (v2.7.10b) [9] with --twopassMode Basic, --sjdbOverhang 100, --outSAMunmapped None, and --outSAMtype BAM SortedByCoordinate. Then, StringTie [10] was used for assembling alignments files generated by STAR. We assembled the transcripts for each sample. Thus, we obtained six datasets of full-length transcripts.

## S1.6 Implementation of differential splicing

To apply the differential splicing module, the six datasets of full-length transcripts from PC3E and GS689 cell line were merged by Stringtie —merge. In total, we obtained 78,429 full-length transcripts. Using RSEM [11], we obtained expression levels of the transcripts in PC3E and GS689, respectively.

The two transcript expression matrixes were put into the differential splicing module of MkcDBGAS (see S1.1). The module generated a dpsi (ΔPSI ) file, including a differential transcript between two conditions and their corresponding *p*-value. Finally, a total of 5,783 transcripts with *p*-value less than 0.05 were obtained, of which top 20 were shown in Supplementary Table S15.

## S1.7 Validation using the AS dataset obtained by rMATS

First, we used RNA-Seq data from PC3E cell line to obtain the AS events by rMATS. The alignments file obtained by STAR (see S1.5) and the gtf annotation file generated by StringTie were put into rMATS [7] (v4.1.2) to detect splicing events with the following parameters: --readLength 101, -t paired, and --variable-read-length. rMATS focused on five types of AS events and predicted 164,286 AS events, including 108,221 ES, 13,256 AA, 10,825 AD, 9,801 IR, and 22,183 MXE, involving 431,840 transcript pairs.

Second, we obtained the AS events by MkcDBGAS. Transcripts from the three replications of PC3E (see S1.5) were merged into a dataset of full-length transcripts. Then, the full-length transcripts served as input to MkcDBGAS for identifying AS and classifying AS using the human model. MkcDBGAS identified 391,517 transcript pairs and 156,568 AS events, including 114,291 ES, 11,478 AA, 10,217 AD, 6,589 IR, 12,137 AF, 10,743AL, and 7,823 MX.

Using the AS events obtained by rMATS as the ground truth, the true positive dataset included 387,445 transcript pairs. The precision of MkcDBGAS in the identification was 98.96% and the recall was 89.72%. To evaluate the classification, according to the mechanisms, AF, AL, and MX events in MkcDBGAS were treated as MXE in rMATS. We used the human model in MkcDBGAS to classify the four types of AS events generated by MkcDBGAS and validated the AS event types using the AS set obtained by rMATS. The overall accuracy is 91.73%. These results indicated that MkcDBGAS performs well in the prediction of AS.

# References

1. Iqbal Z, Caccamo M, Turner I et al. De novo assembly and genotyping of variants using colored de Bruijn graphs, Nature Genetics 2012;44:226-232.

2. Trincado JL, Entizne JC, Hysenaj G et al. SUPPA2: fast, accurate, and uncertainty-aware differential splicing analysis across multiple conditions, Genome Biology 2018;19:40.

3. Cai CZ, Han LY, Ji ZL et al. SVM-Prot: web-based support vector machine software for functional classification of a protein from its primary sequence, Nuclc Acids Research 2003:3692-3697.

4. Yeo GW, Van Nostrand EL, Liang TY. Discovery and analysis of evolutionarily conserved intronic splicing regulatory elements, PLoS Genetics 2007;3:e85.

5. Chen TQ, Guestrin C. XGBoost: a scalable tree boosting system, In 22nd SIGKDD Conference on Knowledge Discovery and Data Mining 2016:785-794.

6. Swami A, Jain R. Scikit-learn: machine learning in python, Journal of Machine Learning Research 2013;12:2825-2830.

7. Shen S, Park JW, Lu ZX et al. rMATS: robust and flexible detection of differential alternative splicing from replicate RNA-Seq data, Proceedings of the National Academy of Sciences of the United States of America 2014;111:E5593-5601.

8. Bolger AM, Lohse M, Usadel B. Trimmomatic: a flexible trimmer for Illumina sequence data, Bioinformatics 2014;30:2114-2120.

9. Dobin A, Davis CA, Schlesinger F et al. STAR: ultrafast universal RNA-seq aligner, Bioinformatics 2013;29:15-21.

10. Pertea M, Pertea GM, Antonescu CM et al. StringTie enables improved reconstruction of a transcriptome from RNA-seq reads, Nature Biotechnology 2015;33:290-+.

11. Li B, Dewey CN. RSEM: accurate transcript quantification from RNA-Seq data with or without a reference genome, BMC Bioinformatics 2011;12:323.
